# Low frequency of paleoviral infiltration across the avian phylogeny

**DOI:** 10.1186/s13059-014-0539-3

**Published:** 2014-12-11

**Authors:** Jie Cui, Wei Zhao, Zhiyong Huang, Erich D Jarvis, M Thomas P Gilbert, Peter J Walker, Edward C Holmes, Guojie Zhang

**Affiliations:** Marie Bashir Institute for Infectious Diseases and Biosecurity, Charles Perkins Centre, School of Biological Sciences and Sydney Medical School, The University of Sydney, Sydney, NSW 2006 Australia; China National GeneBank, BGI-Shenzhen, Shenzhen, 518083 China; Howard Hughes Medical Institute, Duke University Medical Center, Department of Neurobiology, Box 3209, Durham, North Carolina 27710 USA; Centre for GeoGenetics, Natural History Museum of Denmark, University of Copenhagen, Øster Voldgade 5-7, DK-1350 Copenhagen, Denmark; CSIRO Animal, Food and Health Sciences, Australian Animal Health Laboratory, Geelong, Victoria 3220 Australia; Centre for Social Evolution, Department of Biology, University of Copenhagen, Universitetsparken 15, DK-2100 Copenhagen, Denmark; Trace and Environmental DNA Laboratory, Department of Environment and Agriculture, Curtin University, Perth, Western Australia 6102 Australia; Program in Emerging Infectious Diseases, Duke-NUS Graduate Medical School, Singapore, 169857 Singapore

## Abstract

**Background:**

Mammalian genomes commonly harbor endogenous viral elements. Due to a lack of comparable genome-scale sequence data, far less is known about endogenous viral elements in avian species, even though their small genomes may enable important insights into the patterns and processes of endogenous viral element evolution.

**Results:**

Through a systematic screening of the genomes of 48 species sampled across the avian phylogeny we reveal that birds harbor a limited number of endogenous viral elements compared to mammals, with only five viral families observed: Retroviridae, Hepadnaviridae, Bornaviridae, Circoviridae, and Parvoviridae. All nonretroviral endogenous viral elements are present at low copy numbers and in few species, with only endogenous hepadnaviruses widely distributed, although these have been purged in some cases. We also provide the first evidence for endogenous bornaviruses and circoviruses in avian genomes, although at very low copy numbers. A comparative analysis of vertebrate genomes revealed a simple linear relationship between endogenous viral element abundance and host genome size, such that the occurrence of endogenous viral elements in bird genomes is 6- to 13-fold less frequent than in mammals.

**Conclusions:**

These results reveal that avian genomes harbor relatively small numbers of endogenous viruses, particularly those derived from RNA viruses, and hence are either less susceptible to viral invasions or purge them more effectively.

**Electronic supplementary material:**

The online version of this article (doi:10.1186/s13059-014-0539-3) contains supplementary material, which is available to authorized users.

## Background

Vertebrate genomes commonly harbor retrovirus-like [1] and non-retrovirus-like [2] viral sequences, resulting from past chromosomal integration of viral DNA (or DNA copies of viral RNA) into host germ cells. Tracing the evolutionary histories of these endogenous viral elements (EVEs) can provide important information on the origin of their extant counterparts, and provide an insight into host genome dynamics [3-7]. Recent studies have shown that these genomic ‘fossils’ can also influence the biology of their hosts, both beneficially and detrimentally; for example, by introducing novel genomic rearrangements, influencing host gene expression, as well as evolving into new protein-coding genes with cellular functions (that is, ‘gene domestication’) [4,6].

Because integration into host genomes is intrinsic to the replication cycle of retroviruses which employ reverse transcriptase (RT), it is no surprise that retroviruses are commonly found to have endogenous forms in a wide range of animal genomes [8]. Indeed, most of the EVEs present in animal genomes are of retroviral origin - endogenous retroviruses (ERVs) - and EVEs representing all retroviral genera, with the exception of *Deltaretrovirus*, have been found to possess endogenous forms. Remarkably, recent studies have revealed the unexpected occurrence of non-retroviral elements in various animal genomes, including RNA viruses that lack a DNA form in their replication cycle [2,6]. Since their initial discovery, EVEs in animal genomes have been documented for families of double-stranded (ds)DNA viruses (virus classification Group I) - Herpesviridae; single-stranded (ss)DNA viruses (Group II) - Circoviridae and Parvoviridae; ssRNA viruses (Group IV) - Bornaviridae and Filoviridae; ssRNA-RT viruses (Group VI) - Retroviridae; and dsDNA-RT viruses (Group VII) - Hepadnaviridae [6].

To date, most studies of animal EVEs have focused on mammals due to their relatively high density of sampling. In contrast, few studies on the EVEs present in avian species have been undertaken. The best-documented avian EVEs are endogenous hepadnaviruses. These virally derived elements were first described in the genome of a passerine bird - the zebra finch [9] - and then in the genome of the budgerigar [10] as well as some other passerines [11], and may have a Mesozoic origin in some cases [11]. Also of note was the discovery of a great diversity of ERVs in the genomes of zebra finch, chicken and turkey, most of which remain transcriptionally active [12]. In contrast, most mammalian ERVs are inert.

In this study, we systematically mined 48 avian genomes for EVEs of all viral families, as one of a body of companion studies on avian genomics [13,14]. Importantly, our data set represents all 32 neognath and two of the five palaeognath orders, and thus represents nearly all major orders of extant birds. Such a large-scale data analysis enabled us to address a number of key questions in EVE evolution, namely (i) what types of viruses have left such genomic fossils across the avian phylogeny and in what frequencies, (ii) what are the respective frequencies of EVE inheritance between species and independent species-specific insertion, and (iii) what is the frequency and pattern of avian EVE infiltration compared with other vertebrates?

## Results

### Genome scanning for avian endogenous viral elements

**Figure 1:**
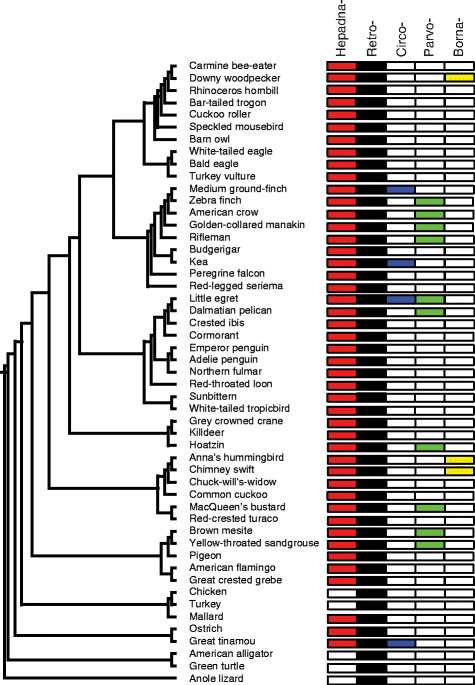
**Distribution of endogenous viral elements of all virus families across the avian phylogeny.** EVEs are colored according to virus family and marked on the species tree. Colors are as follows: red, Hepadnaviridae; black, Retroviridae; blue, Circoviridae; green, Parvoviridae; and yellow, Bornaviridae. The phylogeny is based on the results of our phylogenomics consortium whole genome analyses across all the species shown.

Our *in silico* genomic mining of the 48 avian genomes [13,14] (Table S1 in Additional file 1) revealed the presence of five families of endogenous viruses - Retroviridae, Hepadnaviridae, Circoviridae, Parvoviridae, and Bornaviridae (Figure 1), almost all of which (>99.99%) were of retroviral origin. Only a single family of RNA viruses (Group IV; the Bornaviridae) was present. Notably, three closely related oscine passerine birds - the American crow, medium ground-finch and zebra finch - possessed greater ERV copy numbers in their genomes than the avian average (Table 1; discussed in detail below), while their suboscine passerine relatives - rifleman and golden-collared manakin - possessed lower ERV numbers close to the avian average (Table 1) and occupied basal positions in the passerine phylogeny (Figure 1). Hence, there appears to have been an expansion of ERVs coincident with the species radiation of the suborder Passeri.

**Table 1:** **Endogenous viral element copy numbers in avian genomes**

**Species name**	**Hepadna-**	**Borna-**	**Circo-**	**Parvo-**	**Retroviral copy number**					
					**Total**	**Alpha-**	**Beta-**	**Gamma-**	**Epsilon-**	**Others** ^**a**^
*Acanthisitta chloris*	2	0	0	1	302	8	111	160	9	14
*Anas platyrhynchos*	4	0	0	0	281	7	54	186	17	17
*Antrostomus carolinensis*	2	0	0	0	246	15	76	119	16	20
*Apaloderma vittatum*	2	0	0	0	258	10	97	130	11	10
*Aptenodytes forsteri*	2	0	0	0	232	11	80	104	12	25
*Balearica regulorum*	2	0	0	0	244	13	65	113	23	30
*Buceros rhinoceros*	3	0	0	0	217	9	59	113	12	24
*Calypte anna*	3	4	0	0	424	27	181	157	17	42
*Cariama cristata*	3	0	0	0	315	13	78	176	20	28
*Cathartes aura*	2	0	0	0	199	11	33	115	11	29
*Chaetura pelagica*	2	1	0	0	383	15	113	213	13	29
*Charadrius vociferus*	1	0	0	0	467	25	161	221	18	42
*Chlamydotis macqueenii*	1	0	0	1	216	8	50	127	10	21
*Columba livia*	2	0	0	0	245	11	81	116	17	20
*Colius striatus*	1	0	0	0	237	9	94	110	7	17
*Corvus brachyrhynchos*	1	0	0	2	1,032	13	475	472	22	50
*Cuculus canorus*	2	0	0	0	191	11	73	95	2	10
*Egretta garzetta*	2	0	1	1	289	23	95	129	16	26
*Eurypyga helias*	2	0	0	0	288	6	104	147	12	19
*Falco peregrinus*	2	0	0	0	336	15	90	196	7	28
*Fulmarus glacialis*	2	0	0	0	245	10	65	121	11	38
*Gallus gallus*	0	0	0	0	573	21	146	228	54	124
*Gavia stellata*	4	0	0	0	207	12	37	125	12	21
*Geospiza fortis*	10	0	1	0	785	11	340	371	26	37
*Haliaeetus albicilla*	2	0	0	0	301	11	103	136	15	36
*Haliaeetus leucocephalus*	2	0	0	0	419	23	134	190	27	45
*Leptosomus discolor*	3	0	0	0	301	17	96	141	17	30
*Manacus vitellinus*	4	0	0	1	324	7	142	151	6	18
*Meleagris gallopavo*	0	0	0	0	303	7	73	140	21	62
*Melopsittacus undulatus*	38	0	0	0	485	27	117	284	26	31
*Merops nubicus*	2	0	0	0	418	11	149	191	31	36
*Mesitornis unicolor*	1	0	0	1	451	10	153	242	21	25
*Nestor notabilis*	5	0	1	0	223	8	65	116	20	14
*Nipponia nippon*	3	0	0	0	302	35	79	127	28	33
*Opisthocomus hoazin*	1	0	0	1	425	10	151	208	21	35
*Pelecanus crispus*	2	0	0	3	283	13	86	114	22	48
*Phalacrocorax carbo*	68	0	0	0	305	11	87	153	27	27
*Phaethon lepturus*	2	0	0	0	480	9	110	312	14	35
*Phoenicopterus ruber*	2	0	0	0	209	9	54	100	20	26
*Picoides pubescens*	2	1	0	0	502	9	164	278	20	31
*Podiceps cristatus*	3	0	0	0	366	7	123	187	23	26
*Pterocles gutturalis*	1	0	0	1	165	10	43	82	8	22
*Pygoscelis adeliae*	2	0	0	0	244	12	64	123	21	24
*Struthio camelus*	2	0	0	0	132	7	30	61	8	26
*Taeniopygia guttata*	13	0	0	1	725	19	302	322	34	48
*Tauraco erythrolophus*	1	0	0	0	397	5	168	198	5	21
*Tinamus major*	3	0	2	0	328	8	148	140	7	25
*Tyto alba*	5	0	0	0	477	10	169	244	16	38

^a^Retroviral elements that matched the *Retroviridae* but not to a specific genus.

We next consider each of the EVE families in turn.

### Endogenous viral elements related to the Retroviridae

As expected, ERVs were by far the most abundant EVE class in the avian genomes, covering the genera *Alpha-*, *Beta-*, *Gamma-*, and *Epsilonretrovirus*, with total ERV copy numbers ranging from 132 to 1,032. The greatest numbers of ERVs were recorded in the three oscine passerines (American crow, medium ground-finch and zebra finch, respectively) that exhibited EVE expansion (Table 1). ERVs related to beta- and gammaretroviruses were the most abundant in all avian genomes as noted in an important earlier study of three avian genomes [12]. In contrast, ERVs derived from epsilonretroviruses were extremely rare, with very few copies distributed (Additional file 2). We also found that ERVs related to alpharetroviruses were widely distributed in avian phylogeny, although with very low copy numbers [12]. In accord with the overall genetic pattern among the EVEs, the three oscine passerines exhibited greater numbers of ERVs than other taxa (two- to three-fold higher than the average; Table 1). This suggests that an ERV expansion occurred in the oscine passerines subsequent to their split from the suboscines. Phylogenetic analysis revealed that this pattern was due to frequent invasions of similar beta- and gammaretroviruses in these species (Table 1; Additional file 2).

Strikingly, the avian and non-avian (American alligator, green turtle and anole lizard) genomes seldom shared orthologous sequences (that is, only a few avian sequences can be aligned with those of non-avians and without matching flanking regions) and all their ERVs were distantly related (Additional file 2), indicative of a lack of vertical or horizontal transmission among these vertebrates. In addition, no non-retroviral elements were found in the non-avian genomes using our strict mining pipeline.

### Endogenous viral elements related to the Hepadnaviridae

Hepadnaviruses have very small genomes (approximately 3 kb) of partially double-stranded and partially single-stranded circular DNA. Their replication involves an RNA intermediate that is reverse transcribed in the cytoplasm and transported as cDNA back into the nucleus. Strikingly, we found endogenous hepadnaviral elements in all the avian genomes studied (Table S2 in Additional file 1), such that they were the most widely distributed non-retroviral EVEs recorded to date. In this context it is important to note that no mammalian endogenous hepadnaviruses have been described even though primates are major reservoirs for exogenous hepatitis B viruses [15].

Our phylogenetic analysis revealed a number of notable evolutionary patterns in the avian endogenous hepadnaviruses: (i) endogenous hepadnaviruses exhibited a far greater phylogenetic diversity, depicted as diverse clades, than their exogenous relatives (Additional file 3), suggesting they were older, although an acceleration in evolutionary rates among some hepadnaviral EVEs cannot be excluded; (ii) exogenous hepadnaviruses formed a tight monophyletic group compared with the endogenous elements (Additional file 3), indicative of a turnover of exogenous viruses during avian evolution; (iii) there was a marked difference in copy number (from 1 to 68) among avian species (Table S2 in Additional file 1), suggestive of the frequent gain and loss of viruses during avian evolution; and (iv) there was a phylogeny-wide incongruence between the virus tree (Additional file 3) and the host tree (*P* = 0.233 using ParaFit method), indicative of multiple independent genomic integration events as well as potential cross-species transmission events.

Despite the evidence for independent integration events, it was also clear that some hepadnavirus EVEs were inherited from a common ancestor of related avian groups, and perhaps over deep evolutionary time-scales. We documented these cases by looking for pairs of endogenous hepadnaviruses from different avian hosts that received strong (>70%) bootstrap support (Data S1 in Additional file 4) and which occupied orthologous locations. Specifically: (i) in the genomes of the white-tailed and bald eagles, the 5′ end of an hepadnavirus EVE was flanked by a same unknown gene while the 3′ end was flanked by the *dendritic cell immunoreceptor* (*DCIR*) gene (Additional file 3); (ii) an EVE shared by the emperor penguin and Adelie penguin (Additional file 3) was flanked by a same unknown gene at the 5′ end and the *Krueppel-like factor 8-like* gene at the 3′ end; and (iii) the ostrich and the great tinamou had the same flanking genes, albeit of unknown function, at both ends of an EVE.

**Figure 2:**
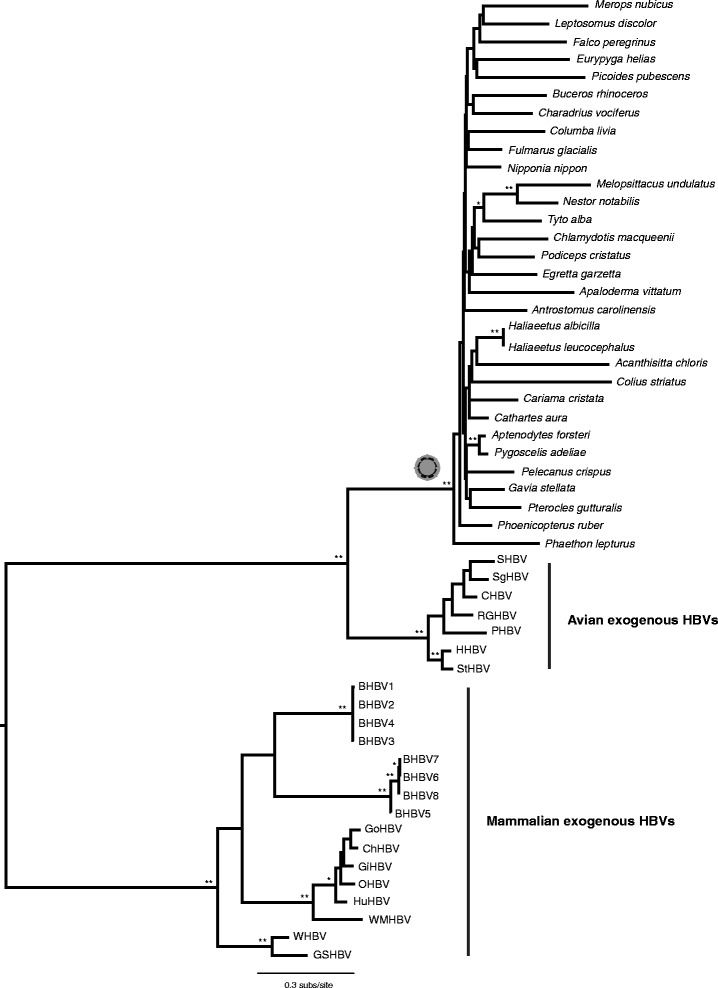
**Phylogenetic tree of exogenous and endogenous hepadnaviruses generated using complete polymerase (P) protein sequences.** Bootstrap values lower than 70% are not shown; single asterisks indicate values higher than 70%, while double asterisks indicate values higher than 90%. Branch lengths are drawn to a scale of amino acid substitutions per site (subs/site). The tree is midpoint rooted for purposes of clarity only. The exogenous hepadnaviruses are marked. A cartoon of a virus particle marks the phylogenetic location of an inherited hepadnavirus invasion. Avian host species names are used to denote avian endogenous hepadnaviruses and scaffold numbers are given in Table S2 in Additional file 1. All abbreviations are given in Table S9 in Additional file 1. HBV, hepatitis B virus.

We also recorded a rare case of vertical transmission of a hepadnavirus with a complete genome that has seemingly been inherited by 31 species (Table S2 in Additional file 1) prior to the diversification of the Neoaves 73 million years ago [14]. This virus has been previously denoted as eZHBV_C [11], and was flanked by the *furry homolog* (*FRY*) gene at both the 5′ and 3′ ends. Our hepadnavirus phylogeny (Figure 2) showed that this EVE group clustered tightly with extremely short internal branches, although with some topological patterns that were inconsistent with the host topology (Figure 1). A lack of phylogenetic resolution notwithstanding, this mismatch between the virus and host trees could be also in part be due to incomplete lineage sorting, in which there has been insufficient time for allele fixation during the short time period between bird speciation events. Indeed, Neoaves are characterized by a rapid species radiation [16].

Strikingly, we observed that two Galliformes species, chicken and turkey, have seemingly purged their hepadnaviral EVEs. Specifically, genomic mining revealed no hepadnaviral elements in these galliformes, even though their closest relatives (Anseriformes) contained such elements. In support of this genome purging, we noted that one hepadnaviral element present in the mallard genome has been severely degraded through frequent mutation in the chicken genome (Additional file 5). In addition, remnants of orthologous 5′ and 3′ regions could also be found in the turkey genome, although the rest of the element was deleted (Additional file 5).

### Endogenous viral elements related to the Bornaviridae

Bornaviruses (family Bornaviridae) are linear, unsegmented negative-sense ssRNA viruses with genomes of approximately 9 kb. They are unusual among animal RNA viruses in their ability to replicate within the host cell nucleus, which in turn assists endogenization. Indeed, orthomyxoviruses and some insect rhabdoviruses also replicate in the nucleus and both have been found to occur as endogenous forms in insect genomes [2]. Endogenous elements of bornaviruses, denoted endogenous bornavirus-like N (EBLN) [2,17,18] and endogenous bornavirus-like L (EBLL) [2,18], have been discovered in mammalian genomes, including humans, and those present in primates have been dated to have arisen more than 40 million years ago [17,18]. Although exogenous bornaviruses circulate in both mammals and birds and cause fatal diseases [19,20], endogenous bornaviruses have not yet been documented in avian species.

We report, for the first time, that both EBLN and EBLL are present in several avian genomes (Additional file 6), although in only three species and with very low copy numbers (1 to 4; Table S3 in Additional file 1): the Anna’s hummingbird, the closely related chimney swift, and the more distantly related woodpecker. Both EBLN and EBLL in the genome of Anna’s hummingbird were divergent compared with other avian or mammalian viruses. The chimney swift possessed a copy of EBLN, which was robustly grouped in the phylogenetic tree with the EVE present in Anna’s hummingbird (Figure S4A in Additional file 6). However, as these viral copies did not share the same flanking regions in the host genomes, as well as the inconsistent phylogenetic positions of the EBLN (Figure S4A in Additional file 6) and EBLL (Figure S4C in Additional file 6) of Anna’s hummingbird, they likely represent independent integration events. In addition, due to the close relationships among some of the viruses in different species, it is possible that cross-species transmission has occurred because of shared geographical distributions (for example, woodpeckers are widely distributed across the United States, with geographic distributions that overlap with those of Anna’s hummingbirds). The EBLN in the downy woodpecker was likely to have entered the host genome recently as in the phylogenetic tree it was embedded within the genetic diversity of exogenous viruses; the same pattern was observed in the case of the two viral copies in the genome of Anna’s hummingbird (Figure S4B in Additional file 6). Similar to previous studies in mammals [21], we found that more species have incorporated EBLN than EBLL. However, compared with their wide distribution in mammalian genomes, it was striking that only three avian species carried endogenous bornavirus-like elements.

### Endogenous viral elements related to the Circoviridae

Circoviruses (family Circoviridae) possess approximately 2 kb ssDNA, nonenveloped and unsegmented circular genomes, and replicate in the nucleus via a rolling circle mechanism. They are known to infect birds and pigs and can cause a wide range of severe symptoms such as Psittacine circovirus disease. There are two main open reading frames, usually arranged in an ambisense orientation, that encode the replication (Rep) and capsid (Cap) proteins. Endogenous circoviruses (eCiVs) are rare, and to date have only been reported in four mammalian genomes, with circoviral endogenization in carnivores dating to at least 42 million years [22].

We found circoviruses to be incorporated into only four avian genomes - medium ground finch, kea, egret, and tinamou - and at copy numbers of only 1 to 2 (Additional file 7; Table S5 in Additional file 1). There were at least two divergent groups of eCiVs in the viral phylogenetic tree, one in the medium ground-finch and great tinamou (Figure S5A-C in Additional file 7), which was closely related to exogenous avian circoviruses, and another in the little egret and kea (Figure S5C,D in Additional file 7), which was only distantly related to avian exogenous counterparts. The large phylogenetic distances among these endogenous viruses are suggestive of independent episodes of viral incorporation. In addition, two pieces of evidence strongly suggested that eCiVs in the medium ground-finch and great tinamou (Figure S5A-C in Additional file 7) have only recently entered host genomes: (i) they had close relationships with their exogenous counterparts, and (ii) they maintained complete (or nearly complete) open reading frames (Table S5 in Additional file 1).

### Endogenous viral elements related to the Parvoviridae

The family Parvoviridae comprises two subfamilies - Parvovirinae and Densovirinae - that infect diverse vertebrates and invertebrates, respectively. Parvoviruses typically possess linear, non-segmented ssDNA genomes with an average size of approximately 5 kb, and replicate in the nucleus. Parvoviruses have been documented in a wide range of hosts, including humans, and can cause a range of diseases [23]. Recent studies revealed that endogenous parvoviruses (ePaVs) have been broadly distributed in mammalian genomes, with integration events dating back at least 40 million years [22].

We found multiple entries of ePaVs with very low copy numbers (1 to 3; Table S5 in Additional file 1) in 10 avian genomes (Additional file 8), and they were not as widely distributed as those parvoviruses present in mammalian genomes [22]. All avian ePaVs were phylogenetically close to exogenous avian parvoviruses with the exception of a single one from the brown mesite, which was distantly related to all known animal parvoviruses (Additional file 8). We also found several cases of apparently vertical transmission. For example, one common ePaV in the American crow and rifleman was flanked by the same unknown host gene; the viral copy in the golden-collared manakin and zebra finch was flanked by the *tyrosine-protein phosphatase non-receptor type 13* (*PTPN13*) gene at the 5′ end and the same unknown gene at the 3′ end; and one viral element in the little egret and Dalmatian pelican was flanked by a same chicken repeat 1 (CR1) at the 5′ end and *collagen alpha 1 gene* (*COL14A1*) at the 3′ end (Data S2 in Additional file 4). These findings suggest both independent integration and vertical transmission (that is, common avian ancestry) for ePAVs that have seemingly existed in birds for at least 30 million years (that is, the separation time of *Corvus* and *Acanthisitta* [14]).

### Low frequency of retroviral endogenous viral elements in bird genomes

**Figure 3:**
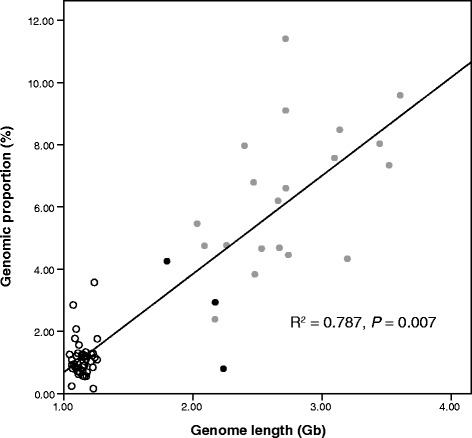
**Relationship between the proportion (percentage) of retrovirus-like elements in each vertebrate genome and host genome size.** The y-axis shows the proportion of LTR-retrotransposons in a variety of vertebrate genomes, while the x-axis indicates genome length in gigabases (Gb). The solid line marks the phylogenetic linear regression for host genome size and the EVE proportion of the genome. Hosts are recognized as follows: hollow circles, birds; black, American alligator, green turtle and anole lizard; grey, mammals.

**Figure 4:**
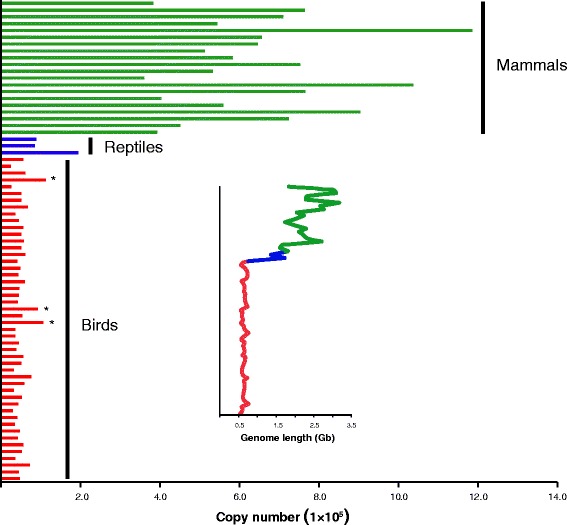
**Copy numbers of retroviral endogenous viral elements among birds, American alligator, green turtle, anole lizard, and mammals.** Different host groups are colored as red (birds), blue (American alligator, green turtle and anole lizard) and green (mammals). A trend of increasing genome size is also noted. Species are listed from bottom to top in accordance with the bird species order given in Table S6 in Additional file 1, and the order among the American alligator, green turtle, anole lizard, and mammals given in Table S7 in Additional file 1. Asterisks indicate three oscine passerines showing an EVE expansion.

To determine the overall pattern and frequency of infiltration of EVEs in the genomes of birds, American alligator, green turtle, anole lizard, and mammals, we documented the phylogeny-wide abundance of long terminal repeat (LTR)-retrotransposons of retrovirus-like origin [24]. As retroviral elements comprise >99.99% of avian EVEs they obviously represent the most meaningful data set to explore patterns of EVE evolution. This analysis revealed that retroviral EVEs are far less common in birds than in mammals: the average retroviral proportion of the genome was 1.12% (range 0.16% to 3.57%) in birds, 2.39% to 11.41% in mammals, and 0.80% to 4.26% in the genomes of American alligator, green turtle and anole lizard (Tables S6 and S7 in Additional file 1). Strikingly, there was also a simple linear relationship between host genome size and EVE proportion (R^2^ = 0.787, *P* = 0.007; Figure 3). Of equal note was the observation that EVE copy numbers in bird genomes were an order of magnitude less frequent than in mammals (Figure 4; Tables S6 and S7 in Additional file 1), and that the relationship between viral copy number and host genome size exhibited a linear trend (R^2^ = 0.780, *P* < 0.001). Importantly, in all cases (that is, genome size versus proportion and genome size versus copy number) we employed phylogenetic regression analyses to account for the inherent phylogenetic non-independence of the data points.

## Discussion and conclusions

Although a diverse array of viruses can possess endogenous forms [2], our analysis revealed that they are uncommon in avian genomes, especially those derived from RNA viruses. Indeed, among RNA viruses, we found only bornavirus endogenized forms occurred in avian genomes, and these had a sporadic distribution and very low frequencies. Although bird genomes are approximately one-third to one-half the size of those of mammals [25,26], the proportion of their genomes that comprises EVEs and their EVE copy numbers are 6 and 13 times less frequent, respectively. It is generally acknowledged that the genome size reduction associated with flying avian species evolved in the asurischian dinosaur lineage [25]. Our broad-scale genomic screening also suggested that a low frequency of EVEs was an ancestral trait in avian lineage, especially in the case of ERVs, such that there has been an expansion of EVE numbers in mammals concomitant with an increase in their genome sizes. Also of note was that although some genomic integration events in birds were vertical, allowing us to estimate an approximate time-scale for their invasion over many millions of years, by far the most common evolutionary pattern in the avian data was the independent integration of EVEs into different species/genera.

There are a variety of reasons why EVE numbers could be so relatively low in avian genomes. First, it is theoretically possible that birds have been exposed to fewer viral infections than mammals. However, this seems unlikely as, although they are likely to have been examined less intensively than mammals [27], exogenous viruses of various kinds are found in avian species (for example, Coronaviridae*,* Flaviviridae*,* Hepadnaviridae*,* Orthomyxoviridae*,* Paramyxoviridae*,* Poxviridae*,* Retroviridae). In addition, the most common phylogenetic pattern we noted was that of independent integration, suggesting the presence of diverse exogenous infections. However, it is notable that mammals apparently harbor a more diverse set of exogenous retroviruses than birds, as well as a greater abundance of ERVs, which is indicative of a deep-seated evolutionary interaction between host and virus [28]. For example, the only gammaretrovirus known in birds is reticuloendotheliosis virus (REV), and a recent study suggested that avian REVs have a mammalian origin [29]. This is consistent with our observation that there are no endogenized forms of REVs among this diverse set of avian genomes.

It is also possible that birds are in some way refractory to EVE integration following viral infection. ERVs can replicate both as retrotransposons and as viruses via infection as well as re-infection. Although bird cells are known to be susceptible to certain retroviruses [1], the replication of avian ERVs within the host genome could be suppressed, at least in part, by host-encoded factors. However, a general conclusion of our study is that non-retroviral EVEs are seemingly rare in all vertebrates, such that their integration appears to be generically difficult, and the relative abundance of endogenous retroviruses in birds (albeit low compared with mammals) indicates that they are able to enter bird genomes, with some being actively transcribed and translated [12]. Our observation of a lineage-specific ERV expansion in three passerines also argues against a general refractory mechanism.

A third explanation is that birds are particularly efficient at purging EVEs especially for viruses with retroviral origin from their genomes, a process that we effectively ‘caught in the act’ in the case of the galliform hepadnaviruses. Indeed, our observation of a very low frequency of LTR-retrotransposons in avian genomes may reflect the action of a highly efficient removal mechanism, such as a form of homologous recombination. Hence, it is likely that active genome purging must be responsible for some of the relative absence of EVEs in birds, in turn retaining a selectively advantageous genomic compactness [30]. Clearly, additional work is needed to determine which of these, or other mechanisms, explain the low EVE numbers in avian genomes.

## Materials and methods

### Genome sequencing and assembly

To systematically study endogenous viral elements in birds, we mined the genomes of 48 avian species (Table S1 in Additional file 1). Of these, three genomes - chicken [31], zebra finch [32] and turkey [33] - were downloaded from Ensembl [34]. The remaining genomes were acquired as part of our avian comparative genomics and phylogenomics consortium [13,14]. All genomes can be obtained from our two databases: CoGe [35] and Phylogenomics Analysis of Birds [36]. American alligator, green turtle, anole lizard, and 20 mammal genomes (Table S7 in Additional file 1) were downloaded from Ensembl [34] and used for genomic mining and the subsequent comparative analysis.

### Genomic mining

Chromosome and whole genome shotgun assembles [13,34-36] of all species (Table S1 in Additional file 1) were downloaded and screened *in silico* using tBLASTn and a library of representative viral protein sequences derived from Groups I to VII (dsDNA, ssDNA, dsRNA, +ssRNA, -ssRNA, ssRNA-RT, and dsDNA-RT) of the 2009 ICTV (International Committee on Taxonomy of Viruses) [37] species list (Additional file 9). All viral protein sequences were used for genomic mining. Host genome sequences that generated high-identity (E-values <1e^-5^) matches to viral peptides were extracted. Matches similar to host proteins were filtered and discarded. The sequences were considered virus-related if they were unambiguously matched viral proteins in the NCBI nr (non-redundant) database [38] and the PFAM database [39]. The putative viral gene structures were inferred using GeneWise [40]. The *in silico* mining of LTR-retrotransposons was performed using RepeatMasker [41].

### Phylogenetic inference

To establish the phylogenetic positions of the avian EVEs, particularly in comparison with their exogenous counterparts, we collected all relevant reference viral sequences (Table S9 in Additional file 1) from GenBank [42]. Protein sequences (both EVEs and exogenous viruses) were aligned using MUSCLE [43] and checked manually. Phylogenetic trees were inferred using the maximum likelihood method available in PhyML 3.0 [44], incorporating the best-fit amino acid substitution models determined by ProtTest 3 [45]. The robustness of each node in the tree was determined using 1,000 bootstrap replicates. We subdivided our viral data into 16 categories for phylogenetic analysis (see Results): 1) endogenous hepadnaviruses, using both complete and partial P (polymerase) protein sequences from positions 429 to 641 (reference sequence DHBV, NC_001344); 2) EBLN, using partial N (nucleoprotein) protein sequences, from positions 43 to 224 (BDV, NC_001607); 3) EBLL, using partial L (RNA-dependent RNA polymerase) protein sequences, from positions 121 to 656; 4) eCiV Cap, using complete Cap (capsid) protein sequences (GooCiV, NC_003054); 5) eCiV Rep data set 1, using complete Rep (replicase) protein sequences; 6) eCiV Rep data set 2, using partial Rep protein sequences, from positions 160 to 228; 7) eCiV Rep data set 3, using partial Rep protein sequences, from positions 8 to 141; 8) ePaV Cap data set 1, using partial Cap protein sequences, from positions 554 to 650 (DucPaV, NC_006147); 9) ePaV Cap data set 2, using partial Cap protein sequences, from positions 406 to 639; 10) ePaV Cap data set 3, using partial Cap protein sequences, from positions 554 to 695; 11) ePaV Cap data set 4, using partial Cap protein sequences, from positions 662 to 725; 12) ePaV Rep data set 1, using partial Rep protein sequences, from positions 104 to 492; 13) ePaV Rep data set 2, using partial Rep protein sequences, from positions 245 to 383; 14) ePaV Rep data set 3, using partial Rep protein sequences, from positions 300 to 426; 15) ePaV Rep data set 4, using partial Rep protein sequences, from positions 1 to 40; and 16) ERVs, using the retroviral motif ‘DTGA-YMDD’ of Pro-Pol sequences. The best-fit models of amino acid substitution in each case were: 1) JTT + Γ; 2) JTT + Γ; 3) LG + Γ; 4) RtREV + Γ; 5) LG + I + Γ; 6) LG + Γ; 7) LG + I + Γ; 8) LG + Γ; 9) WAG + I + Γ; 10) LG + Γ; 11) LG + Γ; 12) LG + Γ; 13) LG + I + Γ; 14) LG + I + Γ; 15) LG + Γ; and 16) JTT + Γ.

### Statistical analysis

To account for the phylogenetic relationships of avian taxa when investigating patterns of EVE evolution we employed phylogenetic linear regression as implemented in R [46]. Specifically, using Mesquite [47] we manually created a tree that matched the host vertebrate phylogeny [14,48]. For the subsequent phylogenetic regression analysis we utilized the ‘phylolm’ package in R [49], which provides a function for fitting phylogenetic linear regression and phylogenetic logistic regression.

The extent of co-divergence between viruses and hosts was tested by using ParaFit [50], as implemented in the COPYCAT package [51]. The significance of the test was derived from 99,999 randomizations of the association matrix.

### Data availability

Data can be accessed by GigaDB [52]. Alternatively, the IDs of NCBI BioProject/Sequence Read Archive (SRA)/study are as follows: *Chaetura pelagica*, PRJNA210808/SRA092327/SRP026688; *Calypte anna*, PRJNA212866/SRA096094/SRP028275; *Charadrius vociferus*, PRJNA212867/SRA096158/SRP028286; *Corvus brachyrhynchos*, PRJNA212869/SRA096200/SRP028317; *Cuculus canorus*, PRJNA212870/SRA096365/SRP028349; *Manacus vitellinus*, PRJNA212872/SRA096507/SRP028393; *Ophisthocomus hoazin*, PRJNA212873/SRA096539/SRP028409; *Picoides pubescens*, PRJNA212874/SRA097131/SRP028625; *Struthio camelus*, PRJNA212875/SRA097407/SRP028745; *Tinamus guttatus*, PRJNA212876/SRA097796/SRP028753; *Acanthisitta chloris*, PRJNA212877/SRA097960/SRP028832; *Apaloderma vittatum*, PRJNA212878/SRA097967/SRP028834; *Balearica regulorum*, PRJNA212879/SRA097970/SRP028839; *Buceros rhinoceros*, PRJNA212887/SRA097991/SRP028845; *Antrostomus carolinensis*, PRJNA212888/SRA098079/SRP028883; *Cariama cristata*, PRJNA212889/SRA098089/SRP028884; *Cathartes aura*, PRJNA212890/SRA098145/SRP028913; *Chlamydotis macqueenii*, PRJNA212891/SRA098203/SRP028950; *Colius striatus*, PRJNA212892/SRA098342/SRP028965; *Eurypyga helias*, PRJNA212893/SRA098749/SRP029147; *Fulmarus glacialis*, PRJNA212894/SRA098806/SRP029180; *Gavia stellata*, PRJNA212895/SRA098829/SRP029187; *Haliaeetus albicilla*, PRJNA212896/SRA098868/SRP029203; *Haliaeetus leucocephalus*, PRJNA237821/SRX475899, SRX475900, SRX475901, SRX475902/SRP038924; *Leptosomus discolor*, PRJNA212897/SRA098894/SRP029206; *Merops nubicus*, PRJNA212898/SRA099305/SRP029278; *Mesitornis unicolor*, PRJNA212899/SRA099409/SRP029309; *Nestor notabilis*, PRJNA212900/SRA099410/SRP029311; *Pelecanus crispus*, PRJNA212901/SRA099411/SRP029331; *Phaethon lepturus*, PRJNA212902/SRA099412/SRP029342; *Phalacrocorax carbo*, PRJNA212903/SRA099413/SRP029344; *Phoenicopterus ruber*, PRJNA212904/SRA099414/SRP029345; *Podiceps cristatus*, PRJNA212905/SRA099415/SRP029346; *Pterocles gutturalis*, PRJNA212906/SRA099416/SRP029347; *Tauraco erythrolophus*, PRJNA212908/SRA099418/SRP029348; *Tyto alba*, PRJNA212909/SRA099419/SRP029349; *Nipponia nippon*, PRJNA232572/SRA122361/SRP035852; *Egretta garzetta*, PRJNA232959/SRA123137/SRP035853. The following IDs are released before this study: *Aptenodytes forsteri*, PRJNA235982/SRA129317/SRP035855; *Pygoscelis adeliae*, PRJNA235983/SRA129318/SRP035856; *Gallus gallus*, PRJNA13342/SRA030184/SRP005856; *Taeniopygia guttata*, PRJNA17289/SRA010067/SRP001389; *Meleagris gallopavo*, PRJNA42129/Unknown/Unknown; *Melopsittacus undulatus*/PRJEB1588/ERA200248/ERP002324; *Anas platyrhynchos*, PRJNA46621/SRA010308/SRP001571; *Columba livia*, PRJNA167554/SRA054954/SRP013894; *Falco peregrinus*, PRJNA159791/SRA055082/SRP013939; *Geospiza fortis*, PRJNA156703/SRA051234/SRP011940.

## Additional files

Additional file 1: Table S1 Avian genomes used for genomic mining. **Table S2.** Endogenous hepadnaviruses in avian genomes. **Table S3.** Endogenous bornaviruses in avian genomes. **Table S4.** Endogenous circoviruses in avian genomes. **Table S5.** Endogenous parvoviruses in avian genomes. **Table S6.** LTR-retrotransposon composition of avian genomes. **Table S7.** LTR-retrotransposon composition of American alligator, green turtle, anole lizard and mammalian genomes. **Table S9.** Reference sequences used for phylogenetic analyses. Additional file 2: Figure S1 Phylogenetic tree of endogenous retroviruses (ERVs). The tree was inferred using the conserved motif ‘DTGA-YMDD’ within the Pro-Pol region of retroviruses (approximately 320 amino acids in length, although this differs among retrovirus genera). Bootstrap values lower than 70% are not shown; single asterisks indicate values higher than 70%, while double asterisks indicate values higher than 90%. Branch lengths are drawn to a scale of amino acid substitutions per site (subs/site). The tree is midpoint rooted for purposes of clarity only. The host name indicates the species from which the ERV was obtained. Exogenous retroviruses are highlighted using family names. ERVs of alligator, turtle and lizard origin are also highlighted. Additional file 3: Figure S2 Phylogenetic tree of exogenous and endogenous avian hepadnaviruses. Bootstrap values lower than 70% are not shown; single asterisks indicate values higher than 70%, while double asterisks indicate values higher than 90%. Branch lengths are drawn to a scale of amino acid substitutions per site (subs/site). The tree is midpoint rooted for purposes of clarity only. The exogenous hepadnaviruses are highlighted. Avian host species names are used to denote avian endogenous hepadnaviruses, and different EVEs from the same host are numbered. All abbreviations are provided in Table S9 in Additional file 1. Additional file 4
**Data S1.** Alignments of the orthologous hepadnaviral scaffolds. **Data S2.** Alignments of the orthologous parvoviral scaffolds. Additional file 5: Figure S3 Alignment of a hepadnaviral element in the genome of mallard duck with orthologous (and partial) sequences found in the genomes of chicken and turkey. Note that we found a 94% match to the 5′ conserved region (marked as C) in turkey, and a 39% match to the orthologous chicken sequence; 45% of the central 12,042-bp virus-like sequence matched the 5′ variable region (marked as V). The relatively conserved nucleotides in chicken showing virus-like characteristics are boxed. Asterisks indicate the conserved nucleotides in the alignment, dashes denote deletions. Additional file 6: Figure S4 Phylogenetic trees of endogenous and exogenous bornaviruses. The phylogenies contain **(A)** endogenous bornavirus-like N (nucleoprotein) (EBLN) and **(B)** avian endogenous bornavirus-like L (RNA-dependent RNA polymerase) (EBLL) sequences. Bootstrap values lower than 70% are not shown; single asterisks indicate values higher than 70%, while double asterisks indicate values higher than 90%. Branch lengths are drawn to a scale of amino acid substitutions per site (subs/site). The trees are midpoint rooted for purposes of clarity only. Avian host species names for those that harbor EVEs are given in parentheses and different EVEs from the same host are numbered. All abbreviations are provided in Table S9 in Additional file 1. Additional file 7: Figure S5 Phylogenetic trees of endogenous circoviruses. **(A-D)** The phylogenies contain avian endogenous circoviruses (eCiVs) Cap (A) and Rep (B-D). Bootstrap values lower than 70% are not shown; single asterisks indicate values higher than 70%, while double asterisks indicate values higher than 90%. Branch lengths are drawn to a scale of amino acid substitutions per site (subs/site). The trees are midpoint rooted for purposes of clarity only. Avian host species names for those that harbor EVEs are given in parentheses. All abbreviations are provided in Table S9 in Additional file 1. Additional file 8: Figure S6 Phylogenetic trees of endogenous and exogenous parvoviruses. **(A-H)** The phylogenies contain avian endogenous parvoviruses (ePaVs) Cap (A-D) and Rep (E-H). Bootstrap values lower than 70% are not shown; single asterisks indicate values higher than 70%, while double asterisks indicate values higher than 90%. Branch lengths are drawn to a scale of amino acid substitutions per site (subs/site). The trees are midpoint rooted for purposes of clarity only. Avian host species names for those that harbor EVEs are given in parentheses and different EVEs from the same host are numbered. All abbreviations are provided in Table S9 in Additional file 1. Additional file 9: Table S8 Reference viral sequences used for genomic searching.

## Abbreviations

ds: double-stranded
EBLL: endogenous bornavirus-like L
EBLN: endogenous bornavirus-like N
eCiV: endogenous circovirus
ePaV: endogenous parvovirus
ERV: endogenous retrovirus
EVE: endogenous viral element
REV: reticuloendotheliosis virus
RT: reverse transcriptase
SRA: Sequence Read Archive
ss: single-stranded
