# Supplementary material for: Low frequency of paleoviral infiltration across the avian phylogeny
Source: Genome Biol. 2014 Dec 11;15(12):539. doi: 10.1186/s13059-014-0539-3 (PMC4272516; doi:10.1186/s13059-014-0539-3)
Supplement: Additional file 1: Table S1 — Avian genomes used for genomic mining. Table S2. Endogenous hepadnaviruses in avian genomes. Table S3. Endogenous bornaviruses in avian genomes. Table S4. Endogenous circoviruses in avian genomes. Table S5. Endogenous parvoviruses in avian genomes. Table S6. LTR-retrotransposon composition of avian genomes. Table S7. LTR-retrotransposon composition of American alligator, green turtle, anole lizard and mammalian genomes. Table S9. Reference sequences used for phylogenetic analyses. [file 13059_2014_539_MOESM1_ESM.docx]

**Supplementary Materials for**

Low Frequency of Paleoviral Infiltration Across the Avian Phylogeny

Jie Cui*, Wei Zhao, Zhiyong Huang, Erich D. Jarvis, M. Thomas P. Gilbert, Peter J. Walker, Edward C. Holmes, Guojie Zhang*

*Correspondence to: zhanggj@genomics.org.cn (G.Z.); jiecui@yahoo.com (J.C.).

**Figure legends**

**Fig. S1.** Phylogenetic tree of endogenous retroviruses (ERVs). The tree was inferred using the conserved motif “DTGA-YMDD” within the Pro-Pol region of retroviruses (~320 amino acids in length, although this differs among retrovirus genera). Bootstrap values lower than 70% are not shown; one star (*) represents values higher than 70%, while two stars (**) represents values higher than 90%. Branch lengths are drawn to a scale of amino acid substitutions per site (subs/site). The tree is midpoint rooted for purposes of clarity only. The host name indicates the species from which the ERV was obtained. Exogenous retroviruses are highlighted using family names. ERVs of alligator, turtle and lizard origin are also highlighted.

**Fig. S2.** Phylogenetic tree of exogenous and endogenous avian hepadnaviruses. Bootstrap values lower than 70% are not shown; one star (*) represents values higher than 70%, while two stars (**) represents values higher than 90%. Branch lengths are drawn to a scale of amino acid substitutions per site (subs/site). The tree is midpoint rooted for purposes of clarity only. The exogenous hepadnaviruses are highlighted. Avian host species names are used to denote avian endogenous hepadnaviruses, and different EVEs from the same host are numbered. All abbreviations are provided in Table S9.

**Fig. S3.** Alignment of a hepadnaviral element in the genome of mallard duck with orthologous (and partial) sequences found in the genomes of chicken and turkey. Note that we found a 94% match to the 5’ conserved region (marked as C) in turkey, and a 39% match to the orthologous chicken sequence; 45% of the central 12,042-bp virus-like sequence matched the 5’ variable region (marked as V). The relatively conserved nucleotides in chicken showing virus-like characteristics are boxed. Asterisks represent the conserved nucleotides in the alignment, dashes denote deletions.

**Fig. S4.** Phylogenetic trees of endogenous and exogenous bornaviruses. The phylogenies contain (A) endogenous bornavirus-like N (nucleoprotein) (EBLN) and (B) avian endogenous bornavirus-like L (RNA-dependent RNA polymerase) (EBLL) sequences. Bootstrap values lower than 70% are not shown; one star (*) represents values higher than 70%, while two stars (**) represents values higher than 90%. Branch lengths are drawn to a scale of amino acid substitutions per site (subs/site). The trees are midpoint rooted for purposes of clarity only. Avian host species names for those that harbor EVEs are given in parentheses and different EVEs from the same host are numbered. All abbreviations are provided in Table S9.

**Fig. S5.** Phylogenetic trees of endogenous circoviruses. The phylogenies contain avian endogenous circoviruses (eCiVs) Cap (A) and Rep (B, C and D). Bootstrap values lower than 70% are not shown; one star (*) represents values higher than 70%, while two stars (**) represents values higher than 90%. Branch lengths are drawn to a scale of amino acid substitutions per site (subs/site). The trees are midpoint rooted for purposes of clarity only. Avian host species names for those that harbor EVEs are given in parentheses. All abbreviations are provided in Table S9.

**Fig. S6.** Phylogenetic trees of endogenous and exogenous parvoviruses. The phylogenies contain avian endogenous parvoviruses (ePaVs) Cap (A, B, C, and D) and Rep (E, F, G, and H). Bootstrap values lower than 70% are not shown; one star (*) represents values higher than 70%, while two stars (**) represents values higher than 90%. Branch lengths are drawn to a scale of amino acid substitutions per site (subs/site). The trees are midpoint rooted for purposes of clarity only. Avian host species names for those that harbor EVEs are given in parentheses and different EVEs from the same host are numbered. All abbreviations are provided in Table S9.

**Table S1.** Avian genomes used for genomic mining.

| **Species** | **Common name** | **Order** | **Family** | **Coverage** |
| --- | --- | --- | --- | --- |
| **Birds** |  |  |  |  |
| *Acanthisitta chloris* | Rifleman | Passeriformes | Acanthisittidae | 29X |
| *Anas platyrhynchos* | Mallard | Anseriformes | Anatidae | 50X |
| *Antrostomus carolinensis* | Chuck-will’s-widow | Caprimulgiformes | Caprimulgidae | 30X |
| *Apaloderma vittatum* | Bar-tailed trogon | Trogoniformes | Trogonidae | 28X |
| *Aptenodytes forsteri* | Emperor penguin | Sphenisciformes | Spheniscidae | 60X |
| *Balearica regulorum* | Grey crowned crane | Gruiformes | Gruidae | 33X |
| *Buceros rhinoceros* | Rhinoceros hornbill | Coraciiformes | Bucerotidae | 35X |
| *Calypte anna* | Anna's hummingbird | Trochiliformes | Trochilidae | 110X |
| *Cariama cristata* | Red-legged seriema | Cariamiformes | Cariamidae | 24X |
| *Cathartes aura* | Turkey vulture | OD* | Cathartidae | 25X |
| *Chaetura pelagica* | Chimney swift | Apodiformes | Apodidae | 106X |
| *Charadrius vociferus* | Killdeer | Charadriiformes | Charadriidae | 100X |
| *Chlamydotis macqueenii* | MacQueen’s bustard | Gruiformes | Otidae | 27X |
| *Columba livia* | Pigeon | Columbiformes | Columbidae | 64X |
| *Colius striatus* | Speckled mousebird | Coliiformes | Coliidae | 27X |
| *Corvus brachyrhynchos* | American crow | Passeriformes | Corvidae | 90X |
| *Cuculus canorus* | Common cuckoo | Cuculiformes | Cuculidae | 100X |
| *Egretta garzetta* | Little egret | Ciconiiformes | Ardeidae | 74X |
| *Eurypyga helias* | Sunbittern | Eurypygiformes | Eurypygidae | 33X |
| *Falco peregrinus* | Peregrine falcon | Falconiformes** | Falconidae | 105X |
| *Fulmarus glacialis* | Northern fulmar | Procellariiformes | Procellariidae | 33X |
| *Gallus gallus* | Chicken | Galliformes | Phasianidae | 7.1X |
| *Gavia stellata* | Red-throated loon | Gaviiformes | Gaviidae | 33X |
| *Geospiza fortis* | Medium ground-finch | Passeriformes | Thraupidae | 115X |
| *Haliaeetus albicilla* | White-tailed eagle | Falconiformes** | Accipitridae | 26X |
| *Haliaeetus leucocephalus* | Bald eagle | Falconiformes** | Accipitridae | 30X |
| *Leptosomus discolor* | Cuckoo roller | Leptosomatiformes | Leptosomatidae | 32X |
| *Manacus vitellinus* | Golden-collared manakin | Passeriformes | Pipridae | 110X |
| *Meleagris gallopavo* | Turkey | Galliformes | Phasianidae | 30X |
| *Melopsittacus undulatus* | Budgerigar | Psittaciformes | Psittaculidae | >30X |
| *Merops nubicus* | Carmine bee-eater | Coraciiformes | Meropidae | 37X |
| *Mesitornis unicolor* | Brown mesite | Mesitornithiformes | Mesitornithidae | 29X |
| *Nestor notabilis* | Kea | Psittaciformes | Nestoridae | 32X |
| *Nipponia nippon* | Crested ibis | Ciconiiformes | Threskiornithidae | 105X |
| *Opisthocomus hoazin* | Hoatzin | Opisthocomiformes | Opisthocomidae | 100X |
| *Pelecanus crispus* | Dalmatian pelican | Pelecaniformes | Pelecanidae | 34X |
| *Phalacrocorax carbo* | Cormorant | Pelecaniformes | Phalacrocoracidae | 24X |
| *Phaethon lepturus* | White-tailed tropicbird | Phaethontiformes | Phaethontidae | 39X |
| *Phoenicopterus ruber* | American flamingo | Phoenicopteriformes | Phoenicopteridae | 33X |
| *Picoides pubescens* | Downy woodpecker | Piciformes | Picidae | 105X |
| *Podiceps cristatus* | Great crested grebe | Podicipediformes | Podicipedidae | 30X |
| *Pterocles gutturalis* | Yellow-throated sandgrouse | Columbiformes | Pteroclididae | 25X |
| *Pygoscelis adeliae* | Adelie penguin | Sphenisciformes | Spheniscidae | 60X |
| *Struthio camelus* | Ostrich | Struthioniformes | Struthionidae | 85X |
| *Taeniopygia guttata* | Zebra finch | Passeriformes | Estrildidae | 6X |
| *Tauraco erythrolophus* | Red-crested turaco | Cuculiformes | Musophagidae | 30X |
| *Tinamus major* | Great tinamou | Tinamiformes | Tinamidae | 100X |
| *Tyto alba* | Barn owl | Strigiformes | Tytonidae | 27X |

* Order disputed

** Synonym Accipitriformes

**Table S2.** Endogenous hepadnaviruses in avian genomes.

| **Name** | **Copy no.** | **Scaffold/locus** |
| --- | --- | --- |
| *Acanthisitta chloris* | 2 | scaffold27280**, scaffold7507 |
| *Anas platyrhynchos* | 4 | scaffold490, scaffold3733, C18139412, C19237742 |
| *Antrostomus carolinensis* | 2 | scaffold6095, scaffold16207** |
| *Apaloderma vittatum* | 2 | scaffold18931, scaffold16435** |
| *Aptenodytes forsteri* | 2 | scaffold155**, scaffold191 |
| *Balearica regulorum* | 3 | scaffold25654, scaffold18147, scaffold1498 |
| *Buceros rhinoceros* | 3 | scaffold27050, scaffold36794**, scaffold1204** |
| *Calypte anna* | 3 | scaffold20, scaffold422, scaffold33 |
| *Cariama cristata* | 3 | scaffold26485, scaffold5097**, scaffold329** |
| *Cathartes aura* | 2 | scaffold29482, scaffold19789** |
| *Chaetura pelagica* | 2 | scaffold53, scaffold83 |
| *Charadrius vociferus* | 1 | scaffold16** |
| *Chlamydotis macqueenii* | 1 | scaffold36512** |
| *Columba livia* | 2 | scaffold27, scaffold59** |
| *Colius striatus* | 1 | scaffold2903** |
| *Corvus brachyrhynchos* | 1 | scaffold103 |
| *Cuculus canorus* | 2 | scaffold803, scaffold15 |
| *Egretta garzetta* | 2 | scaffold40**, scaffold226 |
| *Eurypyga helias* | 2 | scaffold15072**, scaffold28437 |
| *Falco peregrinus* | 2 | scaffold17**, scaffold443 |
| *Fulmarus glacialis* | 2 | scaffold26062, scaffold36944** |
| *Gavia stellata* | 4 | scaffold12476, scaffold17967**, scaffold48443**, scaffold50963** |
| *Geospiza fortis* | 10 | scaffold181, scaffold391, scaffold262, scaffold13, scaffold425, scaffold396 |
|  |  | scaffold657, scaffold897, scaffold1268, scaffold206** |
| *Haliaeetus albicilla* | 2 | scaffold35452, scaffold23856** |
| *Haliaeetus leucocephalus* | 2 | scaffold146, scaffold2403** |
| *Leptosomus discolor* | 3 | scaffold34810, scaffold6175**, scaffold17668 |
| *Manacus vitellinus* | 4 | scaffold21, scaffold565, scaffold406, scaffold129 |
| *Melopsittacus undulatus* | 38 | scf900160269947, scf900160276873, scf900160276922, scf900160276922* |
|  |  | scf900160276944, scf900160276944*, scf900160276949, scf900160276980 |
|  |  | scf900160277007, scf900160277009, scf900160277017, scf900160277021 |
|  |  | scf900160277026, scf900160277033, scf900160277038**, scf900160277038* |
|  |  | scf900160277043, scf900160277048, scf900160277052, scf900160277057 |
|  |  | scf900160277058, scf900160277059, scf900160277059*, scf900160277062 |
|  |  | scf900160277063, scf900160277064, scf900160277068**, scf900160277068* |
|  |  | scf900160277069, scf900160277070, scf900160277070*, scf900160277070* |
|  |  | scf900160277070*, scf900160277080, scf900160277086**, scf900160277086* |
|  |  | scf900160277086*, scf900160277086* |
| *Merops nubicus* | 2 | scaffold9374**, scaffold5729 |
| *Mesitornis unicolor* | 1 | scaffold28184 |
| *Nestor notabilis* | 5 | scaffold30203**, scaffold205, scaffold11426, scaffold33418, C14828811 |
| *Nipponia nippon* | 3 | scaffold60**, scaffold155, scaffold729 |
| *Opisthocomus hoazin* | 1 | scaffold15 |
| *Pelecanus crispus* | 2 | scaffold24122, scaffold18295** |
| *Phalacrocorax carbo* | 68 | scaffold2420, scaffold920, scaffold29610, scaffold10400, scaffold32010 |
|  |  | scaffold33570, scaffold581, scaffold6321, scaffold26701, scaffold451 |
|  |  | scaffold311, scaffold8271, scaffold43671, scaffold3941, scaffold34581 |
|  |  | scaffold3172, scaffold20432, scaffold58022, scaffold16932, scaffold352 |
|  |  | scaffold41102, scaffold4263, scaffold9913, scaffold57863, scaffold11093 |
|  |  | scaffold4763, scaffold24453, scaffold23754, scaffold21504, scaffold23034 |
|  |  | scaffold20175, scaffold23825, scaffold9105, scaffold8605, scaffold5895 |
|  |  | scaffold14725, scaffold19015, scaffold8716, scaffold12026, scaffold27156 |
|  |  | scaffold34026, scaffold3386, scaffold2006, scaffold9156, scaffold13616 |
|  |  | scaffold25906, scaffold12197, scaffold1857, scaffold23237, scaffold2597 |
|  |  | scaffold33047, scaffold7927, scaffold28087, scaffold14538, scaffold30778 |
|  |  | scaffold6878, scaffold7088, scaffold17438, scaffold24928, scaffold53998 |
|  |  | scaffold2378, scaffold14158, scaffold31259, scaffold52389, scaffold3549 |
|  |  | scaffold2949, scaffold18299, scaffold23119 |
| *Phaethon lepturus* | 2 | scaffold39851, scaffold3985** |
| *Phoenicopterus ruber* | 2 | scaffold37654, scaffold29997** |
| *Picoides pubescens* | 2 | scaffold148**, scaffold209 |
| *Podiceps cristatus* | 3 | scaffold6043, scaffold11994**, scaffold36720 |
| *Pterocles gutturalis* | 1 | scaffold28936** |
| *Pygoscelis adeliae* | 2 | scaffold465, scaffold14** |
| *Struthio camelus* | 2 | scaffold51, scaffold97 |
| *Taeniopygia guttata* | 13 | Chr:Un, Chr:24, Chr:1, Chr:1A*, Chr:19, Chr:20, Chr:12, Chr:8, Chr:6, Chr:6* |
|  |  | Chr:Un, Chr:26, Chr:Z |
| *Tauraco erythrolophus* | 1 | scaffold35184 |
| *Tinamus major* | 3 | scaffold4190, scaffold2594, scaffold9516 |
| *Tyto alba* | 5 | scaffold12462**, scaffold3534, scaffold37935**, scaffold38087, scaffold3677** |

* Different copy in same locus

** Containing complete viral genome; viruses sharing common ancestry were shaded gray and used for phylogenetic analysis.

Chr: Chromosome

Un: Unidentified

**Table S3.** Endogenous bornaviruses in avian genomes.

| **Name** | **Copy no.** | **Scaffold/locus** |
| --- | --- | --- |
| *Calypte anna* | 4 | scaffold5**, scaffold76***, scaffold76*^,^ **, scaffold29** |
| *Chaetura pelagica* | 1 | scaffold405** |
| *Picoides pubescens* | 1 | scaffold212** |

*Different copy in same locus

**EBLN

***EBLL

**Table S4.** Endogenous circoviruses in avian genomes.

| **Name** | **Copy no.** | **Scaffold/locus** |
| --- | --- | --- |
| *Egretta garzetta* | 1 | scaffold453** |
| *Geospiza fortis* | 1 | scaffold72** |
| *Nestor notabilis* | 1 | scaffold7610** |
| *Tinamus major* | 2 | C16032422*, scaffold13393** |

*eCiV-Cap

**eCiV-Rep

**Table S5.** Endogenous parvoviruses in avian genomes.

| **Name** | **Copy no.** | **Scaffold/locus** |
| --- | --- | --- |
| *Acanthisitta chloris* | 1 | scaffold6994** |
| *Chlamydotis macqueenii* | 1 | scaffold13103*** |
| *Corvus brachyrhynchos* | 2 | scaffold229**, scaffold61** |
| *Egretta garzetta* | 1 | scaffold81*** |
| *Manacus vitellinus* | 1 | scaffold270** |
| *Mesitornis unicolor* | 1 | scaffold28758*** |
| *Opisthocomus hoazin* | 1 | scaffold101** |
| *Pelecanus crispus* | 3 | scaffold56836***, scaffold56836*^,^ **, scaffold1346*** |
| *Pterocles gutturalis* | 1 | scaffold23885*** |
| *Taeniopygia guttata* | 1 | Chr:4** |

*Different copy in same locus

**ePaV-Cap

***ePaV-Rep

Chr: chromosome

**Table S6.** LTR-retrotransposon composition of avian genomes.

| **Species** | **Genome size (Gb)** | **Copy no.** | **% Genome** |
| --- | --- | --- | --- |
| *Acanthisitta chloris* | 1.045 | 47,700 | 1.260 |
| *Anas platyrhynchos* | 1.105 | 45,516 | 1.003 |
| *Antrostomus carolinensis* | 1.145 | 72,277 | 1.269 |
| *Apaloderma vittatum* | 1.086 | 36,297 | 0.857 |
| *Aptenodytes forsteri* | 1.258 | 53,226 | 1.090 |
| *Balearica regulorum* | 1.139 | 56,738 | 1.240 |
| *Buceros rhinoceros* | 1.105 | 42,583 | 0.712 |
| *Calypte anna* | 1.115 | 47,253 | 0.717 |
| *Cariama cristata* | 1.146 | 35,616 | 0.739 |
| *Cathartes aura* | 1.180 | 41,296 | 0.693 |
| *Chaetura pelagica* | 1.125 | 30,390 | 0.677 |
| *Charadrius vociferus* | 1.225 | 44,198 | 0.846 |
| *Chlamydotis macqueenii* | 1.095 | 52,745 | 1.210 |
| *Columba livia* | 1.112 | 33,190 | 0.625 |
| *Colius striatus* | 1.086 | 59,031 | 1.770 |
| *Corvus brachyrhynchos* | 1.095 | 77,178 | 2.076 |
| *Cuculus canorus* | 1.159 | 32,049 | 0.552 |
| *Egretta garzetta* | 1.212 | 51,358 | 1.278 |
| *Eurypyga helias* | 1.098 | 56,224 | 1.208 |
| *Falco peregrinus* | 1.174 | 38,416 | 1.120 |
| *Fulmarus glacialis* | 1.153 | 45,128 | 0.904 |
| *Gallus gallus* | 1.109 | 36,318 | 1.299 |
| *Gavia stellata* | 1.145 | 36,060 | 0.569 |
| *Geospiza fortis* | 1.073 | 106,906 | 2.851 |
| *Haliaeetus albicilla* | 1.145 | 53,877 | 1.267 |
| *Haliaeetus leucocephalus* | 1.259 | 92,956 | 1.761 |
| *Leptosomus discolor* | 1.148 | 42,536 | 0.852 |
| *Manacus vitellinus* | 1.160 | 45,363 | 0.913 |
| *Meleagris gallopavo* | 1.062 | 46,055 | 1.087 |
| *Melopsittacus undulatus* | 1.117 | 60,909 | 1.563 |
| *Merops nubicus* | 1.067 | 43,380 | 0.808 |
| *Mesitornis unicolor* | 1.101 | 49,272 | 0.833 |
| *Nestor notabilis* | 1.062 | 40,997 | 0.921 |
| *Nipponia nippon* | 1.240 | 61,061 | 1.160 |
| *Opisthocomus hoazin* | 1.209 | 51,694 | 1.037 |
| *Pelecanus crispus* | 1.169 | 57,945 | 1.337 |
| *Phalacrocorax carbo* | 1.155 | 51,236 | 1.171 |
| *Phaethon lepturus* | 1.167 | 56,347 | 1.223 |
| *Phoenicopterus ruber* | 1.145 | 44,767 | 0.854 |
| *Picoides pubescens* | 1.175 | 36,509 | 0.562 |
| *Podiceps cristatus* | 1.152 | 68,177 | 1.165 |
| *Pterocles gutturalis* | 1.080 | 52,013 | 0.948 |
| *Pygoscelis adeliae* | 1.226 | 51,137 | 1.297 |
| *Struthio camelus* | 1.228 | 25,865 | 0.162 |
| *Taeniopygia guttata* | 1.236 | 112,431 | 3.575 |
| *Tauraco erythrolophus* | 1.175 | 61,374 | 1.203 |
| *Tinamus major* | 1.060 | 24,451 | 0.240 |
| *Tyto alba* | 1.139 | 56,605 | 1.208 |

LTR-retrotransposon length is measured in bp.

LTR-retrotransposon categories: LTR/Caulimovirus, LTR/Copia, LTR/DIRS, LTR/ERV, LTR/ERV1, LTR/ERVK, LTR/ERVL, LTR/Gypsy, LTR/MaLR, LTR/LTR, LTR/Ngaro, LTR/Pao, LTR/TATE, and LTR/Viper.

**Table S7.** LTR-retrotransposon composition of American alligator, green turtle, anole lizard and mammalian genomes.

| **Species** | **Common name** | **Genome size (Gb)** | **Copy no.** | **% Genome** |
| --- | --- | --- | --- | --- |
| *Alligator mississippiensis* | American alligator | 2.173 | 195,126 | 2.935 |
| *Anolis carolinensis* | Carolina anole | 1.799 | 84,925 | 4.259 |
| *Chelonia mydas* | Green sea turtle | 2.236 | 89,472 | 0.799 |
| **Mammals** |  |  |  |  |
| *Bos taurus* | Cow | 2.670 | 560,310 | 4.688 |
| *Canis familiaris* | Dog | 2.532 | 403,562 | 4.664 |
| *Cavia porcellus* | Guinea pig | 2.723 | 765,847 | 6.603 |
| *Cricetulus griseus* | Chinese hamster | 2.400 | 1,037,743 | 7.972 |
| *Dipodomys ordii* | Kangaroo rat | 2.165 | 360,752 | 2.389 |
| *Equus caballus* | Horse | 2.475 | 532,902 | 6.790 |
| *Heterocephalus glaber* | Naked mole-rat | 2.665 | 753,027 | 6.198 |
| *Homo sapiens* | Human | 3.137 | 807,925 | 8.484 |
| *Ictidomys tridecemlineatus* | Squirrel | 2.478 | 582,659 | 3.834 |
| *Loxodonta africana* | Elephant | 3.197 | 512,689 | 4.336 |
| *Macaca mulatta* | Rhesus macaque | 3.097 | 645,746 | 7.575 |
| *Monodelphis domestica* | Opossum | 3.606 | 656,046 | 9.588 |
| *Mus musculus* | Mouse | 2.717 | 1,186,153 | 11.409 |
| *Myotis davidii* | Mouse-eared bat | 2.089 | 451,593 | 4.751 |
| *Oryctolagus cuniculus* | Rabbit | 2.738 | 543,903 | 4.459 |
| *Pan troglodytes* | Chimpanzee | 3.524 | 709,934 | 7.342 |
| *Pongo abelii* | Orangutan | 3.447 | 764,599 | 8.037 |
| *Pteropus alecto* | Black flying fox | 2.032 | 393,403 | 5.465 |
| *Rattus norvegicus* | Rat | 2.719 | 1,100,749 | 9.101 |
| *Sus scrofa* | Pig | 2.263 | 383,502 | 4.768 |

LTR-retrotransposon length is measured in bp.

LTR-retrotransposon categories: LTR/Caulimovirus, LTR/Copia, LTR/DIRS, LTR/ERV, LTR/ERV1, LTR/ERVK, LTR/ERVL, LTR/Gypsy, LTR/MaLR, LTR/LTR, LTR/Ngaro, LTR/Pao, LTR/TATE, and LTR/Viper.

**Table S9.** Reference sequences used for phylogenetic analyses.

| **Taxon and virus** | **Abbreviation** | **GenBank**  **Accession no.** |
| --- | --- | --- |
| **Hepadnaviridae** |  |  |
| ***Avihepadnavirus*** |  |  |
| *Duck hepatitis B virus* | DHBV | NC_001344 |
| *Parrot hepatitis B virus* | PHBV | NC_016561 |
| *Heron hepatitis B virus* | HHBV | NC_001486 |
| *Stork hepatitis B virus* | StHBV | AJ251937 |
| *Ross’s goose hepatitis B virus* | RGHBV | AY494849 |
| *Sheldgoose hepatitis B virus* | SHBV | NC_005890 |
| *Crane hepatitis B virus* | CHBV | AJ441112 |
| *Snow goose hepatitis B virus* | SgHBV | AF110997 |
| ***Orthohepadnavirus*** |  |  |
| *Bat hepatitis B virus* | BHBV1 | KC790373 |
|  | BHBV2 | KC790374 |
|  | BHBV3 | KC790375 |
|  | BHBV4 | KC790376 |
|  | BHBV5 | KC790378 |
|  | BHBV6 | KC790379 |
|  | BHBV7 | KC790380 |
|  | BHBV8 | KC790381 |
| *Gorilla hepatitis B virus* | GoHBV | AJ131567 |
| *Chimpanzee hepatitis B virus* | ChHBV | D00220 |
| *Gibbon hepatitis B virus* | GiHBV | U46935 |
| *Orangtan hepatitis B virus* | OHBV | AF193863 |
| *Human hepatitis B virus* | HuHBV | NC_003977 |
| *Wooly monkey hepatitis B virus* | WMHBV | AF046996 |
| *Woodchuck hepatitis B virus* | WHBV | NC_004107 |
| *Ground squirrel hepatitis B virus* | GSHBV | NC_001484 |
|  |  |  |
| **Bornaviridae** |  |  |
| *Borna disease virus* | BDV | NC_001607 |
| *Parrot bornavirus* | BVpar | FJ169441 |
| *Mute swan bornvirus* | BVmsw | JQ687270 |
| *Goose bornvirus* | BVgoo | JN251074 |
| *Avian bornavirus* | ABV | JX065210 |
| *Avian bornavirus 1* | ABV1 | FJ002326 |
| *Avian bornavirus 2* | ABV2 | HM998710 |
| *Avian bornavirus 3* | ABV3 | FJ002315 |
| *Avian bornavirus 4* | ABV4 | FJ002325 |
| *Avian bornavirus 5* | ABV5 | AB519144 |
| *Homo sapiens endogenous bornavirus-like 1* | Human EBLN1 | NM_001199938 |
| *Homo sapiens endogenous bornavirus-like 2* | Human EBLN2 | NM_018029 |
| *Macaca mulatta endogenous bornavirus-like 2* | Macaque EBLN2 | NM_001265951 |
| *Pan troglodytes endogenous bornavirus-like 1* | Chimpanzee EBLN1 | XM_521424 |
| *Pongo abelii endogenous bornavirus-like 2* | Orangutan EBLN1 | XP_002813563 |
|  |  |  |
| **Circoviridae** |  |  |
| *Bat circovirus* | BatCiV | JF938082 |
| *Silurus glanis circovirus* | SgCiV | JQ011378 |
| *Canine circovirus* | CanCiV | JQ821392 |
| *Muscovy duck circovirus 1* | DucCiV1 | GQ334371 |
| *Muscovy duck circovirus 2* | DucCiV2 | NC_006561 |
| *Mulard duck circovirus* | MudCiV | NC_005053 |
| *Goose circovirus* | GooCiV | NC_003054 |
| *Swan circovirus* | SwaCiV | EU056310 |
| *Finch circovirus* | FinCiV | NC_008522 |
| *Canary circovirus* | CaaCiV | NC_003410 |
| *Raven circovirus* | RavCiV | NC_008375 |
| *Chicken circovirus* | ChkCiV | HQ738642 |
| *Starling circivorus* | StaCiV | NC_008033 |
| *Chimpanzee circovirus* | ChiCiV | GQ404851 |
| *Gull circovirus* | GulCiV | NC_008521 |
| *Beak and feather disease virus* | BFDV | NC_001944 |
| *Rhinolophus ferrumequinum circovirus* | RfCiV | JQ814849 |
| *Porcine circovirus 1* | PorCiV1 | AF071879 |
| *Porcine circovirus 2* | PorCiV2 | NC_005148 |
| *Bovine circovirus* | BovCiV | AF109397 |
| *Barbel circovirus* | BarCiV | NC_015399 |
| *Labidocera aestiva circovirus* | LaCiV | NC_017843 |
|  |  |  |
| **Parvoviridae** |  |  |
| *Adeno-associated virus 1* | AAV1 | NC_002077 |
| *Adeno-associated virus 2* | AAV2 | NC_001401 |
| *Adeno-associated virus 3* | AAV3 | NC_001729 |
| *Adeno-associated virus 4* | AAV4 | NC_001829 |
| *Adeno-associated virus 5* | AAV5 | NC_006152 |
| *Adeno-associated virus 7* | AAV7 | NC_006260 |
| *Adeno-associated virus 8* | AAV8 | NC_006261 |
| *Avian adeno-associated virus* | AviAAV | NC_006263 |
| *Bovine adeno-associated virus* | BovAAV | NC_005889 |
| *Muscovy duck parvovirus* | DucPaV | NC_006147 |
| *Goose parvovirus* | GooPaV | NC_001701 |
| *Snake parvovirus 1* | SnaPaV1 | NC_006148 |
| *Bat adeno-associated virus* | BatAAV | NC_014468 |
| *California sea lion adeno-associated virus 1* | CalAAV1 | JN420372 |
| *Feline bocavius* | FelBoV | NC_017823 |
| *Porcine bocavirus 3* | PorBoV3 | NC_016031 |
| *Porcine bocavirus 4-1* | PorBoV4-1 | NC_016032 |
| *Porcine bocavirus 5* | PorBoV5 | NC_016647 |
| *Human bocavirus* | HumBoV | NC_007455 |
| *Human bocavirus 2* | HumBoV2 | NC_012042 |
| *Human bocavirus 3* | HumBoV3 | NC_012564 |
| *Human bocavirus 4* | HumBoV4 | NC_012729 |
| *Canine minute virus* | CMV | NC_004442 |
| *Minute virus of mice* | MVM | NC_001510 |
| *Bovine parvovirus* | BovPaV | NC_001540 |
| *Gorilla bocavirus* | GorBoV | NC_014358 |
| *Human erythrovirus* | HEV | NC_004295 |
| *Human parvovirus* | HumPaV | NC_000883 |
| *Mouse parvovirus 1* | MouPaV1 | NC_001630 |
| *Mouse parvovirus 2* | MouPaV2 | NC_008186 |
| *Mouse parvovirus 3* | MouPaV3 | NC_008185 |
| *Mouse parvovirus 4a* | MouPaV4a | NC_011619 |
| *Mouse parvovirus 5a* | MouPaV5a | NC_011618 |
| *LuIII virus* | LuV | NC_004713 |
| *Porcine parvovirus* | PorPaV | NC_001718 |
|  |  |  |
| **Retroviridae** |  |  |
| ***Alpharetrovirus*** |  |  |
| *Avian leukosis virus* | ALV | M37980 |
| *Rous sarcoma virus* | RSV | NC_001407 |
| ***Betaretrovirus*** |  |  |
| *Jaagsiekte sheep retrovirus* | JSV | M80216 |
| *Mason-Pfizer monkey virus* | MPMV | M12349 |
| *Simian retrovirus 1* | SRV1 | M11841 |
| *Simian retrovirus 2* | SRV2 | M16605 |
| *Mouse mammary tumor virus* | MMTV | M15122 |
| *Squirrel monkey retrovirus* | SMR | M23385 |
| ***Deltaretrovirus*** |  |  |
| *Bovine leukemia virus* | BLV | K02120 |
| *Human T-lymphotropic virus 1* | HTLV1 | NC_001436 |
| *Human T-lymphotropic virus 2* | HTLV2 | NC_001488 |
| *Simian T-lymphotropic virus 3* | STLV3 | NC_003323 |
| ***Epsilonretrovirus*** |  |  |
| *Walleye dermal sarcoma virus* | WDSV | AF033822 |
| *Walleye epidermal hyperplasia virus 1* | WEHV1 | AF014792 |
| *Walleye epidermal hyperplasia virus 2* | WEHV2 | AF014793 |
| ***Gammaretrovirus*** |  |  |
| *Reticuloendotheliosis virus* | REV | NC_006934 |
| *Feline leukemia virus* | FLV | NP_047255 |
| *Murine type C retrovirus* | M-CRV | NC_001702 |
| *Pre Xenotropic MuLV-related virus-1* | XMRV | NC_007815 |
| *Rauscher murine leukemia virus* | R-MuLV | NC_001819 |
| *Friend murine leukemia virus* | F-MuLV | NC_001362 |
| *Moloney murine leukemia virus* | M-MuLV | NC_001501 |
| *RD114 retrovirus* | RD114 | NC_009889 |
| *Baboon endogenous virus* | BaEV | D10032 |
| *Killer whale endogenous retrovirus* | KWERV | GQ222416 |
| *Gibbon ape leukemia virus* | GALV | NC_001885 |
| *Koala retrovirus* | KoRV | AF151794 |
| *Mus dunni endogenous virus* | MDEV | AF053745 |
| *Porcine endogenous retrovirus A* | PERV-A | AJ293656 |
| *Porcine endogenous retrovirus B* | PERV-B | AY099324 |
| *Porcine endogenous retrovirus C* | PERV-C | AJ293656 |
| *Rhinolophus ferrumequinum retrovirus* | RfRV | JQ303225 |
| ***Lentivirus*** |  |  |
| *Visna/maedi virus* | VMV | M60609 |
| *Caprine arthritis encephalitis virus* | CAEV | M33677 |
| *Simian immunodeficiency virus* | SIV | X07805 |
| *Human immunodeficiency virus 1* | HIV1 | M62320 |
| *Human immunodeficiency virus 2* | HIV2 | NC_001722 |
| *Puma lentivirus* | PLV | U03982 |
| *Feline immunodeficiency virus* | FIV | NC_001482 |
| *Equine infectious anemia virus* | EIAV | M16575 |
| *Bovine immunodeficiency virus* | BIV | M32690 |
| ***Spumavirus*** |  |  |
| *Macaque simian foamy virus* | SFVmac | X54482 |
| *African green monkey simian foamy virus* | SFVagm | M74895 |
| *Chimpanzee simian foamy virus* | SFVcpz | NC_001364 |
| *Bovine foamy virus* | BFV | U94514 |
| *Equine foamy virus* | EFV | AF201902 |
| *Feline foamy virus* | FFV | Y08851 |
| **Unclassified retrovirus** |  |  |
| *Snakehead retrovirus* | SnRV | U26458 |
