# Supplementary figures and images for: Low frequency of paleoviral infiltration across the avian phylogeny
Source: Genome Biol. 2014 Dec 11;15(12):539. doi: 10.1186/s13059-014-0539-3 (PMC4272516; doi:10.1186/s13059-014-0539-3)

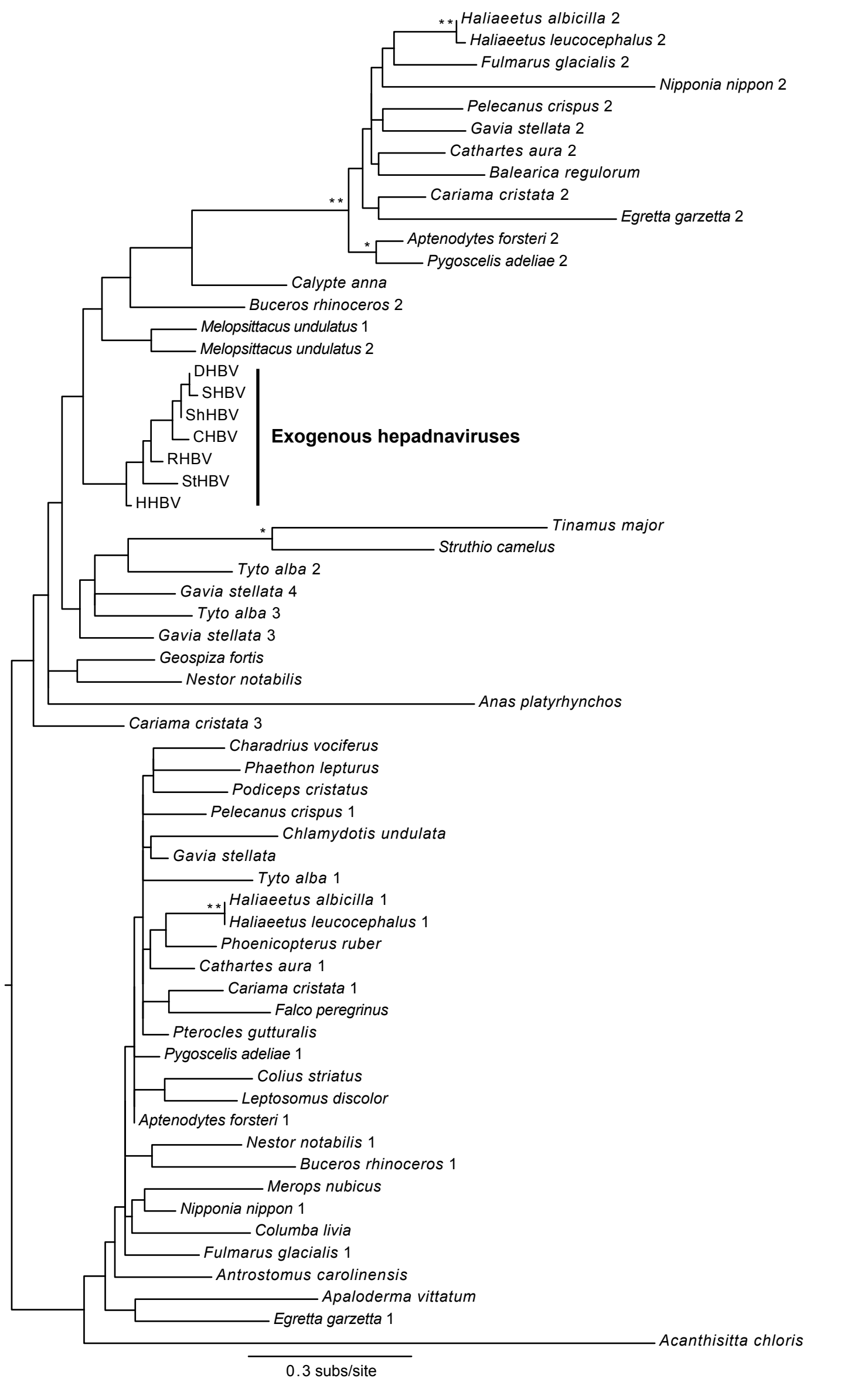

Supplement: Additional file 3: Figure S2 — Phylogenetic tree of exogenous and endogenous avian hepadnaviruses. Bootstrap values lower than 70% are not shown; single asterisks indicate values higher than 70%, while double asterisks indicate values higher than 90%. Branch lengths are drawn to a scale of amino acid substitutions per site (subs/site). The tree is midpoint rooted for purposes of clarity only. The exogenous hepadnaviruses are highlighted. Avian host species names are used to denote avian endogenous hepadnaviruses, and different EVEs from the same host are numbered. All abbreviations are provided in Table S9 in Additional file 1. [file 13059_2014_539_MOESM3_ESM.pdf]

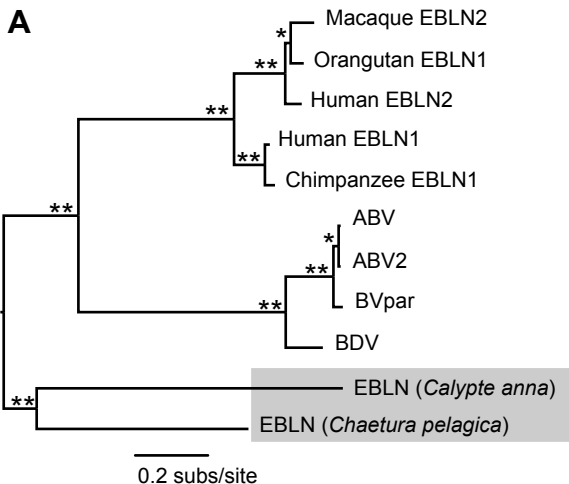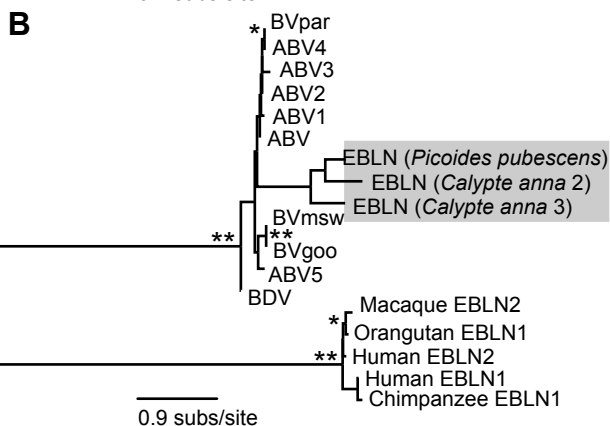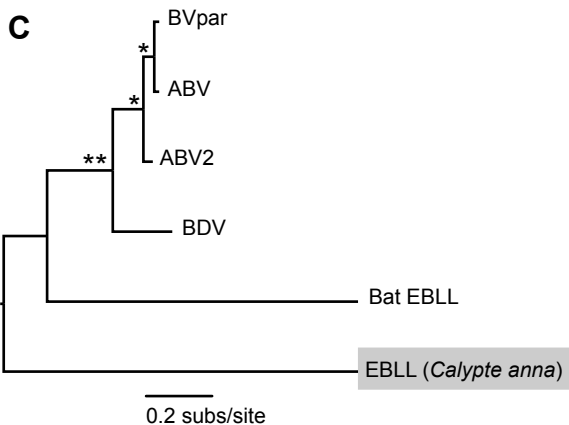

Supplement: Additional file 6: Figure S4 — Phylogenetic trees of endogenous and exogenous bornaviruses. The phylogenies contain (A) endogenous bornavirus-like N (nucleoprotein) (EBLN) and (B) avian endogenous bornavirus-like L (RNA-dependent RNA polymerase) (EBLL) sequences. Bootstrap values lower than 70% are not shown; single asterisks indicate values higher than 70%, while double asterisks indicate values higher than 90%. Branch lengths are drawn to a scale of amino acid substitutions per site (subs/site). The trees are midpoint rooted for purposes of clarity only. Avian host species names for those that harbor EVEs are given in parentheses and different EVEs from the same host are numbered. All abbreviations are provided in Table S9 in Additional file 1. [file 13059_2014_539_MOESM6_ESM.pdf]

**A**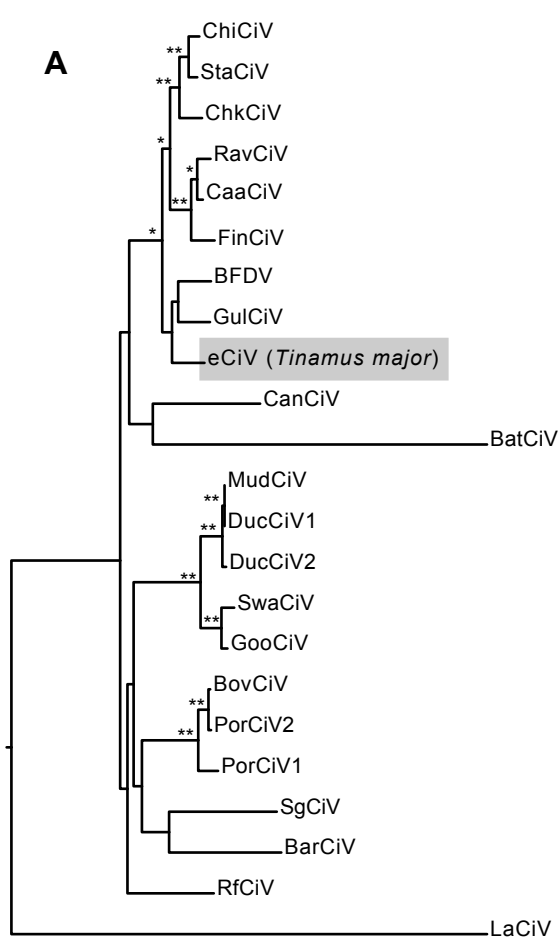**B**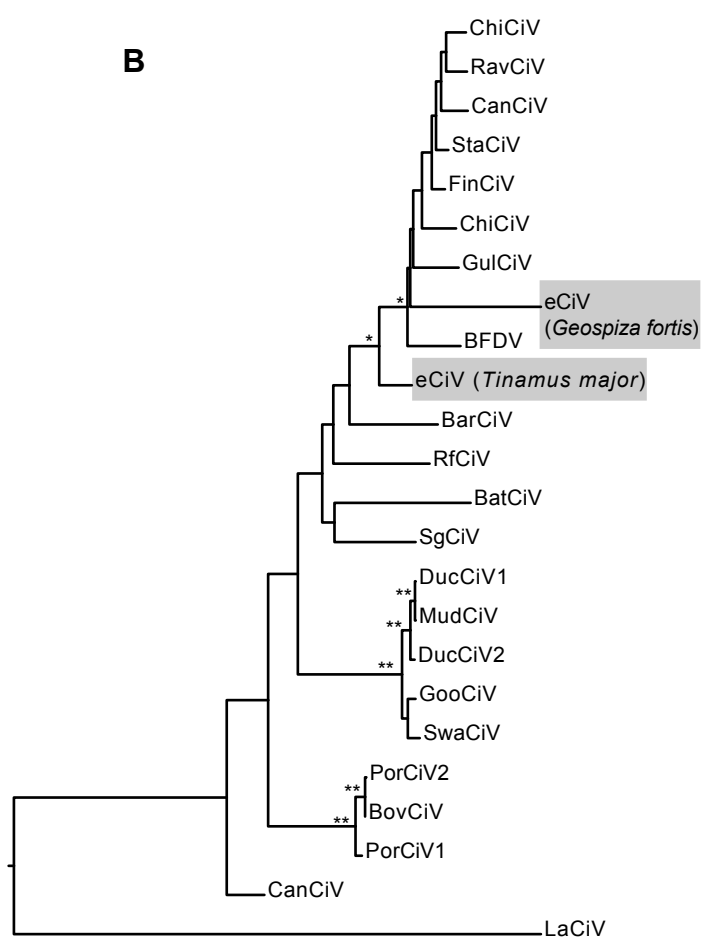**C**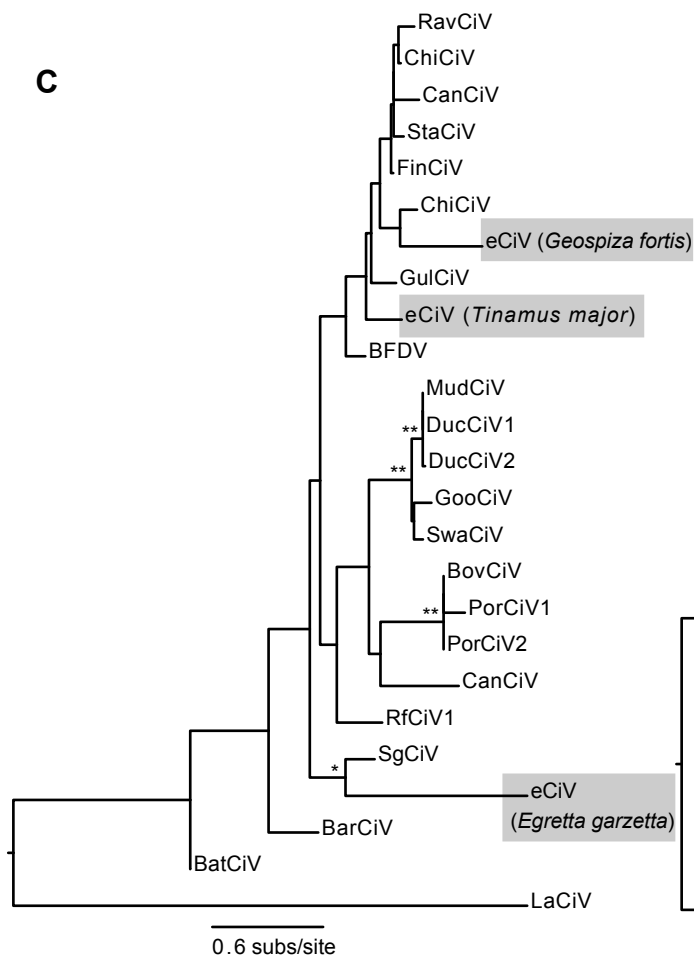**D**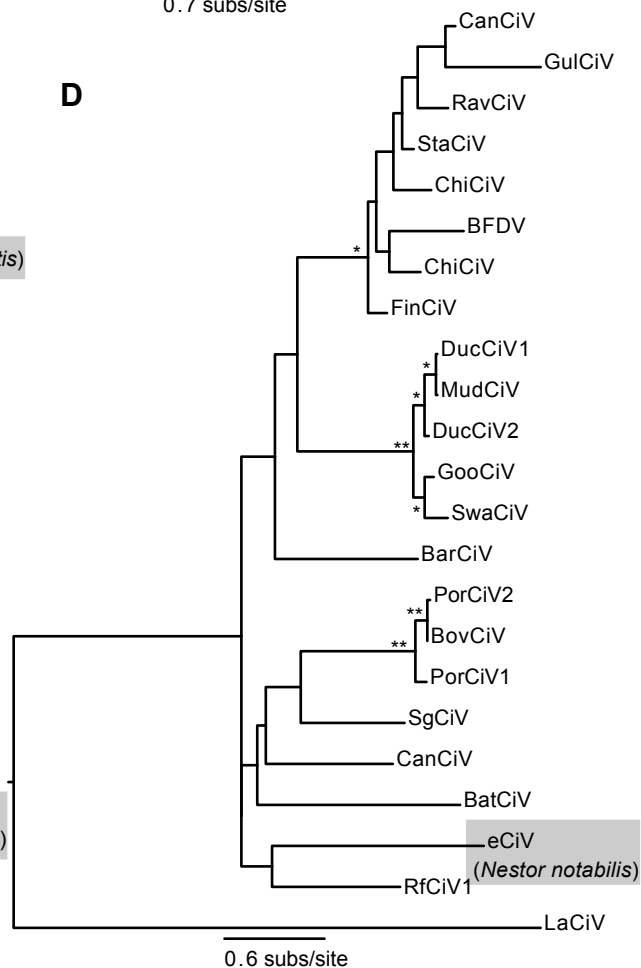

Supplement: Additional file 7: Figure S5 — Phylogenetic trees of endogenous circoviruses. (A-D) The phylogenies contain avian endogenous circoviruses (eCiVs) Cap (A) and Rep (B-D). Bootstrap values lower than 70% are not shown; single asterisks indicate values higher than 70%, while double asterisks indicate values higher than 90%. Branch lengths are drawn to a scale of amino acid substitutions per site (subs/site). The trees are midpoint rooted for purposes of clarity only. Avian host species names for those that harbor EVEs are given in parentheses. All abbreviations are provided in Table S9 in Additional file 1. [file 13059_2014_539_MOESM7_ESM.pdf]

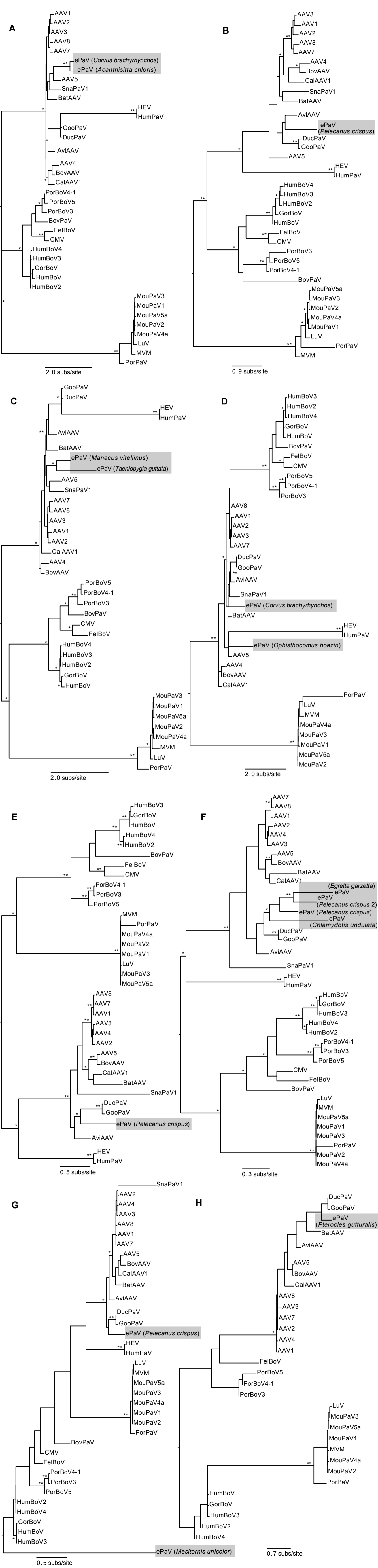

Supplement: Additional file 8: Figure S6 — Phylogenetic trees of endogenous and exogenous parvoviruses. (A-H) The phylogenies contain avian endogenous parvoviruses (ePaVs) Cap (A-D) and Rep (E-H). Bootstrap values lower than 70% are not shown; single asterisks indicate values higher than 70%, while double asterisks indicate values higher than 90%. Branch lengths are drawn to a scale of amino acid substitutions per site (subs/site). The trees are midpoint rooted for purposes of clarity only. Avian host species names for those that harbor EVEs are given in parentheses and different EVEs from the same host are numbered. All abbreviations are provided in Table S9 in Additional file 1. [file 13059_2014_539_MOESM8_ESM.pdf]
