# Supplementary material for: Low frequency of paleoviral infiltration across the avian phylogeny
Source: Genome Biol. 2014 Dec 11;15(12):539. doi: 10.1186/s13059-014-0539-3 (PMC4272516; doi:10.1186/s13059-014-0539-3)
Supplement: Additional file 4 — Data S1. Alignments of the orthologous hepadnaviral scaffolds. Data S2. Alignments of the orthologous parvoviral scaffolds. [file 13059_2014_539_MOESM4_ESM.docx]

**Supplemental Data S1**

Alignments of the orthologous scaffolds, including viral sequences that matching *Hepadnaviridae* which are shaded and their flanking regions. The dot denotes the same nucleotide aligned; the dash denotes deletion. WTE, white-tailed eagle (*Haliaeetus albicilla*); BE, bald eagle (*H. leucocephalus*); EP, emperor penguin (*Aptenodytes forsteri*); AP, Adelie penguin (*Pygoscelis adeliae*); OS, ostrich (*Struthio camelus*); and GT, great tinamou (*Tinamus major*).

**(i) *Haliaeetus albicilla* 1 and** ***H. leucocephalus* 1**

10 20 30 40 50 60 70 80 90 100

....|....|....|....|....|....|....|....|....|....|....|....|....|....|....|....|....|....|....|....|

WTE_scaffold23856 TCCAAAGTGTAATTGAGAACTGTCTAGATGGTGCCTTCAGCAAGGAAACTGAAATGAAATCCATTTGTTAGAATATAGAATCCAAAAGTTTCCTTGTTTT

BE_scaffold2403 ....................................................................................................

110 120 130 140 150 160 170 180 190 200

....|....|....|....|....|....|....|....|....|....|....|....|....|....|....|....|....|....|....|....|

WTE_scaffold23856 TGTTTTGGTTTTTTTTTTTCCTTGAAAAAGCTTTGTGTTGATCTTAAACTGGAAAAAAAGAGGGAGGAAATGGAGAGTGACATGTAAACATTTGAAAGAA

BE_scaffold2403 ..................-.................................................................................

210 220 230 240 250 260 270 280 290 300

....|....|....|....|....|....|....|....|....|....|....|....|....|....|....|....|....|....|....|....|

WTE_scaffold23856 GGTGAAAATAACAAGTTAGATTTGGTCTATTTATCTCAGATTTTAAAAGCATACCAGTGCATTACTTTCTAAAATGCTGGACTGCAGTTGAAAAAGTAGA

BE_scaffold2403 ....................................................................................................

310 320 330 340 350 360 370 380 390 400

....|....|....|....|....|....|....|....|....|....|....|....|....|....|....|....|....|....|....|....|

WTE_scaffold23856 ACACAATCTCCTGTTATCTGTATTTCTGTATTTGAAGAAGGCATATAATATGAATTACAACATATCTCCTAAATATTATGTTTCCTGGTTTGATTTTGCA

BE_scaffold2403 ....................................................................................................

410 420 430 440 450 460 470 480 490 500

....|....|....|....|....|....|....|....|....|....|....|....|....|....|....|....|....|....|....|....|

WTE_scaffold23856 GTGATGAACAGCAGAGGGACTATTTAATGGAGAGAAGAGATCTGGCTATTGATTTTATTTTTTCTTTAGTATTAATAGAAGTTTTGAAACAGGTATGGAG

BE_scaffold2403 ......................................................................T.............................

510 520 530 540 550 560 570 580 590 600

....|....|....|....|....|....|....|....|....|....|....|....|....|....|....|....|....|....|....|....|

WTE_scaffold23856 TTCTTAAATTACCCATTAACTAATAACTGATGGAAATTACCAATACAAACGAATTTTAGGATTCTTTTTCTATTTCCATCTTTAAGAGTCTAATAAAAAC

BE_scaffold2403 ....................................................................................................

610 620 630 640 650 660 670 680 690 700

....|....|....|....|....|....|....|....|....|....|....|....|....|....|....|....|....|....|....|....|

WTE_scaffold23856 ATGAGATACATCCTGCATGCTTGTTCATTGCTGTCTTTCTTGCACGTGTAGGATACATAGTATTATGTGTTCATACAGACTTCACTTTGCCAAGTCTTTG

BE_scaffold2403 ....................................................................................................

710 720 730 740 750 760 770 780 790 800

....|....|....|....|....|....|....|....|....|....|....|....|....|....|....|....|....|....|....|....|

WTE_scaffold23856 GAGCTGAAGTATTACCCTTCAACACAAAATAAACTTTAAATGACTGGCTCATTTCAGCTGTTGTGTTAGTTAGATGCTATTTCAGCTGATACCATACTTC

BE_scaffold2403 .....A..............................................................................................

810 820 830 840 850 860 870 880 890 900

....|....|....|....|....|....|....|....|....|....|....|....|....|....|....|....|....|....|....|....|

WTE_scaffold23856 TGTATGGAAGATACTCATGACTAATACTAGCACTACGGTATGCAGCTTGAAGTGTAATATATGTGTACTCGTTATGTTTTATTCTGTGTTTTCAGATTCC

BE_scaffold2403 ....................................................................................................

910 920 930 940 950 960 970 980 990 1000

....|....|....|....|....|....|....|....|....|....|....|....|....|....|....|....|....|....|....|....|

WTE_scaffold23856 ACTTCATCCTGTAATAGACAGTTTGGTACATGATGTTATTAACTTGGCTTTCAAGCACTTTAAGTACAAAGAAGGGTAAGGCTCTTTTTCCTACAAAAAT

BE_scaffold2403 ....................................................................................................

1010 1020 1030 1040 1050 1060 1070 1080 1090 1100

....|....|....|....|....|....|....|....|....|....|....|....|....|....|....|....|....|....|....|....|

WTE_scaffold23856 TCTGTGGGAAAAAAAAAATCACTTTATTTTTTAATAGTAGTGGTTTAAGCAGTATGCTTTCTCAGCCAAGAATTCTTTCTTGTCTCTTCCTAGGTACCTT

BE_scaffold2403 ....................................................................................................

1110 1120 1130 1140 1150 1160 1170 1180 1190 1200

....|....|....|....|....|....|....|....|....|....|....|....|....|....|....|....|....|....|....|....|

WTE_scaffold23856 GGTCCTAACACTGGAAATATGCACATTGTTGCGGATTTGTATGCAGAAGTAATAGGTGTTCTAGCTCAAGCAAAGTAAGCAAAATTGAATATCGTATTTC

BE_scaffold2403 ....................................................................................................

1210 1220 1230 1240 1250 1260 1270 1280 1290 1300

....|....|....|....|....|....|....|....|....|....|....|....|....|....|....|....|....|....|....|....|

WTE_scaffold23856 CCCCATAAATCTGAGAAGTTCTACTCGCTTAGCGTGTGCATATTCTGAGTTTTTCACTTTCTTAAAGAATAGTGTAGGAATAAATCATTTCAGCTCGTTG

BE_scaffold2403 ....................................................................................................

1310 1320 1330 1340 1350 1360 1370 1380 1390 1400

....|....|....|....|....|....|....|....|....|....|....|....|....|....|....|....|....|....|....|....|

WTE_scaffold23856 GCCCTCTATTCATCCAGATGTTCTAGAGCATACAACTCTAGGGAAGCACAGGCTTTCTGCATCTAGTCTGGTGATACTGAGTAAATTCAACTCGACTACA

BE_scaffold2403 ....................................................................................................

1410 1420 1430 1440 1450 1460 1470 1480 1490 1500

....|....|....|....|....|....|....|....|....|....|....|....|....|....|....|....|....|....|....|....|

WTE_scaffold23856 GCAGCATTATTTTTCTTATCTCAGCAATGACGTCTGTGGAAAGCCCAAAGGGCTGAAGAGAACTATTTTAGTTATTTTTTTTCTGTATGCTGTTTTTTCT

BE_scaffold2403 ....................................................................................................

1510 1520 1530 1540 1550 1560 1570 1580 1590 1600

....|....|....|....|....|....|....|....|....|....|....|....|....|....|....|....|....|....|....|....|

WTE_scaffold23856 CTAGCTGGGCAAACCTAAAAATGGCTATCCTCCAACGTAGCAACATTTTTAAGTCTCTCTGCAGTTATTTCAGTTCAGAACTTAATGCAGCTGCTTCTGA

BE_scaffold2403 ....................................................................................................

1610 1620 1630 1640 1650 1660 1670 1680 1690 1700

....|....|....|....|....|....|....|....|....|....|....|....|....|....|....|....|....|....|....|....|

WTE_scaffold23856 ATCCTTCATCCTGCACTTCTTAGAGCAGATGACATGAACCACTTCGTGGATCTGGTCTTGAATTTTGAATCTGGAAGCAGACAGCTTCAGAAACTCATGT

BE_scaffold2403 ...................................A................................................................

1710 1720 1730 1740 1750 1760 1770 1780 1790 1800

....|....|....|....|....|....|....|....|....|....|....|....|....|....|....|....|....|....|....|....|

WTE_scaffold23856 ACCCAATGTAACATTTCATCATATGCTTACACCACAAACTGAGGAGAGGGTGAAATTTACTGCCGTGACACGGTGTTCTCTGTCTCCTGACTTCTTGGTG

BE_scaffold2403 ....................................................................................................

1810 1820 1830 1840 1850 1860 1870 1880 1890 1900

....|....|....|....|....|....|....|....|....|....|....|....|....|....|....|....|....|....|....|....|

WTE_scaffold23856 TTGCTGAGAGTGACTCTGCTCTACAAAACCAATCAACTTAGCTTGATGTAGCCAAAACAGTTTGAGTTCCTGGAAATCATACAATTTTCACAGACTTCAG

BE_scaffold2403 ....................................................................................................

1910 1920 1930 1940 1950 1960 1970 1980 1990 2000

....|....|....|....|....|....|....|....|....|....|....|....|....|....|....|....|....|....|....|....|

WTE_scaffold23856 CTAAGGCCTTACCTGTGTTTCTAAATTGGCTGGGTCAAAGGCAAAAGGGGAAGTGACATCTGGATCCAAAGCTTCAAATTGACTGTCAAAGGTAGAACAT

BE_scaffold2403 ....................................................................................................

2010 2020 2030 2040 2050 2060 2070 2080 2090 2100

....|....|....|....|....|....|....|....|....|....|....|....|....|....|....|....|....|....|....|....|

WTE_scaffold23856 ACCTGTTCTCCATAAATACTGCTTTTCTTCTCCAGTTCTGAAGGGCCTTGCTAAGATGTTACTACCACTAAAGGACTTCTTCTTGCCAACCTGTTTCTTT

BE_scaffold2403 ....................................................................................................

2110 2120 2130 2140 2150 2160 2170 2180 2190 2200

....|....|....|....|....|....|....|....|....|....|....|....|....|....|....|....|....|....|....|....|

WTE_scaffold23856 AAACTCTCCTGTGCTGTCCATCATAGGCGATAACAAAAGGTTGTTATGTGCTACACCTGCCCATGAAAATGACATTCTTTTCATTTTGTCTCAAAAAGAA

BE_scaffold2403 ....................................................................................................

2210 2220 2230 2240 2250 2260 2270 2280 2290 2300

....|....|....|....|....|....|....|....|....|....|....|....|....|....|....|....|....|....|....|....|

WTE_scaffold23856 CAGGAGGTCCTGTTGGATGATCTTCCTATATGTTATGTTTATGGGCAGTGTGTCTCAAAAGACACCTCCAATAGATTTGTGGGGGAGATAAAAAATGGTC

BE_scaffold2403 ....................................................................................................

2310 2320 2330 2340 2350 2360 2370 2380 2390 2400

....|....|....|....|....|....|....|....|....|....|....|....|....|....|....|....|....|....|....|....|

WTE_scaffold23856 ACAGACCCATCCATCCCAAGTTGTCGTAGTTAATGCTTAAGTGTTACTGTAATGGCTATTAGAAAAAACCTGAAGGGAGGAACAGCCTATCTGAGTATTT

BE_scaffold2403 ....................................................................................................

2410 2420 2430 2440 2450 2460 2470 2480 2490 2500

....|....|....|....|....|....|....|....|....|....|....|....|....|....|....|....|....|....|....|....|

WTE_scaffold23856 TCATAGTTATAATTTCAGCATTTTCTTTTGAAAATATTGCATGTACTTGCAAGAAAACTATCTTTCATCTTGGATTTTTGAAAAACACGTGAGGGGGTTA

BE_scaffold2403 ....................................................................................................

2510 2520 2530 2540 2550 2560 2570 2580 2590 2600

....|....|....|....|....|....|....|....|....|....|....|....|....|....|....|....|....|....|....|....|

WTE_scaffold23856 TTCTAATGATATTCTTAGCTCTCATGCAAAAGAGGGGCATGGTAAGAAAATAATTTGAAAGATGTGGTTATCTAAAGGGACTAAGGACAGGCTGTACACT

BE_scaffold2403 ....................................................................................................

2610 2620 2630 2640 2650 2660 2670 2680 2690 2700

....|....|....|....|....|....|....|....|....|....|....|....|....|....|....|....|....|....|....|....|

WTE_scaffold23856 TTATTTTCTTTTCTGAGTTTATCAGAAGTGAAAGAAAACTGAACAAAAAGAAATCTATTCACATACTGACTGAAATGATGTTTTACAACCAAAGTTGAAT

BE_scaffold2403 ....................................................................................................

2710 2720 2730 2740 2750 2760 2770 2780 2790 2800

....|....|....|....|....|....|....|....|....|....|....|....|....|....|....|....|....|....|....|....|

WTE_scaffold23856 CTTCTTTAGGTTTTTCCCCATTTATATAGCTTACAATACATTTCAAATATTAATGTGTACTTTTTTGTAACTAACCAATGCGAATTCACAGGTTTCCTGC

BE_scaffold2403 ....................................................................................................

2810 2820 2830 2840 2850 2860 2870 2880 2890 2900

....|....|....|....|....|....|....|....|....|....|....|....|....|....|....|....|....|....|....|....|

WTE_scaffold23856 TGTAAAGAAGAAATTTATGGCAGAGTTAAAAGAGTTACGGCATAAAGAACAAAGCCCATATATAGTTCAAAGCATTATCAGTCTTATAATGGGAATGAAA

BE_scaffold2403 ....................................................................................................

2910 2920 2930 2940 2950 2960 2970 2980 2990 3000

....|....|....|....|....|....|....|....|....|....|....|....|....|....|....|....|....|....|....|....|

WTE_scaffold23856 TTCTTTCGCATTAAGATGTATCCTGTGGAGGACTTTGAAGCTTCTCTTCAGTTTATGCAGGTAAAATTCTTAAGTATACAACTATGGTTCCCAGACCTTT

BE_scaffold2403 ....................................................................................................

3010 3020 3030 3040 3050 3060 3070 3080 3090 3100

....|....|....|....|....|....|....|....|....|....|....|....|....|....|....|....|....|....|....|....|

WTE_scaffold23856 TAGAATGGGCTCCCCTTCATCTTTCCTGTAATACTCCAAGATATTTTCCATGTGTCATTCTCACATAAACGTATGTGCAGCTACTTTCTGCTTGTTCTGA

BE_scaffold2403 ....................................................................................................

3110 3120 3130 3140 3150 3160 3170 3180 3190 3200

....|....|....|....|....|....|....|....|....|....|....|....|....|....|....|....|....|....|....|....|

WTE_scaffold23856 ACCTCAGCGCTCCAGTCTCCAATCTCTGGAGTGTATCATTCCATACCAACTTTTCATCAGCTGTATACCCCTAGTCTGTACATCTCACCCTCCAACAAAA

BE_scaffold2403 ....................................................................................................

3210 3220 3230 3240 3250 3260 3270 3280 3290 3300

....|....|....|....|....|....|....|....|....|....|....|....|....|....|....|....|....|....|....|....|

WTE_scaffold23856 GGAGTTTCTGTTCTGTAGACCCCAGACCTTCCACCCTGCCCCATGTCCCTGCCTAGTTATATCTCCATAGCTGCTCACTGCCTCCAAAGTGAATCTTCCA

BE_scaffold2403 ....................................................................................................

3310 3320 3330 3340 3350 3360 3370 3380 3390 3400

....|....|....|....|....|....|....|....|....|....|....|....|....|....|....|....|....|....|....|....|

WTE_scaffold23856 CCATTGACATAGCGGCCAGTGAGGAGAGGACCTTCTGATGGAGAAGAACACAGAGGAAGAGACGAAGAATTGAGGCAAAAGCTGGATGGAATTTCCCTTG

BE_scaffold2403 ....................................................................................................

3410 3420 3430 3440 3450 3460 3470 3480 3490 3500

....|....|....|....|....|....|....|....|....|....|....|....|....|....|....|....|....|....|....|....|

WTE_scaffold23856 GATATGTTGTCTCCTTCCACCACACCTTCACTCCCCTTTCTGTAGGGTACATTCTGTACTCACCTGGTTTGAGAGCCACTAGTACAGAGCATAGTTTGAC

BE_scaffold2403 ....................................................................................................

3510 3520 3530 3540 3550 3560 3570 3580 3590 3600

....|....|....|....|....|....|....|....|....|....|....|....|....|....|....|....|....|....|....|....|

WTE_scaffold23856 TCTCATGAATGCTTTAAATTAATAGATTACATCATTTTGTAAAAGGAAAGAAATATTTTATGTTTAAACATTTTAAAAGTCCACATTCTTGTTGGTAATG

BE_scaffold2403 ....................................................................................................

3610 3620 3630 3640 3650 3660 3670 3680 3690 3700

....|....|....|....|....|....|....|....|....|....|....|....|....|....|....|....|....|....|....|....|

WTE_scaffold23856 TACAATGGGTCTGTTCCCATCAGCAGGTTTATTTAGCTTAACCTTTTTACTCCCCTGTTTTTTCTTGGGCTCACCCTCACAGCTACATGGTTTGTAGCCA

BE_scaffold2403 ....................................................................................................

3710 3720 3730 3740 3750 3760 3770 3780 3790 3800

....|....|....|....|....|....|....|....|....|....|....|....|....|....|....|....|....|....|....|....|

WTE_scaffold23856 CTCATCCAGTAAAATACATGTTTTATATAAACCTCTGTGTAAAGATCAGCTTATTGATGGTGTCAGTGGAGTAAAAAATCAATTTTAGATGCCCACAAGT

BE_scaffold2403 ....................................................................................................

3810 3820 3830 3840 3850 3860 3870 3880 3890 3900

....|....|....|....|....|....|....|....|....|....|....|....|....|....|....|....|....|....|....|....|

WTE_scaffold23856 AAAGATTTGACGTTGGAATACAAATACACAGGCGTATAGACACACAGAGCCATTTCTCTTAAGTCCTGAACAGGAAGCACATTCTATTTCAGTTTTTCAA

BE_scaffold2403 ....................................................................................................

3910 3920 3930 3940 3950 3960 3970 3980 3990 4000

....|....|....|....|....|....|....|....|....|....|....|....|....|....|....|....|....|....|....|....|

WTE_scaffold23856 TGTGTTTTAAACCATATTTAACAACATATTTTAAACATATTTAACAACACAGAAGCAGCTCATTTTTCTTCTTACCTCACACTTCATTATATGAACACAT

BE_scaffold2403 ....................................................................................................

4010 4020 4030 4040 4050 4060 4070 4080 4090 4100

....|....|....|....|....|....|....|....|....|....|....|....|....|....|....|....|....|....|....|....|

WTE_scaffold23856 CTTAGTATGTGTTGACTTAATGTAAGTTATCCAGAGCTGGCAAGATATCAAAAGTAGTGTGTGTTCAGTGCACATGACTAATATGAGTCAATGACTTTGT

BE_scaffold2403 ....................................................................................................

4110 4120 4130 4140 4150 4160 4170 4180 4190 4200

....|....|....|....|....|....|....|....|....|....|....|....|....|....|....|....|....|....|....|....|

WTE_scaffold23856 GGCACAGCACTGCTGGGATCCAGTGTTCACTGCTATTGAAATCAGTGGTAAAGCTCCCATATAGTAGAACTCAAAATTTGGATGCAGATGATCTCTGCCT

BE_scaffold2403 ....................................................................................................

4210 4220 4230 4240 4250 4260 4270 4280 4290 4300

....|....|....|....|....|....|....|....|....|....|....|....|....|....|....|....|....|....|....|....|

WTE_scaffold23856 TCCCTGACTATAGTATCTCAGCACGAAAATAATTCTTAGATATACTACAATGCATCATAAGGTGCACTGTGTATGTAGGAAAGCCAGTTTTGCTCAGTTT

BE_scaffold2403 ....................................................................................................

4310 4320 4330 4340 4350 4360 4370 4380 4390 4400

....|....|....|....|....|....|....|....|....|....|....|....|....|....|....|....|....|....|....|....|

WTE_scaffold23856 GGTGAAATACAGTTTGATTCTTTGGGCGATTTCTCTGTTAGTTTGAGTATCTACATGCAGTGCTTCAGCTACAAGCTCTTGTCACCAGCTCTGCTCTAGT

BE_scaffold2403 ....................................................................................................

4410 4420 4430 4440 4450 4460 4470 4480 4490 4500

....|....|....|....|....|....|....|....|....|....|....|....|....|....|....|....|....|....|....|....|

WTE_scaffold23856 AGAGAGCCACACTTTCAGAAGGTAAGCTAACCTCAGGAGCCAGCTCCAGAAGAGCCTCTCTGCCACCATGGAGTGCAGAAGCAGCTCAGGAAGGCTGCTG

BE_scaffold2403 ....................................................................................................

4510 4520 4530 4540 4550 4560 4570 4580 4590 4600

....|....|....|....|....|....|....|....|....|....|....|....|....|....|....|....|....|....|....|....|

WTE_scaffold23856 CCTTTGCATAGGGGCCTTCAGCTGTGTAGCAGAAATGGCTGGAGTGACTGAAGCCATGTGGTGTAGAATCCTGATTGCAGCCAGGAAGAATCAGGCATGG

BE_scaffold2403 ....................................................................................................

4610 4620 4630 4640 4650 4660 4670 4680 4690 4700

....|....|....|....|....|....|....|....|....|....|....|....|....|....|....|....|....|....|....|....|

WTE_scaffold23856 TCTAAAGTCTGAACTGCTCTGCGCGCGTGTGCAGCAAAAAAGGTCAGTGGGGACCTTCCTTGTAAAGTGAGTGGTTGTTTGGGTCTTGGGCCCAGAAAGG

BE_scaffold2403 ..................................G.................................................................

4710 4720 4730 4740 4750 4760 4770 4780 4790 4800

....|....|....|....|....|....|....|....|....|....|....|....|....|....|....|....|....|....|....|....|

WTE_scaffold23856 AAGAAGTAACTGGAGCAACACGAGTTGTCATCCTCAGAGAAGTTGTACAGAAACATGAGGGGATGCTTATCTGCCCTCCCTCACTTCCTAAGCAGCACAG

BE_scaffold2403 ....................................................................................................

4810 4820 4830 4840 4850 4860 4870 4880 4890 4900

....|....|....|....|....|....|....|....|....|....|....|....|....|....|....|....|....|....|....|....|

WTE_scaffold23856 CTGTGTGGATAGTAGTTCGCTGCATGTTTAGGACAAAAGTACGTTTCTACTTTCTCTCTTAATCCCCAATTCCAGGCTGGAAATTGTGGTGGTTTGCACC

BE_scaffold2403 ....................................................................................................

4910 4920 4930 4940 4950 4960 4970 4980 4990 5000

....|....|....|....|....|....|....|....|....|....|....|....|....|....|....|....|....|....|....|....|

WTE_scaffold23856 ACAGGTCTCATCCCTCACATAGCACAGTTCTATATTGACCCTTAATCTGCGGTGGATCCTAGCAGTGAAATTTATCCCCAGATAATTACGGTAAGATTGT

BE_scaffold2403 ....................................................................................................

5010 5020 5030 5040 5050 5060 5070 5080 5090 5100

....|....|....|....|....|....|....|....|....|....|....|....|....|....|....|....|....|....|....|....|

WTE_scaffold23856 TCCTTACCTTGAGTATCTTACCAGAGAAATACGTTCCTTTTCTGGCCTCTCAGAGCGCTGTGCAAAGCAGTCCTGTGGGCTATTACTCCTGCTCCTGAAG

BE_scaffold2403 ....................................................................................................

5110 5120 5130 5140 5150 5160 5170 5180 5190 5200

....|....|....|....|....|....|....|....|....|....|....|....|....|....|....|....|....|....|....|....|

WTE_scaffold23856 GTATACACAGGGAAGGCATGAGGTCAGTCCCTGAATCCAACTCCCTTTCCATCCCCAAAAGACCACTGGGCACAACTGGAATCAGTGGAGATTTTGTGGA

BE_scaffold2403 ....................................................................................................

5210 5220 5230 5240 5250 5260 5270 5280 5290 5300

....|....|....|....|....|....|....|....|....|....|....|....|....|....|....|....|....|....|....|....|

WTE_scaffold23856 TTATCATGGTTAGAGTTAACAGATCCTTGATTTTGACTTATGTCCTGCTTTGTGCTTGCTGCAAAACGCAGATTTAGAAGTTAATAGACAAATATTTTCT

BE_scaffold2403 ....................................................................................................

5310 5320 5330 5340 5350 5360 5370 5380 5390 5400

....|....|....|....|....|....|....|....|....|....|....|....|....|....|....|....|....|....|....|....|

WTE_scaffold23856 GTGGGATTTGCAAGATATAAAGTCACTGACACTTGAGGTGCATTCAGCAAACATTTAGAGGTCTTTATTCCAGCTGATACACAATTATGATTCTTGTTTT

BE_scaffold2403 ....................................................................................................

5410 5420 5430 5440 5450 5460 5470 5480 5490 5500

....|....|....|....|....|....|....|....|....|....|....|....|....|....|....|....|....|....|....|....|

WTE_scaffold23856 GTTTTGTTTTGTTACAGGAATGTGCACATTATTTCCTTGAAGTTAAAGACAAAGATATCAAGCATGCACTGGCAGGACTGTTTGTTGAAATTCTTGTCCC

BE_scaffold2403 ....................................................................................................

5510 5520 5530 5540 5550 5560 5570 5580 5590 5600

....|....|....|....|....|....|....|....|....|....|....|....|....|....|....|....|....|....|....|....|

WTE_scaffold23856 TGTAGCTGCTGTGAGTTTATTTTTCATAATTTTTTGTGAGGTTATTTACATAATTTCTTCAAATTGGATAAAACAACTTGTGAAATGGTGGTTTATTAAC

BE_scaffold2403 ....................................................................................................

5610 5620 5630 5640 5650 5660 5670 5680 5690 5700

....|....|....|....|....|....|....|....|....|....|....|....|....|....|....|....|....|....|....|....|

WTE_scaffold23856 ATGAAATTTTCTTTCAGGCTGTTAAAAATGAAGTGAATGTCCCCTGTCTGAGGAACTTCGTTGAAAGCCTGTATGATACAACACTTGAACTTTCCTCACG

BE_scaffold2403 ....................................................................................................

5710 5720 5730 5740 5750 5760 5770 5780 5790 5800

....|....|....|....|....|....|....|....|....|....|....|....|....|....|....|....|....|....|....|....|

WTE_scaffold23856 AAAGAAACACTCGCTGGTTAGTAACCCTAAGAAGCAGAGGCTAAAATTCTTTGTCAGAAATATCTCTGAGCAAGACTCTTGGGAAATCGCAAAACTTTTT

BE_scaffold2403 ....................................................................................................

5810 5820 5830 5840 5850 5860 5870 5880 5890 5900

....|....|....|....|....|....|....|....|....|....|....|....|....|....|....|....|....|....|....|....|

WTE_scaffold23856 TCTGTGTTTGGCTTTATAACCAATACATTTCTATTTGCAGGGAAGGTTATTGTGTTTGCAGAAAATAACCATATTTATTTTATT----------CAGAAT

BE_scaffold2403 .........................................................................NNNNNNNNNN.TTATTTTATT......

5910 5920 5930 5940 5950 5960 5970 5980 5990 6000

....|....|....|....|....|....|....|....|....|....|....|....|....|....|....|....|....|....|....|....|

WTE_scaffold23856 ATGATGTAAATCAGTCTGAATCAAACAGATGTTTTCTGCTTCTGGTTCTGTGATTATTAAATGGTTAGACTTTCTCATACTTGTTATGTGTAAACATTAG

BE_scaffold2403 ....................................................................................................

6010 6020 6030 6040 6050 6060 6070 6080 6090 6100

....|....|....|....|....|....|....|....|....|....|....|....|....|....|....|....|....|....|....|....|

WTE_scaffold23856 GAGAGGAGAAAGAATAGAGTTCACTTCTTGTCAAGCGAGAACAAAT------------------------------------------------------

BE_scaffold2403 ...................................NNNNNNNNNN.TTATTTTATTCAGAATATGATGTAAATCAGTCTGAATCAAACAGATGTTTTCTG

6110 6120 6130 6140 6150 6160 6170 6180 6190 6200

....|....|....|....|....|....|....|....|....|....|....|....|....|....|....|....|....|....|....|....|

WTE_scaffold23856 ----------------------------------------------------------------------------------------------------

BE_scaffold2403 CTTCTGATTCTGTGATTATTAAATGGTTAGACTTTCTCATACTTGTTATGTGTAAACATTAGGAGAGGAGAAAGAATAGAGTTCACTTCTTGTCAAANNN

6210 6220 6230 6240 6250 6260 6270 6280 6290 6300

....|....|....|....|....|....|....|....|....|....|....|....|....|....|....|....|....|....|....|....|

WTE_scaffold23856 ----------------------------------------------------GAGGCATGTGGGAACAACTCTTTCATGGGGCTTAAATAATACATTGTA

BE_scaffold2403 NNNNNNNNNNNNNNNNNNNNNNNNNNNNNNNNNNNNNNNNNCGAGAACAAAT................................................

6310 6320 6330 6340 6350 6360 6370 6380 6390 6400

....|....|....|....|....|....|....|....|....|....|....|....|....|....|....|....|....|....|....|....|

WTE_scaffold23856 GGAATGAAAAAATGTATAACACATGCCCTTTTAAATCCGTAACTTGGCTACTGTGCAGCTCCCTATTTCATTTTTTTCTTGTGAGATGAACAGTGGCACT

BE_scaffold2403 ....................................................................................................

6410 6420 6430 6440 6450 6460 6470 6480 6490 6500

....|....|....|....|....|....|....|....|....|....|....|....|....|....|....|....|....|....|....|....|

WTE_scaffold23856 GCAGTGTATTTTTGGAAGCATGAGTTGATCTGAATTAAATGAGGATCTGAGTCAGTTTGATGCTGGCCGCCTTAAGCAGTCTGTCTCTCCAACTGGGTCA

BE_scaffold2403 ....................................................................................................

6510 6520 6530 6540 6550 6560 6570 6580 6590 6600

....|....|....|....|....|....|....|....|....|....|....|....|....|....|....|....|....|....|....|....|

WTE_scaffold23856 GTGTACGTCAGCAATGCAGTGTTAGCTAATCCGTTTTGAAGTCTTGGACCTTCTAAGTAAAGAAAGTTGTCATTTGTCCTCCCTCGTTCTATGGGAAAGT

BE_scaffold2403 ....................................................................................................

6610 6620 6630 6640 6650 6660 6670 6680 6690 6700

....|....|....|....|....|....|....|....|....|....|....|....|....|....|....|....|....|....|....|....|

WTE_scaffold23856 CAAAAAGACTTCCCTGTTCCCAGTGCTCTCGGCAAATGGTCACTAGTGTTAGTGAATCTGCTGGGAGGACTGCAGGAGCATGCCTGAGCTCTCTGCCGCT

BE_scaffold2403 ....................................................................................................

6710 6720 6730 6740 6750 6760 6770 6780 6790 6800

....|....|....|....|....|....|....|....|....|....|....|....|....|....|....|....|....|....|....|....|

WTE_scaffold23856 GTTACACCAGTCCCAGGGGAGAGTCAGAACATCTCCTGGGGTGCAGTCCTGATCCTGTTTGAATTAGTGGAAGTCTTGAGATGGATTTCTGATTTCGCTC

BE_scaffold2403 ................................................................................................A...

6810 6820 6830 6840 6850 6860 6870 6880 6890 6900

....|....|....|....|....|....|....|....|....|....|....|....|....|....|....|....|....|....|....|....|

WTE_scaffold23856 TGCTTATGTTTGCGTGGATCTATTCCACTCTGCCAAGTTAATAAAACACTGTTGAAACTGCAGCAGGGTAAGAGCTTTGCTGTACAGTTTTTACACTAAA

BE_scaffold2403 ....................................................................................................

6910 6920 6930 6940 6950 6960 6970 6980 6990 7000

....|....|....|....|....|....|....|....|....|....|....|....|....|....|....|....|....|....|....|....|

WTE_scaffold23856 TAGAACTTCCCCAAACTGGTGGCCGTACTTGCCAAAGTCAATACTACGTGCTTGAGAAAGGGTTGCACAGCTGGGGCTTTGTGTTTGATTGCTTGTCTAC

BE_scaffold2403 ..........................................................................A.........................

7010 7020 7030 7040 7050 7060 7070 7080 7090 7100

....|....|....|....|....|....|....|....|....|....|....|....|....|....|....|....|....|....|....|....|

WTE_scaffold23856 ACATACCACACTGGAGGCTACATAGTTAAAAGTGACAAAATAGAATAGATTCTTGTGAGGGTGTGATTAGAAATACAAACATTGCGAGGAGGAGGAGGAG

BE_scaffold2403 ....................................................................................................

7110 7120 7130 7140 7150 7160 7170 7180 7190 7200

....|....|....|....|....|....|....|....|....|....|....|....|....|....|....|....|....|....|....|....|

WTE_scaffold23856 GAGGGGAAAGGCAAACAGTTGGCAGAAATAACTGCAGTGCATATTTCCAGGCTTCCAGGAAGAAAGCACACTCTTTAGAGTCTGAAGAGGACCTTCCTTT

BE_scaffold2403 ....................................................................................................

7210 7220 7230 7240 7250 7260 7270 7280 7290 7300

....|....|....|....|....|....|....|....|....|....|....|....|....|....|....|....|....|....|....|....|

WTE_scaffold23856 CCCAAGATCAGTGTTTTGTGAATCGGTGCACTCACTGTACTGGCAGGATAGGGCCATTTGTGTTGCCTCATTTTCTATCTGAAAGTATCCCGCATGAGCT

BE_scaffold2403 ....................................................................................................

7310 7320 7330 7340 7350 7360 7370 7380 7390 7400

....|....|....|....|....|....|....|....|....|....|....|....|....|....|....|....|....|....|....|....|

WTE_scaffold23856 CGTCATTGAGGTTCTCTTGATAGTGAATGGAGAGACTTATGAAACTCTAGGAGATCGTTCGTATCTTCCCTCCTAAGGCTCTTCCTTAGGATGGTTTGTA

BE_scaffold2403 ....................................................................................................

7410 7420 7430 7440 7450 7460 7470 7480 7490 7500

....|....|....|....|....|....|....|....|....|....|....|....|....|....|....|....|....|....|....|....|

WTE_scaffold23856 ACGCTCTTGGGATGTTACTAAACTCTGTGATCCTGTCAAGTACTTGACTATTATAATGTTAATATGGTAAGAAAGTCTTGCATGAGAAGACTTCACTTAT

BE_scaffold2403 ....................................................................................................

7510 7520 7530 7540 7550 7560 7570 7580 7590 7600

....|....|....|....|....|....|....|....|....|....|....|....|....|....|....|....|....|....|....|....|

WTE_scaffold23856 CCTCTTCCATATGTATTTTGGACATGAAGATTATAAAACTAAAAATTAACGTACATTTGCCATTTACTCAAGAAACTAGGTCTAACCTAGCTATAGACAA

BE_scaffold2403 ....................................................................................................

7610 7620 7630 7640 7650 7660 7670 7680 7690 7700

....|....|....|....|....|....|....|....|....|....|....|....|....|....|....|....|....|....|....|....|

WTE_scaffold23856 TTTATGTTATATATTACAGGAACAGAGGTTGGGGTTTATGGTGTATTTATACTTTGAGCTGTGACTGAAGTTAGAATTTTAGGGTTAATTTTGAGAATCA

BE_scaffold2403 ....................................................................................................

7710 7720 7730 7740 7750 7760 7770 7780 7790 7800

....|....|....|....|....|....|....|....|....|....|....|....|....|....|....|....|....|....|....|....|

WTE_scaffold23856 TTGGTCTTTAAGATAAAGTTAACTTATTTTTGACTAATAACTATGAAGTCCTGACTGGATATATGAACATGACAACTGAAACAGAAAAGTCTTGCTGAAA

BE_scaffold2403 ....................................................................................................

7810 7820 7830 7840 7850 7860 7870 7880 7890 7900

....|....|....|....|....|....|....|....|....|....|....|....|....|....|....|....|....|....|....|....|

WTE_scaffold23856 TAACAAAGTCATTTTCAAAATTAATAGGTGCATTTTAGCTTATATTTTTTTAGTGTAACATTTACAAGTGGAGTGTCAAAGTAAGTGTTGACAAAGAGAC

BE_scaffold2403 ....................................................................................................

7910 7920 7930 7940 7950 7960 7970 7980 7990 8000

....|....|....|....|....|....|....|....|....|....|....|....|....|....|....|....|....|....|....|....|

WTE_scaffold23856 CAGGTTCCAGTATTTGAACTTCTGTGTTCTGCCTTGCTTCTTTTTATTTTTCACTTTGCAAGATGTAACTACCAGCAGATCCAGGCATTGCCTGTTTTTT

BE_scaffold2403 .......................A............................................................................

8010 8020 8030 8040 8050 8060 8070 8080 8090 8100

....|....|....|....|....|....|....|....|....|....|....|....|....|....|....|....|....|....|....|....|

WTE_scaffold23856 AACACAATTCTTTAGGGTGTTTGACATGTCTCTCAGTAAATATTGTGACTGCAGATTTTGAGGAGTAATAGGCATCTGAGCATTCACAAGTCCCAACAAT

BE_scaffold2403 ....................................................................................................

8110 8120 8130 8140 8150 8160 8170 8180 8190 8200

....|....|....|....|....|....|....|....|....|....|....|....|....|....|....|....|....|....|....|....|

WTE_scaffold23856 CTCTGTGGGTCCTGCTGTTATTATAGTAAAGTTTAGTTATTCCCATGCTATCATTTCTCAGTCAACTGACTTTGAGTCTGTAAAATTGTGACCCATCTTT

BE_scaffold2403 ....................................................................................................

8210 8220 8230 8240 8250 8260 8270 8280 8290 8300

....|....|....|....|....|....|....|....|....|....|....|....|....|....|....|....|....|....|....|....|

WTE_scaffold23856 GGATTTATTATACTGCTATAAGCAGGTATGTGGTTCTTTTTAGTTCATCCATAAAACATATCTGTAACAGCTTTGGAGAAAGGTTCCTTTTACATTGTAC

BE_scaffold2403 ....................................................................................................

8310 8320 8330 8340 8350 8360 8370 8380 8390 8400

....|....|....|....|....|....|....|....|....|....|....|....|....|....|....|....|....|....|....|....|

WTE_scaffold23856 TACTTAACAATGTGTTTATGTTGGCTAGCATGTATTGCACAAAATGGAGGAGTAAGAGCTTTAAGTTTACAGTAGCTCTATTAAGTTACTTTTATGATGT

BE_scaffold2403 ....................................................................................................

8410 8420 8430 8440 8450 8460 8470 8480 8490 8500

....|....|....|....|....|....|....|....|....|....|....|....|....|....|....|....|....|....|....|....|

WTE_scaffold23856 GTAGACATGCAAATAATATGGTATCGACATCCATTTCCTGTTTAGGCTTTGTACCCTTTGGTAACGTGCCTGCTTTGTGTCAGTCAGAAGCAGTTCTTTT

BE_scaffold2403 ....................................................................................................

8510 8520 8530 8540 8550 8560 8570 8580 8590 8600

....|....|....|....|....|....|....|....|....|....|....|....|....|....|....|....|....|....|....|....|

WTE_scaffold23856 TAAACAGATGGCATATTTTCCTCAACAACTGCTTATCCAATCTCAAGGTTAGTATCTTCTGGATTTAATATTGTATTAAAACTTCAGTATGCACTATTGA

BE_scaffold2403 ....................................................................................................

8610 8620 8630 8640 8650 8660 8670 8680 8690 8700

....|....|....|....|....|....|....|....|....|....|....|....|....|....|....|....|....|....|....|....|

WTE_scaffold23856 CCTGTTGCAAATCTGTTTTATTTAAATGTGCTGGATTATTGCTATTATCTGATTCCATACATAATTTTATTCCTGACAGTACAAATTCTTTAGCTGTGCA

BE_scaffold2403 ....................................................................................................

8710 8720 8730 8740 8750 8760 8770 8780 8790 8800

....|....|....|....|....|....|....|....|....|....|....|....|....|....|....|....|....|....|....|....|

WTE_scaffold23856 GATTTTTTCAGTAACAAAAAAGTATTCTTGTGCCTAGTTTGAATTTCTACTGTTTCTCTAACTCAAACCAAGCCTGAGTCAAACAGCTGTGAAACAGGCA

BE_scaffold2403 ......................................G..................................T..........................

8810 8820 8830 8840 8850 8860 8870 8880 8890 8900

....|....|....|....|....|....|....|....|....|....|....|....|....|....|....|....|....|....|....|....|

WTE_scaffold23856 AGATTGTTGATGAAAGTTATTAGCTTGAGTGACATACCAAAACTGACCCACTATTAAGTTGCTTTTTTTCACAAAGGCATTCTCAAACAAAGCATTTGCT

BE_scaffold2403 ...................C................................................................................

8910 8920 8930 8940 8950 8960 8970 8980 8990 9000

....|....|....|....|....|....|....|....|....|....|....|....|....|....|....|....|....|....|....|....|

WTE_scaffold23856 ATTACTTTATCAGAATGGAACTAGTTGCAGGACACTGATTCCGTTGCCTGTTTCACCCTGTTAAGTTGCATTAAAAAAAACTAAACTATTTGAAGTTGTT

BE_scaffold2403 ....................................................................................................

9010 9020 9030 9040 9050 9060 9070 9080 9090 9100

....|....|....|....|....|....|....|....|....|....|....|....|....|....|....|....|....|....|....|....|

WTE_scaffold23856 TCCTTCACATATCCATTCTTACTCCTTTACCATTTGTTAGATCTTTGAAGTTTAAACCTAACTGTCCACTGCGATCTGCTGTGGTTCCTGGCCAGCACAT

BE_scaffold2403 ....................................................................................................

9110 9120 9130 9140 9150 9160 9170 9180 9190 9200

....|....|....|....|....|....|....|....|....|....|....|....|....|....|....|....|....|....|....|....|

WTE_scaffold23856 TCTGTTCAATATACATATGCTAGTCATCGTTACAACTCCTCCCTGTTTCTTTTACTTGTGCAATAACTCACGTCCCATTCCACACTTAAGTGCATCCATA

BE_scaffold2403 ....................................................................................................

9210 9220 9230 9240 9250 9260 9270 9280 9290 9300

....|....|....|....|....|....|....|....|....|....|....|....|....|....|....|....|....|....|....|....|

WTE_scaffold23856 TGAAAGGATGGTCCTGTGCCTACCTGAAAAGGCCAAATTAAGAAGCACAGTGGTTAGCTCAAATGAAGGGCCTCTGAACTGCTGCTTGATGAACCAGTGG

BE_scaffold2403 ....................................................................................................

9310 9320 9330 9340 9350 9360 9370 9380 9390 9400

....|....|....|....|....|....|....|....|....|....|....|....|....|....|....|....|....|....|....|....|

WTE_scaffold23856 TTAGGAGGCTTCTGCAAATGGCACGGGTAGAATATGCAACCATTTTCTCAGCTTAGTGGCAAACTGAGGAGGGAATCTCTGTTCTCCAAAGGCTGAATTC

BE_scaffold2403 ........................A...........................................................................

9410 9420 9430 9440 9450 9460 9470 9480 9490 9500

....|....|....|....|....|....|....|....|....|....|....|....|....|....|....|....|....|....|....|....|

WTE_scaffold23856 CCAAGCTTCTCGACAAGGTATATATTACATCCTTTATTGTTGTTGCTTTCTTGCTGTACTTTGTCTTAATGAAGCAATTTGTATTGACTCTCTTCATATG

BE_scaffold2403 ....................................................................................................

9510 9520 9530 9540 9550 9560 9570 9580 9590 9600

....|....|....|....|....|....|....|....|....|....|....|....|....|....|....|....|....|....|....|....|

WTE_scaffold23856 GATCCTACTATTAGAATAGCTCTTCAAAGTGTTCATAGTCTGTCTGAAGATTTCTTTCCACCTGTGTCTGAATTATTTACTTCTGCTGAAGATGTGTTAG

BE_scaffold2403 ....................................................................................................

9610 9620 9630 9640 9650 9660 9670 9680 9690 9700

....|....|....|....|....|....|....|....|....|....|....|....|....|....|....|....|....|....|....|....|

WTE_scaffold23856 AACCTTATTTGCAAAGAGATTCAGGAAATAAACATGTTGTTATTGCAACTAATTTTACGGGTTTTTATGAGGTTATACAAGCTTTAGAAAAGACAGCTAC

BE_scaffold2403 ....................................................................................................

9710 9720 9730 9740 9750 9760 9770 9780 9790 9800

....|....|....|....|....|....|....|....|....|....|....|....|....|....|....|....|....|....|....|....|

WTE_scaffold23856 AGCTCTTAAGATCTCTGAGGAGCCACCTGGGAATATAGATTTTTCTGAAAATTAGGGAGATCCTGACAAATTATTTTAAACCATTAGAGAAAATATAAGT

BE_scaffold2403 ....................................................................................................

9810 9820 9830 9840 9850 9860 9870 9880 9890 9900

....|....|....|....|....|....|....|....|....|....|....|....|....|....|....|....|....|....|....|....|

WTE_scaffold23856 AAAGTTGCTCCTACCTTACCAATTGAAAATCAAATTGGAATTCACCTTGCCACCTATAATTCATTGACATACGCTTTGATTCATGGGGCTCATAGATTAA

BE_scaffold2403 ....................................................................................................

9910 9920 9930 9940 9950 9960 9970 9980 9990 10000

....|....|....|....|....|....|....|....|....|....|....|....|....|....|....|....|....|....|....|....|

WTE_scaffold23856 TGTGGTGGCACATTCAATCTTTAATCTGGGGAGAAGGTAAGGTAGCTGAATACGTAGCTAAATTAAGAATGTGGTGGGCCACTCCTCAGTACAGAGGAAG

BE_scaffold2403 .....................................................A..............................................

10010 10020 10030 10040 10050 10060 10070 10080 10090 10100

....|....|....|....|....|....|....|....|....|....|....|....|....|....|....|....|....|....|....|....|

WTE_scaffold23856 AGATGCCCCAACCATTGAAGCAATCACTTCATCAGTCAGAGTGGCACCTCAAAGCCCAAACCAAACTCACACAGCTGGAAGAAGAGCTAGATCAACAAAG

BE_scaffold2403 ....................................................................................................

10110 10120 10130 10140 10150 10160 10170 10180 10190 10200

....|....|....|....|....|....|....|....|....|....|....|....|....|....|....|....|....|....|....|....|

WTE_scaffold23856 GAGGAGAAATCCTTCCATTAGAAGGGGAGTTGTGCACACCAGGACAGTATATGGGTCTCCACGATCTTGTGTGGGCTCCCATGAACCACATAATGGATCA

BE_scaffold2403 ....................................................................................................

10210 10220 10230 10240 10250 10260 10270 10280 10290 10300

....|....|....|....|....|....|....|....|....|....|....|....|....|....|....|....|....|....|....|....|

WTE_scaffold23856 GGTTGCTCAAGAGCCTCCACACCCAATAGAGCACAATCTCCTTCCCAAAACCGTTAAAGTCAAGCATACACTGGGAAAGCTAAAAGGTTTATATTCTAAT

BE_scaffold2403 ....................................................................................................

10310 10320 10330 10340 10350 10360 10370 10380 10390 10400

....|....|....|....|....|....|....|....|....|....|....|....|....|....|....|....|....|....|....|....|

WTE_scaffold23856 CATATTTCTGATTTTAATATAAACTGGCAAATCTCTGATCTTACAGAAACAAACTTTTCTCTTGAATTAATAAAGGAGACTCCTGCCAGGACATGGAAAT

BE_scaffold2403 ....................................................................................................

10410 10420 10430 10440 10450 10460 10470 10480 10490 10500

....|....|....|....|....|....|....|....|....|....|....|....|....|....|....|....|....|....|....|....|

WTE_scaffold23856 ATTTGACCCTGGCCAAACTCTGGCCAAAGGGTATTTCATACAAACCTGTTAAAAGTGGTGTTAAAGTTAATGTGTATCCTGGTTTTAAAATGGAACATTT

BE_scaffold2403 ....................................................................................................

10510 10520 10530 10540 10550 10560 10570 10580 10590 10600

....|....|....|....|....|....|....|....|....|....|....|....|....|....|....|....|....|....|....|....|

WTE_scaffold23856 ATTGTTAACTAAACTTTATTTACAAAAAAATATTCAAGGCAGGAATTCTATATAGGAGAGAGTCAAAGCACCGTGTTTCATTTAGAGGCAAAGTTTTTCA

BE_scaffold2403 ....................................................................................................

10610 10620 10630 10640 10650 10660 10670 10680 10690 10700

....|....|....|....|....|....|....|....|....|....|....|....|....|....|....|....|....|....|....|....|

WTE_scaffold23856 GTGGGAGCAACAGTTCCTTGTCTCCAAACCACATTGGACAAGGAGAAAGCTCCAGTACAATTTGAGCCGAGGTTGCAAAACTTACTTTACAAAGAATGGG

BE_scaffold2403 ....................................................................................................

10710 10720 10730 10740 10750 10760 10770 10780 10790 10800

....|....|....|....|....|....|....|....|....|....|....|....|....|....|....|....|....|....|....|....|

WTE_scaffold23856 CCAAACAATGCCTATCTCGAGAATGAATCCATGGGTACCAACGCAATATCCCTTTGGCCTGAATGGGCCAGACCTAACGCCATATTATTGGAAAACAGAA

BE_scaffold2403 ..............................................................................A.....................

10810 10820 10830 10840 10850 10860 10870 10880 10890 10900

....|....|....|....|....|....|....|....|....|....|....|....|....|....|....|....|....|....|....|....|

WTE_scaffold23856 ATGGAGGAATTAAAGAAAATGGGCCTCACTCCTGGCAGTCCGGTTGCTCCTCCGCTAATCACAGAAGAAGAGGCAGAGGCACTGAAAAAGAAAATTCTAG

BE_scaffold2403 ....................................................................................................

10910 10920 10930 10940 10950 10960 10970 10980 10990 11000

....|....|....|....|....|....|....|....|....|....|....|....|....|....|....|....|....|....|....|....|

WTE_scaffold23856 AAAAACAACAACAATGGGTAAAAGGCAGCAGCACTTATCAATCAAGAAACA--------------------------GCAGATCCTGTCAGAACTGTCAC

BE_scaffold2403 .........................NNNNNNNNNNNNNNNNNNNNNNNNNNCAGCAGCACTTATCAATCAAGAAACA.......................

11010 11020 11030 11040 11050 11060 11070 11080 11090 11100

....|....|....|....|....|....|....|....|....|....|....|....|....|....|....|....|....|....|....|....|

WTE_scaffold23856 CAACTGCTCCCGTGTTCAAAAGGCCCACATTCACTTTGACAAATTCTTCGGCACCAACAGCAAGTTGGGCGCTGCCAGCAGTG-----------------

BE_scaffold2403 .........................................................................NNNNNNNNNNCAGCAGCACTTATCAAT

11110 11120 11130 11140 11150 11160 11170 11180 11190 11200

....|....|....|....|....|....|....|....|....|....|....|....|....|....|....|....|....|....|....|....|

WTE_scaffold23856 ----------------------------------------------------------------------------------------------------

BE_scaffold2403 CAAGAAACAGCAGATCCTGTCAGAACTGTCACCAACTGCTCCCGCGTTCAAAAGGCCCACATTCACTTTGACAAATTCTTCGGCACCAACAGCAAGTTGG

11210 11220 11230 11240 11250 11260 11270 11280 11290 11300

....|....|....|....|....|....|....|....|....|....|....|....|....|....|....|....|....|....|....|....|

WTE_scaffold23856 -----------------------------------------------GTAAAGGAACCACATCCAATGCCAATACCTCCTGTTGGGAAAGTGAGGAGGAT

BE_scaffold2403 GCGCCNNNNNNNNNNNNNNNNNNNNNNNNNNNNNNNNGCCAGCAGTG.....................................................

11310 11320 11330 11340 11350 11360 11370 11380 11390 11400

....|....|....|....|....|....|....|....|....|....|....|....|....|....|....|....|....|....|....|....|

WTE_scaffold23856 GTCAGATATATATTAGGCCAAATACTCCTTGTCCTAATAGGATTACTGACGGGGTTTTTGTTGTTGACAAAAGTCCTCAAAATACTAAAGAGGCTCTATT

BE_scaffold2403 ....................................................................................................

11410 11420 11430 11440 11450 11460 11470 11480 11490 11500

....|....|....|....|....|....|....|....|....|....|....|....|....|....|....|....|....|....|....|....|

WTE_scaffold23856 GGTGATGGACTTCTCTCAGTTTTCCAAAGGAAGCCACGCTATGTGCTTTCCAAATACTGGGCACCCAGTCACCATGCACTTGCACAGACCTTGCCCATGG

BE_scaffold2403 ....................................................................................................

11510 11520 11530 11540 11550 11560 11570 11580 11590 11600

....|....|....|....|....|....|....|....|....|....|....|....|....|....|....|....|....|....|....|....|

WTE_scaffold23856 ACATGCCCATGATTTCCGTGGATGTTTCTCAAGCTTTTTATCATCTTCTTCTCAATCCTGCTTCTGCTATACAGCTTGTTGTTTCTGACGGAAAAGTGGT

BE_scaffold2403 ....................................................................................................

11610 11620 11630 11640 11650 11660 11670 11680 11690 11700

....|....|....|....|....|....|....|....|....|....|....|....|....|....|....|....|....|....|....|....|

WTE_scaffold23856 CTACTATTTTCAGAAAACTCCAATGATATCGGTCTCAGCCCCTTTCTCCTCCGTCTCTTCTCAACTGCCCTCGCAGCTGGACTATCTTGTCGCTAGAATA

BE_scaffold2403 ....................................................................................................

11710 11720 11730 11740 11750 11760 11770 11780 11790 11800

....|....|....|....|....|....|....|....|....|....|....|....|....|....|....|....|....|....|....|....|

WTE_scaffold23856 TTTGGACTTTTGCTTATAGGGATGACTTCCTCCTTTGCCACACAAGCTCTTATTACCTTAACTCCATTAGCCAGTGTCTGCTGTTTTCTTGAAAGCTTTG

BE_scaffold2403 ....................................................................................................

11810 11820 11830 11840 11850 11860 11870 11880 11890 11900

....|....|....|....|....|....|....|....|....|....|....|....|....|....|....|....|....|....|....|....|

WTE_scaffold23856 GGGTAAGAATAAATTTTGATTAACATACTCCATCACCTGTTGAAGAAATCAAATTCTTAGGACTGAAATTTACAAGGACTGACATGATGATACCTGGTGA

BE_scaffold2403 ....................................................................................................

11910 11920 11930 11940 11950 11960 11970 11980 11990 12000

....|....|....|....|....|....|....|....|....|....|....|....|....|....|....|....|....|....|....|....|

WTE_scaffold23856 AAAATAGATTGAAAGTAAACAGGTTATTAAACAAATTGATTGTAACCAATGTTATGATGTTAAAATGCTGCAAAGATTAATAGGCTGTGTAAACTTTGGC

BE_scaffold2403 ....................................................................................................

12010 12020 12030 12040 12050 12060 12070 12080 12090 12100

....|....|....|....|....|....|....|....|....|....|....|....|....|....|....|....|....|....|....|....|

WTE_scaffold23856 ATTCCTTTTACTACTTATTCTAATCATGTGCTACAACCATTGCATGCTGCTGTAATAAACAAAAAGGATTCTCCATTTTCTGTTCCATATAAAGGCTTAT

BE_scaffold2403 ....................................................................................................

12110 12120 12130 12140 12150 12160 12170 12180 12190 12200

....|....|....|....|....|....|....|....|....|....|....|....|....|....|....|....|....|....|....|....|

WTE_scaffold23856 TGTATAAAATGTGTTGGCAAGGAGTTAAATGGAAATTGCAACCCAAGGACAGTGTTTCTATTCCTAAGCTTGTGACAGATGCCATGCTGGAAGTTGGAGC

BE_scaffold2403 ....................................................................................................

12210 12220 12230 12240 12250 12260 12270 12280 12290 12300

....|....|....|....|....|....|....|....|....|....|....|....|....|....|....|....|....|....|....|....|

WTE_scaffold23856 CATATCCCATATCATTGGTGGGTTGTCTTGCTTCCATTGTGCAGGACCTAGACCAATACACATTCAAGAATTAATGATGGCATTGGTTGCAGTTTCTTTG

BE_scaffold2403 ....................................................................................................

12310 12320 12330 12340 12350 12360 12370 12380 12390 12400

....|....|....|....|....|....|....|....|....|....|....|....|....|....|....|....|....|....|....|....|

WTE_scaffold23856 ATCAAACCGTGGTCTTTGATCTGTGACTCAACATTCGTCTGCAGACAAAAATTCAGCTCTTTGCCCTGGAGATTTGCACTGTGAGCCAGGCAAATCTTAT

BE_scaffold2403 ....................................................................................................

12410 12420 12430 12440 12450 12460 12470 12480 12490 12500

....|....|....|....|....|....|....|....|....|....|....|....|....|....|....|....|....|....|....|....|

WTE_scaffold23856 CTCGGGTCAAAGTGTACTGGAATCCTTCAAAGTTTAACCCAACGGATGGCCCAACACGTGGGAGACTATCTGATTGGACTGCATATACTTGCACACTCCT

BE_scaffold2403 ....................................................................................................

12510 12520 12530 12540 12550 12560 12570 12580 12590 12600

....|....|....|....|....|....|....|....|....|....|....|....|....|....|....|....|....|....|....|....|

WTE_scaffold23856 CAGAAAGCCCTTAAAGCTGCCACGGAAACAAAAACCCACATATGACCAAAGAGACCAAAAGACAATCTTGTACTAGTTCACAAGTTTCTGAAAATAACAC

BE_scaffold2403 ....................................................................................................

12610 12620 12630 12640 12650 12660 12670 12680 12690 12700

....|....|....|....|....|....|....|....|....|....|....|....|....|....|....|....|....|....|....|....|

WTE_scaffold23856 CAATCCAGACAGATAATCCACTTCAGTCCCGCTGATCCCAGATGCCATGTTAATTAGGCCAGCCTAAGCATCCACAGATTTTTGTCCTTTCACTTTTGAA

BE_scaffold2403 ....................................................................................................

12710 12720 12730 12740 12750 12760 12770 12780 12790 12800

....|....|....|....|....|....|....|....|....|....|....|....|....|....|....|....|....|....|....|....|

WTE_scaffold23856 TATCATTTATTTATCTTCTGTGTTCTTCATACCAGGTCTCCACCATAGGGCAACAGCTTTTGAATTGACAGAATCCACTGAAGATGAGATTTTCTTTTCA

BE_scaffold2403 ..................................................................................................A.

12810 12820 12830 12840 12850 12860 12870 12880 12890 12900

....|....|....|....|....|....|....|....|....|....|....|....|....|....|....|....|....|....|....|....|

WTE_scaffold23856 GAGTCAAATTGAGGTGGTTTTCAGATAGAGATTTTAAATTGTAACATGTATCTAATGTAACTGACTTGGGTTTTTCGACAACTTAAGTAATACCAGGAGA

BE_scaffold2403 ....................................................................................................

12910 12920 12930 12940 12950 12960 12970 12980 12990 13000

....|....|....|....|....|....|....|....|....|....|....|....|....|....|....|....|....|....|....|....|

WTE_scaffold23856 GGTAGGTCAGATGCATAAGTGTCCTACAGCTACAGGAATGGGGTACTGTGCAGCCGTGAGGCTGTATATTCTCTGAACCTTGACATCCATGAAGTGCAGA

BE_scaffold2403 ....................................................................................................

13010 13020 13030 13040 13050 13060 13070 13080 13090 13100

....|....|....|....|....|....|....|....|....|....|....|....|....|....|....|....|....|....|....|....|

WTE_scaffold23856 ACTTCCTTTCCTTTCTGATTTGTGCCTGAAGTTTATTCTTACATTATACATTTGTCTAGAATAAGGACCCAAAAATGGCTCGAGTTGCACTGGAATCTCT

BE_scaffold2403 ....................................................................................................

13110 13120 13130 13140 13150 13160 13170 13180 13190 13200

....|....|....|....|....|....|....|....|....|....|....|....|....|....|....|....|....|....|....|....|

WTE_scaffold23856 GTACAGACTACTGTGGGTTTACATGATCAGAATTAAATGTGAAAGCAACACGGCTACCCAAAGGTAAGATGCTGTATTTGTAATGATAATCAGGCCTTGT

BE_scaffold2403 ....................................................................................................

13210 13220 13230 13240 13250 13260 13270 13280 13290 13300

....|....|....|....|....|....|....|....|....|....|....|....|....|....|....|....|....|....|....|....|

WTE_scaffold23856 AGAGTGGATGTTGCCAGTGAAGAAGGAGCTTTTATAGTTCTCCTGAATTCCTTTGANNNNNNNNNNNNNNNNNNNNNCTGTGAAATAAAAACTCAACTGT

BE_scaffold2403 ........................A..................................................-----....................

13310 13320 13330 13340 13350 13360 13370 13380 13390 13400

....|....|....|....|....|....|....|....|....|....|....|....|....|....|....|....|....|....|....|....|

WTE_scaffold23856 ACACAATAAAATATTCTCTTTTGTGGCACCATCAGTTATTCTCCCACCTATTAAAATTACTGTATAAAGTGTTTCTGCTTTTGGAAGAACTAGAAATCAC

BE_scaffold2403 ....................................................................................................

13410 13420 13430 13440 13450 13460 13470 13480 13490 13500

....|....|....|....|....|....|....|....|....|....|....|....|....|....|....|....|....|....|....|....|

WTE_scaffold23856 TNNNNNNNNNNNNNNNNNNNNNNNNNNNNNGAAATATTTTTGTAGTGGCATGTCATTGACTGGTTTATTGTTACTTGTAATTTAGTAATGAAATAAAATA

BE_scaffold2403 .......................----------...................................................................

13510 13520 13530 13540 13550 13560 13570 13580 13590 13600

....|....|....|....|....|....|....|....|....|....|....|....|....|....|....|....|....|....|....|....|

WTE_scaffold23856 ATTACATTGAATTTGTAGCAAAGGATCAGAACAAGATACCCCATTACATTTTCATTTCAAAGGAAATACATTCTAGTCTCTCTAAATGGAGGAGGATTTC

BE_scaffold2403 ....................................................................................................

13610 13620 13630 13640 13650 13660 13670 13680 13690 13700

....|....|....|....|....|....|....|....|....|....|....|....|....|....|....|....|....|....|....|....|

WTE_scaffold23856 CAACTTACGTTTCATGGTAAGTGAAGGTCTGCAAGGTCCAGAAATGTCTAATGCTTTTCTTCCTTCTTTTACAGTCGGCTTATTACTATTGTCACAACAC

BE_scaffold2403 .......T............................................................................................

13710 13720 13730 13740 13750 13760 13770 13780 13790 13800

....|....|....|....|....|....|....|....|....|....|....|....|....|....|....|....|....|....|....|....|

WTE_scaffold23856 TTTTCCCAAAAGGGTCCCGTGGTGTTGTGCCAAGAGATATGCCTCTAAACATCTTTGTGAAGATAATACAGTTTATTGCACAGGTATGGGAGAACCATGC

BE_scaffold2403 ....................................................................................................

13810 13820 13830 13840 13850 13860 13870 13880 13890 13900

....|....|....|....|....|....|....|....|....|....|....|....|....|....|....|....|....|....|....|....|

WTE_scaffold23856 ATATGTCTTTCCAATTAATGAATAACTTGTTTTCCCAATAACAAATTAACAAAGAAGTAAACTTCTATTGTCTGAATTTTTTGCAACCCCATTGTCAGTA

BE_scaffold2403 ....................................................................................................

13910 13920 13930 13940 13950 13960 13970 13980 13990 14000

....|....|....|....|....|....|....|....|....|....|....|....|....|....|....|....|....|....|....|....|

WTE_scaffold23856 TCTTTTCATCTAGGCAATGATGCAATTATAGATTGAGCTGGGGCAGACTTCTTTTGAACAACAGGTTTTGATTAATTTAATTCCACTGAAAATGGATAAA

BE_scaffold2403 ....................................................................................................

14010 14020 14030 14040 14050 14060 14070 14080 14090 14100

....|....|....|....|....|....|....|....|....|....|....|....|....|....|....|....|....|....|....|....|

WTE_scaffold23856 AATGTTTTCACTTCAACTCAAATGTATTTTTTGTCAGTTTTTGGAATTGCCAGCAAGTTAGAAAATGCATTTGTTGACAGGCTCTGACTGCCAATAAAGA

BE_scaffold2403 ....................................................................................................

14110 14120 14130 14140 14150 14160 14170 14180 14190 14200

....|....|....|....|....|....|....|....|....|....|....|....|....|....|....|....|....|....|....|....|

WTE_scaffold23856 TCAAAATAATAAGCCATATTTTGTCCTGATACTGAATCATTCCTACTATATAGGACAATGAGTTGCATTGCTTTTAAAGCAATAAGTCAGAGGAAAACCA

BE_scaffold2403 ....................................................................................................

14210 14220 14230 14240 14250 14260 14270 14280 14290 14300

....|....|....|....|....|....|....|....|....|....|....|....|....|....|....|....|....|....|....|....|

WTE_scaffold23856 AGTTCAGCATGTAGCTGACACATTTAGTTTTCATCAGTACAAATGGGAATTGCTAAAACAAACTAACCTGCAGTGCTGGAAGTTTCATCTCTTTTATTAC

BE_scaffold2403 ....................................................................................................

14310 14320 14330 14340 14350 14360 14370 14380 14390 14400

....|....|....|....|....|....|....|....|....|....|....|....|....|....|....|....|....|....|....|....|

WTE_scaffold23856 TTAAATTCAGAAAACATTCCCATTGAGCAAAGCACTTAAGCACGTGCTTAAAGCCTGTGAATAGGAATGAATTAAACAGATGCTTAAAATCTTCCCTGAA

BE_scaffold2403 ....................................................................................................

14410 14420 14430 14440 14450 14460 14470 14480 14490 14500

....|....|....|....|....|....|....|....|....|....|....|....|....|....|....|....|....|....|....|....|

WTE_scaffold23856 CTGTGCCATCTGTGAACAAGCTCCTTTTATGTAAAATACTATGTTACTACCATAATTACCACAGTTTAAATGAAATAATTAAAAGCTTTATTACTAAAAT

BE_scaffold2403 ....................................................................................................

14510 14520 14530 14540 14550 14560 14570 14580 14590 14600

....|....|....|....|....|....|....|....|....|....|....|....|....|....|....|....|....|....|....|....|

WTE_scaffold23856 GGATCATTTTCTATTTAAATGCTATAAATTTTTGAATTTGTCAACATACAGCAGTGTTGAAAGCTTAGATCTGATAATACGTTCTGCATACTTCTGAAAA

BE_scaffold2403 ...............................................................................T....................

14610 14620 14630 14640 14650 14660 14670 14680 14690 14700

....|....|....|....|....|....|....|....|....|....|....|....|....|....|....|....|....|....|....|....|

WTE_scaffold23856 TGTTTTTCCGAACATTTAAATAAATCCTGATTAAACTGGTTTTCAGCATTCTATTGCCTTAAAGAAAATAAGTAGCAGAAGTGAGCAGCTTCATGATTTG

BE_scaffold2403 ....................................................................................................

14710 14720 14730 14740 14750 14760 14770 14780 14790 14800

....|....|....|....|....|....|....|....|....|....|....|....|....|....|....|....|....|....|....|....|

WTE_scaffold23856 CAAAATTTTCACTATTTTAGGAACGTTTAGATTTTGCAATGAAAGAAATTATCTTTGACTTCCTTTGTGTTGGAAAGCCAGCGAAAGCTTTCAGTCTCAA

BE_scaffold2403 ....................................................................................................

14810 14820 14830 14840 14850 14860 14870 14880 14890 14900

....|....|....|....|....|....|....|....|....|....|....|....|....|....|....|....|....|....|....|....|

WTE_scaffold23856 CCCTGAGGTATGTTGTGCATCTCCATTGTTTTAAGTTAGTATATATTGTATTGTGCTACATTTACTTTCTGGTTACATTCTAAGCGTGCTTTCACCTTTT

BE_scaffold2403 ....................................................................................................

14910 14920 14930 14940 14950 14960 14970 14980 14990 15000

....|....|....|....|....|....|....|....|....|....|....|....|....|....|....|....|....|....|....|....|

WTE_scaffold23856 TCCAGTTTTCAGGCCAAATCTTGAACTTGATTTATTGGTAAAATACATCTGTGGACTTTGGTCTGTGAAATGTCACATTGTTTATGCCTACCTCATATTT

BE_scaffold2403 ....................................................................................................

15010 15020 15030 15040 15050 15060 15070 15080 15090 15100

....|....|....|....|....|....|....|....|....|....|....|....|....|....|....|....|....|....|....|....|

WTE_scaffold23856 CCTCAGTGAGGACTATCAAATGTAAAGTAGTGCAAATGTATAAAAGAAAAAGATGAATAGAACTATTGATCACAATCTCTCCTTATCACTCTGCTGTTGT

BE_scaffold2403 ............G.......................................................................................

15110 15120 15130 15140 15150 15160 15170 15180 15190 15200

....|....|....|....|....|....|....|....|....|....|....|....|....|....|....|....|....|....|....|....|

WTE_scaffold23856 TATATGCAATGCCTTAAATTATTTTGATATAAAAATAAGTTTTTGTAACAATTTCAGTGTTTTTCAAAAGAGTTTTACTGAAAAAGAGTACATGATCCAA

BE_scaffold2403 ..............................................................................................G.....

15210 15220 15230 15240 15250 15260 15270 15280 15290 15300

....|....|....|....|....|....|....|....|....|....|....|....|....|....|....|....|....|....|....|....|

WTE_scaffold23856 GAAGAATTGAAGTTGACAAAAAAGAAATAACTCTAATATTAAAGTTTTACTGCTCTGACCAGTAAAGCTCCAGTAGTGTTAGGGTCATGAGTCAGCTTCC

BE_scaffold2403 ....................................................................................................

15310 15320 15330 15340 15350 15360 15370 15380 15390 15400

....|....|....|....|....|....|....|....|....|....|....|....|....|....|....|....|....|....|....|....|

WTE_scaffold23856 TAGTGCTTTCCCTAGAGAAAACAATCAGAAATTTGAAAAACGTTAAAAAAAAAAAAAAAAAAAGTGGTAAAATAATGAAAAATTCTCTTGAAAATAATGT

BE_scaffold2403 ..............................................................-.....................................

15410 15420 15430 15440 15450 15460 15470 15480 15490 15500

....|....|....|....|....|....|....|....|....|....|....|....|....|....|....|....|....|....|....|....|

WTE_scaffold23856 ATTTGCTTATAATTTTGAAAACATTTTCATTTGAAAATTAAGAATGAACACAAGAAAAATTAAAAGCATTTGTGATCATCTCATAAAATATGTCTGTATC

BE_scaffold2403 ....................................................................................................

15510 15520 15530 15540 15550 15560 15570 15580 15590 15600

....|....|....|....|....|....|....|....|....|....|....|....|....|....|....|....|....|....|....|....|

WTE_scaffold23856 AATCTTGCAGTATTAAACAGTCATAGCTATTGACTAAGATGTTAAAATAGAATGCGTTACTTTACAAAAACTATATTTGATTTTAAAAAAATTTGGAAAA

BE_scaffold2403 ....................................................................................................

15610 15620 15630 15640 15650 15660 15670 15680 15690 15700

....|....|....|....|....|....|....|....|....|....|....|....|....|....|....|....|....|....|....|....|

WTE_scaffold23856 GTAGATTAAACTTTTTACTGTCAAAAGCAGCAGAAGTGAAAGGCAAGCTTTACCTTGAGCACAGCTTTCATCTTTCTTCAGCAGAGTAAAGAAAAATGAA

BE_scaffold2403 ....................................................................................................

15710 15720 15730 15740 15750 15760 15770 15780 15790 15800

....|....|....|....|....|....|....|....|....|....|....|....|....|....|....|....|....|....|....|....|

WTE_scaffold23856 ACAGAATTCTTGAGCAGGCAGTCCCCACCACGTTCCGGGTCAGTCACCCGCAGTATTGACTGAGGAAGGTCCTGCAAAGGACGTTGTGTATTAGCATTGC

BE_scaffold2403 ....................................................................................................

15810 15820 15830 15840 15850 15860 15870 15880 15890 15900

....|....|....|....|....|....|....|....|....|....|....|....|....|....|....|....|....|....|....|....|

WTE_scaffold23856 ATGAAGCTGAGCTCATGGGGAGTGGACGTTGTCCTTAGCTTCTTCATGAATCAACATCTGAAATTTTACTGTTTCACAACAATGTAAATCCGAAAACAGT

BE_scaffold2403 ....................................................................................................

15910 15920 15930 15940 15950 15960 15970 15980 15990 16000

....|....|....|....|....|....|....|....|....|....|....|....|....|....|....|....|....|....|....|....|

WTE_scaffold23856 GAAATAACAGGGCTACTTTTTGGCGTACATAGACTGTAGCATTCTAAAAAAAATACCCATCATCTATGTAAATGCACCTCTGGGAGACTGTCAGTAGGAC

BE_scaffold2403 ....................................................................................................

16010 16020 16030 16040 16050 16060 16070 16080 16090 16100

....|....|....|....|....|....|....|....|....|....|....|....|....|....|....|....|....|....|....|....|

WTE_scaffold23856 AGCCTCTGAGTTTTTGGAATACATTCCTGGGAAATCAAAACATTCTGGTCTTTTATTAATTGTTGCAATGCAAAGCTTGCAAGTTTTCTGCTGGTGAATT

BE_scaffold2403 ....................................................................................................

16110 16120 16130 16140 16150 16160 16170 16180 16190 16200

....|....|....|....|....|....|....|....|....|....|....|....|....|....|....|....|....|....|....|....|

WTE_scaffold23856 CCCAGAAAGCATTCAGGTCACTTAGACTCACGTTCCTCATGTTTTCACATTGCAGTTTTGGCGCTGAGCACCATTCCAGACTTTTCACTCCATATGGTCA

BE_scaffold2403 ................................................G...................................................

16210 16220 16230 16240 16250 16260 16270 16280 16290 16300

....|....|....|....|....|....|....|....|....|....|....|....|....|....|....|....|....|....|....|....|

WTE_scaffold23856 GCCCTGGGGCCTTTTGAGACAGTTATTGATCTTTTTTTGTAATGGACCACATGGGACATTTCTGTTTAACTGAAGGTCCAGTTCAGGTGCATTACTAAAC

BE_scaffold2403 ....................................................................................................

16310 16320 16330 16340 16350 16360 16370 16380 16390 16400

....|....|....|....|....|....|....|....|....|....|....|....|....|....|....|....|....|....|....|....|

WTE_scaffold23856 ACCAGGTCATATCCACGTGTTTTATCCTGAAGATAAAAAGATAATGTACTAATGAAAATAGTTAGAAATAAAGTTTTGAGAGGGGAGAGCACTGCTCACA

BE_scaffold2403 ....................................................................................................

16410 16420 16430 16440 16450 16460 16470 16480 16490 16500

....|....|....|....|....|....|....|....|....|....|....|....|....|....|....|....|....|....|....|....|

WTE_scaffold23856 GTGCAGATATGAATTCACTATTTCATGGGCATTTTTCGTAAAATACATCAAACTAGTAAATGTAGCGAAGGATATGGGTAGAATATGTTTATTTGATTAT

BE_scaffold2403 ....................................................................................................

16510 16520 16530 16540 16550 16560 16570 16580 16590 16600

....|....|....|....|....|....|....|....|....|....|....|....|....|....|....|....|....|....|....|....|

WTE_scaffold23856 GATACTCATTCATCTTCTGAATTTTACAGAGAATGAATATTGGTCTGAGAGCTTTCTTGGTAATTGCTGATAGCTTGCAGCAAAAAGATGGTGAACCTCC

BE_scaffold2403 ....................................................................................................

16610 16620 16630 16640 16650 16660 16670 16680 16690 16700

....|....|....|....|....|....|....|....|....|....|....|....|....|....|....|....|....|....|....|....|

WTE_scaffold23856 AATGCCAGTGACGGGAGCTGTCCTTCCCTCTGGAAACACTCTCAGAGTGAAGAAAACCTATTTGAGCAAAACTCTTACAGAGGAGGAAGCTAAAATGATA

BE_scaffold2403 ....................................................................................................

16710 16720 16730 16740 16750 16760 16770 16780 16790 16800

....|....|....|....|....|....|....|....|....|....|....|....|....|....|....|....|....|....|....|....|

WTE_scaffold23856 GGTCAGTAAGAGAATGGACAATATAGAAACTTAGATGAGAATGAATGTGAAAAAGTCTTCAATACCTGAGCAGAATAATTTACTGTTGTTAAGTATTCAA

BE_scaffold2403 ....................................................................................................

16810 16820 16830 16840 16850 16860 16870 16880 16890 16900

....|....|....|....|....|....|....|....|....|....|....|....|....|....|....|....|....|....|....|....|

WTE_scaffold23856 TTGATTTCTTTATTGTAAACATTTACTTTATTGTGCATTCTAATGAAATGTATAGTTTAGTTTGGCAAATGCTGCTTTTTTTAATTATTTCAGGAAAAGT

BE_scaffold2403 ....................................................................................................

16910 16920 16930 16940 16950 16960 16970 16980 16990 17000

....|....|....|....|....|....|....|....|....|....|....|....|....|....|....|....|....|....|....|....|

WTE_scaffold23856 CCTCAGTCGTGCTCCACAAGTGGTAGCAACCCTGCATTCTGGGGGGGGGTTAAGCCCACAGGTAGTTAAGAACTAGCTCCTTTCACCGCTGAATAGCTGT

BE_scaffold2403 ....................................................................................................

17010 17020 17030 17040 17050 17060 17070 17080 17090 17100

....|....|....|....|....|....|....|....|....|....|....|....|....|....|....|....|....|....|....|....|

WTE_scaffold23856 GTTCATTCATGAATATTCATCTTTTTAGAAACAGTGCCTGTGCTCAGAAAATGTACCAGCTTTGAAAACCAGCAGAGATTCCTTTGATATCTTCATTTTA

BE_scaffold2403 ....................................................................................................

17110 17120 17130 17140 17150 17160 17170 17180 17190 17200

....|....|....|....|....|....|....|....|....|....|....|....|....|....|....|....|....|....|....|....|

WTE_scaffold23856 ATTGCAGTATCACACACCAAGTGAAAATTTCAAAATGACAGAAACTTTTAAATGTAAAATAAAGCTGAAGTGCCTAAGTTAAAAGCATTGTTACCTTCGG

BE_scaffold2403 ....................................................................................................

17210 17220 17230 17240 17250 17260 17270 17280 17290 17300

....|....|....|....|....|....|....|....|....|....|....|....|....|....|....|....|....|....|....|....|

WTE_scaffold23856 CTTCCTTCGTAGTCAATGTAGAGAGAGAATCCTGTGCTGTAGAAGAGCTTCTTTATCATGCCCTGAAGGTGCCTGGTTTTGCTGGCAAAAAAGAAAATTT

BE_scaffold2403 ....................................................................................................

17310 17320 17330 17340 17350 17360 17370 17380 17390 17400

....|....|....|....|....|....|....|....|....|....|....|....|....|....|....|....|....|....|....|....|

WTE_scaffold23856 GGCTACTCACCTTAGCACTTCATTAAGTCCCCAAAGATACATGAATGAAACAAAGTGTACATATTTCTACACCAGTAACAAAGACATGTGTTGTACTGTT

BE_scaffold2403 ....................................................................................................

17410 17420 17430 17440 17450 17460 17470 17480 17490 17500

....|....|....|....|....|....|....|....|....|....|....|....|....|....|....|....|....|....|....|....|

WTE_scaffold23856 GATAGGTCATTGTAGTAATATCAAGAAAATAAGCGATGTAAGACCCACTGCTGTTGAAATGTGCAACAGACATAGTATTTAGTGTTGTCACTAATTCAGT

BE_scaffold2403 ....................................................................................................

17510 17520 17530 17540 17550 17560 17570 17580 17590 17600

....|....|....|....|....|....|....|....|....|....|....|....|....|....|....|....|....|....|....|....|

WTE_scaffold23856 TCATTTTGGCTACCCCTCATGCATTTGATAATAAAAGTTTGCACTATTGAATTCTTATATTTTATTACCAATTTCTTCACTATTTTGTGATACAACATTC

BE_scaffold2403 ....................................................................................................

17610 17620 17630 17640 17650 17660 17670 17680 17690 17700

....|....|....|....|....|....|....|....|....|....|....|....|....|....|....|....|....|....|....|....|

WTE_scaffold23856 TAGTTTCCATGTATAACATGTAAGATTAAGCTAGGTAATTCCTATATTTTCTTTTGCCTTCTACTCTGCCATTGAACGTTTGAACATACCAGATCTGAAA

BE_scaffold2403 ....................................................................................................

17710 17720 17730 17740 17750 17760 17770 17780 17790 17800

....|....|....|....|....|....|....|....|....|....|....|....|....|....|....|....|....|....|....|....|

WTE_scaffold23856 ATTGATGTCATTTTGAAATTCTAAGCTTACACTGTCTTTATCAAATAGGAAAAGAATGGACTAGAGAATAGATGATTAAAAATATGTTGTTCAGTGTAAA

BE_scaffold2403 ....................................................................................................

17810 17820 17830 17840 17850 17860 17870 17880 17890 17900

....|....|....|....|....|....|....|....|....|....|....|....|....|....|....|....|....|....|....|....|

WTE_scaffold23856 TATATAAAAATTCATTAAGAAAATTAAAATTTTAGCCTTTGATGAATATAGGAAAAGAGTTAATTCAAGTTTTATTTTTGAAGTATTTGATGTCATCAGC

BE_scaffold2403 ....................................................................................................

17910 17920 17930 17940 17950 17960 17970 17980 17990 18000

....|....|....|....|....|....|....|....|....|....|....|....|....|....|....|....|....|....|....|....|

WTE_scaffold23856 TGCTGTAAAGCTGTGTGTCTAATATTGTTTTGCACATAAAATGTCATGCCATTAATTTTCATACCAAGTAAGTTTTAGGAGAGCTCATTCAGGTTCCTTT

BE_scaffold2403 ....................................................................................................

18010 18020 18030 18040 18050 18060 18070 18080 18090 18100

....|....|....|....|....|....|....|....|....|....|....|....|....|....|....|....|....|....|....|....|

WTE_scaffold23856 TTTCTCTCTGCTATGGTGTTTAAACCTTGCTACTGGATCAGAGCTTTTACTTCTGTCATGAAAAGTCACAAAATACATTGTAGAACTTAGCAAACTGCAG

BE_scaffold2403 ....................................................................................................

18110 18120 18130 18140 18150 18160 18170 18180 18190 18200

....|....|....|....|....|....|....|....|....|....|....|....|....|....|....|....|....|....|....|....|

WTE_scaffold23856 CTTCATTTGACTATGTAGGGACATATTTTGACTAGGTATCTTTTAATTTTTCCTTCAGGAAAAACATGTGGACATATCTATCACTTGGGCTATTTTTACA

BE_scaffold2403 ....................................................................................................

18210 18220 18230 18240 18250 18260 18270 18280 18290 18300

....|....|....|....|....|....|....|....|....|....|....|....|....|....|....|....|....|....|....|....|

WTE_scaffold23856 TACAATAGCATTCTTTTTGGAGAGGGCAACGTATTCTGTTGTGGAATCAGAATATCATTGCTAGTGGAATATGCTATCTCATGAGGAAAACATTCTTAAT

BE_scaffold2403 ....................................................................................................

18310 18320 18330 18340 18350 18360 18370 18380 18390 18400

....|....|....|....|....|....|....|....|....|....|....|....|....|....|....|....|....|....|....|....|

WTE_scaffold23856 TCATGCAGCACAGCATGAGTAGACAGCTTTCTTTTTCAAAATATACATATCTATAAATATCCCAAATTATACAAAAATGCATATTATTGCAGGTATGTCA

BE_scaffold2403 ....................................................................................................

18410 18420 18430 18440 18450 18460 18470 18480 18490 18500

....|....|....|....|....|....|....|....|....|....|....|....|....|....|....|....|....|....|....|....|

WTE_scaffold23856 TTATATTATTCTCAAGTAAGAAAAGCCGTGGACAACATACTCAGACACCTTGACAAGGAAGTGGGTCGGTGCATGATGCTAACCAACATACAGATGCTTA

BE_scaffold2403 ....................................................................................................

18510 18520 18530 18540 18550 18560 18570 18580 18590 18600

....|....|....|....|....|....|....|....|....|....|....|....|....|....|....|....|....|....|....|....|

WTE_scaffold23856 ACAAAGAACCTGAAGACATGATTACGTGAGTGCTAAAGAACAGTAATTTCCATAAAATATTGAGACGTAATTTCAGAACTTTTTCTCTACTTTTATCTAT

BE_scaffold2403 ....................................................................................................

18610 18620 18630 18640 18650 18660 18670 18680 18690 18700

....|....|....|....|....|....|....|....|....|....|....|....|....|....|....|....|....|....|....|....|

WTE_scaffold23856 TGTGAGTCATCAGAGAAATAGCAAGAAACAGGATTATTTAGCTTAGTTAGTATATACTTTGCAAAACTGTTTATGACAAGTTGATTGTACTTCTTTACAT

BE_scaffold2403 ..............................A.....................................................................

18710 18720 18730 18740 18750 18760 18770 18780 18790 18800

....|....|....|....|....|....|....|....|....|....|....|....|....|....|....|....|....|....|....|....|

WTE_scaffold23856 GTTGCAATGTTTGGAACTGAACTGATGCTCTCGACATAGCCAAAATCATGTGTGAATCCTATGAATGTTTAGATGGTAGTAGCTGAAAACCCAAAGTGGA

BE_scaffold2403 ....................................................................................................

18810 18820 18830 18840 18850 18860 18870 18880 18890 18900

....|....|....|....|....|....|....|....|....|....|....|....|....|....|....|....|....|....|....|....|

WTE_scaffold23856 AAACCTTCGCTCAGGTCACTGTTTCAGTGTATATTTGCATCACTGAACTTCAAAATTAATGCAACAAATTCTGTAAATCCCTATTAATAATAATATTTCA

BE_scaffold2403 ....................................................................................................

18910 18920 18930 18940 18950 18960 18970 18980 18990 19000

....|....|....|....|....|....|....|....|....|....|....|....|....|....|....|....|....|....|....|....|

WTE_scaffold23856 GAATTTCCATTACTATTCATGCCTGTAATCAAAATCAAATCTTAATTACAAACCCAAGTCATTTTGTGGTAACAGTCTTGGATTTAAGCAGCACCCCACT

BE_scaffold2403 ....................................................................................................

19010 19020 19030 19040 19050 19060 19070 19080 19090 19100

....|....|....|....|....|....|....|....|....|....|....|....|....|....|....|....|....|....|....|....|

WTE_scaffold23856 GGACCAGAATCAAACTAGTACATCTCAAGCAGTGTTGAACAGCAGCATTTTACACTCATCCAAAATTATCGATGAGGGAAGTTTCCCAGAGAGAGTTCTA

BE_scaffold2403 .....................................................................T.T............................

19110 19120 19130 19140 19150 19160 19170 19180 19190 19200

....|....|....|....|....|....|....|....|....|....|....|....|....|....|....|....|....|....|....|....|

WTE_scaffold23856 GTGAGGTGGATATCTAGTAGAATTTAGAGACACAGAGATATCATTTGACACATGCAGAGCACAGGTTGTAGAGACCCCAGCTGTAAGGAGACCTTGCATA

BE_scaffold2403 ....................................................................................................

19210 19220 19230 19240 19250 19260 19270 19280 19290 19300

....|....|....|....|....|....|....|....|....|....|....|....|....|....|....|....|....|....|....|....|

WTE_scaffold23856 TCTTTCCTGTTAGAGGGGTGGACATGGTAGTTACGCTAGAAGAGAAACAACCCCTGGAAAAAATGTTTGAGGTGTAATGAAAAGAACATTGGTCTCTTCG

BE_scaffold2403 ....................................................................................................

19310 19320 19330 19340 19350 19360 19370 19380 19390 19400

....|....|....|....|....|....|....|....|....|....|....|....|....|....|....|....|....|....|....|....|

WTE_scaffold23856 AGATTGTTTTTTTTCATATACTGTCAAAAAAAGAGACAACTTGCTAAAAATACCATGAGTGGTAGGTAGACTGCAATGACTGGCCAAATTCAACTACCTT

BE_scaffold2403 ......G.............................................................................................

19410 19420 19430 19440 19450 19460 19470 19480 19490 19500

....|....|....|....|....|....|....|....|....|....|....|....|....|....|....|....|....|....|....|....|

WTE_scaffold23856 TTACAGCAGATATAATCTTTGTGTTATAATGTCATTCCTCTGCCCTTGTTTGTTGCTGTGACTGTGTAGATGAGATTGTCAGCTCTTTGTGTCTGTAGCA

BE_scaffold2403 ....................................................................................................

19510 19520 19530 19540 19550 19560 19570 19580 19590 19600

....|....|....|....|....|....|....|....|....|....|....|....|....|....|....|....|....|....|....|....|

WTE_scaffold23856 TTGCCTTACTTTATTTATATTAAATGCTAATATTAGTATCTGATAATATTTCATATTTTTCTTATAGTACTACCCAGTATACTGCTCAGTACTCACACCC

BE_scaffold2403 ....................................................................................................

19610 19620 19630 19640 19650 19660 19670 19680 19690 19700

....|....|....|....|....|....|....|....|....|....|....|....|....|....|....|....|....|....|....|....|

WTE_scaffold23856 AGTGTTCATAACCACTTTGGGTATCCAGGCTGTTGAGAGAGGGGAATTGAGTTTTGAAACTCACATTTTATTCCCAAAAGATAATTGCATAGTAAAACCT

BE_scaffold2403 .............NNNNNNNNNNNNNNNNNNNNNNNNNNNNNNNNNNNNNNNNNNNNNNNNNNNNNNNNNNNNNNNNNNNNNNNNNNNNNNNNNNNNNNN

19710 19720 19730 19740 19750 19760 19770 19780 19790 19800

....|....|....|....|....|....|....|....|....|....|....|....|....|....|....|....|....|....|....|....|

WTE_scaffold23856 TGAGCACTTATATAAGAAGCATACTTGACCTTAGCTATAAATGTCCTTTCTGTGGTTTCTCAGCATCACTAAGGTGGTGTTTATTTTTAGAGTCAGTG--

BE_scaffold2403 NNNNNNNNNNNNNNNNNNNNNNNNNNNNNNNNNNNNNNNNNNNNNNNNNNNNNNNNNNNNNNNNNNNNNNNNNNNNNNNNNNNNNNNNNNNNNNNNNNCC

19810 19820 19830 19840 19850 19860 19870 19880 19890 19900

....|....|....|....|....|....|....|....|....|....|....|....|....|....|....|....|....|....|....|....|

WTE_scaffold23856 ----------------------------------------------------------------------------------------------------

BE_scaffold2403 AAAAGATAATTGCATAGTAAAACCTTGAGCACTTATATAAGAAGCATACTTGACCTTAGCTATAAATGTCCTTTCTGTGGTTTCTCAGCATCACTAAGGT

19910 19920 19930 19940 19950 19960 19970 19980 19990 20000

....|....|....|....|....|....|....|....|....|....|....|....|....|....|....|....|....|....|....|....|

WTE_scaffold23856 -----------------------AAAAAATAGACTTAAAAGAGTTTTAGATTTCAGGAAGTCTTATGGTTCGGTAGTAACTTTTGTCAAGAGACATAAAG

BE_scaffold2403 GGTGTTTATTTTTAGAGTCAGTG.............................................................................

20010 20020 20030 20040 20050 20060 20070 20080 20090 20100

....|....|....|....|....|....|....|....|....|....|....|....|....|....|....|....|....|....|....|....|

WTE_scaffold23856 ATTTTAAAGGTTGGAAGATATAGACATGCATGTTAATGTGTGTTTGTGTTGTGCTTTATATCCAGTTGACCTATAACTTAATATATACACTTGAAAGAAA

BE_scaffold2403 ....................................................................................................

20110 20120 20130 20140 20150 20160 20170 20180 20190 20200

....|....|....|....|....|....|....|....|....|....|....|....|....|....|....|....|....|....|....|....|

WTE_scaffold23856 AAACACATCAGAACACATTTTTACTTGTTCTTCTAAGGAAAAAAAATCCAGGATGCTTAATTATCAGTCGATCTAAAATCATGTGAACTCATGAAAAACA

BE_scaffold2403 ....................................................................................................

20210 20220 20230 20240 20250 20260 20270 20280 20290 20300

....|....|....|....|....|....|....|....|....|....|....|....|....|....|....|....|....|....|....|....|

WTE_scaffold23856 TCATTGCCTGGTAGTATAAGCATTTAAAATAGCCCTTTTTAAAACTGCATTTTGCATGATAACTGGAAAAGCCAGTATATTGGTTTTATGTAATTGCTTA

BE_scaffold2403 ....................................................................................................

20310 20320 20330 20340 20350 20360 20370 20380 20390 20400

....|....|....|....|....|....|....|....|....|....|....|....|....|....|....|....|....|....|....|....|

WTE_scaffold23856 TCATTTTACTCACAGAACTTCATGCTTCACTAGAGTTACGTATTTATTTCAGTTCTTATGGGCTTTAATACATAGATACCACACTACAATTATCTATATT

BE_scaffold2403 ....................................................................................................

20410 20420 20430 20440 20450 20460 20470 20480 20490 20500

....|....|....|....|....|....|....|....|....|....|....|....|....|....|....|....|....|....|....|....|

WTE_scaffold23856 TTAAAGTTATGGCTTTTGTCATATTTACATATAGTTAATGTAACTGGATATTGTAATGGTATTTTTAATAACAAAATAAGAATCAGAGGGAAGTCGCTAG

BE_scaffold2403 ....................................................................................................

20510 20520 20530 20540 20550 20560 20570 20580 20590 20600

....|....|....|....|....|....|....|....|....|....|....|....|....|....|....|....|....|....|....|....|

WTE_scaffold23856 GCAATGAATAGTCATAACATTTTAAGTATACAGATTCTGGAATTTTCTTTGGTAGTGTTAGACATGCTTCTGTATTTAATGACTGATCCTTTAAGCATTT

BE_scaffold2403 ....................................................................................................

20610 20620 20630 20640 20650 20660 20670 20680 20690 20700

....|....|....|....|....|....|....|....|....|....|....|....|....|....|....|....|....|....|....|....|

WTE_scaffold23856 ATTGTTGAAGTAGTAAATGCCACTGGAGAGTCTTACTAAAATCCTCACTAAGGGTCATCCTTTGCATCCTCCACTGGGATATCCATTTCAATACTGGTTA

BE_scaffold2403 ....................................................................................................

20710 20720 20730 20740 20750 20760 20770 20780 20790 20800

....|....|....|....|....|....|....|....|....|....|....|....|....|....|....|....|....|....|....|....|

WTE_scaffold23856 TAATTACTTTCCAGCATCTAGTTAGGATCGGGCATCCATTTATTCTAACATACAAACCTGTTCTTGCAAGTACTTCAAAGATGTTGGCTGACAATACCTA

BE_scaffold2403 ....................................................................................................

20810 20820 20830 20840 20850 20860 20870 20880 20890 20900

....|....|....|....|....|....|....|....|....|....|....|....|....|....|....|....|....|....|....|....|

WTE_scaffold23856 ACTAAAAGCTGATACCGCTGGTATGGAAACACAAGCTTCCAGGTGAGGACAGCTGTTGGTGTGTTCATCCAGAGCTCGTCCTGTTGGATTCTGCGTCGGG

BE_scaffold2403 ....................................................................................................

20910 20920 20930 20940 20950 20960 20970 20980 20990 21000

....|....|....|....|....|....|....|....|....|....|....|....|....|....|....|....|....|....|....|....|

WTE_scaffold23856 CAGCGATGAGCGCAGAGCCCTGTGAGGCCCCCCCTTGCCAGACACTTCACATGCTGTGCCTGGTAGCTCAGATAAGTTTGGAACTTGAGCCTCAGGTTTG

BE_scaffold2403 ....................................................................................................

21010 21020 21030 21040 21050 21060 21070 21080 21090 21100

....|....|....|....|....|....|....|....|....|....|....|....|....|....|....|....|....|....|....|....|

WTE_scaffold23856 CATCTTCTCCTGCTAGTCTTCTGAGCAGCTGATAAGCCATAGAACACCCACAGATTTTATATTTATAAGTAATTACTCATTTGCCTTCAGAAGAGTTTCC

BE_scaffold2403 ....................................................................................................

21110 21120 21130 21140 21150 21160 21170 21180 21190 21200

....|....|....|....|....|....|....|....|....|....|....|....|....|....|....|....|....|....|....|....|

WTE_scaffold23856 TCTCAGGCTATTAGTTTACGTGCCTCCATTTTAAACCCACAAACTGTTTACCCTTGCTAGGAACTGATAATAAGGAAAATCTGCAAAGTTCACTTTCCAT

BE_scaffold2403 ....................................................................................................

21210 21220 21230 21240 21250 21260 21270 21280 21290 21300

....|....|....|....|....|....|....|....|....|....|....|....|....|....|....|....|....|....|....|....|

WTE_scaffold23856 TTCAAGTCAGCAAGGAAGATTCACTGGTTTGCCTGCAGATAAAATTACCTATAGCACTTTACTGTTTACAACTGTAAAAATATAATATTTTGCACTAGTT

BE_scaffold2403 ....................................................................................................

21310 21320 21330 21340 21350 21360 21370 21380 21390 21400

....|....|....|....|....|....|....|....|....|....|....|....|....|....|....|....|....|....|....|....|

WTE_scaffold23856 GTTAATCCAACTAGTATTTGCATTAGCAGTAATAGTTCAAATGGATTTGTTTATAACCAAATTCTCAGATGAGATGTTGTATTTTACTCTTTTTCAAGTC

BE_scaffold2403 ....................................................................................................

21410 21420 21430 21440 21450 21460 21470 21480 21490 21500

....|....|....|....|....|....|....|....|....|....|....|....|....|....|....|....|....|....|....|....|

WTE_scaffold23856 AGTTGCTGACACCTTCTTTCAAGTTCTGTCTGTTAGGATACAGAGACAGTGAGACAGATAATTCATTACGAAAATAGTTTCCCCTCATTGTACAATTTAG

BE_scaffold2403 ....................................................................................................

21510 21520 21530 21540 21550 21560 21570 21580 21590 21600

....|....|....|....|....|....|....|....|....|....|....|....|....|....|....|....|....|....|....|....|

WTE_scaffold23856 GTCAGTAAATTAAAATCATATCATCCTTTTATTAATGTAGTATATATCCAAGCTGATAAGTTTATAGGGTGTTAAGAGAATACAAAACTGTCTTTAATCT

BE_scaffold2403 ....................................................................................................

21610 21620 21630 21640 21650 21660 21670 21680 21690 21700

....|....|....|....|....|....|....|....|....|....|....|....|....|....|....|....|....|....|....|....|

WTE_scaffold23856 TTGAATTAAGTAAGCCTAATGTCAGTTTATGCCTTTTCTGTACCCATTATTTCAGTGAATGCTCAGTGTTAATCTATAAATCTTACTTTAGACTTGTGAG

BE_scaffold2403 ....................................................................................................

21710 21720 21730 21740 21750 21760 21770 21780 21790 21800

....|....|....|....|....|....|....|....|....|....|....|....|....|....|....|....|....|....|....|....|

WTE_scaffold23856 ATGATATTGCACTCAGGCTTTCTCTGTGACTGAACACAGAAGTAAGGTATCCATATGCAATGTCCTCCTGTTGGGTGCAGAGGGGAGTAATGTAGTAAGC

BE_scaffold2403 ....................................................................................................

21810 21820 21830 21840 21850 21860 21870 21880 21890 21900

....|....|....|....|....|....|....|....|....|....|....|....|....|....|....|....|....|....|....|....|

WTE_scaffold23856 AGGGCATTGGACTGGCATGCAGGAAAATTAGGTTCATTTTCTGTTTGTCACTGACTTTCAGTGTGGTCTTAAGTAGCTTACAATGCTTCTCAGTGTCTCT

BE_scaffold2403 ....................................................................................................

21910 21920 21930 21940 21950 21960 21970 21980 21990 22000

....|....|....|....|....|....|....|....|....|....|....|....|....|....|....|....|....|....|....|....|

WTE_scaffold23856 CTTCCCTCCTGCCCCCCTGCCCCGGTTCTTTTCTAGTTTGATTGGAAGCATATTAAAGCAGGAACAGTCTTTCCCTGCATGTTTATAGGAAAGTCGCTAA

BE_scaffold2403 ....................................................................................................

22010 22020 22030 22040 22050 22060 22070 22080 22090 22100

....|....|....|....|....|....|....|....|....|....|....|....|....|....|....|....|....|....|....|....|

WTE_scaffold23856 ATAGGACCTTGCTTTTTGATTCTTCCGAAGCATATTACAGGCTAATAATTAATAATACTGTGGGAAAGACTGTACAGTTTGAAATGCTGTTACGCTTTGG

BE_scaffold2403 ....................................................................................................

22110 22120 22130 22140 22150 22160 22170 22180 22190 22200

....|....|....|....|....|....|....|....|....|....|....|....|....|....|....|....|....|....|....|....|

WTE_scaffold23856 TTATTTCCTCTATACTCAATCATCAAATAATAAATCATCGAGTTAAAAATGAGCAAATTAAAAAAAAAACATTTTGGGGTCTGTCTGAAGGACTTCTTTA

BE_scaffold2403 ....................................................................-...............................

22210 22220 22230 22240 22250 22260 22270 22280 22290 22300

....|....|....|....|....|....|....|....|....|....|....|....|....|....|....|....|....|....|....|....|

WTE_scaffold23856 ATCTGCATTCTTACAAAAAAATACTGGAAACAAGTCAGAAAACTGCATCTGTTGTGAAAATAATCAGCAGCTGAAACTTGAGCTACTTTTAGAAGACTTA

BE_scaffold2403 ....................................................................................................

22310 22320 22330 22340 22350 22360 22370 22380 22390 22400

....|....|....|....|....|....|....|....|....|....|....|....|....|....|....|....|....|....|....|....|

WTE_scaffold23856 AGGACTCCCAACCTATTTGGTTCATGCTGAACTTTGTGCTCAACCACTTTCCCAGGCTGTCAGGGAGCAGTTGAGCCATTCCACAGGTCAGAGCAGCCTT

BE_scaffold2403 ....................................................................................................

22410 22420 22430 22440 22450 22460 22470 22480 22490 22500

....|....|....|....|....|....|....|....|....|....|....|....|....|....|....|....|....|....|....|....|

WTE_scaffold23856 GGGGCTACTAAAACATATTTTAGCTGATAATGGTTGCTAAAGTGCTGCTATGTCAGACTGGGATCAACAGAAACTCTCTGGCCATACCTACCTCTAAATT

BE_scaffold2403 ..........................................................A.........................................

22510 22520 22530 22540

....|....|....|....|....|....|....|....|....

WTE_scaffold23856 ATGCTTCTTTTTCAATGTAATAATGTGTGGTGTGTGATTTCCAT

BE_scaffold2403 ............................................

**(ii) *Haliaeetus albicilla* 2 and *H. leucocephalus* 2**

10 20 30 40 50 60 70 80 90 100

....|....|....|....|....|....|....|....|....|....|....|....|....|....|....|....|....|....|....|....|

WTE_scaffold35452 CCATGCTCTACCACGGCGCCCGGGCAGCACCAACACGGCGCATCGGCTCTTGCCGGGACGGAGGGACTTGGTCCCGCCGGTGCCACCCCAACTCCGGACC

BE_scaffold146 ....................................................................................................

110 120 130 140 150 160 170 180 190 200

....|....|....|....|....|....|....|....|....|....|....|....|....|....|....|....|....|....|....|....|

WTE_scaffold35452 CCCCACCGGGGCTGGAGCCCACCTGCGTGGCTGGGGAAAGGTCAAAACAACCCGCAGCAGGGAAACGCTCTCCTAAATTGAGTCCTCTGTGCTGAAACAA

BE_scaffold146 .....................................................................................C..............

210 220 230 240 250 260 270 280 290 300

....|....|....|....|....|....|....|....|....|....|....|....|....|....|....|....|....|....|....|....|

WTE_scaffold35452 GGGGTTTGTTTTAGTTTTTAAAAGCGATAGCTTTTTTTTTTAATGAGAAAATGCCGTTTNNNNNNNNNNNNNNNNNNNNNNNNNNNNNNNAAACTGATTT

BE_scaffold146 ........................................................................................----........

310 320 330 340 350 360 370 380 390 400

....|....|....|....|....|....|....|....|....|....|....|....|....|....|....|....|....|....|....|....|

WTE_scaffold35452 TTAAAGACAAGGAGATTGCTGCTTGCTCTCACTTCTCATTCCCAGAGGAGAAAAAGGGAAGGATCAAAAATAAATAGATTAAGTGAAGGGGTAAAGGGAT

BE_scaffold146 ....................................................................................................

410 420 430 440 450 460 470 480 490 500

....|....|....|....|....|....|....|....|....|....|....|....|....|....|....|....|....|....|....|....|

WTE_scaffold35452 AAACCTTGGGGGGAAAAAAACAACACCACAACAACAAAGGAAAAGTTTAAAAGGCGCAGATATGAGGGCACAGTGCTTTCCAGCTCAGCTCCTACAGAGA

BE_scaffold146 ....................................................................................................

510 520 530 540 550 560 570 580 590 600

....|....|....|....|....|....|....|....|....|....|....|....|....|....|....|....|....|....|....|....|

WTE_scaffold35452 AACCAAACAACTGTGTTTGGGTTTCTCTTTTTGAGAGAGGGGGAAAAAAGGAGAGTAAAAAGCCAGAGCAACACAGGAATAAAAATCACATTTCTGGGGG

BE_scaffold146 ....................................................................................................

610 620 630 640 650 660 670 680 690 700

....|....|....|....|....|....|....|....|....|....|....|....|....|....|....|....|....|....|....|....|

WTE_scaffold35452 GAAAAAAACAAACCAAACCATTTCTCATCCAGACGCATCCAGCCCCAACTGTCCCCAGCACCTTCTCCAGGAGCTGTACTGTGTCCGGCTAAGT------

BE_scaffold146 .....................................................................................A........AAGAGC

710 720 730 740 750 760 770 780 790 800

....|....|....|....|....|....|....|....|....|....|....|....|....|....|....|....|....|....|....|....|

WTE_scaffold35452 --------------------------------------------------------------------------------------TAAGTAAGAGCCCA

BE_scaffold146 CCAATCGCAGGGTCTTCTCTCCCACTCCAGGCTCATGCACCCCAAATCAGCCCCGAGACCTCCCTTCCCCGCTTTCTGCTGCCTCC.GG.C.G..T....

810 820 830 840 850 860 870 880 890 900

....|....|....|....|....|....|....|....|....|....|....|....|....|....|....|....|....|....|....|....|

WTE_scaffold35452 AT-----------------------------------------------CGCAGGGTCTTCTCTCCCACTCCAGGCTCATGCACCCCAAATCAGCCCCGA

BE_scaffold146 CCTCGGCTGCGCCTTTGCAGGGGGGGCTGCTGCTGTCACCCCCTCCCGGNNNNNNNNNNNNNNNNNNNNNNNNNNNNNNNNNNNNNNNNNNNNNNNNNNN

910 920 930 940 950 960 970 980 990 1000

....|....|....|....|....|....|....|....|....|....|....|....|....|....|....|....|....|....|....|....|

WTE_scaffold35452 GACCTCCCTTCCCCGCTTTCTGCTGCCTCCTGGGCAGGATCCCACCTCGGCTGCGCCTTTGCAGGGGGGGCTGCTGCTGTCACCCCCTCCCGGGACATCC

BE_scaffold146 NNNNNNNNNNNNNNNNNNNNNNNNNNNNNNNNNNNNNNNNNNNNNNNNNNNNNNNNNNNNNNNNNNNNNNNNNNNNNNNNNNNNNNNNNNNNNNNNNNNN

1010 1020 1030 1040 1050 1060 1070 1080 1090 1100

....|....|....|....|....|....|....|....|....|....|....|....|....|....|....|....|....|....|....|....|

WTE_scaffold35452 CCAGCACCTTCAGGGGACTCGGGGACCAGAAAAATGGGGAAAGCCTATTCCCATGGGGTTGCTGACACCCCGGTGAGCGCGGGGGTGCCACGGGGACTTC

BE_scaffold146 NNNNNNNNNNNNNNNNNNNNNNNNNNNNNNNNNNNNNNNNNNNNNNNNNNNNNNN.............................................

1110 1120 1130 1140 1150 1160 1170 1180 1190 1200

....|....|....|....|....|....|....|....|....|....|....|....|....|....|....|....|....|....|....|....|

WTE_scaffold35452 ACACCAGCCACACACGTGGTCCAGGTCCGCGATCATTCAAACGATGCCAGGAGCCATGGCCGGCGAGTTTTGGTGTCTCACAGCTGGCATTCCCCCATGC

BE_scaffold146 ............................A.......................................................................

1210 1220 1230 1240 1250 1260 1270 1280 1290 1300

....|....|....|....|....|....|....|....|....|....|....|....|....|....|....|....|....|....|....|....|

WTE_scaffold35452 CACATCCAAGCCAGGGTCCAAAAAGCATCAGCTGGGGGCTGAGCATCCCAGAGAGCGGGGCAAGGGGTGATGGCTCCAAAACTCCACTTGGGAAACACCT

BE_scaffold146 ....................................................................................................

1310 1320 1330 1340 1350 1360 1370 1380 1390 1400

....|....|....|....|....|....|....|....|....|....|....|....|....|....|....|....|....|....|....|....|

WTE_scaffold35452 TCCCCGAGGGCACCAACTTTACAGAGTTGGCATAAAACATCCTCTAGTTGCAAGAAAACTTTGCCCTGAGCAGGATGAGGCCCTTAATAAACACAGGGAA

BE_scaffold146 ....................................................................................................

1410 1420 1430 1440 1450 1460 1470 1480 1490 1500

....|....|....|....|....|....|....|....|....|....|....|....|....|....|....|....|....|....|....|....|

WTE_scaffold35452 AGCAGCGTGGGATAGGGATCAGTCGGGTTTTAACAAACCCTGGCTTCCAGCAGCATAAACACACAACGAATACAAACGGTTTCTATCAATGCGAGACTTT

BE_scaffold146 ....................................................................................................

1510 1520 1530 1540 1550 1560 1570 1580 1590 1600

....|....|....|....|....|....|....|....|....|....|....|....|....|....|....|....|....|....|....|....|

WTE_scaffold35452 GGACCAACATCGAGGTCCTCCCGAGGAGCCGCTCATGCTGAACAAGGGAAAATTCAGCTCTCGCGGCAGTGGGATGGGACATTCAGAAATGTTGAGCCCT

BE_scaffold146 ....................................................................................................

1610 1620 1630 1640 1650 1660 1670 1680 1690 1700

....|....|....|....|....|....|....|....|....|....|....|....|....|....|....|....|....|....|....|....|

WTE_scaffold35452 TGGAAAAGCAAACAAGGGGGAACAGCTTGGGCCTTGCCAGGAGCATCTTCCCCGCGTGGCTTTGCTTTCCAGGGAAGCCATGCTGCTGCTGGAGCAGATA

BE_scaffold146 ....................................................................................................

1710 1720 1730 1740 1750 1760 1770 1780 1790 1800

....|....|....|....|....|....|....|....|....|....|....|....|....|....|....|....|....|....|....|....|

WTE_scaffold35452 CCAGGGACAGGATGGGCTCGTAGGTGATGCCAGGGAGATGGGGGACATCTCCACCAAGCCTCGCAGAACCAACCACGCGTGGAAATTGAAACCCACCACG

BE_scaffold146 ....................................................................................................

1810 1820 1830 1840 1850 1860 1870 1880 1890 1900

....|....|....|....|....|....|....|....|....|....|....|....|....|....|....|....|....|....|....|....|

WTE_scaffold35452 CAACGAAAGCTCGATGCTCGCAGCTGAGGGTGGGGGTCCTGGGGTGTGAGGGACACAGGCAGCAGGGTTGCACCCTGCAAAGGCAGGCGTGGGGCTGGCT

BE_scaffold146 ....................................................................................................

1910 1920 1930 1940 1950 1960 1970 1980 1990 2000

....|....|....|....|....|....|....|....|....|....|....|....|....|....|....|....|....|....|....|....|

WTE_scaffold35452 CTGTGGTGCCGTCTTCCCATCTCTGGAGGAGGTGGTAACAACAACTGAAGCGCGTCTTGGAAAGCAGCTAGACGATGATCAACATCACACTCCTCATCAT

BE_scaffold146 .........................................................................A..........................

2010 2020 2030 2040 2050 2060 2070 2080 2090 2100

....|....|....|....|....|....|....|....|....|....|....|....|....|....|....|....|....|....|....|....|

WTE_scaffold35452 ACAGCCTTGAGGAATGCTCTGGTATGCTATTATGAATATAAGAACTTTGGGCAGAAAATATCAGCGTTAATACCTGACAGAAGTCTAGCTGCTGAACTTA

BE_scaffold146 ..............................................................G.....................................

2110 2120 2130 2140 2150 2160 2170 2180 2190 2200

....|....|....|....|....|....|....|....|....|....|....|....|....|....|....|....|....|....|....|....|

WTE_scaffold35452 TTCAATATTCAGATACAAATTTAGGTATTAGCCTCAGGAGACAATTTGGATTCCATTTATCTTGCTTAGAATTTGGGGACAATTGAATTTATAATTTCCT

BE_scaffold146 ....................................................................................................

2210 2220 2230 2240 2250 2260 2270 2280 2290 2300

....|....|....|....|....|....|....|....|....|....|....|....|....|....|....|....|....|....|....|....|

WTE_scaffold35452 CTGGAACCTGGATAAGAACTCCTGCTGCATATAGACCACCAAATGCACCCATTCTTTCATCCTTACCTGAGCACTGGCATAGACCAAGGAGAAGAAGATC

BE_scaffold146 ....................................................................................................

2310 2320 2330 2340 2350 2360 2370 2380 2390 2400

....|....|....|....|....|....|....|....|....|....|....|....|....|....|....|....|....|....|....|....|

WTE_scaffold35452 AAGAACTCCATCTAGATCTCCTAGAAGAAGCAGTCATTCAAAGTCCCTGCACAGAAGATGATCTCCATCTCCCAAAAAGTGATCAGCCCACCTTACACAT

BE_scaffold146 ....................................................................................................

2410 2420 2430 2440 2450 2460 2470 2480 2490 2500

....|....|....|....|....|....|....|....|....|....|....|....|....|....|....|....|....|....|....|....|

WTE_scaffold35452 CCTTAAGGACTTCGGAGGACTATATCACAATCCTGAATCAAAATTTAATAAGGACTGGAAAATTCCTCAATTTTCTTCTTGTCATCTAGAAGAAAATTAT

BE_scaffold146 ....................................................................................................

2510 2520 2530 2540 2550 2560 2570 2580 2590 2600

....|....|....|....|....|....|....|....|....|....|....|....|....|....|....|....|....|....|....|....|

WTE_scaffold35452 AAATTAGCCATACTAGATAGTGGGTGGCCCCTACTGCTATCTGCACACTTATAGGTACCAAGTGCAACTTATTTGCCATTACTTTGTGCCATCAAAAATA

BE_scaffold146 ....................................................................................................

2610 2620 2630 2640 2650 2660 2670 2680 2690 2700

....|....|....|....|....|....|....|....|....|....|....|....|....|....|....|....|....|....|....|....|

WTE_scaffold35452 AATATGAAGCATCATTACTTCATCTGGTCATGTGTGCTGACTACCTATGTAATCTTTTTGAGTCTGGTATGGTTTGTAGAAAAGGCAGTAATAAGCACTG

BE_scaffold146 ....................................................................................................

2710 2720 2730 2740 2750 2760 2770 2780 2790 2800

....|....|....|....|....|....|....|....|....|....|....|....|....|....|....|....|....|....|....|....|

WTE_scaffold35452 GGAGTGTTGCTTACCGCCTATAGATGGGAGGCATAATAGGGCTGTGAGCCCCTCAATTTCAACCAGCAAAAAAATTTGTATCAACGTCAAGAAGATTCTA

BE_scaffold146 ....................................................................................................

2810 2820 2830 2840 2850 2860 2870 2880 2890 2900

....|....|....|....|....|....|....|....|....|....|....|....|....|....|....|....|....|....|....|....|

WTE_scaffold35452 TCTCAGGAATGGAAACAAGACAGAAGATAGTATCTCAGGGATCATGGGTACCTGATAAGTCCCACAAACTTGGAATTGACTGGGGCAAGGACCCAACTGA

BE_scaffold146 ....................................................................................................

2910 2920 2930 2940 2950 2960 2970 2980 2990 3000

....|....|....|....|....|....|....|....|....|....|....|....|....|....|....|....|....|....|....|....|

WTE_scaffold35452 TACAAGATGCAGCAAAGGTCCTTACTGGACTAAAGAAGAAGGAACCTCCTCCGGCACCACCTCAACCACAACCACCTCCAGGGCAACCACCCCCATCTGT

BE_scaffold146 ....................................................................................................

3010 3020 3030 3040 3050 3060 3070 3080 3090 3100

....|....|....|....|....|....|....|....|....|....|....|....|....|....|....|....|....|....|....|....|

WTE_scaffold35452 AGGGAATTGCCCCAAACAGGGTGGATGTGCTAATGCAAATCCACCAGGATGCTCTGGCACAAGAAGTGGGATTGGGGTCTCAAGATTCTTTACAGGTGCT

BE_scaffold146 ....................................................................................................

3110 3120 3130 3140 3150 3160 3170 3180 3190 3200

....|....|....|....|....|....|....|....|....|....|....|....|....|....|....|....|....|....|....|....|

WTE_scaffold35452 GGATCCGGTAGCATTCCAGAAACTGAAGCAAGAAATGGGAGATGCAAGAAACAAGGACGAATTACAAGCAGCGATCAACAGAGTAAATGGGGCATGGGTA

BE_scaffold146 ....................................................................................................

3210 3220 3230 3240 3250 3260 3270 3280 3290 3300

....|....|....|....|....|....|....|....|....|....|....|....|....|....|....|....|....|....|....|....|

WTE_scaffold35452 AAGGTAAAACGGGCACCCTGAAGGAGTTTCCAGTCACAAAACAAATTTGTAAGGCCCACTGCAACATCCAGGAATGGTTTATCCTTACCGGCCTCTGGAA

BE_scaffold146 ....................................................................................................

3310 3320 3330 3340 3350 3360 3370 3380 3390 3400

....|....|....|....|....|....|....|....|....|....|....|....|....|....|....|....|....|....|....|....|

WTE_scaffold35452 CAGCATCCCACAGTACCGCAGGGACATCCTTGGCAGTACCACAACAGGAGAGAGGGTCTGCACGTATAGAACGTGTGCCTATCATAGTCAAATCAGAAGT

BE_scaffold146 ....................................................................................................

3410 3420 3430 3440 3450 3460 3470 3480 3490 3500

....|....|....|....|....|....|....|....|....|....|....|....|....|....|....|....|....|....|....|....|

WTE_scaffold35452 GTCCCCATGACTGGTTGTATTGTTAAAAGGGTTACTGGGGGCTGCTTTCTTGTGGACAACAATCCTAGAAATACTACAGAAGCTAGACTGGTGGTGGACT

BE_scaffold146 ........................................................................................C...........

3510 3520 3530 3540 3550 3560 3570 3580 3590 3600

....|....|....|....|....|....|....|....|....|....|....|....|....|....|....|....|....|....|....|....|

WTE_scaffold35452 TCTCTCAGTTTTCAAGAAAACCCCATAAAGTATCCTGGCCTAATTTTTTTTGCCCGAACCTCAGGGCATTGTCCCGGTTACTGCCCTGAGGGTTGTCCTG

BE_scaffold146 ................G...................................................................................

3610 3620 3630 3640 3650 3660 3670 3680 3690 3700

....|....|....|....|....|....|....|....|....|....|....|....|....|....|....|....|....|....|....|....|

WTE_scaffold35452 CGCATTGTTGAATGTCTCTGTGGCTTTTTATCAATATTAATCCTAATGCTAGTTGTTTTGTTACTATTGGGGTACCTGGATTACAAGGGTTATACACCTG

BE_scaffold146 ....................................................................................................

3710 3720 3730 3740 3750 3760 3770 3780 3790 3800

....|....|....|....|....|....|....|....|....|....|....|....|....|....|....|....|....|....|....|....|

WTE_scaffold35452 TACATCCCCCTCTACTAGATGGGGATTGTCCAGGGAGCTGCATCTCAAATCAGCGCTGCAAGAAACCCCAGAGCTGTACAAACAGCAAATCTTGTTGCTG

BE_scaffold146 ....................................................................................................

3810 3820 3830 3840 3850 3860 3870 3880 3890 3900

....|....|....|....|....|....|....|....|....|....|....|....|....|....|....|....|....|....|....|....|

WTE_scaffold35452 TTACAAAAGTACAAATGAACCAACTCACGTTGCTGTTGCACCCCACAGTCACGGGCTTCAGGAAGATTCCTATGGGGATTGGCCTTAGCCCATTTCTCTT

BE_scaffold146 ...............A....................................................................................

3910 3920 3930 3940 3950 3960 3970 3980 3990 4000

....|....|....|....|....|....|....|....|....|....|....|....|....|....|....|....|....|....|....|....|

WTE_scaffold35452 GTGTTTATCGTCTGTATTACTTGTTCAGTATTACAGGAAGCAATTTGTCCACATTTTTACTTTTGCTTACATGGATGATTTGGTGGTGGGGTGTAAAAAC

BE_scaffold146 ....................................................................................................

4010 4020 4030 4040 4050 4060 4070 4080 4090 4100

....|....|....|....|....|....|....|....|....|....|....|....|....|....|....|....|....|....|....|....|

WTE_scaffold35452 CCTACTCATCTTCATGCAGCAGTCCATCATACTATTCATCATTTATTGTCACTGGGAGTAAGTATAAATAAAGATAAAAGAAAGTTTTCAGGAAAATATT

BE_scaffold146 ....................................................................................................

4110 4120 4130 4140 4150 4160 4170 4180 4190 4200

....|....|....|....|....|....|....|....|....|....|....|....|....|....|....|....|....|....|....|....|

WTE_scaffold35452 TGTATTTACCAACTGGGAAGTTTTGGAACTTTACCTAGCGATGATAAAATTTATAAATCAAGAGTCACTTTTCTCTCCATCCCATCACCAGAGCGTGGCC

BE_scaffold146 ....................................................................................................

4210 4220 4230 4240 4250 4260 4270 4280 4290 4300

....|....|....|....|....|....|....|....|....|....|....|....|....|....|....|....|....|....|....|....|

WTE_scaffold35452 TCGCAGCAGCGGGGACTGGATGAACCCCACTCCTAGAAGCGGGGTGCACGCCGGGCTGCGCCTGCCCAGCAGCGATCAACCTGGCAATGATTTCCCAAGG

BE_scaffold146 ....................................................................................................

4310 4320 4330 4340 4350 4360 4370 4380 4390 4400

....|....|....|....|....|....|....|....|....|....|....|....|....|....|....|....|....|....|....|....|

WTE_scaffold35452 CGGAGCAGCTTTTCGTTTCACAGATGGGCCAAAGCCATCCCCAGCACCCTTATCTGCCGCCGGCCCCGCGAGAAGGGAAACGACCTTGAGTCTATCAACN

BE_scaffold146 ....................................................................................................

4410 4420 4430 4440 4450 4460 4470 4480 4490 4500

....|....|....|....|....|....|....|....|....|....|....|....|....|....|....|....|....|....|....|....|

WTE_scaffold35452 NNNNNNNNNNNNNNNNNNNNNNNNNNNNNNNNNNNNNNNNNNNNNNNNNNNNNNNNNNNNNNNNNNNNNNNNNNNNNNNNNNNNNNNNNNNNNNNNNNNN

BE_scaffold146 ....................................................................................................

4510 4520 4530 4540 4550 4560 4570 4580 4590 4600

....|....|....|....|....|....|....|....|....|....|....|....|....|....|....|....|....|....|....|....|

WTE_scaffold35452 NNNNNNNNNNNNNNNNNNNNNNNNNNNNNNNNNNNNNNNNNNNNNNNNNNNNNNNNNNNNNNNNNNNNNNNNGCCCCCGCGGAGCCGGGCGTCCCCTGAC

BE_scaffold146 ........................................................................----------------------------

4610 4620 4630 4640 4650 4660 4670 4680 4690 4700

....|....|....|....|....|....|....|....|....|....|....|....|....|....|....|....|....|....|....|....|

WTE_scaffold35452 CGGCGTTGGTCTAAGGTCTTGTTTTGTCCCCACGTGCCACGCAACACCGAGGACAGCGTGTCTTGCTCAAACGAGCTACGGCGATAACAGCAGCTCTCCC

BE_scaffold146 ------------------------------------------------------------------------------------....G...........

4710 4720 4730 4740 4750 4760 4770 4780 4790 4800

....|....|....|....|....|....|....|....|....|....|....|....|....|....|....|....|....|....|....|....|

WTE_scaffold35452 ATGACCCGGCTACACAACCCAGTTTCCTCCCGTCATTCCTTGGGTGCNNNNNNNNNNNNNNNNNNNNNNNNNNNNNNNNNNNNNNNNNNNNNNNNNNNNN

BE_scaffold146 ....................................................................................................

4810 4820 4830 4840 4850 4860 4870 4880 4890 4900

....|....|....|....|....|....|....|....|....|....|....|....|....|....|....|....|....|....|....|....|

WTE_scaffold35452 NNNNNNNNNNNNNNNNNNNNNNNNNNNNNNNNNNNNNNNNNNNNNNNNNNNNNNNNNNNNNNNNNNNNNNNNNNNNNNNNNNNNNNNNNNNNNNNNNNNN

BE_scaffold146 ....................................................................................................

4910 4920 4930 4940 4950 4960 4970 4980 4990 5000

....|....|....|....|....|....|....|....|....|....|....|....|....|....|....|....|....|....|....|....|

WTE_scaffold35452 NNNNNNNNNNNNNNNNNNNNNNNNNNNNNNNNNNNNNNNGAGAACAGGGAAGCCAGACCCTGATGTCCCCTCCCGGCACTGGGTGGGGATCAAGCACTCA

BE_scaffold146 ....................................................................................................

5010 5020 5030 5040 5050 5060 5070 5080 5090 5100

....|....|....|....|....|....|....|....|....|....|....|....|....|....|....|....|....|....|....|....|

WTE_scaffold35452 GGGTTCATGGGAATGGTGAGGAAGGACACCCGAGTCCTCCTCTCACCAGCTAGCAGAGGGTGCAGGAGCCAGGAGGTGAAGCCCTGGGCTGAGTTACAGC

BE_scaffold146 ....................................................................................................

5110 5120 5130 5140 5150 5160 5170 5180 5190 5200

....|....|....|....|....|....|....|....|....|....|....|....|....|....|....|....|....|....|....|....|

WTE_scaffold35452 GACTTGAGTTAAAAGGCAAGAAAGGCTCTGAACTTTGAGGAAATCTGCNNNNNNNNNNNNNNNNNNNNNNNNNNNNNNNNNNNNNNNNNNNNNNNNNNNN

BE_scaffold146 ....................................................................................................

5210 5220 5230 5240 5250 5260 5270 5280 5290 5300

....|....|....|....|....|....|....|....|....|....|....|....|....|....|....|....|....|....|....|....|

WTE_scaffold35452 NNNNNNNNNNNNNNNNNNNNNNNNNNNNNNNNNNNNNNNNNNNNNNNNNNNNNNNNNNNNNNNNNNNNNNNNNNNNN-------AGTTGGTGCGGACCAG

BE_scaffold146 .............................................................................CCTCACC................

5310 5320 5330 5340 5350 5360 5370 5380 5390 5400

....|....|....|....|....|....|....|....|....|....|....|....|....|....|....|....|....|....|....|....|

WTE_scaffold35452 TCACCACTGGGTTGGGTCACAAGGGAGCCCCAGGAGCCACCCTGCATGGGTGACAGCCCAGCATCATGGCCCTGGTCACATCCCCGGCTGGGGTCCCACA

BE_scaffold146 .................................................................................................G..

5410 5420 5430 5440 5450 5460 5470 5480 5490 5500

....|....|....|....|....|....|....|....|....|....|....|....|....|....|....|....|....|....|....|....|

WTE_scaffold35452 GCACATCCCACCAAGGAGGTGGTGGCCAGAGAAGGGGGCCCAGACTCTGGCTGGGGGGCACTGAGCCTTGCTTTCCTCCTGCAGCAGCTTTTCCNNNNNN

BE_scaffold146 ....................................................................................................

5510 5520 5530 5540 5550 5560 5570 5580 5590 5600

....|....|....|....|....|....|....|....|....|....|....|....|....|....|....|....|....|....|....|....|

WTE_scaffold35452 NNNNNNNNNNNNNNNNNNNNNNNNNNNNNNNNNNNNNNNNNNNNNNNNNNNNNNNNNNNNNNNNNNNNNNNNNNNNNNNNNNNNNNNNNNNNNNNNNNNN

BE_scaffold146 ....................................................................................................

5610 5620 5630 5640 5650 5660 5670 5680 5690 5700

....|....|....|....|....|....|....|....|....|....|....|....|....|....|....|....|....|....|....|....|

WTE_scaffold35452 NNNNNNNNNNNNNNNNNNNNNNNNNNNNNNNNNNNNNNNNNNNNNNNNNNNNNNNNNNNNNNNNNNN----ACCTTGTGGGAGACACACGCGGACCAGGG

BE_scaffold146 ...................................................................GGAA....................T........

5710 5720 5730 5740 5750 5760 5770 5780 5790 5800

....|....|....|....|....|....|....|....|....|....|....|....|....|....|....|....|....|....|....|....|

WTE_scaffold35452 AAGGGGGACAGCCCCCCCACACACACACAGCTTGCCTTTCACCATCAGCAGGAGACAAAGGTCTGCAAAGCTGCTGGAGCAACCCGCAGCCTGGCCTCCC

BE_scaffold146 ................A...................................................................................

5810 5820

....|....|....|....|...

WTE_scaffold35452 CCGCTCCCCCACCGAAACCCAAA

BE_scaffold146 .......................

**(iii) *Aptenodytes forsteri* 2 and *Pygoscelis adeliae* 2**

10 20 30 40 50 60 70 80 90 100

....|....|....|....|....|....|....|....|....|....|....|....|....|....|....|....|....|....|....|....|

EP_scaffold155 CCTAATTTTAGGATTCTTCTTTCTATTTCCGTCTTTAAGAGTGTAATAAAAACATGAGATACACCCTGCATGCTTGTTTATTGCTGTCTTTCTTGCACCT

AP_scaffold14 ..........................................C.........................................................

110 120 130 140 150 160 170 180 190 200

....|....|....|....|....|....|....|....|....|....|....|....|....|....|....|....|....|....|....|....|

EP_scaffold155 GTAGGATACGTAGTATTCTGTGCTCATACAGACTTTGCTTTGCCAAGTCTTTGGAGCTAAAGTATTTCTCTTCAATACTGAATAAACTTTAAATGGCTGG

AP_scaffold14 ........T...................T.................A.........................-......A....................

210 220 230 240 250 260 270 280 290 300

....|....|....|....|....|....|....|....|....|....|....|....|....|....|....|....|....|....|....|....|

EP_scaffold155 CTCATTTCAGGTGTTTTGTTAGCTAGATGCTGTGTCAGCTGATACCATATTTGTGTAGGGAAGATACCCAGGAGTAATACAAGCACTAAGGTATGCAGCT

AP_scaffold14 ..........C..C...............................................-......................................

310 320 330 340 350 360 370 380 390 400

....|....|....|....|....|....|....|....|....|....|....|....|....|....|....|....|....|....|....|....|

EP_scaffold155 TGAAGTGTAATATATGTGTACCGGTTATGTTTTACTTTGTGTTTTCAGATTCCACTTCATCCTGTAATAGACAGTTTGGTACATGATGTTATTAACTTGG

AP_scaffold14 ....................A...............................................................................

410 420 430 440 450 460 470 480 490 500

....|....|....|....|....|....|....|....|....|....|....|....|....|....|....|....|....|....|....|....|

EP_scaffold155 CTTTCAAGCACTTTAAATACAAAGAAGGGTAAGGCTCTTCTTCACACAAAAATTCTATGAAAAAAATTATTTGCTGAATTTTTGAATTGTAGTGGTTTAA

AP_scaffold14 .....................................A.T..................T.........................................

510 520 530 540 550 560 570 580 590 600

....|....|....|....|....|....|....|....|....|....|....|....|....|....|....|....|....|....|....|....|

EP_scaffold155 GCAGGATGCTTTCTCAGCCAAGAATTCTTTCTTGTCTCTTGCTAGGTACCTTGGTCCTAACACTGGAAATATGCACATTGTTGCAGATTTGTATGCAGAA

AP_scaffold14 ....................................................................................................

610 620 630 640 650 660 670 680 690 700

....|....|....|....|....|....|....|....|....|....|....|....|....|....|....|....|....|....|....|....|

EP_scaffold155 GTAATAGGTGTTCTAGCTCAAGCAAAGTAAGTAAAATTGAATACCATATATAATGGAAATCCGAGAAGTTTTACTTGCTTAGAATATGAATGCTCTGATT

AP_scaffold14 .............................................................T....................C.........T.......

710 720 730 740 750 760 770 780 790 800

....|....|....|....|....|....|....|....|....|....|....|....|....|....|....|....|....|....|....|....|

EP_scaffold155 TTCTCGCTTTCTTAAAAGATAGTGTAGAAATAAATCATTTCAGCTCACTGGCCCTCTGTTCATCCAAGTGTTCTAGAGCATAGAACTCTAGGGAATCACA

AP_scaffold14 ...........................G...................................................G....................

810 820 830 840 850 860 870 880 890 900

....|....|....|....|....|....|....|....|....|....|....|....|....|....|....|....|....|....|....|....|

EP_scaffold155 GACTTTCTGCATCTAGCCCAGTGACACTGAATAAATTCAACTAGACTACAGCAGCATTATTTTTCTTATCTCAGCGATGACGACTGTGGAAAGCCCAAAG

AP_scaffold14 ...........................CA.............................................T.........................

910 920 930 940 950 960 970 980 990 1000

....|....|....|....|....|....|....|....|....|....|....|....|....|....|....|....|....|....|....|....|

EP_scaffold155 GGCTGAAGAGAGCTATTTTAATTAATCTTTTTTCTATATGCTATTTTTTCTCTAGCTGGGCAAACCTAAAAATGGCTATACTCCAACATAGCAACATTTT

AP_scaffold14 ....................................................................................................

1010 1020 1030 1040 1050 1060 1070 1080 1090 1100

....|....|....|....|....|....|....|....|....|....|....|....|....|....|....|....|....|....|....|....|

EP_scaffold155 AAATTTTTTCTGCAGTTATTTCCATTTAGAGCTTAATGCAACTGCTTCTGAATCCTTCATCCTGCACTTCTTAGTGCAGATGGCACAGTAAAGACAGAAA

AP_scaffold14 .........-.............G......A...........................................C.........................

1110 1120 1130 1140 1150 1160 1170 1180 1190 1200

....|....|....|....|....|....|....|....|....|....|....|....|....|....|....|....|....|....|....|....|

EP_scaffold155 ACACTTTGTTGAGTCCGGTCTTAAATTTTGAATCTGGAAGCAGACAGATTCAGAAACTCATGTACCCTATATAATGTCTCATCATATGCCTATCACACAA

AP_scaffold14 .......................................................G.........................---................

1210 1220 1230 1240 1250 1260 1270 1280 1290 1300

....|....|....|....|....|....|....|....|....|....|....|....|....|....|....|....|....|....|....|....|

EP_scaffold155 ACTAAGCAGAAGAGTGAAATTTACTGCGCCAACATGGATCCATGTCAGTGATCTCTGTCTCCTGACTTCTTGGTGTTACTGAGAGCGAGTCTGCTCTAAA

AP_scaffold14 ...........................C.....G......TG..........................................................

1310 1320 1330 1340 1350 1360 1370 1380 1390 1400

....|....|....|....|....|....|....|....|....|....|....|....|....|....|....|....|....|....|....|....|

EP_scaffold155 AGACCGATCAACTTAACCAAACTTATTTTTTTAGCTTGATGTAGGCAAACCAGTTTGAGTTCCTGGAAATCATATGACTTTCACAGACGTCAGCTAAGGC

AP_scaffold14 .....A.................C.....................................A...A.....................T............

1410 1420 1430 1440 1450 1460 1470 1480 1490 1500

....|....|....|....|....|....|....|....|....|....|....|....|....|....|....|....|....|....|....|....|

EP_scaffold155 CTTACCTGGGCTTCTAAATTGGCTGGGTCAAAGGCAAAAGGGGAAGTGACATCTGGATCCGAAGCTTGAAGTTGACTGTCAAAGACAAAACATACCTGTT

AP_scaffold14 ....................................................................................................

1510 1520 1530 1540 1550 1560 1570 1580 1590 1600

....|....|....|....|....|....|....|....|....|....|....|....|....|....|....|....|....|....|....|....|

EP_scaffold155 CTCCATAAATACTGTTTTTCTCCTCCAATTCTGAAGGGCCTTGCTAAGATGTTACTACCACTGAAGGACTTCTTCCTGCCAACCTGTTTTTTTCAACTCT

AP_scaffold14 .....................................................G.....G.......-------..........................

1610 1620 1630 1640 1650 1660 1670 1680 1690 1700

....|....|....|....|....|....|....|....|....|....|....|....|....|....|....|....|....|....|....|....|

EP_scaffold155 CCTGTGTTCCCTGTCAGAGGCGATAACA---GGTTGTTAGGTGCCACACCTGCCCATGAAAATGACATTCTTTTCATTTTGTCTCAAAAAGGACAGGAGA

AP_scaffold14 ............A...............ACA........C....T.......................................................

1710 1720 1730 1740 1750 1760 1770 1780 1790 1800

....|....|....|....|....|....|....|....|....|....|....|....|....|....|....|....|....|....|....|....|

EP_scaffold155 TCCTGTTGGATGATCTTCCTATATGTTATTTTTATGGACAGTGTGTCTCAAAAGACACCTCCAGTAGACTCTTGGGGGGG-TCAAAAATGGTCACAGACC

AP_scaffold14 ................................................................................G...................

1810 1820 1830 1840 1850 1860 1870 1880 1890 1900

....|....|....|....|....|....|....|....|....|....|....|....|....|....|....|....|....|....|....|....|

EP_scaffold155 ACCCCATCCCAAATTGTCGTAGTTAATGCTTAAGTGTTACTGTAATGGCTATTAGAAAAACCCTGAAGGGAGGAATAGCCTATCTGAATATTTTCATGGT

AP_scaffold14 ..................A..............................................................G........C.........

1910 1920 1930 1940 1950 1960 1970 1980 1990 2000

....|....|....|....|....|....|....|....|....|....|....|....|....|....|....|....|....|....|....|....|

EP_scaffold155 TATCATTTAAGCATTTTCTTTTGAAAACATTGCATGTACTGACAAGAAAACTATCGTTCATCTTGGATTTTTTAAAAACATGTGAGGGGATTATTCTAAT

AP_scaffold14 .........................................G............T.................G...........................

2010 2020 2030 2040 2050 2060 2070 2080 2090 2100

....|....|....|....|....|....|....|....|....|....|....|....|....|....|....|....|....|....|....|....|

EP_scaffold155 GATATTGTTAGCTCTTATGTAAAAGAGGGGTGTGGTAAGAAAATAATTGGGAAGATATGGTTATCCAAAGGGACTAAGATCAGGCTGTACACTTCGTTTT

AP_scaffold14 ..............................................................................................AA....

2110 2120 2130 2140 2150 2160 2170 2180 2190 2200

....|....|....|....|....|....|....|....|....|....|....|....|....|....|....|....|....|....|....|....|

EP_scaffold155 CTTTTCTGAGTTTATCAGAAGTGAAAGAAAACCAAACAAAAAGAAATCTCTTCACATACTGACTGAAATGGTATTTTACAACTGAGGTTGAATCTCCTTT

AP_scaffold14 ...............................G...............................................................T....

2210 2220 2230 2240 2250 2260 2270 2280 2290 2300

....|....|....|....|....|....|....|....|....|....|....|....|....|....|....|....|....|....|....|....|

EP_scaffold155 AGGTTCTTTCCCATTTATATACCTCACAATACATTTCAAATATGAATCTATACTTCTTTGTAACTAACCAATGTGAATTCATAGATTTCCTGCTGTAAAG

AP_scaffold14 .....T..........C.T......................G........C.................A........C......................

2310 2320 2330 2340 2350 2360 2370 2380 2390 2400

....|....|....|....|....|....|....|....|....|....|....|....|....|....|....|....|....|....|....|....|

EP_scaffold155 AAGAAATTCATGGCGGAGTTAAAAGAGTTACGGCATAAAGAACAAAGCCCATATGTAGTTCAAAGCATTATCAGTCTTATAATGGGAATGAAATTCTTTC

AP_scaffold14 ....................................................................................................

2410 2420 2430 2440 2450 2460 2470 2480 2490 2500

....|....|....|....|....|....|....|....|....|....|....|....|....|....|....|....|....|....|....|....|

EP_scaffold155 GCATTAAGATGTATCCTGTGGAGGACTTTGAAGCTTCTCTTCAGTTTATGCAGGTAAAATTCCTAAGTATATAACTGTGGTTCCCAGACCTTTTAGAATG

AP_scaffold14 ......................................................................G.............................

2510 2520 2530 2540 2550 2560 2570 2580 2590 2600

....|....|....|....|....|....|....|....|....|....|....|....|....|....|....|....|....|....|....|....|

EP_scaffold155 GGCTCCCCTTCATCTTTCCTGTAATACTCCAGGAGATTTTCCATATGTCATTCCCACATAAATGTATGTACAGCTACTTTCTGTTTGTTCTGAACCTCAG

AP_scaffold14 ...................................G................................................................

2610 2620 2630 2640 2650 2660 2670 2680 2690 2700

....|....|....|....|....|....|....|....|....|....|....|....|....|....|....|....|....|....|....|....|

EP_scaffold155 CACTCCAGTCTCCAGTCCCTGGAGTGTATCATTCCATACCAACTTTTCATCAGCCTTACAGCCCTAGTCCCTACATCTCACCCTCCAACAAAAAG--TTT

AP_scaffold14 ..............A...........................................T.C..................................AG...

2710 2720 2730 2740 2750 2760 2770 2780 2790 2800

....|....|....|....|....|....|....|....|....|....|....|....|....|....|....|....|....|....|....|....|

EP_scaffold155 TGGTTCTGTAGACCCCAAACCTTCCACCCTGCCATCCCACAGCTGTTCCTTGCCCTGTGTCCCTGCCCAGCTATGTCTCCCTAGCTGCTCACTGCCTCCA

AP_scaffold14 .............T...G..............................G..................T......................G.........

2810 2820 2830 2840 2850 2860 2870 2880 2890 2900

....|....|....|....|....|....|....|....|....|....|....|....|....|....|....|....|....|....|....|....|

EP_scaffold155 AAGTGAATCTTCCACCCTTGACATAGTTGCTAGAGAGGAGAGGAACTTCTGATAGAGAAAAACACAGAGGAAGAGACAAAGAATTGAGGCAAAAGCTGGA

AP_scaffold14 ...............A....................................................................................

2910 2920 2930 2940 2950 2960 2970 2980 2990 3000

....|....|....|....|....|....|....|....|....|....|....|....|....|....|....|....|....|....|....|....|

EP_scaffold155 TGGAATTTCCCTTGGATGTGTCTCCTTCCACCACACCTTCACCCCCCTTCCTGTAGGGTGCATTCTGTACCCTCCTGGTTTGAGAGCCACTAGTACAGAG

AP_scaffold14 ..............T..T...............................................C......C...........................

3010 3020 3030 3040 3050 3060 3070 3080 3090 3100

....|....|....|....|....|....|....|....|....|....|....|....|....|....|....|....|....|....|....|....|

EP_scaffold155 CATAGTTTGACTATCATGAATGCTTTAAATTAACACATTACATAATTTTGTAAAAGGAAAGAAAATCTTTTATGTTTAAGCATTTTTAAAAGGCCACATT

AP_scaffold14 ...................................G.......C....................................G...................

3110 3120 3130 3140 3150 3160 3170 3180 3190 3200

....|....|....|....|....|....|....|....|....|....|....|....|....|....|....|....|....|....|....|....|

EP_scaffold155 TCTTGTTGGTAATATACGATGGGTTCTGTTCCATCAGCAGGTTTATTTAGCTTAACCTTTTTATTTCCATTTTTTTCTTGGGATCAGTGTCATGGCTACG

AP_scaffold14 .................A...A..............................................G.................CCA.........T.

3210 3220 3230 3240 3250 3260 3270 3280 3290 3300

....|....|....|....|....|....|....|....|....|....|....|....|....|....|....|....|....|....|....|....|

EP_scaffold155 TGGTTTGTAGCCAGTCATCCAGTAAAATACACGTTTTATGTAAACCTCCATGTAAAGATCAGCTTATTGATGGTGTCAATGGAGTAAAAAGTCAATGTTA

AP_scaffold14 ..................G.................................................................................

3310 3320 3330 3340 3350 3360 3370 3380 3390 3400

....|....|....|....|....|....|....|....|....|....|....|....|....|....|....|....|....|....|....|....|

EP_scaffold155 GATGCCCACAAGTAAGGATTTGACATTGGAGTACAAATACACATGTGTACAGATACACAGAGCCATTTCTCTAAATCCTGAGCAGGAATCACATTCTGTT

AP_scaffold14 ..............................A.................G................................................A..

3410 3420 3430 3440 3450 3460 3470 3480 3490 3500

....|....|....|....|....|....|....|....|....|....|....|....|....|....|....|....|....|....|....|....|

EP_scaffold155 TCAATTTTGCAATGTGTTTTAAAGCATATTTAACAACATATTTAAAAGATATTTAACAACACAGGAACAGCTCATTTTTCTTCTTACCTTGCACTTCATT

AP_scaffold14 ............................................G...........................T...........................

3510 3520 3530 3540 3550 3560 3570 3580 3590 3600

....|....|....|....|....|....|....|....|....|....|....|....|....|....|....|....|....|....|....|....|

EP_scaffold155 ACATGAACACATCTTAGTATGTGTTGAGTTAATGTAAGTTATCCAGAGCTGGCAAGATATCAAAGTAGTGTGTGTTCAGTGCACATGACTGATATGAGTG

AP_scaffold14 .TG....................C...C............................G.................................A.........

3610 3620 3630 3640 3650 3660 3670 3680 3690 3700

....|....|....|....|....|....|....|....|....|....|....|....|....|....|....|....|....|....|....|....|

EP_scaffold155 AATGACTTTGTGGCATAGCTTTATGGCACAGCACTGCTGGGATCCAGTGTTCACTGGTATTGAAATCAGTGGTAAAGCTCCCGTAGAGTAGACTAAAAAT

AP_scaffold14 ....G..........................................C.....................................T............G.

3710 3720 3730 3740 3750 3760 3770 3780 3790 3800

....|....|....|....|....|....|....|....|....|....|....|....|....|....|....|....|....|....|....|....|

EP_scaffold155 TGGGATGCAAATTATCTCTACCTTGCTCATGACTACAGTATCTCAGCACCAAATTAATTCTTATATATACTACAATGCATCATAAGATGCACTGTGTATG

AP_scaffold14 .........................................A..........................................................

3810 3820 3830 3840 3850 3860 3870 3880 3890 3900

....|....|....|....|....|....|....|....|....|....|....|....|....|....|....|....|....|....|....|....|

EP_scaffold155 TAGTTTTGCTCAGTTTGGTGAAATACACTTA-CAGTTCGATTCTGTGGACTATCAGTCATTAGTTTGAGTATCTGCATGCAGTGCGTCAGCTACAAGCTC

AP_scaffold14 ...............................A..A.............G.C.......G.........................................

3910 3920 3930 3940 3950 3960 3970 3980 3990 4000

....|....|....|....|....|....|....|....|....|....|....|....|....|....|....|....|....|....|....|....|

EP_scaffold155 TGTCACACCAGCTCTGCTCTAGTAGAGAGAGACACTTTCAAAGAGTGAGCTAATCTCAGGAGCGAGCTCCAGAAAAGCCTGTCTTCCACCATGGAGTGCA

AP_scaffold14 ...........................................G..A..................A..................................

4010 4020 4030 4040 4050 4060 4070 4080 4090 4100

....|....|....|....|....|....|....|....|....|....|....|....|....|....|....|....|....|....|....|....|

EP_scaffold155 GAAGCAGCCCAGGGAGGCTACTGTCTTTGCATAGGGGCCTCAGCTGTGTAGCAGAAATGGCCAGAGTGACTGAAGCCATGTGGTGTAGAATCCTGATTGC

AP_scaffold14 ...............................C....................................................................

4110 4120 4130 4140 4150 4160 4170 4180 4190 4200

....|....|....|....|....|....|....|....|....|....|....|....|....|....|....|....|....|....|....|....|

EP_scaffold155 AGCCAGGAAAAATTATGCGTGGTCTAAAGTGTAAACTGCTCTGCACGCATGTGCAGTAAAAAAGGTCAGTGGGGAACATCCTTGTAATGTCAGTGGCTGT

AP_scaffold14 ..............................A..............T...C..................................................

4210 4220 4230 4240 4250 4260 4270 4280 4290 4300

....|....|....|....|....|....|....|....|....|....|....|....|....|....|....|....|....|....|....|....|

EP_scaffold155 TTGGGCCTTGGCGCCAGAGAGGAAGAAGTAACTGGAGCAATGTGATTTGTCGTCCTCAGAGAAGTTGTACAGAAACACAAGAGTGGGTGGATGCTTATCT

AP_scaffold14 ........................................C...........................................................

4310 4320 4330 4340 4350 4360 4370 4380 4390 4400

....|....|....|....|....|....|....|....|....|....|....|....|....|....|....|....|....|....|....|....|

EP_scaffold155 TCCCTCCCTCATTTCCTGAGCAGCGCAGCTGTGTAGGTAGTAGCTTGCTGCATGTTTAGGACAAAAGTACATTTTAACTTTCTCTCTCAATCCCCAGTTG

AP_scaffold14 ...........C........................A............C..............................................A...

4410 4420 4430 4440 4450 4460 4470 4480 4490 4500

....|....|....|....|....|....|....|....|....|....|....|....|....|....|....|....|....|....|....|....|

EP_scaffold155 CAGGTTGGAAATCGTGGTGGTTTGCACCACAGGTCTCTTCCCTGATACAGCACAGTTCTGTATTGACCCTTAATCTGCAATGGATCCTAGCAGTGAAGTT

AP_scaffold14 ............T..................................T.........................A..........................

4510 4520 4530 4540 4550 4560 4570 4580 4590 4600

....|....|....|....|....|....|....|....|....|....|....|....|....|....|....|....|....|....|....|....|

EP_scaffold155 TATCCAAAATATTTACAGTAAGATTGTTCCTTCCCTTGAGTATCTTACCAGAGAAGTATGTTCCTTTTCTGGCCTCCCAGAGCATTGCGTAAAGCAGTCC

AP_scaffold14 C......................................................................................T............

4610 4620 4630 4640 4650 4660 4670 4680 4690 4700

....|....|....|....|....|....|....|....|....|....|....|....|....|....|....|....|....|....|....|....|

EP_scaffold155 ATGCTTTTACTCTTGCTCCTGAAGGTATACACAGGGAAGGCATGCGGTCAGCCC--GAGTCCAACTCCCTTTCCATCCCCAGAACACCACTGGGCACAGC

AP_scaffold14 ..............C.............................A.........CT...A..............................CA........

4710 4720 4730 4740 4750 4760 4770 4780 4790 4800

....|....|....|....|....|....|....|....|....|....|....|....|....|....|....|....|....|....|....|....|

EP_scaffold155 AGGAATCAGTGGAGAGTTTGTGGATTGTCATGGCCAGAATAACAGATCCTCGATTTTGACTTATGTCCTGCTTTGTGCTTGCTGCAAAATGCGGACTTCG

AP_scaffold14 ......-----------.........A.......T...G.............................................................

4810 4820 4830 4840 4850 4860 4870 4880 4890 4900

....|....|....|....|....|....|....|....|....|....|....|....|....|....|....|....|....|....|....|....|

EP_scaffold155 ACGTTAATAGACAAACGTTTTCTGTGGGATTTGCAAGATAAAAATAGTCACTGACACTTCACATGCATTCAGCAAACATTTAGAGGTCTTTATTCCAGCT

AP_scaffold14 ...............T....................................................................................

4910 4920 4930 4940 4950 4960 4970 4980 4990 5000

....|....|....|....|....|....|....|....|....|....|....|....|....|....|....|....|....|....|....|....|

EP_scaffold155 GATAAGCAATTTATGTTTCTTGTTTTGTTTTGTTTTAGGAATGTGCACATTATTTCCTTGAAGTTAAAGACAAAGATATCAAGCATGCACTGGCAGGACT

AP_scaffold14 ....................................................................................................

5010 5020 5030 5040 5050 5060 5070 5080 5090 5100

....|....|....|....|....|....|....|....|....|....|....|....|....|....|....|....|....|....|....|....|

EP_scaffold155 GTTTGTTGAAATTCTTGTCCCTGTAGCTGCTGTGAGTTTATTTTTCAGGTTGTTTGCATAATTTCTTTAAATTAGATAAAACAGCTTGTGATGTAGTGGT

AP_scaffold14 ......................................C....................................................CA.....T.

5110 5120 5130 5140 5150 5160 5170 5180 5190 5200

....|....|....|....|....|....|....|....|....|....|....|....|....|....|....|....|....|....|....|....|

EP_scaffold155 TTATTAATCTGAAATTGTCTTTCAGGCTGTTAAAAATGAAGTGAATGTCCCCTGTCTGAGGAACTTTGTTGAAAGCCTGTATGATACAACACTTGAACTT

AP_scaffold14 ........A.......T...................................................................................

5210 5220 5230 5240 5250 5260 5270 5280 5290 5300

....|....|....|....|....|....|....|....|....|....|....|....|....|....|....|....|....|....|....|....|

EP_scaffold155 TCCTCACGAAAGAAGCACTCATTGGTTAGTAACCCTAAGAAGCAGAGGCTAAAACTCTTTGTCAGAAATATCTCTGAGCAAGAGTCTTGGGAAATTCCCA

AP_scaffold14 ....................................................................................................

5310 5320 5330 5340 5350 5360 5370 5380 5390 5400

....|....|....|....|....|....|....|....|....|....|....|....|....|....|....|....|....|....|....|....|

EP_scaffold155 GAACTGGTTGCAAAACTTTTTTCTGCATTAGGCTTTATAACCAATACATTTCTATTTGCAGGGAAGGTATTTGTGTTTGCAGAAAATAACAGTATTTTAT

AP_scaffold14 ....................................................................................................

5410 5420 5430 5440 5450 5460 5470 5480 5490 5500

....|....|....|....|....|....|....|....|....|....|....|....|....|....|....|....|....|....|....|....|

EP_scaffold155 TTTATTTTATTTTATTTTATTTTATTTTATTTTATTCAGAATATGATCAAAACCAGTCTGAATCCAACAGATGTTTTCAGCTTCTGGGTCTGTGATTATT

AP_scaffold14 .C.........-----------------------------------------------------------------------------------------

5510 5520 5530 5540 5550 5560 5570 5580 5590 5600

....|....|....|....|....|....|....|....|....|....|....|....|....|....|....|....|....|....|....|....|

EP_scaffold155 AAACGGTTAGGTTTTCTCATACTTGTTATGTGTAAACATTAGGAGGCGAGGAGAAAGAATAGAGTTCACTTCTTGTCAAGCGAGAACAAATGAGGAATGT

AP_scaffold14 ----------------------------------------------------------------------------------------------------

5610 5620 5630 5640 5650 5660 5670 5680 5690 5700

....|....|....|....|....|....|....|....|....|....|....|....|....|....|....|....|....|....|....|....|

EP_scaffold155 GGGAACAACTTTTTCACGGGGCTTAAATAATACATTGTAGGAACAAAAAAATGTATAACACATGCCCTTTTAAATCCGTAACTTGGCTACTGTGCAGCTC

AP_scaffold14 ----------------------------------------------------------------------------------------------------

5710 5720 5730 5740 5750 5760 5770 5780 5790 5800

....|....|....|....|....|....|....|....|....|....|....|....|....|....|....|....|....|....|....|....|

EP_scaffold155 CCTATTTCATTTTTTTCTTGTGAGATGAACAGTGGCACTGCAGTGTATTTTTGGAAGCATGAGTTGATCTGAATTAAATGAAGATCTGAGTCAGTTTGAC

AP_scaffold14 ----------------------------------------------------------------------------------------------------

5810 5820 5830 5840 5850 5860 5870 5880 5890 5900

....|....|....|....|....|....|....|....|....|....|....|....|....|....|....|....|....|....|....|....|

EP_scaffold155 GCTGGCTGCCTTAAGCAGTCTGTCTCTCCAACTGGGTCAGCGAACGTCAGCACTCCAGCATTAGCTAATCTGTTTTGAAGTTATTTTATTTTATTTTATT

AP_scaffold14 ----------------------------------------------------------------------------------------------------

5910 5920 5930 5940 5950 5960 5970 5980 5990 6000

....|....|....|....|....|....|....|....|....|....|....|....|....|....|....|....|....|....|....|....|

EP_scaffold155 TTATTTTATTTTATTCAGAATATGATCAAAACCAGTCTGAATCCAACAGATGTTTTCAGCTTCTGGGTCTGTGATTATTAAACGGTTAGGTTTTCTCATA

AP_scaffold14 ---------------.......CA.......T...........A........................................................

6010 6020 6030 6040 6050 6060 6070 6080 6090 6100

....|....|....|....|....|....|....|....|....|....|....|....|....|....|....|....|....|....|....|....|

EP_scaffold155 CTTGTTATGTGTAAACATTAGGAGGCGAGGAGAAAGAATAGAGTTCACTTCTTGTCAAGCGAGAACAAATGAGGAATGTGGGAACAACTTTTTCACGGGG

AP_scaffold14 ...................G..............................................................................A.

6110 6120 6130 6140 6150 6160 6170 6180 6190 6200

....|....|....|....|....|....|....|....|....|....|....|....|....|....|....|....|....|....|....|....|

EP_scaffold155 CTTAAATAATACATTGTAGGAACAAAAAAATGTATAACACATGCCCTTTTAAATCCGTAACTTGGCTACTGTGCAGCTCCCTATTTCATTTTTTTCTTGT

AP_scaffold14 .........C...................................A......................................................

6210 6220 6230 6240 6250 6260 6270 6280 6290 6300

....|....|....|....|....|....|....|....|....|....|....|....|....|....|....|....|....|....|....|....|

EP_scaffold155 GAGATGAACAGTGGCACTGCAGTGTATTTTTGGAAGCATGAGTTGATCTGAATTAAATGAAGATCTGAGTCAGTTTGACGCTGGCTGCCTTAAGCAGTCT

AP_scaffold14 ....................G...............................................................................

6310 6320 6330 6340 6350 6360 6370 6380 6390 6400

....|....|....|....|....|....|....|....|....|....|....|....|....|....|....|....|....|....|....|....|

EP_scaffold155 GTCTCTCCAACTGGGTCAGCGAACGTCAGCACTCCAGCATTAGCTAATCTGTTTTGAAGTCTTGGACCTTTTAAGTAAAGAACGTTGTCGTTCATCCTCC

AP_scaffold14 .................................G.................................................A................

6410 6420 6430 6440 6450 6460 6470 6480 6490 6500

....|....|....|....|....|....|....|....|....|....|....|....|....|....|....|....|....|....|....|....|

EP_scaffold155 CTCGTTCTATGGGAAAGTCAAAAAGAATTCCCTGTTCCCAGCGCTCTCGGCAAATGCTGGTCACTAGCGCTAGCGAATCTGCCGAGTGTGTTTGAGCTCA

AP_scaffold14 .........................GC....T................A.........................A.G.....T.G.AAGA----------

6510 6520 6530 6540 6550 6560 6570 6580 6590 6600

....|....|....|....|....|....|....|....|....|....|....|....|....|....|....|....|....|....|....|....|

EP_scaffold155 CACTCTGCAGAAGTGTGTTTGAGCTCTCTGCTGCCGTGGCACCAGTCCCCATGCAGTCCCAGGGAGAGTCAGACATCCCCTGGAGCACAGTCCTGATCCG

AP_scaffold14 ----......G..........................TA.............................................................

6610 6620 6630 6640 6650 6660 6670 6680 6690 6700

....|....|....|....|....|....|....|....|....|....|....|....|....|....|....|....|....|....|....|....|

EP_scaffold155 GTTTGAATTAGTGGGAGACTGGGGACGGATTTCTGATTTCACTCTGCTTGTGTTTGTGTGGCTCTATTCCACTCTGCCGAGTTAATAAAACATTGTTGAA

AP_scaffold14 .........................T..............................................................G...........

6710 6720 6730 6740 6750 6760 6770 6780 6790 6800

....|....|....|....|....|....|....|....|....|....|....|....|....|....|....|....|....|....|....|....|

EP_scaffold155 ACTGCAGCAGGGTAAGAGCTTTGCTGTACAGTTTTTACACTAAATAGAACTTCCCCAAACTGGTGGCCGTACTTGCCAAAGTCAATACTACATGCTTGAG

AP_scaffold14 ..............G................................................................................C....

6810 6820 6830 6840 6850 6860 6870 6880 6890 6900

....|....|....|....|....|....|....|....|....|....|....|....|....|....|....|....|....|....|....|....|

EP_scaffold155 AAAGGATTGCACAACTGGGGCTTTGCATTTGATTGCTTGTCTGCAGGTACCACACTGGAGGCTACATAGTTAAAAGTGGCAACATAGAATAGATTCTTGT

AP_scaffold14 .............G............................A..C................................A...A.................

6910 6920 6930 6940 6950 6960 6970 6980 6990 7000

....|....|....|....|....|....|....|....|....|....|....|....|....|....|....|....|....|....|....|....|

EP_scaffold155 GAGGGTGTGATTAGAAATACAAACATTGTGAGGAGGAGGAGGAGGGGAAAGGCAAACAGTTGGCAGAAATAGCTGCAGTGCATATTTTCAGGCTTCCAGG

AP_scaffold14 ........................G..........------...........................................................

7010 7020 7030 7040 7050 7060 7070 7080 7090 7100

....|....|....|....|....|....|....|....|....|....|....|....|....|....|....|....|....|....|....|....|

EP_scaffold155 AAGAAAGCACACTCTTTAGAGTCTGAAGAGGACCTTCTTTTCCAAGATCAGTGTTTTGTGAATCAGTACACTCACTGTACAGGCAGGATGGGGGCATTCA

AP_scaffold14 .....................................C...............................C..............C...............

7110 7120 7130 7140 7150 7160 7170 7180 7190 7200

....|....|....|....|....|....|....|....|....|....|....|....|....|....|....|....|....|....|....|....|

EP_scaffold155 TCTTGCCTCATTCTCTATCTAAAAGTGTTTCGTCTGAGCTAGTCGTTGAGGTTCTCTTGATAGTGAACGCAGACAATTAGGAAACTCCAGGAGATGATTT

AP_scaffold14 ...............................A....................................................................

7210 7220 7230 7240 7250 7260 7270 7280 7290 7300

....|....|....|....|....|....|....|....|....|....|....|....|....|....|....|....|....|....|....|....|

EP_scaffold155 CTGTCTTCCCTCCTAGGGGTCTTCTTAGGATGGGTTGTAACATTCTTGGGATGTTACTGAACTCTGTGATCCTGTCAAGTACTCAACTATTATAGACATA

AP_scaffold14 G..................................C...........................................................T....

7310 7320 7330 7340 7350 7360 7370 7380 7390 7400

....|....|....|....|....|....|....|....|....|....|....|....|....|....|....|....|....|....|....|....|

EP_scaffold155 ATGTTAATATGGTAAGAAGGTCTTCCGTGAGAAGACTTCAATTATCCTCTCCCATATGTATTTTGGACATGAAGATTATAAAAATAAAAATTAATGTACA

AP_scaffold14 ..................A.....................G...........................................................

7410 7420 7430 7440 7450 7460 7470 7480 7490 7500

....|....|....|....|....|....|....|....|....|....|....|....|....|....|....|....|....|....|....|....|

EP_scaffold155 TTTGCCATTTGTTCAAGAAACTAGGTCTAACCTAGCTATAGACAATTTATGTTATATATTGCAGGAACAGAAGTTGGGGTTTATGGTGTATTTATACTTT

AP_scaffold14 ..........................................................C.........................................

7510 7520 7530 7540 7550 7560 7570 7580 7590 7600

....|....|....|....|....|....|....|....|....|....|....|....|....|....|....|....|....|....|....|....|

EP_scaffold155 GATCTGTGACTGAAATTAGAATTTTAGGGTTAATTTTGAGAATCATTGGTCTTTAAGATAAAGTTACCTTATTTTTGACTAATAACTTTAAAATCCTGAT

AP_scaffold14 ..G....................C............................................................................

7610 7620 7630 7640 7650 7660 7670 7680 7690 7700

....|....|....|....|....|....|....|....|....|....|....|....|....|....|....|....|....|....|....|....|

EP_scaffold155 TGGGTATATTAGCATGACAACTGAAACACAAAAGTCTTGCTGAAATAGCAAAGTCAGTTTCAAAATTAATAGGTGCATTTTAGCTTATTCTTTTTAGTGT

AP_scaffold14 ....................................................................................................

7710 7720 7730 7740 7750 7760 7770 7780 7790 7800

....|....|....|....|....|....|....|....|....|....|....|....|....|....|....|....|....|....|....|....|

EP_scaffold155 AACATTTTCAAGATGAGTGTCAAAGTAAGTGTTGACAAAGAGACCAGGTTCCAGTATTTGAACTTCTACGTTCTGCCTTGCTTCTTCTTATTTTTCACTT

AP_scaffold14 ......................G..............................T..C.......................T...................

7810 7820 7830 7840 7850 7860 7870 7880 7890 7900

....|....|....|....|....|....|....|....|....|....|....|....|....|....|....|....|....|....|....|....|

EP_scaffold155 CACAAGATGTACCTACCAGCAGATGCAGGCATTGCCTGTATTTTAACACAATTCTTCAGGGTGTTTGACATGTCTCTCAGTAAACATTGTGACTGCAGAT

AP_scaffold14 ........................................................T...........................T...............

7910 7920 7930 7940 7950 7960 7970 7980 7990 8000

....|....|....|....|....|....|....|....|....|....|....|....|....|....|....|....|....|....|....|....|

EP_scaffold155 TTTGAGGAGTAATAGGCATCTGAGCATTCACAAGTCCCAACAATCTCTGTGGGTCCTGTTGTTATTATAATAAAGTTTAGTTATTTCCATGATACCATTT

AP_scaffold14 .......................................................T...................................C..T.....

8010 8020 8030 8040 8050 8060 8070 8080 8090 8100

....|....|....|....|....|....|....|....|....|....|....|....|....|....|....|....|....|....|....|....|

EP_scaffold155 CTCAGTTAACTGACAGAAATTAGCCCTGTGAGTCTGTAAAATTGTGGCCCATCTTTGGATTTATTATACTGCTATAAGTAGACATGTGGTTGTTTTTATT

AP_scaffold14 .....................................................................................A............G.

8110 8120 8130 8140 8150 8160 8170 8180 8190 8200

....|....|....|....|....|....|....|....|....|....|....|....|....|....|....|....|....|....|....|....|

EP_scaffold155 TCATCCATAAAACATATCTGTAACAGCTTTAGAGAAAGGTTCCTTTTAAATTGTACTACTTAACAATGTGTTTACGTTGGCTAACACGTATTGCACAAAA

AP_scaffold14 .........................A.................................................A.CA.....................

8210 8220 8230 8240 8250 8260 8270 8280 8290 8300

....|....|....|....|....|....|....|....|....|....|....|....|....|....|....|....|....|....|....|....|

EP_scaffold155 TGGAGGACTTAGAGCTTTAAGTTTACAGTAGCTGTATTAAGTTACTTTTATGTTGTGTAGACATGCAAATAACATGGTATCGACATCCATTTCCTGTTTA

AP_scaffold14 .......G.......................................C........................T...........................

8310 8320 8330 8340 8350 8360 8370 8380 8390 8400

....|....|....|....|....|....|....|....|....|....|....|....|....|....|....|....|....|....|....|....|

EP_scaffold155 GGCTTTGTACCCTTTGGTAACATGCCTGCTTTGTGTCAGTCAGAAGCAGTTCTTTTTAAACAGATGGCATATTTTCCTCAACAACTGCCTATCCAATCTC

AP_scaffold14 ....................................................................................................

8410 8420 8430 8440 8450 8460 8470 8480 8490 8500

....|....|....|....|....|....|....|....|....|....|....|....|....|....|....|....|....|....|....|....|

EP_scaffold155 AAGGTTAGTATCTTCTGGTTTTAATATTGTATTAAAACTACAGTATGTACTATTGATCTGCTGCAAATCCATTTAGGTAAATGTGCTGAATTATTGCTAT

AP_scaffold14 .......................................T............................................................

8510 8520 8530 8540 8550 8560 8570 8580 8590 8600

....|....|....|....|....|....|....|....|....|....|....|....|....|....|....|....|....|....|....|....|

EP_scaffold155 TATCTGATTCCATACATAATTTTATTCCCGACAGTACAAATTCTTTAGCTGTGCAGATTTTTTCAGTAACAAAAAAGTGTTCTTGTGCCTAGTTTGAATT

AP_scaffold14 ........................................................G........A.....................G............

8610 8620 8630 8640 8650 8660 8670 8680 8690 8700

....|....|....|....|....|....|....|....|....|....|....|....|....|....|....|....|....|....|....|....|

EP_scaffold155 TCTATGTTTCTCTAGCTCAAACCAAGCCTGAGTCAAACAGCTGTGAAACAGGCAAGATTGTTGATGAAAGTTATTAGCTCGAGTGACATACCCAAACTGA

AP_scaffold14 ...........T..................C.....................................................................

8710 8720 8730 8740 8750 8760 8770 8780 8790 8800

....|....|....|....|....|....|....|....|....|....|....|....|....|....|....|....|....|....|....|....|

EP_scaffold155 CCCAATATT------CTTTTTTCACAAAGGCATTCTCAAACAAAGCCCTTGTTATTACTTTATCAGAATGGAACTAGTTGCAGGACACTGATTCCATTGC

AP_scaffold14 .........AACTTG...............G.....A...............................................................

8810 8820 8830 8840 8850 8860 8870 8880 8890 8900

....|....|....|....|....|....|....|....|....|....|....|....|....|....|....|....|....|....|....|....|

EP_scaffold155 CTGTTTCACTCTGTTAAGTTGCATTAAAAAAACCTAAACTATTTTAAACTGTTTCCTCCACATATCCATTCTTATTCCTTTACCATTTGTTAGATCTTTG

AP_scaffold14 ...............................C....................................................................

8910 8920 8930 8940 8950 8960 8970 8980 8990 9000

....|....|....|....|....|....|....|....|....|....|....|....|....|....|....|....|....|....|....|....|

EP_scaffold155 AAGTTTAAGACTAACTGTCCACTGCAATCTGCTGTGGTTCCTGGCCAGCACATACTGTTCAATACACATATGCTAGTTATCATTACTACTCCTCCATGTT

AP_scaffold14 .............................A......................................................................

9010 9020 9030 9040 9050 9060 9070 9080 9090 9100

....|....|....|....|....|....|....|....|....|....|....|....|....|....|....|....|....|....|....|....|

EP_scaffold155 TCTTTTACTTGCGCAATAACTCATGTCCCATTCCACACTTGAAGTGGATCCATATGAAAGGATGGTCCTGTGCCTACCTGAAAAGTCCAATATAAGAAGC

AP_scaffold14 ....C...................................A.......................A...................................

9110 9120 9130 9140 9150 9160 9170 9180 9190 9200

....|....|....|....|....|....|....|....|....|....|....|....|....|....|....|....|....|....|....|....|

EP_scaffold155 ACAGTGAAGTGGTTAGCTCAAATGAAGGGCCTCCAAACTGCTGCTTGATGAACCAGTGGTTAGGAGGCTTCTGCAAACGGCACAGGTAGAATATGCAACC

AP_scaffold14 .............................................................G......................................

9210 9220 9230 9240 9250 9260 9270 9280 9290 9300

....|....|....|....|....|....|....|....|....|....|....|....|....|....|....|....|....|....|....|....|

EP_scaffold155 ACTTTCTCAGCTCAGAGGCAAACTGTGGAGGGAATCTCTGTTCTCCAAAAGTTGAATTCCCAAGTTTGTCGACAAGGTATATGTTACATCCTTTATTGCC

AP_scaffold14 ......................................................................A.............................

9310 9320 9330 9340 9350 9360 9370 9380 9390 9400

....|....|....|....|....|....|....|....|....|....|....|....|....|....|....|....|....|....|....|....|

EP_scaffold155 GTTGCTTTACTGCTGTACTTTATCTTTGTCTTAATGAAACAATTTGTATTGACTCTCTTCATATGGATCCTCCTGTTAGAATAGCTCTTCAGAATGTTCA

AP_scaffold14 .C.......T...............C......--......................T...........................................

9410 9420 9430 9440 9450 9460 9470 9480 9490 9500

....|....|....|....|....|....|....|....|....|....|....|....|....|....|....|....|....|....|....|....|

EP_scaffold155 TAGTCTGTCTGAAGATTTCTTTCCACCTGTACCTGAATTACTTACTTTTGCTAAAGATGTATTAGAACCTTATTTGCAAAGAGATTCAGAAAATAAACGT

AP_scaffold14 ............................................A.......................................................

9510 9520 9530 9540 9550 9560 9570 9580 9590 9600

....|....|....|....|....|....|....|....|....|....|....|....|....|....|....|....|....|....|....|....|

EP_scaffold155 TGTTGTTGCAACTAATTTTTTGGCTTTTTATGAGGTTGTACAAGCTTTAGAAAAGGCAGCTACAGCTGTAAGATCTCTGAGGAGCCAGCTGGGAATACAG

AP_scaffold14 .......................A............................................................................

9610 9620 9630 9640 9650 9660 9670 9680 9690 9700

....|....|....|....|....|....|....|....|....|....|....|....|....|....|....|....|....|....|....|....|

EP_scaffold155 GTTTTTCTGAAAATTAGGGAGATCCTGACAAATTATTTGAAACCATTACAGAAAATATAAGTAAGTTGCTCCTACCTTACCAATTGAAAATCTAATTGGA

AP_scaffold14 A..........................................T................----....................................

9710 9720 9730 9740 9750 9760 9770 9780 9790 9800

....|....|....|....|....|....|....|....|....|....|....|....|....|....|....|....|....|....|....|....|

EP_scaffold155 ATTCACCTTGCCACCTATAATTTGTTGACATATACTTCTATTCACAGGGCTCATAGATTAATGTGGTGGCACATTCAATCTTTAGTTTGGGGAGGAGGTA

AP_scaffold14 ..............................................................................................A.....

9810 9820 9830 9840 9850 9860 9870 9880 9890 9900

....|....|....|....|....|....|....|....|....|....|....|....|....|....|....|....|....|....|....|....|

EP_scaffold155 AGGTAGCTGAATACATGGCTAAATTAAGAACGTGGTTGGCCACTCCTTCTCAGTACAGAGGAGGAGATGCCCCAACCATAAAGCAATCACTTCACCAGTC

AP_scaffold14 ..............................................................A.....................................

9910 9920 9930 9940 9950 9960 9970 9980 9990 10000

....|....|....|....|....|....|....|....|....|....|....|....|....|....|....|....|....|....|....|....|

EP_scaffold155 AG--TAACACCTCAAAGCCCAAACTAAACTCATGCAGCTGGAAGAACAGCTAGGTCAACAAGAAGGAGAAATCCTTCCATTAGAAGGAGAGTTGTGCACA

AP_scaffold14 ..AG.GG.......................................G......A..............................................

10010 10020 10030 10040 10050 10060 10070 10080 10090 10100

....|....|....|....|....|....|....|....|....|....|....|....|....|....|....|....|....|....|....|....|

EP_scaffold155 CCAGGGCAGTATATGGGACTCCATGATCTTGTGTGGGTTCCCATGAACCACATAATGGATCAGGTTTCTCAAGAGCCTCCACACCCCATAAAGCACAATC

AP_scaffold14 .......................C...............................C...............G............................

10110 10120 10130 10140 10150 10160 10170 10180 10190 10200

....|....|....|....|....|....|....|....|....|....|....|....|....|....|....|....|....|....|....|....|

EP_scaffold155 TCTTTCCCAAAACCATTAAGGTCAAGCATACACTGGGAAAGCTAAAAGGTTTATCTTCTAATCATATCTCTGATTTTAATATAAACTGGCAAATCTCTGA

AP_scaffold14 ...............................G....................................................................

10210 10220 10230 10240 10250 10260 10270 10280 10290 10300

....|....|....|....|....|....|....|....|....|....|....|....|....|....|....|....|....|....|....|....|

EP_scaffold155 TCTTACAGAAACAAACTTTTCTCTTGAATTAATAAAGGAGACTCCTGCCAGGACATGGAAATACTTGACCCAGCCAAATTCTGGCCAAAGGGCATTTCAT

AP_scaffold14 ......G...............................................................T.............................

10310 10320 10330 10340 10350 10360 10370 10380 10390 10400

....|....|....|....|....|....|....|....|....|....|....|....|....|....|....|....|....|....|....|....|

EP_scaffold155 ACAATCCTGTTGAAAGCGGTGTAAAAGTTAATAAGTAACCTGGTTTTGAAATGGAACATCTATTGTTAACTAAACTTCATTTACAAAAAACTATTCAAGG

AP_scaffold14 ..T...................G....................................T........................................

10410 10420 10430 10440 10450 10460 10470 10480 10490 10500

....|....|....|....|....|....|....|....|....|....|....|....|....|....|....|....|....|....|....|....|

EP_scaffold155 CAGGAATTCTATATAGGAGAGAGTCAAAGCACTGTGTTTCATTTAGAGGAAAAGTTTTTCAGTGGGAGCAACAGTTCCTTGTCTCCAGACTACATGGGAC

AP_scaffold14 .................G...................................A..............................................

10510 10520 10530 10540 10550 10560 10570 10580 10590 10600

....|....|....|....|....|....|....|....|....|....|....|....|....|....|....|....|....|....|....|....|

EP_scaffold155 AAGGAGAAAGCTCCAGTACAATTTGAGCTGAGGGTGCAGAACTTAATTTACAAAGAATGGGTCAAACAATGCCCATCTCGGGAACAAATCCATGGGTACG

AP_scaffold14 ...............................................................................A...................A

10610 10620 10630 10640 10650 10660 10670 10680 10690 10700

....|....|....|....|....|....|....|....|....|....|....|....|....|....|....|....|....|....|....|....|

EP_scaffold155 ACACAATATCCCTTTGGCCTGAATAGGCCAGACCTAATGCCATATTATTGGAAAAGAGAAATGGAGGAATTAAAGAAAATGGGTGTCACTCCTGGCAGTC

AP_scaffold14 ...................................................................................C...G............

10710 10720 10730 10740 10750 10760 10770 10780 10790 10800

....|....|....|....|....|....|....|....|....|....|....|....|....|....|....|....|....|....|....|....|

EP_scaffold155 CGGTTGCTCCTTTGCCAATCACAGAAGAAGAGGCGGAGGCATTGAAAAAGAAATTTATAGAAGAGCAACAACAACAAATAAAAGCCAGCAGCACTTATC-

AP_scaffold14 ...........C......................A.....................C..........................................T

10810 10820 10830 10840 10850 10860 10870 10880 10890 10900

....|....|....|....|....|....|....|....|....|....|....|....|....|....|....|....|....|....|....|....|

EP_scaffold155 ---AAGAAACAGAGCAGATCCTGGCAGAGCTCTGTCACTGACTGCTCCTGTGTTCAAAAGGCCCACATCAACTTCGACAAATTCTTCGGCACCAACAGTG

AP_scaffold14 ATC.................................G.............C....................................A............

10910 10920 10930 10940 10950 10960 10970 10980 10990 11000

....|....|....|....|....|....|....|....|....|....|....|....|....|....|....|....|....|....|....|....|

EP_scaffold155 AGCTGGGCACTGCCAGCAGTGGTAAAGGAACCACACCCAGTGCCAATACCTCCTGTT-------------------------------------------

AP_scaffold14 ..T..............................T.G....................CTCTTGAGTGAAGAATCATAGAATCATAGAATCATTAAGGTTGG

11010 11020 11030 11040 11050 11060 11070 11080 11090 11100

....|....|....|....|....|....|....|....|....|....|....|....|....|....|....|....|....|....|....|....|

EP_scaffold155 ----------------------------------------------------------------------------------------------------

AP_scaffold14 AAGAGACCTCCAAGATCATCGAGTCCAACCGTCAACCCAACACCACCATGCCCACTAAACCATGTCCCTAAGCGCCTCATCTACACATCTTTTAAATACC

11110 11120 11130 11140 11150 11160 11170 11180 11190 11200

....|....|....|....|....|....|....|....|....|....|....|....|....|....|....|....|....|....|....|....|

EP_scaffold155 ----GGGAAAGTGA--------------------------------------------------------------------------------------

AP_scaffold14 TCCA....TG....CTCAACCACTTCCCTGGGCAGCCTCTTCCAATGTTTAACCACTCTTTCAGTAAAGAAATTTTTCCTCACATCCAATCTAAACCTCC

11210 11220 11230 11240 11250 11260 11270 11280 11290 11300

....|....|....|....|....|....|....|....|....|....|....|....|....|....|....|....|....|....|....|....|

EP_scaffold155 ----------------------------------------------------------------------------------------------------

AP_scaffold14 CCTGGCGCAACTTGAGGCCATTTCCTCTCATCCTTTCGCCAGTTACTTGGGAGAAGAGACCAACACCCACCTCGCTACAACCTCCTTTCAGGTAGTTGTA

11310 11320 11330 11340 11350 11360 11370 11380 11390 11400

....|....|....|....|....|....|....|....|....|....|....|....|....|....|....|....|....|....|....|....|

EP_scaffold155 ----------------------------------------------------------------------------------------------------

AP_scaffold14 GAGAGCGATGAGGTCTCCCCTCAGCCTCCTTTTCTCCAGGCTAAACAACCCCAGCTCCCTCAGCCGCTCCTCATAAGACTTGTTCTCCAGACCCCTCACC

11410 11420 11430 11440 11450 11460 11470 11480 11490 11500

....|....|....|....|....|....|....|....|....|....|....|....|....|....|....|....|....|....|....|....|

EP_scaffold155 ----------------------------------------------------------------------------------------------------

AP_scaffold14 AGCCTCATTGCCCTTCTCTGGACACGCTCCAACACCTCAACGTCCTTCTTGTAGTGAGGGGCCCAAAACTGAACACAGTATTTGAGGTGCGGCCTCACCA

11510 11520 11530 11540 11550 11560 11570 11580 11590 11600

....|....|....|....|....|....|....|....|....|....|....|....|....|....|....|....|....|....|....|....|

EP_scaffold155 ----------------------------------------------------------------------------------------------------

AP_scaffold14 GTGCCGAGTACAGGGGCACGATCACTTCCCTGCTCCTGCTGGCCACACTGTTTCTGATACAGGCCAGGATGCCTTTGGCCTTCTTGGCCGCCTGGGCACA

11610 11620 11630 11640 11650 11660 11670 11680 11690 11700

....|....|....|....|....|....|....|....|....|....|....|....|....|....|....|....|....|....|....|....|

EP_scaffold155 -----------------------------------------AGAAGATGTCAGATATATTAGGCCAAATACTCCTGGTCCTAATAGGATTACTGGCTGGG

AP_scaffold14 TTGCTGGCTCACGTTCAGCCGGCTGTCGACCAACACCCCCA...................................................CA......

11710 11720 11730 11740 11750 11760 11770 11780 11790 11800

....|....|....|....|....|....|....|....|....|....|....|....|....|....|....|....|....|....|....|....|

EP_scaffold155 TTTTTCTTGTTGACAAAAATCCTTGAAATACTAAAGAGGCTCAATTGGTGGTGGACTTCTCTCAGTTTTCCAAAGGGAGACATGCTATGTGCTTTCCAAA

AP_scaffold14 .......................C..........................................................C.................

11810 11820 11830 11840 11850 11860 11870 11880 11890 11900

....|....|....|....|....|....|....|....|....|....|....|....|....|....|....|....|....|....|....|....|

EP_scaffold155 GTACTGGGCACCCAACCACCATGCACTTGCACAGATCTTGCCCATGGACATGCCCATGATTTCCCTGGATGTTTCTCAGGCTTTTTAACATCTTCCTCTC

AP_scaffold14 .......C............................................................................................

11910 11920 11930 11940 11950 11960 11970 11980 11990 12000

....|....|....|....|....|....|....|....|....|....|....|....|....|....|....|....|....|....|....|....|

EP_scaffold155 AATCCTGCTTCTGCCGTGCAGCTTGCTGTTTCTGATGGAAAACTGGTCTACTATTTTCGGAAAGCTCCAATGATATCAGTCTCAGCCCTTTTCTCCTCCA

AP_scaffold14 ...............A....................................G....T..........................................

12010 12020 12030 12040 12050 12060 12070 12080 12090 12100

....|....|....|....|....|....|....|....|....|....|....|....|....|....|....|....|....|....|....|....|

EP_scaffold155 TCTCTTCTCAGCTGCCCTCGCAGCTGAACAATTTTGTCATTGGAATATTTGGACTTTTGCTTATAGGGATGACTTCCTCCTCTGCCACACAAGCTCTTGT

AP_scaffold14 ..........A.......T..............................................................G..................

12110 12120 12130 12140 12150 12160 12170 12180 12190 12200

....|....|....|....|....|....|....|....|....|....|....|....|....|....|....|....|....|....|....|....|

EP_scaffold155 TACCTTAACTCAGTTAGCCACAGTGTCTGCTGTTCTCTTGAAAGCTTTGGGGTAAGAATAAACTTTGATAAACATACTCCATCACCTGTTGAAGAAATCA

AP_scaffold14 ............T..........G.A..........................................................................

12210 12220 12230 12240 12250 12260 12270 12280 12290 12300

....|....|....|....|....|....|....|....|....|....|....|....|....|....|....|....|....|....|....|....|

EP_scaffold155 AATTCTTAGGACTGAAATTTACAAGGGCTGACATGATGATACCTGATGAAAAATGGATTGAAAGTAAACAGGTTATTAAACAAATCGATTGTAACAAATG

AP_scaffold14 ..........................A..................................C.......................G..............

12310 12320 12330 12340 12350 12360 12370 12380 12390 12400

....|....|....|....|....|....|....|....|....|....|....|....|....|....|....|....|....|....|....|....|

EP_scaffold155 TTATGATTTTAAAATCCTGCAAAGATTAATAGGCCACGTAAATTTTGTCATTCCTTTTACTACTTACTCTAATCATGGGCTACAACCATTGTATGCTGCT

AP_scaffold14 ....................................T........................................T......................

12410 12420 12430 12440 12450 12460 12470 12480 12490 12500

....|....|....|....|....|....|....|....|....|....|....|....|....|....|....|....|....|....|....|....|

EP_scaffold155 ATAATAAACAAAAAGGATTTTCAATTTTCTGTTCCATATAAAGCTTTATTGTATAAAATGTGTTGTCAAGGAGTTAAATGGAAATTGCAACCCAAGGACA

AP_scaffold14 .......................G............................................................................

12510 12520 12530 12540 12550 12560 12570 12580 12590 12600

....|....|....|....|....|....|....|....|....|....|....|....|....|....|....|....|....|....|....|....|

EP_scaffold155 GTGTTCCTATTCTTAGGCTTGTGACAGATGCCACGTTGAAAGTTGGAGCCATATCCCATATCATCGGCGGGTTGTCTTCCTTCCATTTTGCAGGACCTAG

AP_scaffold14 ............C......................................................T................G...............

12610 12620 12630 12640 12650 12660 12670 12680 12690 12700

....|....|....|....|....|....|....|....|....|....|....|....|....|....|....|....|....|....|....|....|

EP_scaffold155 ACAAATACACATTCAAGAATTATTGATGGCATCGGTTGCAGTTTCTTTGATCAAACCGCGGCCTTTGATCTGTGACTCAACATTTGTCTGCAGACAAAAA

AP_scaffold14 ....................................................................................................

12710 12720 12730 12740 12750 12760 12770 12780 12790 12800

....|....|....|....|....|....|....|....|....|....|....|....|....|....|....|....|....|....|....|....|

EP_scaffold155 TTCAGCTCTTTGCAGTGGAGATTTGCACTGTTGGGCCAGGCAAATCTTATCTCGGGTCAAAGTGTACTGGTCTCTCTCAAAGTTTAACCCAGTGGATGGC

AP_scaffold14 ......................................................................A.............................

12810 12820 12830 12840 12850 12860 12870 12880 12890 12900

....|....|....|....|....|....|....|....|....|....|....|....|....|....|....|....|....|....|....|....|

EP_scaffold155 CCAACACATGGGAGACCATCTGATTGGACTGCATATACTTGCACCCTACTCAGAAAGCCCTTAAAACTGCCACAGAAACAGAAAACTGCATATGACCAAA

AP_scaffold14 .........................................................................................G..........

12910 12920 12930 12940 12950 12960 12970 12980 12990 13000

....|....|....|....|....|....|....|....|....|....|....|....|....|....|....|....|....|....|....|....|

EP_scaffold155 GAGACCCGAAGACAATCTTATACTAGTTCAAAAGGTTCCAAAAATAAACAACAATCCAGACAGATAATCCAATTCAGTCCAGCTGATCCCAGATGCCATG

AP_scaffold14 ..................................T...............G.................................................

13010 13020 13030 13040 13050 13060 13070 13080 13090 13100

....|....|....|....|....|....|....|....|....|....|....|....|....|....|....|....|....|....|....|....|

EP_scaffold155 TTAATTAGGTCAGCTTGAGCATCCACAGATTTTTGTCCTTTCACTACTGAATACGTGCAGTGCATTTATTTAT--CTGCTGTGTTCTTCATACCATGTCT

AP_scaffold14 ..............C.A....................................T...................AT.........................

13110 13120 13130 13140 13150 13160 13170 13180 13190 13200

....|....|....|....|....|....|....|....|....|....|....|....|....|....|....|....|....|....|....|....|

EP_scaffold155 CCACCATAGGGC------TTTTGAATTGACAGAATTAACTGAAGATA-GGTTTTCTTTTCAGAGTCAAATTCAGGTGATTTTCAAATAGAGATTTTAAAT

AP_scaffold14 ............AACAGC.................C...........A............G.......................................

13210 13220 13230 13240 13250 13260 13270 13280 13290 13300

....|....|....|....|....|....|....|....|....|....|....|....|....|....|....|....|....|....|....|....|

EP_scaffold155 TGTAACATGTATCTACTTTAACTAACTTGATACTTGGGTTTTTCAACAACTTAAGTAGTACCAGGAGAGGTAGGTCAGATGCATAAGCATCCTGCAGCTA

AP_scaffold14 .....................................................................A..A..........A.......T........

13310 13320 13330 13340 13350 13360 13370 13380 13390 13400

....|....|....|....|....|....|....|....|....|....|....|....|....|....|....|....|....|....|....|....|

EP_scaffold155 CAGGAATGGGGTACCGGGCAGCTGTGAGGCTGTATCTTTTTGTAGTGTCATTACGAAAACTCTGAACCTTGACATCCATTAAGTGCAGAACTTCCTTTCT

AP_scaffold14 ............G.T........A.............................T..............................................

13410 13420 13430 13440 13450 13460 13470 13480 13490 13500

....|....|....|....|....|....|....|....|....|....|....|....|....|....|....|....|....|....|....|....|

EP_scaffold155 AATTTGTGCCTGAAGTTCATTCTTACGTTATATGTTTCTCTAGAATAAGGACCCCAAAATGGCTCGAGTTGCACTGGAATCTCTGTACAGACTACTGTGG

AP_scaffold14 .................T..................................................................................

13510 13520 13530 13540 13550 13560 13570 13580 13590 13600

....|....|....|....|....|....|....|....|....|....|....|....|....|....|....|....|....|....|....|....|

EP_scaffold155 GTTTACATGATCAGAATTAAATGTGAAAGCAACACAGCTACCCAAAGGTAAGATGCTGTATTTGTAATGATAATCAAGCCTTGTAGAGTAGATGTTGCCA

AP_scaffold14 ....................................................................................................

13610 13620 13630 13640 13650 13660 13670 13680 13690 13700

....|....|....|....|....|....|....|....|....|....|....|....|....|....|....|....|....|....|....|....|

EP_scaffold155 CTGAAGAGCTTTTATAGTTCTCCTGAATTCCTTTGAATTGAACCTATTCCTGCTGTGGAACAAAAAACTCAACTGTACACAACAAAATATTCCCTTTTGT

AP_scaffold14 ...........................C........................A....---T....C..A...............................

13710 13720 13730 13740 13750 13760 13770 13780 13790 13800

....|....|....|....|....|....|....|....|....|....|....|....|....|....|....|....|....|....|....|....|

EP_scaffold155 GGCACCATCAGTTATTCTCCCACCTATTAAAATTACCATATAAAGTGTTTCTGCTTTTGGAAGAACTAAAAATCACTATTTGCCTGGTTAAAGTTAGAAA

AP_scaffold14 .................................C..TG........C..............................C......................

13810 13820 13830 13840 13850 13860 13870 13880 13890 13900

....|....|....|....|....|....|....|....|....|....|....|....|....|....|....|....|....|....|....|....|

EP_scaffold155 TATTTTTGTAGTGGCATGT---------------------------CATTGACTGGTTTATTGTTACTTGTAATGTAGTAATGAGATAAAATAATTAGAT

AP_scaffold14 ...................TGTTTGACTGGTCATTGTTATGGCTGT....................T..........A...........T.......CG.

13910 13920 13930 13940 13950 13960 13970 13980 13990 14000

....|....|....|....|....|....|....|....|....|....|....|....|....|....|....|....|....|....|....|....|

EP_scaffold155 TGAATTTGTAACAAAGGATCAGAACAAGATACCCCATTACATTCTCCTTTCAAAGGAAATACATTCTAGTCTTTCTAAATGGAGTAGGATTTCCAACTTA

AP_scaffold14 ....................................................................................................

14010 14020 14030 14040 14050 14060 14070 14080 14090 14100

....|....|....|....|....|....|....|....|....|....|....|....|....|....|....|....|....|....|....|....|

EP_scaffold155 -GTTTCATAGTAAGCGAAGGTCTACAAGGTCCAGATATGTCTAATGCTTTTTTTCCTTCTTTTACAGTCGACTTATTACTATTGTCACAACACTTTTCCC

AP_scaffold14 C..................................A....................C..........................A................

14110 14120 14130 14140 14150 14160 14170 14180 14190 14200

....|....|....|....|....|....|....|....|....|....|....|....|....|....|....|....|....|....|....|....|

EP_scaffold155 AAAAGGGTCCCGTGGTGTTGTGCCAAGAGATATGCCTCTAAACATCTTTGTGAAGATAATACAGTTTATTGCACAGGTATGGGAGAACCATGCATATGTC

AP_scaffold14 ....................................................................................................

14210 14220 14230 14240 14250 14260 14270 14280 14290 14300

....|....|....|....|....|....|....|....|....|....|....|....|....|....|....|....|....|....|....|....|

EP_scaffold155 TTTCCAATTAATGAATAACTTGTTTTCCCAATAACAAATTAACAAATAAGTAAACATCTATTGTCTGAATTTTTTGCAACCCCATTGTCAGTATCTTTCA

AP_scaffold14 .....................C..............................................................................

14310 14320 14330 14340 14350 14360 14370 14380 14390 14400

....|....|....|....|....|....|....|....|....|....|....|....|....|....|....|....|....|....|....|....|

EP_scaffold155 TCTAGGCAATGATAACATTATGGATTGAGCTGGGGCAGACTTCTTTTGAACAGGTTTTGATTAACTTCATTCCACTGAAAATAAATAAAAATGTTTCCAC

AP_scaffold14 .....................A........C.....................------------------------------------------------

14410 14420 14430 14440 14450 14460 14470 14480 14490 14500

....|....|....|....|....|....|....|....|....|....|....|....|....|....|....|....|....|....|....|....|

EP_scaffold155 TTCAACTCAAATGTATTTTTTGTCAGTTTTTGGAATTGCCAGCAAGTCAGTAAACATCTATTGTCTGAATTTTTTGCAACCCCATTGTCAGTATCTTTCA

AP_scaffold14 ----------------------------------------------------------------------------------------------------

14510 14520 14530 14540 14550 14560 14570 14580 14590 14600

....|....|....|....|....|....|....|....|....|....|....|....|....|....|....|....|....|....|....|....|

EP_scaffold155 TCTAGGCAATGATAACATTATGGATTGAGCTGGGGCAGACTTCTTTTGAACAGGTTTTGATTAACTTCATTCCACTGAAAATAAATAAAAATGTTTCCAC

AP_scaffold14 -------------------------------------------------................G..............................T...

14610 14620 14630 14640 14650 14660 14670 14680 14690 14700

....|....|....|....|....|....|....|....|....|....|....|....|....|....|....|....|....|....|....|....|

EP_scaffold155 TTCAACTCAAATGTATTTTTTGTCAGTTTTTGGAATTGCCAGCAAGCCAGAAAATGATTTGTTGACAGGCTTTGACTGTCAATAAAATTAAAAATAATAA

AP_scaffold14 ..............................................T.....................................................

14710 14720 14730 14740 14750 14760 14770 14780 14790 14800

....|....|....|....|....|....|....|....|....|....|....|....|....|....|....|....|....|....|....|....|

EP_scaffold155 GCCATATTTTGTCCTGATACTGAATCATTCCTACTATATAGGACATTGAGTTGCACTGCACAG--TAAGTCAGAGGAAAACCAAGTTCTTCATGTAGCTG

AP_scaffold14 ...............................................................AG...................................

14810 14820 14830 14840 14850 14860 14870 14880 14890 14900

....|....|....|....|....|....|....|....|....|....|....|....|....|....|....|....|....|....|....|....|

EP_scaffold155 ACACATTTAGTTTTCATCAGTTCAAATGGGAATTGCTAAAATAAACTGACCTTCAGTGCTGGAAGTTTCATCTTTTTTATTA----GATTCAGAAAACAT

AP_scaffold14 .........................................................................C..C.....CTTA..............

14910 14920 14930 14940 14950 14960 14970 14980 14990 15000

....|....|....|....|....|....|....|....|....|....|....|....|....|....|....|....|....|....|....|....|

EP_scaffold155 TCCCATTGAGAAAGGCACTTAAGCACATGCTTAAAGTCTCTGAATAGGAATGAATTAAACAGATGCTTAAAATCTTCCCTGAACTGCGCCATCTGTTAAC

AP_scaffold14 ..............................................A.......................................T.............

15010 15020 15030 15040 15050 15060 15070 15080 15090 15100

....|....|....|....|....|....|....|....|....|....|....|....|....|....|....|....|....|....|....|....|

EP_scaffold155 AAGCTCCTTTTATATAAAA-TACTATGTTAGTACCATAATTACCACAGTTTAAATGAAATAATTAAAAGCTTTATTTCCAAAATGGATCATTTTCTATTT

AP_scaffold14 ...................A................................................................................

15110 15120 15130 15140 15150 15160 15170 15180 15190 15200

....|....|....|....|....|....|....|....|....|....|....|....|....|....|....|....|....|....|....|....|

EP_scaffold155 AAATGCTATAAATTTTTTAATTTGTCAACATACAGCAGTGTTGAAAGCTTAGATCTGATAATATGTTCTGCATACTTCTGAAAAATCTGTTTTTCCGTAC

AP_scaffold14 ......................................C.............................................-..........T....

15210 15220 15230 15240 15250 15260 15270 15280 15290 15300

....|....|....|....|....|....|....|....|....|....|....|....|....|....|....|....|....|....|....|....|

EP_scaffold155 GTTTAAATAAATCCTGATTAAACTGGTTTTCAACATTCTGTTGCCTTAAAGAAAATAAGTAGCAGAAGTGAGCAGCTTAATGATTTGCAATATTTTTACT

AP_scaffold14 .........C..........................................................................................

15310 15320 15330 15340 15350 15360 15370 15380 15390 15400

....|....|....|....|....|....|....|....|....|....|....|....|....|....|....|....|....|....|....|....|

EP_scaffold155 ATTTTAGGAACGTTTAGATTTTGCAATGAAAGAAATTATCTTTGACTTCCTTTGTGTTGGAAAACCAGCAAAAGCTTTCAGTCTCAACCCTGAGGTATGA

AP_scaffold14 ....................................................................................................

15410 15420 15430 15440 15450 15460 15470 15480 15490 15500

....|....|....|....|....|....|....|....|....|....|....|....|....|....|....|....|....|....|....|....|

EP_scaffold155 TGTGCATCTGCATTTTTTTGAGTTAGTATTTATTGTACTGTGCTACATTTACTTTCTGGTTACATTCTAAGCATGCTTTCACCTTTGTCCAGTTTTCAGG

AP_scaffold14 .....G..............................G...............................................................

15510 15520 15530 15540 15550 15560 15570 15580 15590 15600

....|....|....|....|....|....|....|....|....|....|....|....|....|....|....|....|....|....|....|....|

EP_scaffold155 CCAAATCTTGAACTTGATTTATTGGTAAAAGACATCTGTGGACTTTGATCTGTGAAATGTCACACTGTTTATGCCTATCTCATGTTTCCTCAGTGAAGAT

AP_scaffold14 ...........................G.........A.......................................................C......

15610 15620 15630 15640 15650 15660 15670 15680 15690 15700

....|....|....|....|....|....|....|....|....|....|....|....|....|....|....|....|....|....|....|....|

EP_scaffold155 TATCAAACATGATGTAGTGCAAATGTATTAAAAAAAAAGAAAAAGATAAATAAAACTATTGATCACAGTATCTCCTTATCACTCTGCTGTTGTTATATGC

AP_scaffold14 ............................------....C..............................................C..............

15710 15720 15730 15740 15750 15760 15770 15780 15790 15800

....|....|....|....|....|....|....|....|....|....|....|....|....|....|....|....|....|....|....|....|

EP_scaffold155 AATGCCTTAAACTATTTTAATATAAAAACAAATTTTCTGTAACAATTTCACTGTTTTTCAAAACAGTTTTACTGAGAAAGAGTACCTGAACCAAGAAGAA

AP_scaffold14 ............................T..............................................A........................

15810 15820 15830 15840 15850 15860 15870 15880 15890 15900

....|....|....|....|....|....|....|....|....|....|....|....|....|....|....|....|....|....|....|....|

EP_scaffold155 TTGAAGATGAGAAAAAAGAAAAAACTCTAATAAAGTTTTACCAATCTGACTAGTAAAACTCTAGGAGCATTAGTGTCATGAGTCAGCTTCCTAGTGCTTT

AP_scaffold14 ..........C...............--.T..G........................................................T......T...

15910 15920 15930 15940 15950 15960 15970 15980 15990 16000

....|....|....|....|....|....|....|....|....|....|....|....|....|....|....|....|....|....|....|....|

EP_scaffold155 CCCTAGAGAAAACAATCAGAAATTTGAGAAAACAATCTGAAATTTGAAAAACTAAAAAAAAAAACCAAAACCAAAAACACCAACCCCCCCCAAAATAAAA

AP_scaffold14 ........................--------------------.........G.......--.......A.....C.....-.................

16010 16020 16030 16040 16050 16060 16070 16080 16090 16100

....|....|....|....|....|....|....|....|....|....|....|....|....|....|....|....|....|....|....|....|

EP_scaffold155 ATAAGTGGTAAAATAATGAAAAATTCTCTTGAAAATAATGTATTTGCTTATAATTTTGAAAACATTTTCATTTGGAAATTAAGAATAAACACATGAAAAA

AP_scaffold14 ....................................................................................................

16110 16120 16130 16140 16150 16160 16170 16180 16190 16200

....|....|....|....|....|....|....|....|....|....|....|....|....|....|....|....|....|....|....|....|

EP_scaffold155 TAAAAAGCATTTGTGATCATCTCATAAAATATGTCCATATTAATCTTGCAGTATAAACAGTCATATCTATTCACTAAAATGTTAAAATAGAATGAGTTAC

AP_scaffold14 ....................................................................................................

16210 16220 16230 16240 16250 16260 16270 16280 16290 16300

....|....|....|....|....|....|....|....|....|....|....|....|....|....|....|....|....|....|....|....|

EP_scaffold155 CTTACAAAAATTATATTTGATACAAAAAAATATTTGGAAAAGTAGACTAAACTTTTTACTGTCAAAAGCAGCAAAAGTGAAAGGCAAGCTTTACTTTGAG

AP_scaffold14 ....................................................................................................

16310 16320 16330 16340 16350 16360 16370 16380 16390 16400

....|....|....|....|....|....|....|....|....|....|....|....|....|....|....|....|....|....|....|....|

EP_scaffold155 CACAGCTTTCATCTTTCTTCAGCAGAGTAAAGAAAAATGAAACACAGTTCTTGAGCAGGCAGTCCCCACCACATCCTGGGTCAGTCACCTGCCGTATTGA

AP_scaffold14 ..............................................A........................T..T.........................

16410 16420 16430 16440 16450 16460 16470 16480 16490 16500

....|....|....|....|....|....|....|....|....|....|....|....|....|....|....|....|....|....|....|....|

EP_scaffold155 CTGAGGAAGGTCCTGCAAAGGGCATTGTGTATTAACATTGCTTGAAGCCGAGCTTATAGGGAATGGACATAGTCCTTAGCTTCTTGATGAATCAACATCT

AP_scaffold14 ..................................G...C................G...........................C.C..............

16510 16520 16530 16540 16550 16560 16570 16580 16590 16600

....|....|....|....|....|....|....|....|....|....|....|....|....|....|....|....|....|....|....|....|

EP_scaffold155 GAAATTTTACTGTTTCTCAACAGCAATGTAAGTCCAAAAACAATGAAATAATGGGGCTACTTTTTGATGTGCATGGACTTCAGCGTTCTAAAAAAATACC

AP_scaffold14 ....................................................................................A...............

16610 16620 16630 16640 16650 16660 16670 16680 16690 16700

....|....|....|....|....|....|....|....|....|....|....|....|....|....|....|....|....|....|....|....|

EP_scaffold155 CATAATCTATGTAAATGCGTCTCTGGGAGACTGTAAGTAGGACAGCCTCTGAGTTTTTGGAATACGTTCCTGGGAAATTGAAACTTTCTGGTCTTTCAGT

AP_scaffold14 ..................A...........T.....................................................................

16710 16720 16730 16740 16750 16760 16770 16780 16790 16800

....|....|....|....|....|....|....|....|....|....|....|....|....|....|....|....|....|....|....|....|

EP_scaffold155 CGTTGCAATGCAAAGCCTGTAAGCTTTCCTCTGGAGAATTCCCTGAAGACATTCAGGTCACTTAGCCTCATTTTCCCGGTGTTTTCACATAGCAGTTTTG

AP_scaffold14 .......................T...............................................C....T.......................

16810 16820 16830 16840 16850 16860 16870 16880 16890 16900

....|....|....|....|....|....|....|....|....|....|....|....|....|....|....|....|....|....|....|....|

EP_scaffold155 GCACTGAGCACCATTCCAGACTTTTCACACAGTATGGACAACCCTGGGGCCTTTCAAGACAGTTATTGATCTTTTTTCATAATGACCACATAGGACATTT

AP_scaffold14 ..G................T.................T..............................................................

16910 16920 16930 16940 16950 16960 16970 16980 16990 17000

....|....|....|....|....|....|....|....|....|....|....|....|....|....|....|....|....|....|....|....|

EP_scaffold155 GTGTTTAACTGAAGGTCTGGTTAAGATGTATTACTAAACTCAAGGTCAAATGCACATGTTTTGTCCTGAAGATAAAGAGATAATGTACTAGTGAAAATAC

AP_scaffold14 .................CA...................................TG..................................A.........

17010 17020 17030 17040 17050 17060 17070 17080 17090 17100

....|....|....|....|....|....|....|....|....|....|....|....|....|....|....|....|....|....|....|....|

EP_scaffold155 CTAGAAATAAAGTTTTGAGAGGGGAGAGCGCTGCTCGTAGTACAGATAAGAATTCACAATTTATTTCATGGGCATTTTTTGTAAAGCATATCAGACTAGT

AP_scaffold14 T.....................A......A...........G...........................................A..............

17110 17120 17130 17140 17150 17160 17170 17180 17190 17200

....|....|....|....|....|....|....|....|....|....|....|....|....|....|....|....|....|....|....|....|

EP_scaffold155 CAATGTAGTGGAGTTACAGAGGAAGTGCTGGCAAGTACATAGCCAAAATTTCAAAGTGATTGAAGAATATGAGTAGAATATGATTATTTGATTACGATAC

AP_scaffold14 .....C.......A......................................................................................

17210 17220 17230 17240 17250 17260 17270 17280 17290 17300

....|....|....|....|....|....|....|....|....|....|....|....|....|....|....|....|....|....|....|....|

EP_scaffold155 TCATTCATCTTCTGAACTTTACAGAGAATGAATATTGGTCTGAGAGCTTTCTTGGTAATTGCTGATAGCTTGCAGCAAAAAGATGGTGAACCTCCAATGC

AP_scaffold14 ....................................................................................................

17310 17320 17330 17340 17350 17360 17370 17380 17390 17400

....|....|....|....|....|....|....|....|....|....|....|....|....|....|....|....|....|....|....|....|

EP_scaffold155 CAGTGACTGGAGCTGTCCTTCCCTCTGGAAACACTCTCAGAGTGAAGAAAACATATTTGAGCAAAACTCTTACAGAGGAGGAAGCTAAAATGATAGGTCA

AP_scaffold14 ....................................................G...............................................

17410 17420 17430 17440 17450 17460 17470 17480 17490 17500

....|....|....|....|....|....|....|....|....|....|....|....|....|....|....|....|....|....|....|....|

EP_scaffold155 GTAAGAGAATGGACAATACAGAAACTTGAATGAGAATGAATGTGAAAAAGACTTCAGTACCTGAGCAGAATAATTGACTATTGTTAAGTATTGAATTGAT

AP_scaffold14 .....................................T............T........................T................-.......

17510 17520 17530 17540 17550 17560 17570 17580 17590 17600

....|....|....|....|....|....|....|....|....|....|....|....|....|....|....|....|....|....|....|....|

EP_scaffold155 TTCCTTATTGTAAACATTTACTTTATTGTGCATTCTAATGAAATATATGGTTTAGTTTGACAAATACTGTTTTTTTAAATTACTTTCAGGAAAAGTCTTC

AP_scaffold14 .......C.............................................................G..............................

17610 17620 17630 17640 17650 17660 17670 17680 17690 17700

....|....|....|....|....|....|....|....|....|....|....|....|....|....|....|....|....|....|....|....|

EP_scaffold155 AGTGGTGCTACACAAGTGGTAGCAACCCTGTGTTCTGGCGGCGTTAAGTACAGAGATAGTTAAGAATTAGCTTCTTTCGGTGCTGAATAGCTGTGTTTAT

AP_scaffold14 ..........................................A.................................C.A...T.................

17710 17720 17730 17740 17750 17760 17770 17780 17790 17800

....|....|....|....|....|....|....|....|....|....|....|....|....|....|....|....|....|....|....|....|

EP_scaffold155 TCATGGATATTCATCTTATTAGAAACAGTGCCTGTGCTCGGAAAATGTACCAGCTTTGAAAACCAGCAGATTCCTTTGATATCCTCATTTTAATTGCAAT

AP_scaffold14 ....................C..................A.....................................C......................

17810 17820 17830 17840 17850 17860 17870 17880 17890 17900

....|....|....|....|....|....|....|....|....|....|....|....|....|....|....|....|....|....|....|....|

EP_scaffold155 ATTTCACACCAAGTGAAACTTTCAAAATGGAAAAAACTTTTAAATGTAAAATAAAGCTGAACTGAAGTGCCTAAGTTAAAAGCACCGTTACCTTGGGCTT

AP_scaffold14 ........................................A.............................T.............................

17910 17920 17930 17940 17950 17960 17970 17980 17990 18000

....|....|....|....|....|....|....|....|....|....|....|....|....|....|....|....|....|....|....|....|

EP_scaffold155 CCTGCGTAGTCAATGTAGAGAGAGAATCCTGTGCTGTAGGAAAAGAGCTTCTGTATGATGCCTTGAAGGCACCTGTTTTTGCTGGCAAAAAAGAAAATTT

AP_scaffold14 ...AA...............................C................................TG.............................

18010 18020 18030 18040 18050 18060 18070 18080 18090 18100

....|....|....|....|....|....|....|....|....|....|....|....|....|....|....|....|....|....|....|....|

EP_scaffold155 GGCTACTCACCTTAGCACTTCATTAAGTCCCCAAAGTTACGTGAATGAAACAAAGT-TACATATTTCTACACCAGCGACAAAGACCTGTGTTGTACTGTT

AP_scaffold14 .......................................T................C..........................G........A.......

18110 18120 18130 18140 18150 18160 18170 18180 18190 18200

....|....|....|....|....|....|....|....|....|....|....|....|....|....|....|....|....|....|....|....|

EP_scaffold155 GCTAGATCATTGTAGTAATATCAAGAAAATAAGCAATGTAAGACCCACTGCTGTTGAAATGTACAACAGACATAGTATTTAGTGCTGTCACTAATTCAGT

AP_scaffold14 ...C...............................G...................C............................................

18210 18220 18230 18240 18250 18260 18270 18280 18290 18300

....|....|....|....|....|....|....|....|....|....|....|....|....|....|....|....|....|....|....|....|

EP_scaffold155 TCATATGCTATCACCTCATGAGG--AAAACATTTTTCTTAATTCATGCAGCACAGCATGAGTAAGCAGCTTTCTTTTTCAAAATATATGTATCTATAAAT

AP_scaffold14 ...........---........CTC.TG.AT......................................................C..............

18310 18320 18330 18340 18350 18360 18370 18380 18390 18400

....|....|....|....|....|....|....|....|....|....|....|....|....|....|....|....|....|....|....|....|

EP_scaffold155 ATCCCAAATTACACAAAAATGCCTATTATTGCAGGTATGTCATTATATTATTCTCAAGTAAGAAAAGCTGTGGACAACATACTCAGACATCTTGACAAGG

AP_scaffold14 ...........T........................................................................................

18410 18420 18430 18440 18450 18460 18470 18480 18490 18500

....|....|....|....|....|....|....|....|....|....|....|....|....|....|....|....|....|....|....|....|

EP_scaffold155 AAGTGGGGCGGTGCATGATGCTAACCAACATACAGATGCTTAACAAAGAACCTGAAGACATGATTACGTGAGTGCTAAA---CAGTAAATCCCATAAAAT

AP_scaffold14 ...............................................................................GAA..................

18510 18520 18530 18540 18550 18560 18570 18580 18590 18600

....|....|....|....|....|....|....|....|....|....|....|....|....|....|....|....|....|....|....|....|

EP_scaffold155 ATTGAGATGTAATTTCAGAACTTTTTTTCTGTCTTATCATGAGTCATCAGAGAAACAGCAAGAAATAGGAACATTTAGCTTAATTAGTATATACTTTGAA

AP_scaffold14 .................................................................................G..................

18610 18620 18630 18640 18650 18660 18670 18680 18690 18700

....|....|....|....|....|....|....|....|....|....|....|....|....|....|....|....|....|....|....|....|

EP_scaffold155 AAACTGTTTATGACAAGTTGATTGTACTTCTTTGCATGTTGCAGTGTTTGGAATTGAACTAATGCTCTCAATGTAGCCAAAATCATGTGTGAATCCTATG

AP_scaffold14 ...................................C................................................................

18710 18720 18730 18740 18750 18760 18770 18780 18790 18800

....|....|....|....|....|....|....|....|....|....|....|....|....|....|....|....|....|....|....|....|

EP_scaffold155 AATGTTTAGATAGTAGAAGTTGAAAGCCCAGAGAGGAAAACCTGTCAGCCTATGCTCAGAACACTGTTTCAGGGTATATTTGCATCACTGAACTTCAAAA

AP_scaffold14 .........G...................................................T.............G........................

18810 18820 18830 18840 18850 18860 18870 18880 18890 18900

....|....|....|....|....|....|....|....|....|....|....|....|....|....|....|....|....|....|....|....|

EP_scaffold155 TGAATGCAACAAATGCTGTAAATCCCTATTAATAATAATCTTTCAAAATTACTATTCATGCCTGTAATCAAAATCAAATCTTAATTATAAACCCAAGTCA

AP_scaffold14 ..............T.....................................................................................

18910 18920 18930 18940 18950 18960 18970 18980 18990 19000

....|....|....|....|....|....|....|....|....|....|....|....|....|....|....|....|....|....|....|....|

EP_scaffold155 TTTTGTGACAAGAGTCTTGGATTTAAGCAGTACCCCACTGGAACAGAATCAAACCAGTATGTCTCAAGCAGTGTTGCACAGCAGCATTTTACACTCTCAT

AP_scaffold14 .....................................................T.....C........................................

19010 19020 19030 19040 19050 19060 19070 19080 19090 19100

....|....|....|....|....|....|....|....|....|....|....|....|....|....|....|....|....|....|....|....|

EP_scaffold155 CCAAAATTATTAATGAGGGAAGTTTCCTGGAGACAGTTGTGGTGAGGTGGACGTCTAGTAGACTTTAGAGAC-ACAGAGACATAATTTGACACATGCAGA

AP_scaffold14 .................................G......A...............................C...........................

19110 19120 19130 19140 19150 19160 19170 19180 19190 19200

....|....|....|....|....|....|....|....|....|....|....|....|....|....|....|....|....|....|....|....|

EP_scaffold155 GCACAGATTGTAGAGATCCCAACTGTAAGGAGACCTTGCATATCTTTCCTGTTACAGAGGTGGACATGGTAGTTATGCTAGAAGAGAAACAACCCCTGGA

AP_scaffold14 ....G.........................................C..................C..................G...............

19210 19220 19230 19240 19250 19260 19270 19280 19290 19300

....|....|....|....|....|....|....|....|....|....|....|....|....|....|....|....|....|....|....|....|

EP_scaffold155 AAAAAGTTTGAAGTGTAATGAAAAGGACATTGGTCTCTTTGAGATTGTTTTTTTCATATACTGTCCAAAAAAAAAAAAAAGATAACTTGCTAAAAATACC

AP_scaffold14 ...........G.................................-..................T--.....T.........C...............G.

19310 19320 19330 19340 19350 19360 19370 19380 19390 19400

....|....|....|....|....|....|....|....|....|....|....|....|....|....|....|....|....|....|....|....|

EP_scaffold155 CTGAGTGGTAGATAGACTGCAATGACTGATCAAATTCAGCTACCTTTTACAGCTGATACAATCTTTATGTTTTAATGACATTCCTCTGCCCTTGTTGCTG

AP_scaffold14 T.....................................A..........................C...........T.C....................

19410 19420 19430 19440 19450 19460 19470 19480 19490 19500

....|....|....|....|....|....|....|....|....|....|....|....|....|....|....|....|....|....|....|....|

EP_scaffold155 TGACTGCGTAGATGAGATTGTAAGCTCTTTGTGTCTTTAGCATTGTCTTACTTTATTTATATTAAATGCCAACATTACTATCCGATAATAATTCGTATTT

AP_scaffold14 ......T.................A...............................................T.....................--....

19510 19520 19530 19540 19550 19560 19570 19580 19590 19600

....|....|....|....|....|....|....|....|....|....|....|....|....|....|....|....|....|....|....|....|

EP_scaffold155 TTCTTATAGTACTACCCAGTATACTGCTCAGTACTCAACAGGCATTTTCATAACCATTTTGGGTATCCAGGCTTTTGCAAGAGGGAAAGTGAGTTTTGAA

AP_scaffold14 ...................................T.-..............................................................

19610 19620 19630 19640 19650 19660 19670 19680 19690 19700

....|....|....|....|....|....|....|....|....|....|....|....|....|....|....|....|....|....|....|....|

EP_scaffold155 ACTCACATTTTATTCCCAAAAGATAATTGCATAGTAAAACCTTGAGCACTTAGATAAGAAGTGTACTTAACCTTAGCTGTAAATGTCCTTTCTATGGTTT

AP_scaffold14 .....................................................G..............................................

19710 19720 19730 19740 19750 19760 19770 19780 19790 19800

....|....|....|....|....|....|....|....|....|....|....|....|....|....|....|....|....|....|....|....|

EP_scaffold155 CTCAGCATCACCGAAGTTGTTTATTTTTAGAGGCAGGGAAAGAATAGACTTTAAAGTGTTTTAAATTTCAGGAAGTCTTATGGTTTGGTAGTAACTTTTA

AP_scaffold14 ....A.......A.......................T...........T.............G.....................................

19810 19820 19830 19840 19850 19860 19870 19880 19890 19900

....|....|....|....|....|....|....|....|....|....|....|....|....|....|....|....|....|....|....|....|

EP_scaffold155 TCAGAGACATAAAGATTTTAAGGTTGGAAGATCTAGACATGTAAGTTACTGTATTTTTGTGTTGTACTTTATATCCAGATGAGCTGTAACTTAATATATA

AP_scaffold14 .........................................C...........................................A..............

19910 19920 19930 19940 19950 19960 19970 19980 19990 20000

....|....|....|....|....|....|....|....|....|....|....|....|....|....|....|....|....|....|....|....|

EP_scaffold155 CATTTGAAAGGAAAAACACATTAGAACACATTTTTACATGTTCTTCCAAGGAAAAAAGATATCTGGGATGCTTAATTATCAGTTGATCTAAAGTCATGTG

AP_scaffold14 .........................................................A..................................A.......

20010 20020 20030 20040 20050 20060 20070 20080 20090 20100

....|....|....|....|....|....|....|....|....|....|....|....|....|....|....|....|....|....|....|....|

EP_scaffold155 AACTCATGAAAAACATCATTAGCTGTTCCTATCAGCATTTAAAATGGCCCTTTTAAAACCTGCATTGTGCGTAACTGGAAAAGCCAATATATTGTTTTTA

AP_scaffold14 ..........G.........................................................................................

20110 20120 20130 20140 20150 20160 20170 20180 20190 20200

....|....|....|....|....|....|....|....|....|....|....|....|....|....|....|....|....|....|....|....|

EP_scaffold155 TGTAATTGCTTATCATTTTATTCACAGAACTTCATGCTTCATTAGAGTTACATATTTATTTTGAGTTCTTATGGGCTTTAATACATAGATACAACACTAC

AP_scaffold14 .........................................C.........................................T................

20210 20220 20230 20240 20250 20260 20270 20280 20290 20300

....|....|....|....|....|....|....|....|....|....|....|....|....|....|....|....|....|....|....|....|

EP_scaffold155 ATTTATCTATATTTTAAAGTTATGGCTTTTGTCATATTTACATATAGTTAATGTTATTGTAAAAACTGAGTATCATAATGCTATATTTAATAACAAAATA

AP_scaffold14 .............................................................T.......A..............T...............

20310 20320 20330 20340 20350 20360 20370 20380 20390 20400

....|....|....|....|....|....|....|....|....|....|....|....|....|....|....|....|....|....|....|....|

EP_scaffold155 AGAATCAGAGGGAAGTAGCTAGGCAATGAATAGTCATAACATACTAAAGTATACGGATTCTGGAATTTTCTTTGGCAGTGTTAGACATGCTTCTGTATTT

AP_scaffold14 ...................................G..................A.............................................

20410 20420 20430 20440 20450 20460 20470 20480 20490 20500

....|....|....|....|....|....|....|....|....|....|....|....|....|....|....|....|....|....|....|....|

EP_scaffold155 AATGACTGATCCTTTAAGCATTTATTGTTGAAGTAGTAAATGCCACTCTGAAGAGTGTTACTAAAATCCTGACTAAGGAGCGTCCTTTGCATCCTCCACT

AP_scaffold14 ..............C..................................................................A.T................

20510 20520 20530 20540 20550 20560 20570 20580 20590 20600

....|....|....|....|....|....|....|....|....|....|....|....|....|....|....|....|....|....|....|....|

EP_scaffold155 GGGATATGAATTTCAATATGGGTTATAATTACTTTCCAATGTCTAGTCAGAATAGGGCATCCATTTTTCTAATATACAAACCTGTTCTTGCAAGTACTTC

AP_scaffold14 ....................................................................................................

20610 20620 20630 20640 20650 20660 20670 20680 20690 20700

....|....|....|....|....|....|....|....|....|....|....|....|....|....|....|....|....|....|....|....|

EP_scaffold155 AAAGATTTTTGCTGGCAATACCTAACTAAAATCTGATACTGCTGGTATAGAAACACAAGCTTCCAGGTGAGGACAGCTGTCGGTGTGTTCATCCAGAGCT

AP_scaffold14 .........................G...........G..........G...............................T...............C...

20710 20720 20730 20740 20750 20760 20770 20780 20790 20800

....|....|....|....|....|....|....|....|....|....|....|....|....|....|....|....|....|....|....|....|

EP_scaffold155 CATCCTTTTGGATCCTGAGTCAGCCAGAGATGACTGCGGAGCCCTGTGAGGCCCCCCTTGCCAGATACTTCCCATGCTGTGCCTCGTAGCTCAGATAAGT

AP_scaffold14 .G....G.................................................................TG..........................

20810 20820 20830 20840 20850 20860 20870 20880 20890 20900

....|....|....|....|....|....|....|....|....|....|....|....|....|....|....|....|....|....|....|....|

EP_scaffold155 TGGGAACTTGAGCCTCAGATTTGGATCTTCTCCTGCTAGTCTTCTGAGCAGCTGATAAGCCACAGAACACCCACGGATTTTATATTTATTAATAATTACT

AP_scaffold14 .........A......................G.........................................A.........................

20910 20920 20930 20940 20950 20960 20970 20980 20990 21000

....|....|....|....|....|....|....|....|....|....|....|....|....|....|....|....|....|....|....|....|

EP_scaffold155 CATTTGTCTTCATAACAGTTTCCTCTCAGACTATTATTTTACATGCCTCCATTTTAAATGCACAAACTGTTTACCCTTGCTGGGCACTGATAGTAAGGAA

AP_scaffold14 .............................G..................................................C...................

.

EP_scaffold155 A

AP_scaffold14 .

**(iv) *Tinamus major* and *Struthio camelus***

10 20 30 40 50 60 70 80 90 100

....|....|....|....|....|....|....|....|....|....|....|....|....|....|....|....|....|....|....|....|

GT_scaffold4190 CTATGAGTCCTAGCCAGTGTTGTGCAGAATGCTTTATACCTGCCTTT------GTTTGTAGAAGATGTCTTTACAATAACTTTCCAATTTAGATACACTC

OS_scaffold51 A.G...........T.A.AC...........G......G........AACTTT....A..T.....C....C.................-........C.

110 120 130 140 150 160 170 180 190 200

....|....|....|....|....|....|....|....|....|....|....|....|....|....|....|....|....|....|....|....|

GT_scaffold4190 TGA-TTTTTTTATTTGCTGAGCCCTACCATATAAGAGTTTTCTGCCAGCAGAAAGCATCATAGTTCTTAAGAATAACT-TAAAGCTAACATCAGCATTTC

OS_scaffold51 ...A..............A............A..T.A...............G..G...GCT..C.C........G..C....C....AGA.T..G....

210 220 230 240 250 260 270 280 290 300

....|....|....|....|....|....|....|....|....|....|....|....|....|....|....|....|....|....|....|....|

GT_scaffold4190 AGCTCTATTTATTGCTGGCTCCATAACCTCTCCCTTTGAGCTACCTGATCCCCACCGGAGAGGCCATGAGACAGG----------AGGCTCCTGTGGCCC

OS_scaffold51 ......G................C.GTG.....TC.CTG...GT.C.G.....GTT......C...C...G....TGAGTGCAGG...............

310 320 330 340 350 360 370 380 390 400

....|....|....|....|....|....|....|....|....|....|....|....|....|....|....|....|....|....|....|....|

GT_scaffold4190 TGGGTCTAAGGATTAAGGGTCCGAGCAATGTCAATCTGGTAGCCAATGTGGGCATGGAGCCACCTCGGCCCTGGGGCTGCAAGTCGGGTGAAATGCGGCT

OS_scaffold51 ..ATC----A..GC.......TA...G......GG..A.C...TGC........A..G..TG..A.A...G......A.TC...G........C..A...

410 420 430 440 450 460 470 480 490 500

....|....|....|....|....|....|....|....|....|....|....|....|....|....|....|....|....|....|....|....|

GT_scaffold4190 CTCTTGGAGGAGCGCCGGGTGGCTGCACTCTTCCCTGTGTGCC--------TTCTGGGCCTGTGGTGGAGCTGGGAACCTTGGCGTGCTGGCTCAGCACC

OS_scaffold51 ...............A.........TGTG.A.T..CAG.....CGACACCT.C.....G................C....C...A............G..

510 520 530 540 550 560 570 580 590 600

....|....|....|....|....|....|....|....|....|....|....|....|....|....|....|....|....|....|....|....|

GT_scaffold4190 GCTGTTTAGCTCAGCGGTGGTTGGAGGCACTGGTGACATTTACTTGGGCCTCATTTCAAATGTTTTCCCCAGTAAATTCAAGGCCAGCGCAGTCCGTTGA

OS_scaffold51 A..A................C............................A...........A...G...-.............TTGA.....C..A....

610 620 630 640 650 660 670 680 690 700

....|....|....|....|....|....|....|....|....|....|....|....|....|....|....|....|....|....|....|....|

GT_scaffold4190 CAATGATCCGTTAAGCAGATGAGTGTAAACAATGGCCATGGGAGAACAGAGGCCGTATTCACCTCGTCTGGGAATATGACACGG-ACCTATAATTCGCAC

OS_scaffold51 .......................................TA............T.C........T...C...............G...........T...

710 720 730 740 750 760 770 780 790 800

....|....|....|....|....|....|....|....|....|....|....|....|....|....|....|....|....|....|....|....|

GT_scaffold4190 CACGTTACTTCTGCCTCATGGTTGCTTTCTTGGTCCTTTTAATGGAATGGCTCTTCTTTGCTGTCTGACCTCACATCCCTCAAGGCAGGACAAAACCCAG

OS_scaffold51 TG..........CTG.A.CT...........T.........G..............---A........................................

810 820 830 840 850 860 870 880 890 900

....|....|....|....|....|....|....|....|....|....|....|....|....|....|....|....|....|....|....|....|

GT_scaffold4190 ACGAAAATATCGCCTTGTTCGTACATAATAAAAAAGTCATTTTCACCCTAAACCAGGACTAGTTTTGCTCTAAAACTGGCGAGCTCTCAATGGAAAGTGG

OS_scaffold51 ...........AT..................................................G..A........T....A........G..........

910 920 930 940 950 960 970 980 990 1000

....|....|....|....|....|....|....|....|....|....|....|....|....|....|....|....|....|....|....|....|

GT_scaffold4190 GCTTGATATTACTTTAACAAAATGTGCAGAAGTGAACCCATTTGTTTATATTGAGCTAGGAAAATCCCTTTGCTTTGCTTTAGCTGCAAATGTTTTTCAC

OS_scaffold51 ..CCA.......C........G..A.........................C...ATG.............CA.......G...T.A..............

1010 1020 1030 1040 1050 1060 1070 1080 1090 1100

....|....|....|....|....|....|....|....|....|....|....|....|....|....|....|....|....|....|....|....|

GT_scaffold4190 ACTACTGTTCAGGACTTCTGTTTTTTTATGTCTGGCCTAAAAGGCAGCTTTCTCCAGCCGTTGAGTACTTGTTAACCCCATTTATGGGAAATGGTTCTGC

OS_scaffold51 ..........................-.C..............A...............A.C...G.........................G.T..A...

1110 1120 1130 1140 1150 1160 1170 1180 1190 1200

....|....|....|....|....|....|....|....|....|....|....|....|....|....|....|....|....|....|....|....|

GT_scaffold4190 AGTGAGTCAGAGGGAAGAATGACACTTATTGAATCACCAGCACTGGTCCCTTGGGTTTTCCTTGGAGGCTTCTCAACTAAATATTAGCTAGGAAAACACA

OS_scaffold51 .C................................................A.....C...........T...............................

1210 1220 1230 1240 1250 1260 1270 1280 1290 1300

....|....|....|....|....|....|....|....|....|....|....|....|....|....|....|....|....|....|....|....|

GT_scaffold4190 GCGACACAGACATTGCCACACGAACTGAAACCACTGGTCCACCTATCATGGCCCTGTCCACACAGACATGCCTATAACCAACCGAGCCCAAGCTCTGTCT

OS_scaffold51 AG.......GG..........TG...C.....G.....T...........................................T.......G....C....

1310 1320 1330 1340 1350 1360 1370 1380 1390 1400

....|....|....|....|....|....|....|....|....|....|....|....|....|....|....|....|....|....|....|....|

GT_scaffold4190 TGGCGTGAGATCAGGCACCGCAGCGTTGTGTCCAAATCTGACATCTCACTGTGCACCGAGTCTTTCCAAGAGGTAGAGGATACTCCCATGTCAGCAGCAG

OS_scaffold51 ....A...................T...CA.....................CT...TA............C..C.......GT........T.......A

1410 1420 1430 1440 1450 1460 1470 1480 1490 1500

....|....|....|....|....|....|....|....|....|....|....|....|....|....|....|....|....|....|....|....|

GT_scaffold4190 CATCTTCTACGCCGGCCTGATGCTCTCAGGCAGAAAATTAAAGTGCTTCCCGATAGCATCCACTGTGTGTCCCTCTTCACTCCAGCTCTGGAGCTAATAG

OS_scaffold51 ..A..CAC.T..T...T..T.........A...C.........C......A.G............CA.C......C....C.......C.......G...

1510 1520 1530 1540 1550 1560 1570 1580 1590 1600

....|....|....|....|....|....|....|....|....|....|....|....|....|....|....|....|....|....|....|....|

GT_scaffold4190 AG--AGGTGGTGATGGCACCAGCAGATAGTGCTGCTCTGCCTCTCTCTGTCTACGCTTTTCCCTTTGGGTGAGGA-CTCACCAAAACTGCCTTCCCCGCA

OS_scaffold51 ..GC.ATG...T.....................T.C..A..........C.C.G........G..........A.G.........G..........AA..

1610 1620 1630 1640 1650 1660 1670 1680 1690 1700

....|....|....|....|....|....|....|....|....|....|....|....|....|....|....|....|....|....|....|....|

GT_scaffold4190 TTGACTTTGA-------------------GTCTCTGCCGTCCATTCCTGCCCCTCCAGAAACAAGCCATTTTTATTGTGC-AAGCTGTCCGAGTAAACTG

OS_scaffold51 C..G.....GCTGTGGGGGGTCCCACCAT......CT.C..............C.TGT.....................TG........TAG....G...

1710 1720 1730 1740 1750 1760 1770 1780 1790 1800

....|....|....|....|....|....|....|....|....|....|....|....|....|....|....|....|....|....|....|....|

GT_scaffold4190 AGTAGTGGCACAAGTGGGTGCCAAAATGGGTATTTATCAGGAATTGTCCAGAAGAGAAGGTTTTTCTTAATCTGGAATTTCAAAGCAGTTGTAATTCCCT

OS_scaffold51 G.........G...C.------------..C....GC...T.....C.A....T..G...........C......G...A...........--..G....

1810 1820 1830 1840 1850 1860 1870 1880 1890 1900

....|....|....|....|....|....|....|....|....|....|....|....|....|....|....|....|....|....|....|....|

GT_scaffold4190 GAAGAATTATTCTACCTTGAATTTTTAAACATTTAAAACATTTAAAAATGTTTTTTTTTTTTAAAAAAATCTTTCTTA--GCCTTAAATGTATCTAAAAT

OS_scaffold51 .................GA....C..--.TG...T...T....T........C...A----A....CTC..C..GC..CT.A....GG...G....G..A

1910 1920 1930 1940 1950 1960 1970 1980 1990 2000

....|....|....|....|....|....|....|....|....|....|....|....|....|....|....|....|....|....|....|....|

GT_scaffold4190 TTAAGCATTATTGTCTGCTGTATAATGAGGTAAGTTATATTTCTTATCCTTCTGTTGCACAGAAGCTTTTATTAAATTTTGAAAAACTTTGAAAAAACTT

OS_scaffold51 ....AAG......C.....A.G....A.AA..G.........A...............G....G..C..CG............G..TG.......G....

2010 2020 2030 2040 2050 2060 2070 2080 2090 2100

....|....|....|....|....|....|....|....|....|....|....|....|....|....|....|....|....|....|....|....|

GT_scaffold4190 ----ACAGTATACATTTTATGTAGGCTGGATACATCTGTTACACAGGAGAC----ACCATAATGTCTTATGTAGGCTTTGCATTTTTTTGTTTTACCTGA

OS_scaffold51 CATGGT....GG...................G.....A...........GTGTTT....CT..C.....CA.................-...........

2110 2120 2130 2140 2150 2160 2170 2180 2190 2200

....|....|....|....|....|....|....|....|....|....|....|....|....|....|....|....|....|....|....|....|

GT_scaffold4190 ATGTATTTTACACTGAAAATAACAGGAAGACATTACTCTGTGTGTGTGTGAAGGGAACAGAGAAATGGAGTGAAGTCATCATGCATCACACTTAGCAGGT

OS_scaffold51 .G........T....T..................G..........A...........A............CA...C......T...T.............

2210 2220 2230 2240 2250 2260 2270 2280 2290 2300

....|....|....|....|....|....|....|....|....|....|....|....|....|....|....|....|....|....|....|....|

GT_scaffold4190 GTGAAAAGTGCCTTAATCCCAGCAGAGATCCCAGCTCCTTCCCAAATTATGCCAAAATAGGACCCAGCACAT--GTCGGGAGTGCTGGAAGAGGATGCAG

OS_scaffold51 .........A..C.G...T.T...T...........G.......................C...AGAT...CAA...A....................G.

2310 2320 2330 2340 2350 2360 2370 2380 2390 2400

....|....|....|....|....|....|....|....|....|....|....|....|....|....|....|....|....|....|....|....|

GT_scaffold4190 GACTGAA-------------------------------TCAGTTCAGCAATCTCTTTAAATCTCAGCAGGAGATTTCAAAGTGTGGTCAA-AAAATCTTG

OS_scaffold51 .......GGGTGTCACACTCCTATCAGCCTGCAATGAA............C..................................A....T....G....

2410 2420 2430 2440 2450 2460 2470 2480 2490 2500

....|....|....|....|....|....|....|....|....|....|....|....|....|....|....|....|....|....|....|....|

GT_scaffold4190 AGCCAAGCTGTGATCCCTGTGGAGGCTGATATTCCTGTTGTCTCCCGGAGGACTCTTCGGATTGGGCTCCAGTTGTATTGCCAAACTCTTGGCAAGATCA

OS_scaffold51 .T......CAC..................................T...A.......T....CA........C...G..........TC.A.........

2510 2520 2530 2540 2550 2560 2570 2580 2590 2600

....|....|....|....|....|....|....|....|....|....|....|....|....|....|....|....|....|....|....|....|

GT_scaffold4190 GGTGTTTCATGGCTCCTCCTCTCTTCCTCTCTCCAGGGTAATTGCTTCCTGCCAAATGAAGGCCTTGATAAAGCAGTTTCAAATGCAGAAATTTCCTCCT

OS_scaffold51 ...........A....CT.CT...C..C..A.....A........AC.T.............T.C........TG....................T.T..

2610 2620 2630 2640 2650 2660 2670 2680 2690 2700

....|....|....|....|....|....|....|....|....|....|....|....|....|....|....|....|....|....|....|....|

GT_scaffold4190 TCGTTTGATTG----AAAAGCCATTGCCTAGTATTGGCAATGACAAATGTCTTTTCCAGTCAAGCTCAAAGCTGGACTCACTTCAGTGGTGCGTGAGGCT

OS_scaffold51 .TC..CC....CTTT............A.....C.........TT....AT..........C...............A........CA............

2710 2720 2730 2740 2750 2760 2770 2780 2790 2800

....|....|....|....|....|....|....|....|....|....|....|....|....|....|....|....|....|....|....|....|

GT_scaffold4190 CCTTAGTACTTTCTATTATGAGGTCTTATTCTTCAGCACCAATAATTTTGTTAAATTTTAACCATTTGGGTTGAATTTTTCCATCCTAGGCATCTGCCTC

OS_scaffold51 T....A.................C.CGC.....AG............G..............T.......C....A........................

2810 2820 2830 2840 2850 2860 2870 2880 2890 2900

....|....|....|....|....|....|....|....|....|....|....|....|....|....|....|....|....|....|....|....|

GT_scaffold4190 AAGCTTTTTGCCTTGTCTTTTAGTCTGTTTTTTATAGGGAGGTGGACAGAGAGCTGGAAATATCATCTAAAAGCTACAGTCATTTCCAAGAGCAAGGCT-

OS_scaffold51 ..........AT....................-.C..CA..A....GG..A.A........T...G.......G.G..A.........G..AGG...T.C

2910 2920 2930 2940 2950 2960 2970 2980 2990 3000

....|....|....|....|....|....|....|....|....|....|....|....|....|....|....|....|....|....|....|....|

GT_scaffold4190 AGGGAAAAAATACTGATTTAGCAGGAGGTGGTGGTGGAG---------TCTCTTGCCGTTTCCACTGGATGAGACCAATGCCTTTTTGAGGGCGGAAGTG

OS_scaffold51 .............G.T.......A.G.....G..GT...GGGGCAAAA...TG.A.TC.....T..AA.CA.T....G.A......G..T...AA.....

3010 3020 3030 3040 3050 3060 3070 3080 3090 3100

....|....|....|....|....|....|....|....|....|....|....|....|....|....|....|....|....|....|....|....|

GT_scaffold4190 GTACAGGTTTGATGCGAG------------GGCCAACTGGAAGCCTGTGGCTGGGCTAAAGGGGGTCAAGATCAACCCAGGACCAACTCCTGTTGACATC

OS_scaffold51 .........G....A...CGGGTATGGGAGA.G.......G......-A.T.....AG...A..ACTGG.....-.TG.......G.......CAC....

3110 3120 3130 3140 3150 3160 3170 3180 3190 3200

....|....|....|....|....|....|....|....|....|....|....|....|....|....|....|....|....|....|....|....|

GT_scaffold4190 AGCTGCTCCTGCAGGGTTGTCACTCTTCTAAAAATCCACTCCAATTTGGAGGGTATCAAAACAAGTCA-TATGTAGTGAGCAGCTTTGTGTGAAAACCTT

OS_scaffold51 .......T...........C.........G.........GTGG.C...T..AC.G.............A.G..C.......G.T......A....G....

3210 3220 3230 3240 3250 3260 3270 3280 3290 3300

....|....|....|....|....|....|....|....|....|....|....|....|....|....|....|....|....|....|....|....|

GT_scaffold4190 GGTTGGAGCGACTGGCCTGGCACAATGCTAAGTCTGTGCCAGAGGCAGAGGTGAAATGCAGTTCCTTAAAGCATCTTAGTTTTGAGTAACC--CCTGTTT

OS_scaffold51 ...CA.G.T....A........TG.GA...G.................G.A..T.....G......C......G.C.T.AG..ATA..GT.AC..CACG.

3310 3320 3330 3340 3350 3360 3370 3380 3390 3400

....|....|....|....|....|....|....|....|....|....|....|....|....|....|....|....|....|....|....|....|

GT_scaffold4190 TAACAGTAATCTCTGCTGCTGTGGTTGCACACATCCTGGCGACTGCAGCAAG--GTCGTTCTGAGAA---CAGCCACCTTCCTTGCATGACC-TGTTGGT

OS_scaffold51 ....T......C.G.....CT...C...G..TG......T.C.....A....CA.AG..C.A.TA..ATG......TTC..A...TGCAG..C....CAC

3410 3420 3430 3440 3450 3460 3470 3480 3490 3500

....|....|....|....|....|....|....|....|....|....|....|....|....|....|....|....|....|....|....|....|

GT_scaffold4190 GTGTGCAAACAGGTAAGCAAAGGCTGGATTGTGCTGCATAAAATTCACTGTGCTGAAGCAAGGCCATGCTTATGACTCTCCATGGTGCTAAGA----TGC

OS_scaffold51 .C.......AG....GCTG..A............CA.G.....GC..TG...........T...TGCA...G.A.....G...A..AG.....CTAC.A.

3510 3520 3530 3540 3550 3560 3570 3580 3590 3600

....|....|....|....|....|....|....|....|....|....|....|....|....|....|....|....|....|....|....|....|

GT_scaffold4190 TTGAGATTTGGACTGCGGTTTGGAGCAAGGTCTAAGCACCACTACAAACTTGGGC-TAATAGACCTTGTAAGGCTGGGGAACCTGTAAGGCCT------A

OS_scaffold51 .ATG.......GG...A..........G............TT...C...C.....C.CG.G....CC..G.....A......TCCC.CTTG..TTCTCG.

3610 3620 3630 3640 3650 3660 3670 3680 3690 3700

....|....|....|....|....|....|....|....|....|....|....|....|....|....|....|....|....|....|....|....|

GT_scaffold4190 GGACAATCGGGTCTAGGTGTATTGCCGAGTTGTTATGCTGCCTGTATTTTACTGCATTTTTTTCCTAATCCTAGCTTTGAATAGTTTATTCATAGACTAT

OS_scaffold51 ...GG...........T.A..........C..CAT.C...TG.A........A..-C...........CT.C..........................G.

3710 3720 3730 3740 3750 3760 3770 3780 3790 3800

....|....|....|....|....|....|....|....|....|....|....|....|....|....|....|....|....|....|....|....|

GT_scaffold4190 AACCTCTCTCAAGGCAAGGGCAGTATTTTCATTGATTTAACATAGCCGAGGTTTCAAGCCAGGTCCTAAAGAATAAATAAGAGATAAGTTTCTCATGATT

OS_scaffold51 ......A.....AC..........G......C...............A..................C...A.....-------..G.........CCG..

3810 3820 3830 3840 3850 3860 3870 3880 3890 3900

....|....|....|....|....|....|....|....|....|....|....|....|....|....|....|....|....|....|....|....|

GT_scaffold4190 CAATGGAGGCTAAACCAGAACATCTGGAATACTCCTCTTCTGCCCTCTCTCAGGAGTGGAGGAGGAA---TATGTTGCCTAATGTTTATGTTAGACAAAT

OS_scaffold51 ......G....G.....T.G..........GT......C.........T.....GCG.A....TAG.GCG..A.....T..TA.....AA..........

3910 3920 3930 3940 3950 3960 3970 3980 3990 4000

....|....|....|....|....|....|....|....|....|....|....|....|....|....|....|....|....|....|....|....|

GT_scaffold4190 ATAGTATACTAGTAAAAAAAGAAATCTGTTGTGTTGGCTGTCACTGGAGAAAGATAACAAATCTTGTGTATGTAATCCAGAGTGAGAGGTGATCTGTCTG

OS_scaffold51 ..TA---GT..A.T.....--...........A.............A....T........T.T...G...............C......C.G.T..A.C.

4010 4020 4030 4040 4050 4060 4070 4080 4090 4100

....|....|....|....|....|....|....|....|....|....|....|....|....|....|....|....|....|....|....|....|

GT_scaffold4190 TGGTTTCAAAAATCTGAAATGCTTGTGGTCATAATATTTACAGCTGTTTATCTCTGGTGGACAAGCAATAACTATTTTACTCTGTTTATATAGGGCAAAA

OS_scaffold51 ......G.....C....C.................G...G...AG...............G..........T........AT...G.G..CG.......C

4110 4120 4130 4140 4150 4160 4170 4180 4190 4200

....|....|....|....|....|....|....|....|....|....|....|....|....|....|....|....|....|....|....|....|

GT_scaffold4190 TTTAATAATGATGCTGAGGAGCAAAACAGCACTTTTGGGTCTT---AAACAAGTAAGAG-----CAGCTCTCCTAGAAGCACCAGTTGTGTTGATATGCT

OS_scaffold51 .........---...................G...CA......CCT.....G.CT.AG.AACAT...T.T...C......C.........A........C

4210 4220 4230 4240 4250 4260 4270 4280 4290 4300

....|....|....|....|....|....|....|....|....|....|....|....|....|....|....|....|....|....|....|....|

GT_scaffold4190 CTTGG--TTGG--GGAATTTAATTATTTATTTATTCTTATTTCTGCTGAGCAGTGAGGATGTGGTGGGC--ACCAGTGAGGACAGTGCACAAACTCTGGA

OS_scaffold51 ...T.CC....CT...G...GC..-...G...T.....T...TATA.ATTTTTCC..AGCACCAA..C.TGG...CCA.CC.G.A.........C..CTG

4310 4320 4330 4340 4350 4360 4370 4380 4390 4400

....|....|....|....|....|....|....|....|....|....|....|....|....|....|....|....|....|....|....|....|

GT_scaffold4190 AGATGTGCGGCC----------TGTCCTTCATTGCCCGTCTGATGCACTTTCATCTCCACTAACACCTGACGTCAAGAGGCCGTCTTCCCTCCTGGCCTT

OS_scaffold51 .......T.TA.GATGCTATTA.T.G...TGC....T.G......T..C....C....C..GT...T...A...........A...............A.

4410 4420 4430 4440 4450 4460 4470 4480 4490 4500

....|....|....|....|....|....|....|....|....|....|....|....|....|....|....|....|....|....|....|....|

GT_scaffold4190 CTCTTCAGCACTTTGGCAGAGATCTTCCCCAAACTTCTTTTCATCTTCTTTGGTAGCTAGAGATAAGATTTTAGTTGTGGGGTGATTCGAACTATTCTGC

OS_scaffold51 ...C....T.GA.......G...........-----T......................C..C..TA....A.C...C...A.....T..G...A....T

4510 4520 4530 4540 4550 4560 4570 4580 4590 4600

....|....|....|....|....|....|....|....|....|....|....|....|....|....|....|....|....|....|....|....|

GT_scaffold4190 ATGCATTTCTCTGCTGTTGCAGAGACCTTGTGTATTAGTGGCTCTAAGCCACTAA-GCCTTTTTCTCCATGCATCATGCTTTTAATCTCTTGAAGACTTA

OS_scaffold51 G.T..G.............................C..C..........T.....TA......G..G..................C............G.

4610 4620 4630 4640 4650 4660 4670 4680 4690 4700

....|....|....|....|....|....|....|....|....|....|....|....|....|....|....|....|....|....|....|....|

GT_scaffold4190 GATGATTTAGTTGAAGTGTCTGTATCTGCTAAAGGTATTTAGCCTACCAGTGATGACTTGGGTATTCAGGAGGTGCAACTAATGTGTTTTTTGAGTAGTA

OS_scaffold51 CC........CC.....-.T.AG..T.A............G.GTG..TT...G..G.C..C....A........C..G....G..C.CC...TG--...T

4710 4720 4730 4740 4750 4760 4770 4780 4790 4800

....|....|....|....|....|....|....|....|....|....|....|....|....|....|....|....|....|....|....|....|

GT_scaffold4190 ACTTTCAACAAACATGTGCAAATACAAGGACTTGGACTGTGAATAA-TCTTTTTT---------------------------------------------

OS_scaffold51 .T......TG..TG...A......T.......CA.G..A..T...GG........GACAGCAGGGAAGGGTTGTATGAGGAGATAAGAACAAGTATATTG

4810 4820 4830 4840 4850 4860 4870 4880 4890 4900

....|....|....|....|....|....|....|....|....|....|....|....|....|....|....|....|....|....|....|....|

GT_scaffold4190 -----------TTTTTTAGGTTGGAGACTTGATTGGAGACAAGTTATTTCATTATTCTGGTCCTTGGCCTGACCCAGGGTGGCTGAGCTCATGTATGCTT

OS_scaffold51 GGTCTAACTCA..................CTG...........C..C.G..C...ATA.AC.A...AT...G..A...AG................T...

4910 4920 4930 4940 4950 4960 4970 4980 4990 5000

....|....|....|....|....|....|....|....|....|....|....|....|....|....|....|....|....|....|....|....|

GT_scaffold4190 TAGCATGCTCAGCATATTG-------------TGCAGCCTAGTGACCTGTTGTGCAAAGAACCATTTATTTTGTTTAGCTTCAACCTGTAACATTCTCAT

OS_scaffold51 G..................CAGAAGGCTCTTG.......C.A..................G.G....G...........G...G...------.......

5010 5020 5030 5040 5050 5060 5070 5080 5090 5100

....|....|....|....|....|....|....|....|....|....|....|....|....|....|....|....|....|....|....|....|

GT_scaffold4190 CTCAGATGAGGCCTCTTAGTTGCTATTTCAGAAGCGATGGTGAAAAAAATTTTAGATGCCTAAAAAAGAATCAAAAGCTATTCAGGAGTTTTAAACATAA

OS_scaffold51 .........TA..-.C.....CTCG.ACTG....A.............T.A.C..GC.TGG.....GT....GGC.............G.......G...

5110 5120 5130 5140 5150 5160 5170 5180 5190 5200

....|....|....|....|....|....|....|....|....|....|....|....|....|....|....|....|....|....|....|....|

GT_scaffold4190 AGGTGCCCTTAATGAT--CTTCTCTACTAAATTTTTCAGAACAATATCAGTTTCAGCAACATTTTCAGTGTATTGAATACATCTTAAAAATGTATTTTGG

OS_scaffold51 .T..AT.....G..TCTG.........................G.........T.......C.C....CAGCA.....GT...C.............G..

5210 5220 5230 5240 5250 5260 5270 5280 5290 5300

....|....|....|....|....|....|....|....|....|....|....|....|....|....|....|....|....|....|....|....|

GT_scaffold4190 ATGTTCATATTTCAAAACAGTATTGAGATGACTTCCACCAGATACACACATCTTGGGTGTCTGACTTAAACTCAGTATCCATTGAATCTTAATGTGACTG

OS_scaffold51 .......G...-.....TGTC....G......................T.......C.A...........G.....G..TG.....C....C....T..A

5310 5320 5330 5340 5350 5360 5370 5380 5390 5400

....|....|....|....|....|....|....|....|....|....|....|....|....|....|....|....|....|....|....|....|

GT_scaffold4190 TCAACTGGAAGTCGGATGTCAGGGAACTGTTGTTTGGTTCTTTCCGCATATTTAATGACTTCTG---AAATGCACTGGGGATACAAGTGCAAGGGGATAC

OS_scaffold51 .AGG...AGGA..T.C..GTG..A....--......A......T.CTG.....C..C.......CTG.......T.ATTTGC.-------.....A....

5410 5420 5430 5440 5450 5460 5470 5480 5490 5500

....|....|....|....|....|....|....|....|....|....|....|....|....|....|....|....|....|....|....|....|

GT_scaffold4190 AAGGTACAATAAACCAGAGTGTGTTTTGGACTGCATTTTTTTTTATGGTGTGAAATGGTCATGGGCTGCTTGTATGGCCTATGGAATTTTGGTCAGCTTT

OS_scaffold51 .........CT..T....T.AAT..----....-.GA..AA....A.A.........AC....A...---CA.....A....A.T...........T...

5510 5520 5530 5540 5550 5560 5570 5580 5590 5600

....|....|....|....|....|....|....|....|....|....|....|....|....|....|....|....|....|....|....|....|

GT_scaffold4190 ATCTGGGCAATATTTTGTATTAAGTTATTTATATATAATGGAAAAGCACATTCTATATTTTCTAGAATAATCTATTTTTCTTTCTCTATACAGACTGCTC

OS_scaffold51 .A.......GC.....A......A.......C..G...A.A.....TG....AC...........C.....................GC...........

5610 5620 5630 5640 5650 5660 5670 5680 5690 5700

....|....|....|....|....|....|....|....|....|....|....|....|....|....|....|....|....|....|....|....|

GT_scaffold4190 ATTCTAAGCAAGTGG------TTTGAGAAAGGTAAGTAAAGGATAAACAATAAATTTGTATGTTATGTAACTATTCTAGTTTAGTGCTTCTCTAATTTAC

OS_scaffold51 ......G........GAAAGT....G.....A...............T.G.T.....T.................A...A....................

5710 5720 5730 5740 5750 5760 5770 5780 5790 5800

....|....|....|....|....|....|....|....|....|....|....|....|....|....|....|....|....|....|....|....|

GT_scaffold4190 TTGGAAGTAAGTCTGTACTACTTTCCTGTCACTCATAAATATTGATAGGTACTATTTTGTTAAATAAATAATTAGGACCATCAATTTCCCCTCGGAATCC

OS_scaffold51 ..AA...........C...............................A........................................A.T.T....A..

5810 5820 5830 5840 5850 5860 5870 5880 5890 5900

....|....|....|....|....|....|....|....|....|....|....|....|....|....|....|....|....|....|....|....|

GT_scaffold4190 ATCTCAAGTGGCACTCT--AGTCTCTCATCAGTTTATAAATACTGGCCAGCTGTGCTCGTCAGTGCCTTAAGAAATTCAAGGGTGGGGACACTTCAGGTC

OS_scaffold51 ....A............CT.A......C....C.A.........A.....A....T.T.G.....T..G.............A...........T..AAT

5910 5920 5930 5940 5950 5960 5970 5980 5990 6000

....|....|....|....|....|....|....|....|....|....|....|....|....|....|....|....|....|....|....|....|

GT_scaffold4190 TAATACAGTACTCTTCTATTTTTCAGGCTTAATTATTAACTTCCCATGCAGATATTTTCATCAACCAGTAGGGGGAATGCTAATTCTTTAGTGTTGCTAG

OS_scaffold51 .........C.......G......G..T.......G........T........G........TG...........T.....T...............C..

6010 6020 6030 6040 6050 6060 6070 6080 6090 6100

....|....|....|....|....|....|....|....|....|....|....|....|....|....|....|....|....|....|....|....|

GT_scaffold4190 TTGGAGGAGCATCTGTCCTCAAGGAAACA--GTACCTGGGGCAAGGAAAGCTAACTGGTTCCTAAGTGCTGTACCAGTGCATAAAAGAAGCAGGAAGAGG

OS_scaffold51 .C.......TG......A..C......T.TA..G.......................................AG......C...G.......A..C...

6110 6120 6130 6140 6150 6160 6170 6180 6190 6200

....|....|....|....|....|....|....|....|....|....|....|....|....|....|....|....|....|....|....|....|

GT_scaffold4190 AAAAGTGGGAAATTTGTG----------------TGTTCCAGGGGCTTTCTTTGAAGCGCAGCAATGCACATAGCTCAGGATGTGGCACTTGTGCTGGAG

OS_scaffold51 ..G..AAA.........CCCGGGTCGGTTATTGC.......A........CC....C...........................A....G...A......

6210 6220 6230 6240 6250 6260 6270 6280 6290 6300

....|....|....|....|....|....|....|....|....|....|....|....|....|....|....|....|....|....|....|....|

GT_scaffold4190 GCCTGGGAGA-TGGATGCATTACTGTGAGGTGGGACAGT-----------------GTCATCTGCTCT--------------------------------

OS_scaffold51 .TT.....A.GCA.....TG.G...CAG.AC.T....A.CTGGGATTTTAGTGCTT....C.C.....ATAGCACCTGTCCATGTGCATGCTTTTGCTCT

6310 6320 6330 6340 6350 6360 6370 6380 6390 6400

....|....|....|....|....|....|....|....|....|....|....|....|....|....|....|....|....|....|....|....|

GT_scaffold4190 ----------------------------------------------------------------------------------------------------

OS_scaffold51 GCAAATAGTATAAAATAACTGACTCTTCTTTACTGGGGCAAATTACTAAGGACCAACTGATGCACAATATCTTATTCCCCACGACAATTTGACTGTGTAT

6410 6420 6430 6440 6450 6460 6470 6480 6490 6500

....|....|....|....|....|....|....|....|....|....|....|....|....|....|....|....|....|....|....|....|

GT_scaffold4190 ---------------------------------------------------------------------------------------------------G

OS_scaffold51 CCATAACCTCAGTTATCTGTCAGAAAATGAGCTTATATTTGCGACTTTCAATTTCATATATAAGATTAGAAAGGCCTAGTATATCACATTGGTCATAAA.

6510 6520 6530 6540 6550 6560 6570 6580 6590 6600

....|....|....|....|....|....|....|....|....|....|....|....|....|....|....|....|....|....|....|....|

GT_scaffold4190 TTTCATTCAAGGAACTCAGTATTGAGCCCAATAACTTTTATTCAACTGAAGTGCAC-TTCCAGAAAGATACTAACTTGTTCTTAAATCTCTAGAGATGGA

OS_scaffold51 .......G.......C..A.......A.T.G..............T.A........C..........GCGA..G..........G..A............

6610 6620 6630 6640 6650 6660 6670 6680 6690 6700

....|....|....|....|....|....|....|....|....|....|....|....|....|....|....|....|....|....|....|....|

GT_scaffold4190 GAATCTTCCACTTCCTTG-AAATGTGGTTTCAATGCCAGAATATGGCTTTTGATTTAATATGAGTTTGTCTGTTTTTAGCTTCTAGTCCTTGACTGTGGC

OS_scaffold51 ......A.....CT....C.............T.C........A..A....A...........A.........C....A.......C...........AT

6710 6720 6730 6740 6750 6760 6770 6780 6790 6800

....|....|....|....|....|....|....|....|....|....|....|....|....|....|....|....|....|....|....|....|

GT_scaffold4190 ATTTTGTTTTGAAATACTGAAATGCTCTAGTTTCTTCTGGGCATTCATTTTTCTGACCAAAGCACTGATGAAACCTTCAG-GGTGGATTCTAGTTGGAGC

OS_scaffold51 ........................TA.CT.............----...........A...A..........T.T.C...T.........C.....C...

6810 6820 6830 6840 6850 6860 6870 6880 6890 6900

....|....|....|....|....|....|....|....|....|....|....|....|....|....|....|....|....|....|....|....|

GT_scaffold4190 AGCAGATCTGGAAGTGTGAATTTCTTCCTGCACATAGCATTTAAAGACATCTGCAAAATGATGTGTAGAAACACATCCGGCATTTCCATACTTCAAGCTC

OS_scaffold51 CA...........A...........G...A...G.........GCAGTGCG.C..C..A......C.....TG.....A.............G...A...

6910 6920 6930 6940 6950 6960 6970 6980 6990 7000

....|....|....|....|....|....|....|....|....|....|....|....|....|....|....|....|....|....|....|....|

GT_scaffold4190 TGGCTGATTTTGGTCAACACAGAACTTTCAAAACATTAAGAAAAGGACTGAAGACT--ACTTTTTTTCACTTTGAAACACGCTGGTGAACATTTAGTTCC

OS_scaffold51 ......G....T..T........G...........C...-........C.C.....TTG.....G..G...........TC...A...GA.........T

7010 7020 7030 7040 7050 7060 7070 7080 7090 7100

....|....|....|....|....|....|....|....|....|....|....|....|....|....|....|....|....|....|....|....|

GT_scaffold4190 AAGGAGGTTGGTTTGGTTTTATTAATTGATGAGCTTTTTTTTTTTTTTTTTCAGAAAATGAATTTTCCTTTGGAGTGGGTCTGACTATGCCCTTTAGGCA

OS_scaffold51 .G.A..-----C...A............C.........C..---------G........T.G...CT......G...TC...C......G..C.C.A...

7110 7120 7130 7140 7150 7160 7170 7180 7190 7200

....|....|....|....|....|....|....|....|....|....|....|....|....|....|....|....|....|....|....|....|

GT_scaffold4190 AATAGTTTATAAGTAGTTTATTTGTAAAGCCAGAAAAAAATCAAGAAGACGTGCAATTTTAATCATCTAGTTGTTGAAAAGAAGAGCCATACAGCACCAC

OS_scaffold51 .GC...........CT.C....C...G......G....TT.T...................C..C....................A.AG.CG..A..T..

7210 7220 7230 7240 7250 7260 7270 7280 7290 7300

....|....|....|....|....|....|....|....|....|....|....|....|....|....|....|....|....|....|....|....|

GT_scaffold4190 CCAAGTGAGTTTCTGTTGTTTACCTTGGTATACATT--CCCCTTCATCAGCA---------------------TATCATAATAGCAG-AATATTCAGTCT

OS_scaffold51 ....----......AC......G.......A...G.AAT.A.CAT....AT.ATATTTATAAAAATCACCCTTC....CC...T..AT......T.....

7310 7320 7330 7340 7350 7360 7370 7380 7390 7400

....|....|....|....|....|....|....|....|....|....|....|....|....|....|....|....|....|....|....|....|

GT_scaffold4190 TTCATGGGTGAATTGATATTGCCATAGAATTTTGAAAAATTAAATGTTTTTTTAAATAACAAATTGAAATAAAAGGTATAATCTTGCCATGTAGTTGTTC

OS_scaffold51 .......A........C.C...TG....................GAGC...........G................A...C.T..........T......

7410 7420 7430 7440 7450 7460 7470 7480 7490 7500

....|....|....|....|....|....|....|....|....|....|....|....|....|....|....|....|....|....|....|....|

GT_scaffold4190 TCACTAAGAACCTCAGAATAAACTCTTTTTGACTCATTAGCCCTCATTCCAATCACAGAC-TCCTTGATTCAGCACAGAACAG--TGCTGGATGTTCCAG

OS_scaffold51 ..T.......TG..T...G.........C....A.....-...........G.TG..TC.C..................G...CC............AC.

7510 7520 7530 7540 7550 7560 7570 7580 7590 7600

....|....|....|....|....|....|....|....|....|....|....|....|....|....|....|....|....|....|....|....|

GT_scaffold4190 TTAAATTCAACCCTTAGGAAATGTGATTTCTCAGCTTGACTGCTGTGTTCAAGCAATAAAAGTCCCTCTTGCTAATTCTAGAAGTAAAGAAAATATTTAC

OS_scaffold51 ..TTG..............C.CA.....C.......G.GT.............A.......A..T...C....................C......G...

7610 7620 7630 7640 7650 7660 7670 7680 7690 7700

....|....|....|....|....|....|....|....|....|....|....|....|....|....|....|....|....|....|....|....|

GT_scaffold4190 CTTTATTATTTGTACAGTGGAAGCTTCCATTTGGTTAGCATAAGATGTCTTCTTCTCTCTCAAAAAAACCCACAATCCAAAAACATCATAGTATTGAAAA

OS_scaffold51 .......G....C....G.C............A..G..G..C.....G..----....TGA........---------.GC......T.CC.........

7710 7720 7730 7740 7750 7760 7770 7780 7790 7800

....|....|....|....|....|....|....|....|....|....|....|....|....|....|....|....|....|....|....|....|

GT_scaffold4190 AAT-AAAATTTATGTTAAAAGCAGAATAATTTTTGCAAGTCATTTAACCAATTTTTTTATTCCAAAGTGAAATTCTGCAGATTTCAAAAACACCATAATA

OS_scaffold51 ..AG........A......G.T.A....G...G.C..........TC.A.GA.......CC................TC.G........-..........

7810 7820 7830 7840 7850 7860 7870 7880 7890 7900

....|....|....|....|....|....|....|....|....|....|....|....|....|....|....|....|....|....|....|....|

GT_scaffold4190 TTTTT-AAGCTCTGCTCCCTTGGACAAAATCTTCCTAGGAGCCATGTTTTTTGTATGAAGGAGTGTAAAATGATCTTCTTACCTCACGGGTGTCTTCTCT

OS_scaffold51 .....T..........AT........C..C..C........T........C.AGC........C..........A..G....T...GA.AG....CTC..

7910 7920 7930 7940 7950 7960 7970 7980 7990 8000

....|....|....|....|....|....|....|....|....|....|....|....|....|....|....|....|....|....|....|....|

GT_scaffold4190 AAGTAAGGAAGATTTTTA--ATGAT--GTGATTCAT------------TTAGTAAAATGAATTATATGGGCA-----TACTTGAA---------------

OS_scaffold51 ..A......G....C...CT.....TCA.T.GC..AAAAAATTCTGTG.C..A.G....GG..G..G..AT.ATATCC.T..T..TTTGGGCTTGGGTTA

8010 8020 8030 8040 8050 8060 8070 8080 8090 8100

....|....|....|....|....|....|....|....|....|....|....|....|....|....|....|....|....|....|....|....|

GT_scaffold4190 ---AAAACCCTGTCCTTGACCAAAATACAGT----------CAAATAGTAGTTTCCTCTACTTGCAGGAAAACTTGAGCCTT----TGATACAATATAGA

OS_scaffold51 CCTG.........T........G.......GAATAGAGGAG.......G.A.....C.GG....T..T....G.GAG.TT..GACA.....T..C.G..C

8110 8120 8130 8140 8150 8160 8170 8180 8190 8200

....|....|....|....|....|....|....|....|....|....|....|....|....|....|....|....|....|....|....|....|

GT_scaffold4190 -------ATATAATTCTTCTT---TTTTTTCATAGAATGCTT------------AAGGTTGGAAGGGACTTCT---AGAGGCCA----------------

OS_scaffold51 TCAGTTT....GG..GC.T..AAT......G.ACA....TA.GAATGGCATGAT...AGCAA..ATA...CTCCAA.A.A....AGTATAAGGGATGGAG

8210 8220 8230 8240 8250 8260 8270 8280 8290 8300

....|....|....|....|....|....|....|....|....|....|....|....|....|....|....|....|....|....|....|....|

GT_scaffold4190 -------------------------TCTAGTCCAACCCCCCTGCACACGTAGGGTCACCTAGAGCACAT------GGCACAG--GGTGGCATCCAAGCGG

OS_scaffold51 ATTAAGAATGTTAATAGAAGAAAAA.G..A.AAG.AATGT.AAA...G.C..A.ATGGA.GTCAA.T..CCCGGC.A.....CA.TC.C.G.G.C...A.

8310 8320 8330 8340 8350 8360 8370 8380 8390 8400

....|....|....|....|....|....|....|....|....|....|....|....|....|....|....|....|....|....|....|....|

GT_scaffold4190 GT----------TTGGAAGATCTCCA-GAGAAGGAGACTCCA--CAAC---------CTCTCTTGGCAACCTGTTCC-----ATATAGCTAAGGTCA---

OS_scaffold51 C.CACAGTCCTT....C...C.AG..T.CC..T.T.C.....TC..G.TGTGCGGCA..GCTGA..AGG.T....G.CCTTA..GC...CGG..GT.AAA

8410 8420 8430 8440 8450 8460 8470 8480 8490 8500

....|....|....|....|....|....|....|....|....|....|....|....|....|....|....|....|....|....|....|....|

GT_scaffold4190 ----------GTTTATATGGTTGCTTTTAATTTTTTTTT--------AAACAAATTTATGAATGTCATGATAT-------------------GTACAAAA

OS_scaffold51 AGAGAGAAGG.....CTG.AAAT...C.CT..CCC.CA.GCATAGGG.GG.....G.GCACTG....CAT.C.TCCGAAACTCAGATTCAGA.C.T....

8510 8520 8530 8540 8550 8560 8570 8580 8590 8600

....|....|....|....|....|....|....|....|....|....|....|....|....|....|....|....|....|....|....|....|

GT_scaffold4190 ATAATT----------GCCAAAAGGCCAATT----ATAAGGTAC----------------------AGAAGAAAGGTGAAATCAG-----------AAAT

OS_scaffold51 C..C..CTGTTTAAATA...C.G.TGTG.C.TTGG..GGAAG..CTAATTACTCTTTCTTTTTAGA.A.G.G......GC.C.CACCATTTGTACA.TG.

8610 8620 8630 8640 8650 8660 8670 8680 8690 8700

....|....|....|....|....|....|....|....|....|....|....|....|....|....|....|....|....|....|....|....|

GT_scaffold4190 GCCAAAACAGAGG-----TAGGCATCAAATA---TGTGTTC---TTCTCTGCCTT--------TCTGTCATCTTTCTTCACAGCCCCTTGTGCCCATGTC

OS_scaffold51 T.T.G..T.A.CTGTGTT..A.TT.TG...TAAT....A..ATG.........CCATGACTCC...................A...........TG.CC.

8710 8720 8730 8740 8750 8760 8770 8780 8790 8800

....|....|....|....|....|....|....|....|....|....|....|....|....|....|....|....|....|....|....|....|

GT_scaffold4190 CTGTCCACTGCCCAGAGCAGCTGTCCCAATC--TCACCAGCTGCTTCATCATTCTCTGTCTTTGAAACAACTCTTTTTATGTTTATTTTTTTCTTCTCTC

OS_scaffold51 .GA....T.C........T.....G...G..ATC.T.TGT...........CC.............G.....A...C...A..CT.....CA--------

8810 8820 8830 8840 8850 8860 8870 8880 8890 8900

....|....|....|....|....|....|....|....|....|....|....|....|....|....|....|....|....|....|....|....|

GT_scaffold4190 AAGTGTTTTGCAAACTTGGGAGAGTAACCAAGAGACAGGACCTCCCATTGGTCAT-CCTTGTGTCTATTCCCACAGTGACCTCCATCCAGCAAAAGGTAA

OS_scaffold51 G.....G.........CA......AG..A.G....G..........TC.....G.T..........G.....GTT...G.....----.....G.T....

8910 8920 8930 8940 8950 8960 8970 8980 8990 9000

....|....|....|....|....|....|....|....|....|....|....|....|....|....|....|....|....|....|....|....|

GT_scaffold4190 GAGCAACTACATGAAAAGTCCTGACCAGCCTGGTCAACACAAGATTGAGCATGCTCAATGAAATGCTTTTAGGGATATTTTTTTCTGCCAAATGCTAACA

OS_scaffold51 ........G....GG..........A.A....A....TC...A..G...........G.A......A...TA..........G-..........T..G..

9010 9020 9030 9040 9050 9060 9070 9080 9090 9100

....|....|....|....|....|....|....|....|....|....|....|....|....|....|....|....|....|....|....|....|

GT_scaffold4190 ACTATTTGCATTTGTATTTTTGTTTTCCAGAACCATAGCCAGATTTGGGCAGATTTTCTTGCGCATATCATTCCTGCCTGATGTTAGTGCAACTGTCCTG

OS_scaffold51 ....C..A.T.-----.....T....T......TG...A......................G......TG...T..........C......G.C-.....

9110 9120 9130 9140 9150 9160 9170 9180 9190 9200

....|....|....|....|....|....|....|....|....|....|....|....|....|....|....|....|....|....|....|....|

GT_scaffold4190 CCAAATTTCAAGCCTTTGATACAAAGACTAGAGGCAAGATTAGATCTTTTCAATG---GAGTACATTTTCACAGAGTAATCCAAGTATGTATATGTATAT

OS_scaffold51 .............................G..A.....T.............G..AAG.....TGC......................A.....---...

9210 9220 9230 9240 9250 9260 9270 9280 9290 9300

....|....|....|....|....|....|....|....|....|....|....|....|....|....|....|....|....|....|....|....|

GT_scaffold4190 ATGTATAAAATTCCTGACACATCTATTGATTGATGATGTTTTCTAAATCTTTCTTGTCAAATTCGAACACCATTTATTCATTAAAATATTCTTTTAGT--

OS_scaffold51 TCCCGC.CC..CTA.----TG.TG.G..GCCTC..........C...C....G....TGG....A..........T.AA...........A.......GT

9310 9320 9330 9340 9350 9360 9370 9380 9390 9400

....|....|....|....|....|....|....|....|....|....|....|....|....|....|....|....|....|....|....|....|

GT_scaffold4190 ---ATTGTGTATGTCT----CTGAAAACATAAATTTATCTCTATTCCAATGCTGTTTTTCAGTTCTGAAGGAAAGTAAATTTGATTTGCAAGTGTTGTAT

OS_scaffold51 TAA.............GCAT.....C..G.TT........A.G..T........C.........T..GC.....C.G..C......-A....A...T...

9410 9420 9430 9440 9450 9460 9470 9480 9490 9500

....|....|....|....|....|....|....|....|....|....|....|....|....|....|....|....|....|....|....|....|

GT_scaffold4190 TCCCCTCCCTGTACATTTTAAAAGGAAGTACGCTTGGTTCTGCACACAGTGAGGGAACAGACATATATTGTTACAGACACAAACACTTTTCAAATGAGTA

OS_scaffold51 ....T.T.......T.............A.TA....A.......TG..A...A.T........................T...GG........G......

9510 9520 9530 9540 9550 9560 9570 9580 9590 9600

....|....|....|....|....|....|....|....|....|....|....|....|....|....|....|....|....|....|....|....|

GT_scaffold4190 TAAAGGAACACATTGCCTTCCACTGTCAATAGAATGTCGAGACTGATTGCATTTTAATTTGATGTTTGCCACCTGCATGTCTCATAAAAAGCATGAAACA

OS_scaffold51 ........AG.............C.......CTG.........A.....T.................ATT.......................CT.....

9610 9620 9630 9640 9650 9660 9670 9680 9690 9700

....|....|....|....|....|....|....|....|....|....|....|....|....|....|....|....|....|....|....|....|

GT_scaffold4190 CCAAACCAGGAGAGTAAGAAAAACAAATCCCTGACTTTTTGCCTTGCCATAATAGAGCTGGAGTTATTTCAGGAACAAGCTGCATTCGTTTTCCCTGGGA

OS_scaffold51 ................................T...............G............CA..GC..T....G.......G...G.......G.....

9710 9720 9730 9740 9750 9760 9770 9780 9790 9800

....|....|....|....|....|....|....|....|....|....|....|....|....|....|....|....|....|....|....|....|

GT_scaffold4190 CTCAAAAATATATGAGAGACTGTGGTTTCCAAAAAAGAATCAAAAAAAAAAAGACAAAAAGACAGCAATGGAAGAGAGAAAAAGACCCTTCC--------

OS_scaffold51 ..T.................CA...........T......-----.....G.A.TG...........C.A.........G............GAGATGCG

9810 9820 9830 9840 9850 9860 9870 9880 9890 9900

....|....|....|....|....|....|....|....|....|....|....|....|....|....|....|....|....|....|....|....|

GT_scaffold4190 ------CTTTGCACTGGGTTGTGCTTGACCGGACCACAGGACCATGCAGTTCCACTGAGTACGACAGCTCATCCTGTGGTTACTGACAAATTCACACATA

OS_scaffold51 TGGTTGT....TG....A..ACA..C.........T.....A...........T..A.CAGAC.......G.....-..............CG....C.C

9910 9920 9930 9940 9950 9960 9970 9980 9990 10000

....|....|....|....|....|....|....|....|....|....|....|....|....|....|....|....|....|....|....|....|

GT_scaffold4190 CCAGGGAGAAAAACAGCAGTGGAAACTGAGAAAAAAAAAAGGATTCTTGAGGGCAATACCTTTGTTGGAG---AATGCAATATAATTAAGCATAGCACTC

OS_scaffold51 .....A.......A.........G...........T----...........A......T......AA...GCA..CA...G.....A.GA.......T..

10010 10020 10030 10040 10050 10060 10070 10080 10090 10100

....|....|....|....|....|....|....|....|....|....|....|....|....|....|....|....|....|....|....|....|

GT_scaffold4190 CTATCACCAGTTATAGCTGCAGTGCTATATGAGATCACCTTGGAAGGTCTGCTAAGGACGTATCAGCAGCACCAAATCTGGATGTTTGTCCTTCCCCCAC

OS_scaffold51 ...A..T...C.......ATG.........T.TC.......------......GG.A.G--.............GG..CA..CA..C...T.........

10110 10120 10130 10140 10150 10160 10170 10180 10190 10200

....|....|....|....|....|....|....|....|....|....|....|....|....|....|....|....|....|....|....|....|

GT_scaffold4190 ATTGACAATAAGGGACAAAC--AGGATGGCAGCCACTAGCTTATGGTGTGTTGCTCCTAATAAGGTTACTGGTAAGTTTTTGTTTTGTTTTGCTTTGTTA

OS_scaffold51 .C...GT.C..A........TC........TA.G..C..........A..C..TC.T-------.C.C..AA....C....-----------.C......

10210 10220 10230 10240 10250 10260 10270 10280 10290 10300

....|....|....|....|....|....|....|....|....|....|....|....|....|....|....|....|....|....|....|....|

GT_scaffold4190 ACAAAACC--TAGAAATACTAAAGAAGCTCAGCTCTTGATGGATTTCTCTGAGTTCTCCTGAAGGAGAAACAAGAAGTCGTTTCTCAAACACTGGAGTCC

OS_scaffold51 ......ATAA..........G..T..A-...A...A..G.T.........CGA......................TA.T.....C.C..T.T....C..T

10310 10320 10330 10340 10350 10360 10370 10380 10390 10400

....|....|....|....|....|....|....|....|....|....|....|....|....|....|....|....|....|....|....|....|

GT_scaffold4190 TAATCTTGTCACACTCCTCAGTCTTGTTCCACAGGATGTGTTCAGCATTTCTTTGGACCAGTTTCAGGC-TTTTCCTCATATTCCTCCTCTTCTCTCCTG

OS_scaffold51 ........C........A..A......G...AG.A.CA.......G.............T..C......A....TT.A...............C...T..

10410 10420 10430 10440 10450 10460 10470 10480 10490 10500

....|....|....|....|....|....|....|....|....|....|....|....|....|....|....|....|....|....|....|....|

GT_scaffold4190 CCACATAGATGCACCTTACAAAGACTTTTGGCTTTTACTCTAGGGACCAGATAGATCAGAG-------------AAATAGCTATCTGTCTAGTGCATAAG

OS_scaffold51 ....T....A................G.-...........C....T.......CG.A.AT.TTTTGTTCTGCTA..TC..GGGAG.A...........GA

10510 10520 10530 10540 10550 10560 10570 10580 10590 10600

....|....|....|....|....|....|....|....|....|....|....|....|....|....|....|....|....|....|....|....|

GT_scaffold4190 TATCAACATTTCCTCAGATTCACAGGATGGAGGAGCTTAGGCAGTGGCTATGAAAATGGTGAGAAATGAGACTAACTTCTTCCCCTTTTCTTGAGCCTCC

OS_scaffold51 ..G..TA.......TG.G.....G..C.A.....A........C.........................C.........C...T....C.........T.

10610 10620 10630 10640 10650 10660 10670 10680 10690 10700

....|....|....|....|....|....|....|....|....|....|....|....|....|....|....|....|....|....|....|....|

GT_scaffold4190 CTGGTGTTGCTTGAGTGCTACTCTCTTGGCTTCAACAAGTTCTTGTCGCTTTTGCTAATTCTTTAGGCAAAACCTGACCTCAGTTGCTCCTTCCTTTTCC

OS_scaffold51 T.A.CA..............AA..A.......T.G.G....T.....AA........T....A.G...........GA.G..TACA..............

10710 10720 10730 10740 10750 10760 10770 10780 10790 10800

....|....|....|....|....|....|....|....|....|....|....|....|....|....|....|....|....|....|....|....|

GT_scaffold4190 CAGTGAGAGTAAAATGCTGCTGTCAGAGAAAGCGGTAGTGCTGTATTTGGAAGTTTTGGGAACCACTTGATTTACAGCTCTGAGATGGTAGGTCTCCTTC

OS_scaffold51 ..T....G...C.........A.........AT...G.....A......T.......T...GT..............T.......CT........T....

10810 10820 10830 10840 10850 10860 10870 10880 10890 10900

....|....|....|....|....|....|....|....|....|....|....|....|....|....|....|....|....|....|....|....|

GT_scaffold4190 CACTGCGTCTGCTGTTATCTTATCCTCATTTGATGGCCACCTGAGATAGTCTGACCATCTCCTTTTATCACAGGTGTGGTGGAAGAGGTAAGGAGAGGCA

OS_scaffold51 .....A...G..C..................C.............G..T......T.C...............A.A...........AAG........AG

10910 10920 10930 10940 10950 10960 10970 10980 10990 11000

....|....|....|....|....|....|....|....|....|....|....|....|....|....|....|....|....|....|....|....|

GT_scaffold4190 GAAGGGAATACTCATGTAATACGAAATCTGTTAACACTGACAGCCCAGCCTGAATGCTGCATTGGAGGCAGTGAATATAGCTTGCTGAATGTATCAAAAA

OS_scaffold51 ..........G...........A..........G..T...........GG......T......T................T.........A.........

11010 11020 11030 11040 11050 11060 11070 11080 11090 11100

....|....|....|....|....|....|....|....|....|....|....|....|....|....|....|....|....|....|....|....|

GT_scaffold4190 CAGTACAATGGATCGTGAATCCTTTTGTTCC-TGAGACATTAAAACCACTGTTTCTGTAAGCTTGGAAGGGTAACCTTTCAAGCTAAATATAAAAGTTAC

OS_scaffold51 TG......A..T..A..T.......C....TAC....AC...........CA..T.....A..CCA......T.........C.C...CT........G.

11110 11120 11130 11140 11150 11160 11170 11180 11190 11200

....|....|....|....|....|....|....|....|....|....|....|....|....|....|....|....|....|....|....|....|

GT_scaffold4190 TTTCAAATTGTAATCTTTCTGCACCCGGGAAATTTGAAGGCCTTACACTGACTCATCTTTATCTTGAGATAGACGATCAGAATATCCAGTCATAGCTATG

OS_scaffold51 ...............C.....AG..T.....T......A....G...............C......T...C----.......C.C........G.A.C..

11210 11220 11230 11240 11250 11260 11270 11280 11290 11300

....|....|....|....|....|....|....|....|....|....|....|....|....|....|....|....|....|....|....|....|

GT_scaffold4190 AATCCAATTTTAATAGGTGCTTTACAAATGCATAATGAAGAGCTTCTAAGTGTGAAACAACAGATGGAGAAGACTAACCAAGAGCACAAAGAAAAGAGAT

OS_scaffold51 G....C.........A..A........G.AT.G.G.A.......CG.....A...G....T......-.C.....G....G..AT.........TC...C

11310 11320 11330 11340 11350 11360 11370 11380 11390 11400

....|....|....|....|....|....|....|....|....|....|....|....|....|....|....|....|....|....|....|....|

GT_scaffold4190 GATATGCCTTAACTTTATAAGAACTTATGATAGTATTGCCACATTTTTATACTGGCAAAAGACAGTTTAAAGAAGAATTTGAAGGATGATAATGAGGTAA

OS_scaffold51 ...........G.........C.G.C..A....C........T.......G....G..C...........G.........................A...

11410 11420 11430 11440 11450 11460 11470 11480 11490 11500

....|....|....|....|....|....|....|....|....|....|....|....|....|....|....|....|....|....|....|....|

GT_scaffold4190 CTATTAATCTGTACTATAATTACAAGCATGCTACATAAAACCACAGTACTGGTTAGTTCTAGGAGGTTGGGTTGTCACATTTCTGGAAGATAAATGACTT

OS_scaffold51 ....C..........C......T.........................T.....CA......AG....A.A.....C..CG..........G..C.T...

11510 11520 11530 11540 11550 11560 11570 11580 11590 11600

....|....|....|....|....|....|....|....|....|....|....|....|....|....|....|....|....|....|....|....|

GT_scaffold4190 TGTTTTGACCAGAGCTTTCCTAAAATCAGAGGTTGTGTTCCAGACATTGTGGTTTGTAACCTGATGTCTCAACAGAGGGATTCCTGACGTGAGAAGGTTG

OS_scaffold51 .......T..G....................AC......T.....G........A.C...T......T...........G..............GA.C..

11610 11620 11630 11640 11650 11660 11670 11680 11690 11700

....|....|....|....|....|....|....|....|....|....|....|....|....|....|....|....|....|....|....|....|

GT_scaffold4190 TTTTATGATAAAATTTGGTAGTTTTTCCCCTTATGTATTGGTTAATGACTAATTCTGTAGTAATTTGGGTTTCAACCAGGGTCTAAGCCCACTAAGTTCA

OS_scaffold51 ..............C....CTG.C..T.T......C.A.......A..G.G.............A.........G.....C..AG.T..T...G.C....

11710 11720 11730 11740 11750 11760 11770 11780 11790 11800

....|....|....|....|....|....|....|....|....|....|....|....|....|....|....|....|....|....|....|....|

GT_scaffold4190 ATG-GATTAGAGTCAGACCTCATTCCTTGCTTCCCTTGTCACTGAATCTGGTCTCAGGAGCCACGTGATGCAAAAAAAGTGCGAGAGGTAAGCAGGAAGA

OS_scaffold51 G.AA....G.G..T.....CTCAG..C..T......A......................A.T..A.....T....--....T......G..A........

11810 11820 11830 11840 11850 11860 11870 11880 11890 11900

....|....|....|....|....|....|....|....|....|....|....|....|....|....|....|....|....|....|....|....|

GT_scaffold4190 TGAGGCAGGATTCCATTTCTCAGGATAGTTTTGCAGTAACTAATTTCCAAGTAGTCAAGAATTAAAAGAAAAAAAA-TCCAAACCTTATTTTGATGCACA

OS_scaffold51 .....T.A---..TG.......A....A.........G.T...C.........T......................G..........TC....G..GGG.

11910 11920 11930 11940 11950 11960 11970 11980 11990 12000

....|....|....|....|....|....|....|....|....|....|....|....|....|....|....|....|....|....|....|....|

GT_scaffold4190 AAGCAAAAAACTTGGGATGGAATAGTGGGTATGCCTGGGACAGAAGGCAATTTGAAGCAGGAAAATTGCCAGGAATTTATTTTCCTTTAAAGTCCCATGG

OS_scaffold51 .G......GT......GA...T...-....................AG.TC.........A.......T........AG.....T.....G...T.....

12010 12020 12030 12040 12050 12060 12070 12080 12090 12100

....|....|....|....|....|....|....|....|....|....|....|....|....|....|....|....|....|....|....|....|

GT_scaffold4190 TTTATTTTTGTTAAAAGTTTGTTTTT-AATCAGCTGGAGTACACATGGGTAGATTTCAGTGGAAACAAAAATTTGAGCACGAAAATAGATTATTTAGAAT

OS_scaffold51 .................CA.A.....T...G.........G...........G.....C......A.....A.......AA...T...............

12110 12120 12130 12140 12150 12160 12170 12180 12190 12200

....|....|....|....|....|....|....|....|....|....|....|....|....|....|....|....|....|....|....|....|

GT_scaffold4190 TACTGGTTTACAAAAGGGAATAAAATTGTCTAAACACCAAAATATGCCAGTAATAGCATTATGGCATGAAATATCCAGCAGAAAACTCCAAATCCTGCCC

OS_scaffold51 ............G....C..................T.......C....A..............T.......G....A.....GG...............

12210 12220 12230 12240 12250 12260 12270 12280 12290 12300

....|....|....|....|....|....|....|....|....|....|....|....|....|....|....|....|....|....|....|....|

GT_scaffold4190 AGAATATCCCACATCCTCAAGTCTACCAAACTATCAAAAAACCGATTGTGTCTTGGTAATTTAGTGGAACTAATAACAGTGCCCTGGAGCAAACTTTATT

OS_scaffold51 .......A........CAG...T.......A.GC..C.....TA.........................AC.................A........GC.

12310 12320 12330 12340 12350 12360 12370 12380 12390 12400

....|....|....|....|....|....|....|....|....|....|....|....|....|....|....|....|....|....|....|....|

GT_scaffold4190 GTACACTGGTCATAGTGTTGTAGATATGGGATTGCCTGATCCATGTTGTGTACTTGTGTACTTGCTGAATAGAGCTGTCATATTCATTGTTTTTTAAGAA

OS_scaffold51 .........G..C.....C.........A..C.....TG............G...................C....A.TG....AG..CA..........

12410 12420 12430 12440 12450 12460 12470 12480 12490 12500

....|....|....|....|....|....|....|....|....|....|....|....|....|....|....|....|....|....|....|....|

GT_scaffold4190 GGAGATTTTATTTTTAACCACAAATGTTATGGGAATATATATATGTTTTTAATGACACATTGTGAGACTGTGAAGATTGACTTGTTTTGGATGCATCATG

OS_scaffold51 CA.T.A..................A........GT...TAC.T.T....GT....................AG......C..A.G..........A....

12510 12520 12530 12540 12550 12560 12570 12580 12590 12600

....|....|....|....|....|....|....|....|....|....|....|....|....|....|....|....|....|....|....|....|

GT_scaffold4190 AGCTTTCATTCTGAACTGAAACCACTGCCAAGC-CAGAAG--TCAGTACAGCTTTGTCCTGTGTCACATAAATA-ATAAGAGAAAGTCCCCTTCTCCCTT

OS_scaffold51 ..........TG................AG...A...G..AG......................A...C.....G.......G..T...TT.........

12610 12620 12630 12640 12650 12660 12670 12680 12690 12700

....|....|....|....|....|....|....|....|....|....|....|....|....|....|....|....|....|....|....|....|

GT_scaffold4190 TAAGCACA-GGAAGTTCGTCTTCACAGAGGTGCTGGAATAGCAGTCCTACC-CCTTATTCTCGGGGCTGGATTGCACCCTGGCAGAGGGACTT-AGGGGT

OS_scaffold51 ...A....A.....G..A...............CA..T.............T......G.G.A..A....T............G.........T.....G

12710 12720 12730 12740 12750 12760 12770 12780 12790 12800

....|....|....|....|....|....|....|....|....|....|....|....|....|....|....|....|....|....|....|....|

GT_scaffold4190 TTCACTTCTGTTATGATCTACTCCAGTGAGGACAACTTCAGGGTGGAGGAAGATTTTATTTTGTCATCATTTCCATGTTCTTGCCACCTGAGACTTTTTC

OS_scaffold51 C...TCG....CC......G........G.....C..---..C....A...A.............T........T..............AG..G......

12810 12820 12830 12840 12850 12860 12870 12880 12890 12900

....|....|....|....|....|....|....|....|....|....|....|....|....|....|....|....|....|....|....|....|

GT_scaffold4190 TGCT--------ATTGTGGGTTGTTGGTATGGACCAACTGACACCATTTCCATGACTATCTTAATCTGCTGTGGTAGAATTCACCTCTCCTAGCTGTTTT

OS_scaffold51 ....GCTACATT......C....C............GA.A.T........T..............T..T.AG.................G..A..-....

12910 12920 12930 12940 12950 12960 12970 12980 12990 13000

....|....|....|....|....|....|....|....|....|....|....|....|....|....|....|....|....|....|....|....|

GT_scaffold4190 CTTCCTAAAATGTAGTCTTCTACTTGAACTTACTGCCTAGTAAATGGTGAGAAAAAAAGAGAGAGGGACTTTCCTAAAAGACTAACTAAGATAATTAGTA

OS_scaffold51 ..G........C.-.C.C.......A.......CCT........-ACA.G..GGG.G.A........G......C......T..C...............

13010 13020 13030 13040 13050 13060 13070 13080 13090 13100

....|....|....|....|....|....|....|....|....|....|....|....|....|....|....|....|....|....|....|....|

GT_scaffold4190 AAAGAAAATGAAACAAAACACAGTCTGGGTACAAATGCATCATGAACAGCCACAGAGTAGAAATTAGAAGAAAACTTATTGCAGTCAAAGAGTGAAATCC

OS_scaffold51 ......T............C..A.A...........T...T.....TG....................T.T---T..C.G..T.CA...........C..

13110 13120 13130 13140 13150 13160 13170 13180 13190 13200

....|....|....|....|....|....|....|....|....|....|....|....|....|....|....|....|....|....|....|....|

GT_scaffold4190 ATGAAGAAGGCAATGAGTAAAATGGACCTTGTTGTAG----------GTTTCTAAGACATGATACAAGAAATAAATGCTTGCAGCAGCAGGAGTTGAGCG

OS_scaffold51 ........CAG..CA..................T...CTGAACACCTA....C..A..GG..........G...T.....A......T.....G.....C

13210 13220 13230 13240 13250 13260 13270 13280 13290 13300

....|....|....|....|....|....|....|....|....|....|....|....|....|....|....|....|....|....|....|....|

GT_scaffold4190 TGTGCACTTCACCGTGGAAAATGGGTGAGTCAAGTATGTAAAACAGATGAGAAAAGAGAGATTTTGTAGTTGAAGCCAATCTCATCCTAGTTTTCACCAG

OS_scaffold51 ..........T..A...G.GG.....T..C..G......G......C....G.................C....AT......TG..A.G.G.........

13310 13320 13330 13340 13350 13360 13370 13380 13390 13400

....|....|....|....|....|....|....|....|....|....|....|....|....|....|....|....|....|....|....|....|

GT_scaffold4190 AAACACAGCACAGTTTGAGAGCAGCATAAGAAAAATA--TTTGAGTGGTCGGGCACTTTCAAGGATTTCACTCTCTTCCAGACAGAAAGCCATAAAGG-T

OS_scaffold51 ....................AT....C.G.G....C.CA..CA......T..C..GGGCG....GG.GG------.AGG...G.....C.TG......C.

13410 13420 13430 13440 13450 13460 13470 13480 13490 13500

....|....|....|....|....|....|....|....|....|....|....|....|....|....|....|....|....|....|....|....|

GT_scaffold4190 GATATTAAAGATAGCAAGGAGGAGGATAATCTAGATTGCCCTTCTGCCTAACAGCCTGTGCTATTCTATGGAGAAATGCACACCATAAAACAATGGCAAC

OS_scaffold51 ..GG.........AT------------------------............T.....A....G.C...........AA.....TG..C..A.........

13510 13520 13530 13540 13550 13560 13570 13580 13590 13600

....|....|....|....|....|....|....|....|....|....|....|....|....|....|....|....|....|....|....|....|

GT_scaffold4190 AGTGACATGTGAGGGCTGAGAGACACAACACTGATAAACTGGAAATCCCAGTAAATCAGAGAAAGAGATGAAAGAAGAGCAGAAATATGTGAAAAAATAT

OS_scaffold51 ..A..------..AAG...T.TGTG-.GA...........AAG....A..............--....A.................G..A.---------

13610 13620 13630 13640 13650 13660 13670 13680 13690 13700

....|....|....|....|....|....|....|....|....|....|....|....|....|....|....|....|....|....|....|....|

GT_scaffold4190 GTGTGTCAATTACAGAAGGATCAGACAGGGAAGGTCTTTTTGTAATCGTCCAAGTGGAATAAACTTTCCTTTTGAGAAAAAGCAAAGTG-AGTTAGAATT

OS_scaffold51 --...C....A.TG............G................C..T.A...................A....A......G..TCCA..C..C...----

13710 13720 13730 13740 13750 13760 13770 13780 13790 13800

....|....|....|....|....|....|....|....|....|....|....|....|....|....|....|....|....|....|....|....|

GT_scaffold4190 GCAGGTCAAAGGAAGAGGAGAGACCAAAGCACCACGCAGCTAGTAACTCAGACAAGGTTTTAGTGAAAGCAAGAGGTGAGAAAGGCAGGAACCAAAAACC

OS_scaffold51 -..AC.....CAGGTCATGAGAG.TG...TTG.GTT..A..C-.GCTGA.TG..G.A..C.........G..........G................G.A

13810 13820 13830 13840 13850 13860 13870 13880 13890 13900

....|....|....|....|....|....|....|....|....|....|....|....|....|....|....|....|....|....|....|....|

GT_scaffold4190 AAAGTGGGAATGATGCAGGAGTGGAGCCTGATGATAGAGAATGGAGTGCAGAAATATCAAGAGGGGAAAATGAAA-ACTGAACGAAGG-CTGTTCAGGAG

OS_scaffold51 C.---.....A.CC.......CA..........---......A...G..T....C....T..........CA...T...C..TA....G...C....TGA

13910 13920 13930 13940 13950 13960 13970 13980 13990 14000

....|....|....|....|....|....|....|....|....|....|....|....|....|....|....|....|....|....|....|....|

GT_scaffold4190 TAAAGGAAGGATGGAAAA---------------------------------------GGAGACACTCAGAACAGTTGAATATAGTCAAAA-ACAGTTTCT

OS_scaffold51 ............A..G..AAGGCACTTGGGAGGGCTGGCAGCAGCAGAAAGGGGATA...T....CT.A....A.CT......A......TG....C.G.

14010 14020 14030 14040 14050 14060 14070 14080 14090 14100

....|....|....|....|....|....|....|....|....|....|....|....|....|....|....|....|....|....|....|....|

GT_scaffold4190 GCAGCTGCAAAACTGAAGGGTTTTGAGAAAAATGTCATTGCAAAGAGAATTTATTGAAATATTGTCAACAGCAATAAGAATTGCTTTTTTTTTTTTTTTG

OS_scaffold51 T................A..C............A.....C......T...........G...........T..G........AGAGC....--------.

14110 14120 14130 14140 14150 14160 14170 14180 14190 14200

....|....|....|....|....|....|....|....|....|....|....|....|....|....|....|....|....|....|....|....|

GT_scaffold4190 GATTGTAAGAAGACATATATTTCACCTTCTCTAAATAGTTGTCTGGCCTGGAATAATGAATATGGTTTAATATGCTGATGTTTTGAATAAAAGGCAGCCT

OS_scaffold51 ..C..-----...................CTC........A.........C.GGT...T.G..CA...........C..A......TC............

14210 14220 14230 14240 14250 14260 14270 14280 14290 14300

....|....|....|....|....|....|....|....|....|....|....|....|....|....|....|....|....|....|....|....|

GT_scaffold4190 TGAGACACTTATTTTGATAAGTTTAT---GACAGAATGGGCTTCAGCGAA-TTCTCTT-------TTTCTAAAGTTTTATTGAAGGAAACTCCTACAGCA

OS_scaffold51 ..G...G.A....G....GG......CAC....AG..........AT..GC.....C.AAGATGT.A......AA...T...C........G.A.TT...

14310 14320 14330 14340 14350 14360 14370 14380 14390 14400

....|....|....|....|....|....|....|....|....|....|....|....|....|....|....|....|....|....|....|....|

GT_scaffold4190 GTGTATCATTCCAAGATTATAAGACATATATAAAATTATTTTTAAATAATATGAAGTTTGCAAGGCTT-GCTCACCTTGCAAAACTGCACTCAGTGT---

OS_scaffold51 A....CA.C.....A.........T.............C..........G........GA........CAT..T.........G...........T.ATA

14410 14420 14430 14440 14450 14460 14470 14480 14490 14500

....|....|....|....|....|....|....|....|....|....|....|....|....|....|....|....|....|....|....|....|

GT_scaffold4190 ------GAGTTTCAGGT----TTTCTGAGTTTCAA-TCTTCTGGCTTACATCTTCAACAAAAAAAAATCTTTAACTTGTAGCAACA---ATATGTGGTGG

OS_scaffold51 GTAATT..A.A.TGAA.GTCT.....A........A....T....................C....T......G...A..AAG.G.GTG...CA......

14510 14520 14530 14540 14550 14560 14570 14580 14590 14600

....|....|....|....|....|....|....|....|....|....|....|....|....|....|....|....|....|....|....|....|

GT_scaffold4190 AT-TTTTTGATATTATTTTATAGCAGTGAAAGACTACTCAGGGTATTCTG--CTTTTAGAATCACTCCTGTTGGAAACTATACATCAGGAAATAATCCTT

OS_scaffold51 C.C.......A............A.C....T......A.T.........TACT...C.........T....G.........................A..

14610 14620 14630 14640 14650 14660 14670 14680 14690 14700

....|....|....|....|....|....|....|....|....|....|....|....|....|....|....|....|....|....|....|....|

GT_scaffold4190 TGCAAAGCTGAAGACATAAACTTGGCCTGTCACTCTTTTGTTTTCCCCTGATTTGCTTGATTATATTTTGTTTTTCTCTTCAGTCACATTTATTTAAATT

OS_scaffold51 ....G............G............TG......C-....T..........T......T.G.......A............T.........T....

14710 14720 14730 14740 14750 14760 14770 14780 14790 14800

....|....|....|....|....|....|....|....|....|....|....|....|....|....|....|....|....|....|....|....|

GT_scaffold4190 TTTCCCTTTCCCTTTCCATCCTTTTCAAATTTTGTTGCTCTTATTTTTT-AAGTGTGTAAGCAGTGATGGAAGGGCTGGGTTCCAGCTGCTAGTTTAATT

OS_scaffold51 ..................A..............A..A............T..........A....A..............................C...

14810 14820 14830 14840 14850 14860 14870 14880 14890 14900

....|....|....|....|....|....|....|....|....|....|....|....|....|....|....|....|....|....|....|....|

GT_scaffold4190 TATCGAAATGTGCAACAATAGCTGAGAATCCCAGAGGCCATACTTGAAATCAAACCTCTGAAAAGTTTTTAAGGCATTTCTAGCTAACTGCTTGGAATCT

OS_scaffold51 ....A................................AT...........TG...T........AA.G..................G.......A...T.

14910 14920 14930 14940 14950 14960 14970 14980 14990 15000

....|....|....|....|....|....|....|....|....|....|....|....|....|....|....|....|....|....|....|....|

GT_scaffold4190 GTGTAGAACCAGATTTCTC-------------------------------------------AGCCAACTGATGAACGGATTTTCATTTTAAAAGACTTG

OS_scaffold51 ..A................TTATTGTGTCAAGCAATTGACATCATGCCCAACAGAAAAATAA......T........ATG..............AG....

15010 15020 15030 15040 15050 15060 15070 15080 15090 15100

....|....|....|....|....|....|....|....|....|....|....|....|....|....|....|....|....|....|....|....|

GT_scaffold4190 TTGGGAAGGAATGGTATTTTGTAAAAGCTTTCTCAGAGGCCTCTGTATAAATTTAATACTTTTTTAATGCTTGAGATTATCAAACATCTTACCATTGAAA

OS_scaffold51 ...AA..A..GCA......CA.....C...GT.T.....T..T.A.....G..A....A....C.....GA....GA.GC..GC..C..--..CCCA...

15110 15120 15130 15140 15150 15160 15170 15180 15190 15200

....|....|....|....|....|....|....|....|....|....|....|....|....|....|....|....|....|....|....|....|

GT_scaffold4190 GCATGAAATATAGGTTGCCCTGTTCTAGGAAGTCTCCTGTCTTAGAAATCTAAATAACTACCAGGATAGTAATTATTTTTTTTTCTATGATGCTCCCTGC

OS_scaffold51 A.C...G..........T.................T...............GT......T---AAG..T...AA.....-----.........AG.....

15210 15220 15230 15240 15250 15260 15270 15280 15290 15300

....|....|....|....|....|....|....|....|....|....|....|....|....|....|....|....|....|....|....|....|

GT_scaffold4190 ATATTTGAAGAGATTACCATTACTGTTTCCATCTAAAGCTTGTCTTAGTAGCATATCTGAAAAAAACAGAAAAATTATTTTCTTGCCATCACTTAAGCTT

OS_scaffold51 .A...CC..AG-------...C.A.G.A..........G----.....A....C.C.........G----.C..A.....C.C.....C...C....T..

15310 15320 15330 15340 15350 15360 15370 15380 15390 15400

....|....|....|....|....|....|....|....|....|....|....|....|....|....|....|....|....|....|....|....|

GT_scaffold4190 AACAATAGATGTTGTGAATGAAATTCTGGCCCTATCCAGGAGCCCCCTGGCAAAGAGGGCCATGGATTTAGAGCAATAGCCTGCTCTG------------

OS_scaffold51 ....GC......CAC.G..........A...A.............T...A...G......TG..TT.C..CG..........C.C.CACTATTGTCTCTC

15410 15420 15430 15440 15450 15460 15470 15480 15490 15500

....|....|....|....|....|....|....|....|....|....|....|....|....|....|....|....|....|....|....|....|

GT_scaffold4190 ------TACTTAGGCAATAGCTTCTCCTGC--ACCAGTCTCCCTTACTTTTGCAAACC------TGTGCATATTC-CCTGGCT-----TTTCTGATG---

OS_scaffold51 TTAGTT.....TT....-.C..GTG...A.TC..TG.CT.TT......GA.TG.G..TGTGAGA.C..A......T....T..GAGTA.GC...G..CTG

15510 15520 15530 15540 15550 15560 15570 15580 15590 15600

....|....|....|....|....|....|....|....|....|....|....|....|....|....|....|....|....|....|....|....|

GT_scaffold4190 ----GACTCAATGGGAATGACAAATA--ACCATTCTGTACTCCAGATAA-------TCTGCACTCTCTGTGTTCATAACTCCAAAACCAAATGAAGTTAT

OS_scaffold51 CTGCA.A.G..GTA......A..T.GCTGT..A.GGAA.TGA..A...GCCGTCCT....T.....A.....CT.......T....T.....A.......

15610 15620 15630 15640 15650 15660 15670 15680 15690 15700

....|....|....|....|....|....|....|....|....|....|....|....|....|....|....|....|....|....|....|....|

GT_scaffold4190 AGAGGTTGCTGCATACTCTAAGGAATTTTATGTTAAATCATATTACATTTCTTTATTATGTCTGTTCATGGCTTCATTCATTTTGTATTCTCTGAATACA

OS_scaffold51 ................A..............C...CT...........A...C..GC.G.....C..G.A....TG........A..............C

15710 15720 15730 15740 15750 15760 15770 15780 15790 15800

....|....|....|....|....|....|....|....|....|....|....|....|....|....|....|....|....|....|....|....|

GT_scaffold4190 AGGCCAGG-TCATTCTCTTGTGTAATTTCGTAAAAACTTGAATTCCTACCAGACCTGAAGTTGGATGGTGGTCAGCTGGGAGGTGAATGATTAATATCCA

OS_scaffold51 ........AC........G...C......A..............G.......GG...........C....A..............TG............T

15810 15820 15830 15840 15850 15860 15870 15880 15890 15900

....|....|....|....|....|....|....|....|....|....|....|....|....|....|....|....|....|....|....|....|

GT_scaffold4190 GCTTAAAGCCTTGAAGAGATGGAAGATAAAGAGAAAGCTACAAGTGGAGCTGAGAAATTCATTTCATGCTATTGAG-GATAGCTTGGACTGCCCCACCTC

OS_scaffold51 ...C....G..................G........T.C.G......GAA......G.A............C...TC.......C........T..T.C.

15910 15920 15930 15940 15950 15960 15970 15980 15990 16000

....|....|....|....|....|....|....|....|....|....|....|....|....|....|....|....|....|....|....|....|

GT_scaffold4190 AACTTCTTGATCTGCCTATATC------------------------------------------------------------------------------

OS_scaffold51 .G............TT.G...TTACTCAAAATACATTTTAAATGCCACGAAAATACATTTTATGACAGTTCTCAGTAGGCCTAAAAAAATGAAGCCAGTT

16010 16020 16030 16040 16050 16060 16070 16080 16090 16100

....|....|....|....|....|....|....|....|....|....|....|....|....|....|....|....|....|....|....|....|

GT_scaffold4190 ----------------------------------------------------------------------------------------------------

OS_scaffold51 AAATATGAACACAGACAAATGATTGAAATACAATAAGAGTGGAGAAATGCACAATAAGACCACTGTGGATCTTTATCAGAAACATATGGTTTTCTCCAGA

16110 16120 16130 16140 16150 16160 16170 16180 16190 16200

....|....|....|....|....|....|....|....|....|....|....|....|....|....|....|....|....|....|....|....|

GT_scaffold4190 ----------------------------------------------------------------------------------------------------

OS_scaffold51 GAGTGAAGTGACCGAGTTGAATCAGCATTGGAAGCGGTAGGAGAAATATCTGGGGATCTAATGAGAAACAGTTCTTCCAAAATGAACAATGTCATCATCC

16210 16220 16230 16240 16250 16260 16270 16280 16290 16300

....|....|....|....|....|....|....|....|....|....|....|....|....|....|....|....|....|....|....|....|

GT_scaffold4190 ----------------------------------------------------------------------------------------------------

OS_scaffold51 AAATCCATCCATTCATCCTGAAAAGATCTGTTTTATGGTGCAGAACTAGGATTTTGTTTCTTTTTTTTTCCCTCTCTCTCTTTTGTTGGCAGACTAGAAA

16310 16320 16330 16340 16350 16360 16370 16380 16390 16400

....|....|....|....|....|....|....|....|....|....|....|....|....|....|....|....|....|....|....|....|

GT_scaffold4190 ----------------------------------------------------------------------------------------------------

OS_scaffold51 GTTTTCTGAAAGCTCAAAATAATGACCGTCTCCTATACATTAAAGTGTCAAATCTGAAAAAATTGTAATATTTGGGGTGGGGCAGGGGAAGAGAAGACAT

16410 16420 16430 16440 16450 16460 16470 16480 16490 16500

....|....|....|....|....|....|....|....|....|....|....|....|....|....|....|....|....|....|....|....|

GT_scaffold4190 ----------------------------------------------------------------------------------------------------

OS_scaffold51 CCAAAATGACCCGGGGCTGAATATGAGACCATTTATAACCAATGCAGAGCTGGCCAATACTGATGAAAGAGCTGCTCTTCCAAACTAATTTTCCAAGAAA

16510 16520 16530 16540 16550 16560 16570 16580 16590 16600

....|....|....|....|....|....|....|....|....|....|....|....|....|....|....|....|....|....|....|....|

GT_scaffold4190 ----------------------------------------------------------------------------------------------------

OS_scaffold51 GTGCCCCTCCTACCTAGGATGCCCACTGCAGATGTTTTTCTATACCCTTTCTTTCCAAGAGGGAGCAGAGGACATTTTGCACAGGATTCCGTAATTTCTT

16610 16620 16630 16640 16650 16660 16670 16680 16690 16700

....|....|....|....|....|....|....|....|....|....|....|....|....|....|....|....|....|....|....|....|

GT_scaffold4190 ----------------------------------------------------------------------------------------------------

OS_scaffold51 TTCTTGCCTGAGGTGCATCTCTCTCATTCTGCCTCTCAATCACCAGCCCCTCTGAGCTACTGAAAGGCAGCTTGCTGACCTGTGCAAGCTGTGTCCCTGT

16710 16720 16730 16740 16750 16760 16770 16780 16790 16800

....|....|....|....|....|....|....|....|....|....|....|....|....|....|....|....|....|....|....|....|

GT_scaffold4190 ----------------------------------------------------------------------------------------------------

OS_scaffold51 ATGAGAGATGAAGAGATGGCTGGTGGTGACCCAGCTCTAAATGGCTAGGGCCACCACCTGGAACTGGGGTCATCCATGCCTTCCTGCTCCAATTGTTTTT

16810 16820 16830 16840 16850 16860 16870 16880 16890 16900

....|....|....|....|....|....|....|....|....|....|....|....|....|....|....|....|....|....|....|....|

GT_scaffold4190 ----------------------------------------------------------------------------------------------------

OS_scaffold51 TCTACACTTAAACTAATGATTCATAATGAATCATTAAAGTGCTATTCCCATAGGCAGAGGGAGAAGGGGTGCTGTATTCTCTTTCTTCTCATAGGAATTA

16910 16920 16930 16940 16950 16960 16970 16980 16990 17000

....|....|....|....|....|....|....|....|....|....|....|....|....|....|....|....|....|....|....|....|

GT_scaffold4190 ----------------------------------------------------------------------------------------------------

OS_scaffold51 ATCTAAAATATTTCTTTTAAAGGTGAATAGGCCTCAATTCAGGGAGATGTGCATTTAAGTTAGAGGTACTCCTGGTTGCATCCTTTTTTTTTTTCCCACT

17010 17020 17030 17040 17050 17060 17070 17080 17090 17100

....|....|....|....|....|....|....|....|....|....|....|....|....|....|....|....|....|....|....|....|

GT_scaffold4190 ----------------------------------------------------------------------------------------------------

OS_scaffold51 CAGAAATTCTTTCTAGGTGAATTCTGGAGATATTTTACTTTTGCTTAGGATCCTCCAGAAGTTTGGTAGCTTCTAGCAGGTTGAATACTACTGTGCAGGC

17110 17120 17130 17140 17150 17160 17170 17180 17190 17200

....|....|....|....|....|....|....|....|....|....|....|....|....|....|....|....|....|....|....|....|

GT_scaffold4190 ----------------------------------------------------------------------------------------------------

OS_scaffold51 AAAGTAGCTCCAGCTAGGCTGCTGTACATGTGATAGCTGTAGGTACATTCATAATGCATGCATGGATCAGCTGAAGAATTCATGCACAGTAAATCCAGTG

17210 17220 17230 17240 17250 17260 17270 17280 17290 17300

....|....|....|....|....|....|....|....|....|....|....|....|....|....|....|....|....|....|....|....|

GT_scaffold4190 --------------------------------------------------GCTCAGTGCTATAATAGAAATCATGCAAAGTACAATGTTCTAAAACGGAC

OS_scaffold51 ATGTTGGGAAATATCAGGCTTGCTGTGCATATGTGATATACAAATATACT....................C.....T....G.G...........T..C.

17310 17320 17330 17340 17350 17360 17370 17380 17390 17400

....|....|....|....|....|....|....|....|....|....|....|....|....|....|....|....|....|....|....|....|

GT_scaffold4190 GCATTATTTTCTCATAATCCTGAAGAAAA--CAGA------------------TTTTATTTATATTTATACAGAGGCCATTTATCCAAAGCATGCTTTGT

OS_scaffold51 AAG......CT.............A....AA....ACAGAAGGATCTGAAATTC.C.GC..TAT...C..A.........GC.......A.....C...A

17410 17420 17430 17440 17450 17460 17470 17480 17490 17500

....|....|....|....|....|....|....|....|....|....|....|....|....|....|....|....|....|....|....|....|

GT_scaffold4190 GATCTGCCAATCACCACAGGTAATTCCTCAAGTTTTTCTGAGTTACTCTGTCTAATGCAATGCCTTCGAGATAAACAACTACAGCATTGGATCAATGCTC

OS_scaffold51 ..............AG...A........T...CC..CA........CA.A..........CCT....T.A...TGTG......A...A.A.......TC.

17510 17520 17530 17540 17550 17560 17570 17580 17590 17600

....|....|....|....|....|....|....|....|....|....|....|....|....|....|....|....|....|....|....|....|

GT_scaffold4190 TAAATCAGGAAGGAAAAATACTGCTGAAGAGCAGCGTTCTCTTCTACTATCATCTCTGCTTAGATGTGTTTCTTATGA---AGTGTCATCTTGATTATTA

OS_scaffold51 ..................................-AC........G.....---......C...AA..........A.TGG............G....G.

17610 17620 17630 17640 17650 17660 17670 17680 17690 17700

....|....|....|....|....|....|....|....|....|....|....|....|....|....|....|....|....|....|....|....|

GT_scaffold4190 AACTATCAAACTATGAAATAAAAAAAACACTGAGCATCTGATGAATAAATAATGTGAAAAATCTACTGCCTGGGGATGAGCTGGTCAGACTCCAGATTTC

OS_scaffold51 ...AC.AG...A..A...G.......C.--.....T.......T..G.G.....CC................A.....G........A..GT.......T

17710 17720 17730 17740 17750 17760 17770 17780 17790 17800

....|....|....|....|....|....|....|....|....|....|....|....|....|....|....|....|....|....|....|....|

GT_scaffold4190 TTTGCCACTTGCTTTCTACATATTTTACATCTCACATATCTTCCATCTTCATAGACTTTGATATTAAGAAGGGACAA-TGTGACCATCAGATCAACTCTA

OS_scaffold51 G....----..T..AGCG.CC.GCA.GGG....C.....TC.T..G.........A...C.C..G..C..AA...C.C....G....T.....GG..A..

17810 17820 17830 17840 17850 17860 17870 17880 17890 17900

....|....|....|....|....|....|....|....|....|....|....|....|....|....|....|....|....|....|....|....|

GT_scaffold4190 CTGCAACCTATATCCTAAGACTCTATCTGCCACTGATTTTCAGAAAAACACCTCCCATTTTATTACTCTGAATTGCATCCATTTTGATCCGCGTTAATGA

OS_scaffold51 .CT..T.A.............A.......T.............G.............C.........G...G..................A.A......T

17910 17920 17930 17940 17950 17960 17970 17980 17990 18000

....|....|....|....|....|....|....|....|....|....|....|....|....|....|....|....|....|....|....|....|

GT_scaffold4190 GCTTAACTGTTCATGTTCCCAAATTATTTGTCATTTTGTAGGTAGTTGTATACATTTGGACAGCAGCTTTGCTTACAAACAGTA-TTTTTCTATTATCTT

OS_scaffold51 .....G......TGTCC.....................C..A..........A....TA.........AC.T....C..A.A..A.......G....T.C

18010 18020 18030 18040 18050 18060 18070 18080 18090 18100

....|....|....|....|....|....|....|....|....|....|....|....|....|....|....|....|....|....|....|....|

GT_scaffold4190 TTTGTTCATGTTTTAAGATACGCATTAATGCTGAATAAAGCATAAATTCTTACTCACGATCTGGAGTTACTCCCTGTGATTTCTGAGAAACTAGTTTT-A

OS_scaffold51 ....................TTG....G......G.....A.G........G....TT....A........TT....A...........GTC......T.

18110 18120 18130 18140 18150 18160 18170 18180 18190 18200

....|....|....|....|....|....|....|....|....|....|....|....|....|....|....|....|....|....|....|....|

GT_scaffold4190 ATTTTTTTAGCACAAAAACATTTTTGGTGCATTCAGATACCAATGTGATGATTAGTGTAATGGTATGAATATAATAGCCTAGTATTAGGAAATATGCCAT

OS_scaffold51 ......C..ATC.....G...............T....-....C.......CAGA.....GA.........G........G..T...T...G........

18210 18220 18230 18240 18250 18260 18270 18280 18290 18300

....|....|....|....|....|....|....|....|....|....|....|....|....|....|....|....|....|....|....|....|

GT_scaffold4190 GTGAACAATGCTTTTAGTAGTCTTAATTTTTGGAAGTTTAAAAGACATGCACTGTTTCATGTAGGAATCATTTCCACATATTGAAAAAAAATGTTAGTTT

OS_scaffold51 ......C..AT.....A.........AC......C......--...C.A..G........................G........G....G.........

18310 18320 18330 18340 18350 18360 18370 18380 18390 18400

....|....|....|....|....|....|....|....|....|....|....|....|....|....|....|....|....|....|....|....|

GT_scaffold4190 TCCCAGAAATGTTCAAGAAAAGTTGACTTTTATATAACTATGAAAAAATGTGCTGCTATGTGCAAAAATCTTTAAAAGAAGTGCTAATTTATATGTCTTT

OS_scaffold51 ..T....................................G........................G.....C.............C.......--......

18410 18420 18430 18440 18450 18460 18470 18480 18490 18500

....|....|....|....|....|....|....|....|....|....|....|....|....|....|....|....|....|....|....|....|

GT_scaffold4190 TCCCCTAATATTCCTACAATGGTATTTATTTTCTTAAAGGAAAGAGCCAATATGTTTGCCTTGCCCAGAAATGTTTATTTGAAAGCACTTTCTGAAAAGC

OS_scaffold51 .T......A..............................A......A........C................A..................T.......T

18510 18520 18530 18540 18550 18560 18570 18580 18590 18600

....|....|....|....|....|....|....|....|....|....|....|....|....|....|....|....|....|....|....|....|

GT_scaffold4190 AAATATGTTGATTTAATCAGTACATTGATTT---------AATAACAAGTGCAAGGGTTAATAATAATGGTGATGATAACATCAAAAGTCCAAGTGTTTT

OS_scaffold51 .......C......T....A..T..G.....CACTGAAGA..........C......C.....T....AA...........AT.G........A..G...

18610 18620 18630 18640 18650 18660 18670 18680 18690 18700

....|....|....|....|....|....|....|....|....|....|....|....|....|....|....|....|....|....|....|....|

GT_scaffold4190 AGCTGTGGTTAAATTTA-GACCCTCGGGGAAAACTGGACCTTGTCAAATGAGGAAAAAAAAATGATTTGTGTAAACCATGAGAAAAAACATTGCCCTAGA

OS_scaffold51 ....A........A...A....A.G...TG..GT.A..G.............--....G..C.........C....TG............G..T.....G

18710 18720 18730 18740 18750 18760 18770 18780 18790 18800

....|....|....|....|....|....|....|....|....|....|....|....|....|....|....|....|....|....|....|....|

GT_scaffold4190 TTTGCTTTTATGT-------TATTCATCATTACTTGGATATCCACATGAAATGGGTGAGAATGCTCACAATGGAAAATGAAAGATTTTTGGGACATCATT

OS_scaffold51 AG.AT........ATTATGT........G...T...............G......-----..TT...T....A............C..............

18810 18820 18830 18840 18850 18860 18870 18880 18890 18900

....|....|....|....|....|....|....|....|....|....|....|....|....|....|....|....|....|....|....|....|

GT_scaffold4190 TTTTTCATCTGAAGATTCATAATGGAAGTGCACTTTTATTTTTCACTGATATCCAGATTTAAACATTTGTCACTGATTCAGGGA-CGGAAACAACGACAT

OS_scaffold51 ....-........A..............G........G.......TCAG...................................GT........T.C...

18910 18920 18930 18940 18950 18960 18970 18980 18990 19000

....|....|....|....|....|....|....|....|....|....|....|....|....|....|....|....|....|....|....|....|

GT_scaffold4190 ATGGTCTGAGTGGCCAATCATACAGCACAAAATAAACAACAAGAAGCGGTCAGAGTGAACATCTGGTGAACATCCCAAAGCTGAAAATGAGCCTGATTAT

OS_scaffold51 .....T..............C...T.......-----.........T........C.......................T..........T.......G.

19010 19020 19030 19040 19050 19060 19070 19080 19090 19100

....|....|....|....|....|....|....|....|....|....|....|....|....|....|....|....|....|....|....|....|

GT_scaffold4190 TCTATGAATATTTATGAGTAAAAAACAATTTAATAATGAGTGAGTCCACTAGTCCACAAACTGTTCACAGTGAGAGAAGTCATTACACATGCAACCAATA

OS_scaffold51 .....................................A..C..A....T...A......G..........G.......A.A......T.A....T.....

19110 19120 19130 19140 19150 19160 19170 19180 19190 19200

....|....|....|....|....|....|....|....|....|....|....|....|....|....|....|....|....|....|....|....|

GT_scaffold4190 TTATGTTGAA-GAGTAATCAAATTAGCTAAAATTTCAAATACTTCTGCTTCTAACAAGCTTGTAGCTCTGCTGGACTTCTTTGTATTTCTGAATCTGGAC

OS_scaffold51 ....T.....TA.......T......T.C...C.............AG.............AAT........A....C....A.........G......A

19210 19220 19230 19240 19250 19260 19270 19280 19290 19300

....|....|....|....|....|....|....|....|....|....|....|....|....|....|....|....|....|....|....|....|

GT_scaffold4190 TCCGAAGACAATCTTTTCCGAAAGATTTAAAATCTCTATTTATAGTTTGTTCTATATTGTATTCACAACCATTGCACAGCTAAGAAAGAACTAGAAACTT

OS_scaffold51 ...T..........C.G..AG.......TT..A..G.G....G.T......A...........GT.T.A.......TCA......G....T....C.A..

19310 19320 19330 19340 19350 19360 19370 19380 19390 19400

....|....|....|....|....|....|....|....|....|....|....|....|....|....|....|....|....|....|....|....|

GT_scaffold4190 TATATTGCCATGATTAGAGTATATGTCATAGACTTTAGAATGCAACAAGCAGTTTAGGATACCAAAACACAATTGTACATGAATCTAAGTGTGCAGCAAA

OS_scaffold51 ..CG...G....C.......G...C...........G....A..G...A.......C....T......T..G.......CATG.AG.........A....

19410 19420 19430 19440 19450 19460 19470 19480 19490 19500

....|....|....|....|....|....|....|....|....|....|....|....|....|....|....|....|....|....|....|....|

GT_scaffold4190 GGAAGGAGAAGATGGATTCTTTGGAACTCAGTATCGATTGAAGCCTGACTGGACATACCTAAAAGATTTCCCTCAGTGTATTGAAAACTTTCAATAACTG

OS_scaffold51 ...........G..A....................T...C.......................G.....ATT..T.CA...GC..........G...A.A

19510 19520 19530 19540 19550 19560 19570 19580 19590 19600

....|....|....|....|....|....|....|....|....|....|....|....|....|....|....|....|....|....|....|....|

GT_scaffold4190 TAAACACAAAAAACTTGATGAGGTAGAGAGACTCACTGAGTCTTGATAAGAAAATTCTTCAGTAAACCTGTAAAAGAAAACTGAAAAACAAAC-AACTAA

OS_scaffold51 ....T...G.........GAG.T.................CA..C..................G.C...--.G..........C...CA..TTTG.T...

19610 19620 19630 19640 19650 19660 19670 19680 19690 19700

....|....|....|....|....|....|....|....|....|....|....|....|....|....|....|....|....|....|....|....|

GT_scaffold4190 A-AAGTGTTGGAAGCAAGCAGAAAGAATGATGAAATACAAGGCTTCAGTCACTTTTCAGTCAGACATTTTGCTGATGACTTTGATGGAGAAATTCTATCA

OS_scaffold51 .C.....C........G...A..GC.GCTT.......................CAGTCAAT.......................C...........GC..

19710 19720 19730 19740 19750 19760 19770 19780 19790 19800

....|....|....|....|....|....|....|....|....|....|....|....|....|....|....|....|....|....|....|....|

GT_scaffold4190 T----TTTCCTGAGCCAGGGATTCAGCCTTAATATTATCCCACCAGCATGCAGACGACATGCCGTTACCACATGTACAAGACTTACATGGAACTGGCATT

OS_scaffold51 CGGAC..CA...G.......T.........T...........T..C.G.A.....AGT..T.TA........CAGG...A......C....CA.......

19810 19820 19830 19840 19850 19860 19870 19880 19890 19900

....|....|....|....|....|....|....|....|....|....|....|....|....|....|....|....|....|....|....|....|

GT_scaffold4190 GCACTGCGATTTCAATTTCATGTTATATC-------TCCACTGTGTTA---CTCCCTTGGTGCCTTTCACTTTTCCCATCACCTATCATTTGGGCTGCTT

OS_scaffold51 ......A..............C..G.C..CATTGCA.GTCT.....C.TGC......CA..AT.....GT.....T.....A.....C....T....A..

19910 19920 19930 19940 19950 19960 19970 19980 19990 20000

....|....|....|....|....|....|....|....|....|....|....|....|....|....|....|....|....|....|....|....|

GT_scaffold4190 TACCATGTCAAACAGGAGCGCTATAGAGCAATGTCTGTCTCTTGTTAAATCTGGTGCATTACTTAACACCTTGGGGCTTGAAAATCTCTGAAGAATTTAG

OS_scaffold51 GC....C.T.........T.....G....G......C...............T..A..............................A......C......

20010 20020 20030 20040 20050 20060 20070 20080 20090 20100

....|....|....|....|....|....|....|....|....|....|....|....|....|....|....|....|....|....|....|....|

GT_scaffold4190 ATACCATTTCTATATGAATAATAACAACTATAATGCTATTTTTTG-ATAAATCTAAGGTATCTTGATGTAGGGATTCATCAAGCTCCCTTGCAGGA-CAA

OS_scaffold51 T.G.T.......C...G.C.G...T..T....G...C...C....G.G...........GG....C..C.T...G....G......TT........G...

20110 20120 20130 20140 20150 20160 20170 20180 20190 20200

....|....|....|....|....|....|....|....|....|....|....|....|....|....|....|....|....|....|....|....|

GT_scaffold4190 GGCATATACTTTT--AAGTAGACATTTAGTTCTCAGTACTGGCTTCAACTGACATTACAAGCCCATTCAAAAATGTATGTATTCTGAATTTCTACAAAAA

OS_scaffold51 ........T....TT..AGG..TG....A.....T..........TC.G...T........T....G............C....................

20210 20220 20230 20240 20250 20260 20270 20280 20290 20300

....|....|....|....|....|....|....|....|....|....|....|....|....|....|....|....|....|....|....|....|

GT_scaffold4190 TAAGTTTGTTTTCTTTAAAAATAATGAGTTTATACTAGTTATTTTTGGTGATGTTTCAGACCGAAATAACACTCTAACTAGCTGCAGAAAGTCTTGTGGC

OS_scaffold51 ...................................G.....................................A..........................

20310 20320 20330 20340 20350 20360 20370 20380 20390 20400

....|....|....|....|....|....|....|....|....|....|....|....|....|....|....|....|....|....|....|....|

GT_scaffold4190 TCACACCACAGCAAATTTTAAATCTT-TGGAGTCAATGGTGCAGTTTTCTCTTAGAAAAATAGATTAGAATAAAGAACTTCCTAGATTTGTTTTCCCAAG

OS_scaffold51 .G............G...........A....................C...................A........G...................T...

20410 20420 20430 20440 20450 20460 20470 20480 20490 20500

....|....|....|....|....|....|....|....|....|....|....|....|....|....|....|....|....|....|....|....|

GT_scaffold4190 CCAGGCTTGGGGTGTGACCAACCTAAAGCATTTTCTGAAACCA----CTCATTTTGTGGGTAGAATAATGTTAAAATCTATTGAAATCACACCATTTTCT

OS_scaffold51 ....................G.......T.G......C.GT..AACA...C.......AA..................AG......C....T........

....|...

GT_scaffold4190 ACAGAAAT

OS_scaffold51 .......A

**Supplemental Data S2**

Alignments of the orthologous scaffolds, including viral sequences that matching *Parvoviridae* which are shaded and their flanking regions. The dot denotes the same nucleotide aligned; the dash denotes deletion. GM, Golden-collared manakin, (*Manacus vitellinus*); ZF, zebra finch (*Taeniopygia guttata*); LE, little egret (*Egretta garzetta*); DP, Dalmatian pelican (*Pelecanus crispus*); AC, American crow (*Corvus brachyrhynchos*); and RF, rifleman (*Acanthisitta chloris*).

**(i) ePaV *Manacus vitellinus* and ePaV *Taeniopygia guttata***

10 20 30 40 50 60 70 80 90 100

....|....|....|....|....|....|....|....|....|....|....|....|....|....|....|....|....|....|....|....|

GM_scaffold270 AAAAAATTGTCT--TTCAGGTGATAACCAGATGTGTTACATA--CATGTTGAGTATAAGTGCTGAAAACCAAGTCACAGGCTGTACTGTCTTAAGTGGAG

ZF_chr4 ........T..CCC.CT.AT.TG....T.CT.T.AC..T.CCAT.TAAC.CTC.G.G.C....TGTTC.ACCT...A..TTG...ACTCA..CCA...CT

110 120 130 140 150 160 170 180 190 200

....|....|....|....|....|....|....|....|....|....|....|....|....|....|....|....|....|....|....|....|

GM_scaffold270 -AGATCTGAATTCATGCCTGTGTGCA----TTCATCAATTTTTTAAGAACTT-------AGATTTGAAAT--------GTATTTGAAGTTTGTTTCAA--

ZF_chr4 C.A.CTCA..A...CAG....T.T..GCTGCCTG...GGG.C.A..ATG...CTGACCA..GCC..G..CCTCTGAAAA.G.C...G.GACA...TG.GT

210 220 230 240 250 260 270 280 290 300

....|....|....|....|....|....|....|....|....|....|....|....|....|....|....|....|....|....|....|....|

GM_scaffold270 ---------TGTATTTATTGGAATTTCAGGAAAAAATTCCCTTTGGTTCCTGGATTCACCAGAGCTGATTTTGTGTCATACTGGTTTTGTTAGTTAATTT

ZF_chr4 TCTGACATC.......C......A..........T.C.....---------.......T..............--..T...........C..........

310 320 330 340 350 360 370 380 390 400

....|....|....|....|....|....|....|....|....|....|....|....|....|....|....|....|....|....|....|....|

GM_scaffold270 GGTTTTGCCTCTGAATCTGAGAAG-------TATATTCTCTCAGGTGTATGGAGGCATTT------------GTAGTGCAGTGGTGAACAACAACATGCA

ZF_chr4 .C........G.C..A..A...G.AGAGCTG.G.G.......T.-....G.........CAAAAGAAAACAG.C................G.........

410 420 430 440 450 460 470 480 490 500

....|....|....|....|....|....|....|....|....|....|....|....|....|....|....|....|....|....|....|....|

GM_scaffold270 AATCCAGCATTGCACTACAGCTTGT---TTCCTAGTTTTAAATATTTATTTATATTTATATCAATCAGTCTTGTTCCTCTGCTAGATTTCCCTCTAGTGA

ZF_chr4 ......A.TG..GG......T..A.GTT.C...GA.....CC.............C.--------------------...............C.......

510 520 530 540 550 560 570 580 590 600

....|....|....|....|....|....|....|....|....|....|....|....|....|....|....|....|....|....|....|....|

GM_scaffold270 CAAACGAAGAGATCTCTGCACTCCCAACTGTGAATGTGCTCCCTGGAGGCAAGTACTCAGGAGCCAAATTGAAGTCAGTGATCAGGATGTTGCGTGGATT

ZF_chr4 ....T....................T..G.......................A..T................................A...........

610 620 630 640 650 660 670 680 690 700

....|....|....|....|....|....|....|....|....|....|....|....|....|....|....|....|....|....|....|....|

GM_scaffold270 GCTGGAACAGGGAGTCCCTTCTAAAGAGATTGAAGTGAGTAACTACTTGTCCCTTAAAGTCCTTTTTTCATGTATGAAACAAGAATAGAATGGCTGGATC

ZF_chr4 .............................................T......T..............C..C-..........A.................

710 720 730 740 750 760 770 780 790 800

....|....|....|....|....|....|....|....|....|....|....|....|....|....|....|....|....|....|....|....|

GM_scaffold270 TTCACTGCCTTGAATACCACAGCTCTTTAATTGGCAGCTCAAAAGGCATGAAAAGAAGTAACACCAAAGACTTCTCTCCCTTCCCCCCACTAGCTGCTGA

ZF_chr4 A.................................A..........AG.........CT.G.......................T...-..A.........

810 820 830 840 850 860 870 880 890 900

....|....|....|....|....|....|....|....|....|....|....|....|....|....|....|....|....|....|....|....|

GM_scaffold270 GACATAAGATTAGTTTGAATTCAATTCAATTCAAAAGTATGTTTACATCATTCTTGGTTCTGGTAAATTGCCGAGTGAATATGATGTTAACAGGCATAGA

ZF_chr4 ..G..G....C............G.A..---T..GT..-.T..............CC............A..AT.......--....A............

910 920 930 940 950 960 970 980 990 1000

....|....|....|....|....|....|....|....|....|....|....|....|....|....|....|....|....|....|....|....|

GM_scaffold270 CAAGTAAATAAAGGCTGTGAAACCTAGCTGCTGAATGTGAAACCAGTCTTTGCAAAGTCTTAAGTCAGCCATGTTATATGATCTCTCTTTTGGGAGAATG

ZF_chr4 ...--------..........GT.C......................T...T..GT.................C..C.........T.....A.......

1010 1020 1030 1040 1050 1060 1070 1080 1090 1100

....|....|....|....|....|....|....|....|....|....|....|....|....|....|....|....|....|....|....|....|

GM_scaffold270 AGATAAAATATTTACTGTATAGTAGGTGGGGGTT---------TTGTTTTGTTTCGTTTCATTTTCTTAGCAGTGTAAAATTAGATTCCTTCTTACAACT

ZF_chr4 .................-.....GT......T..GGTTTGGGG..T....TG..AA............G..T.....G.---..........C.GTG...

1110 1120 1130 1140 1150 1160 1170 1180 1190 1200

....|....|....|....|....|....|....|....|....|....|....|....|....|....|....|....|....|....|....|....|

GM_scaffold270 GCATTATTTTGCATCGCCACATTAGGATTTGATTTTTAACAATTCTATTTGTTAATGACTTCATCATATAATTCCTCTTAACACTTGTTACTGTTCAGTG

ZF_chr4 ..............AT..............A........TT.....G.....----.....G..A.C....CCA......CT.-..A....CAA.T.C..

1210 1220 1230 1240 1250 1260 1270 1280 1290 1300

....|....|....|....|....|....|....|....|....|....|....|....|....|....|....|....|....|....|....|....|

GM_scaffold270 TTTTCCTAATGCATGTGTAATTTCTTCTTTACAGAACCTTCAGGAGTTAAAACCTCTGGATCAGTGTTTGATAGGACAGGCAAAGGAGAACAGGAAAAAG

ZF_chr4 ...-..C....G.CA.......A.C.............................................G.T.....A..G.............G....

1310 1320 1330 1340 1350 1360 1370 1380 1390 1400

....|....|....|....|....|....|....|....|....|....|....|....|....|....|....|....|....|....|....|....|

GM_scaffold270 AACAGATATAAAAACATACTTCCTTGTAAGTCCTAACTAATCTGAAGTTCACTGTTGATATTCTGTTACCAGCTCTATGGTCAGATGGATTAGATCAGAA

ZF_chr4 ......................................C..T....A.....A.....C......CC...GC..G................G...A...T

1410 1420 1430 1440 1450 1460 1470 1480 1490 1500

....|....|....|....|....|....|....|....|....|....|....|....|....|....|....|....|....|....|....|....|

GM_scaffold270 GTCAGATGA------TTTGATTCTCCATCCTAGTAGGGAAAAAATTAAAGCAAGCATTTATAAAGCAGTTTGAGATCAGTGAATAATATATTATTGTATT

ZF_chr4 .GTT....GGGGTGG.......TCTA.........A...TG...G............................--........C.........C...GC.

1510 1520 1530 1540 1550 1560 1570 1580 1590 1600

....|....|....|....|....|....|....|....|....|....|....|....|....|....|....|....|....|....|....|....|

GM_scaffold270 GTAGTAGTTAGGGATAGATGTTCACAGACTTTTCTGTTGTGTCAGCAGATCCTGTGTCTTAAGTGATGCATACATAGGGAGCCAGATAAGATTTTTTT-A

ZF_chr4 ..G...TG.......G..................A.....T.T.....G..GCA........A.C...T......GT.C.A.....C...G.......C.

1610 1620 1630 1640 1650 1660 1670 1680 1690 1700

....|....|....|....|....|....|....|....|....|....|....|....|....|....|....|....|....|....|....|....|

GM_scaffold270 GCTTTACCCATGTAAAATTGGAAAACCAGACATGTATACTATTGTCATTAAATTTCCTGCCATAAAATATTGTTAGGTGTGAGATGTGTCAAGAAGTAAG

ZF_chr4 .T..........C.G..........A....A....G......G....G....................TC..C.---...T..--....TG........A

1710 1720 1730 1740 1750 1760 1770 1780 1790 1800

....|....|....|....|....|....|....|....|....|....|....|....|....|....|....|....|....|....|....|....|

GM_scaffold270 AACTGACAAAGATTAGTTCTGAATGAGAAATATT-AACCTCAGTTTTTCAGAGGTGCTGCAGTTCAAATCATGCATCCTGCCTGTCTGATGATTTGTATT

ZF_chr4 ........G..G........ATG.......AGC.C.G....T...........---------.........CTT....CA........C..T...A....

1810 1820 1830 1840 1850 1860 1870 1880 1890 1900

....|....|....|....|....|....|....|....|....|....|....|....|....|....|....|....|....|....|....|....|

GM_scaffold270 TCTCCTCTAGATGTCTT---CTTTATTCGAGGTACTGATTAATATCCTAAATCAAGTATTTAAGTGATTTATTTTTTTGA-GTAAG----------ATCT

ZF_chr4 ......GC...CA....TTT.......AA.A.........GG.G........T.T..............AT..C..C.A.A.C...CTGTGCAAGC...A

1910 1920 1930 1940 1950 1960 1970 1980 1990 2000

....|....|....|....|....|....|....|....|....|....|....|....|....|....|....|....|....|....|....|....|

GM_scaffold270 GTGGAGATAAGTTCTATGTGAATGTGTAAAGCTCA-CATCTCTATTGGTTGAAGGTCTAATTATGTGCACACATAACATGAGGTCTTTTTATTGTGTGGG

ZF_chr4 ............G..G.....G.......G.T...G..G..G.....C..........G..G.CA.......C...............C.G.........

2010 2020 2030 2040 2050 2060 2070 2080 2090 2100

....|....|....|....|....|....|....|....|....|....|....|....|....|....|....|....|....|....|....|....|

GM_scaffold270 AATTGCAGATGACACCACTAGAGTACCTCTTGGGATTGAAGGTGGATACATCAATGCCAGCTTCATTCGCATGCCGGTGGGGAATGAGGAGTTCGTTTAC

ZF_chr4 G..GA.............C.....C.....C...G........................................C........................

2110 2120 2130 2140 2150 2160 2170 2180 2190 2200

....|....|....|....|....|....|....|....|....|....|....|....|....|....|....|....|....|....|....|....|

GM_scaffold270 ATTGCATGCCAAGGACCTCTTCCTACTACTGTAGCAGATTTCTGGCAAATGGTTTGGGAACAAAACTGTACTGTGATTGCCATGATGACTCAGGAGGTCG

ZF_chr4 ....................................................................................................

2210 2220 2230 2240 2250 2260 2270 2280 2290 2300

....|....|....|....|....|....|....|....|....|....|....|....|....|....|....|....|....|....|....|....|

GM_scaffold270 AAGGAGAAAAGATAAAATGTCAACGTTACTGGCCAGATGTCCTCAATAAAACCACCATGATAACTGATAGACTACGCCTTGCTCTTGTGAGACATCAGCA

ZF_chr4 .G..............G.....G.................G......................A...C.........T.G.............T......

2310 2320 2330 2340 2350 2360 2370 2380 2390 2400

....|....|....|....|....|....|....|....|....|....|....|....|....|....|....|....|....|....|....|....|

GM_scaffold270 GCTGAAGGGCTTTATCATTAGAGTGCTGGAGCTTGAAGAGATTCAGGTAAGTGAATCTAATTCTGCATGCTTAGAGCTTTTT-CTGGTTGTTGGCATATT

ZF_chr4 ............C.....C..............C..............G..................C....G...T.....T...AG.A...C......

2410 2420 2430 2440 2450 2460 2470 2480 2490 2500

....|....|....|....|....|....|....|....|....|....|....|....|....|....|....|....|....|....|....|....|

GM_scaffold270 TATCCTTATACCTCTAGCTTGCCTGCTGTCAACTGCATTGTCTGCATCAAGAGGTCAAACAGGACT-------------------GCATGACTGGGCAAC

ZF_chr4 ....T......T...G....T......T...CT.C........A..........CA.CC...AG..TCTCCTCTTTGCCTAGTGA....T..C.....GA

2510 2520 2530 2540 2550 2560 2570 2580 2590 2600

....|....|....|....|....|....|....|....|....|....|....|....|....|....|....|....|....|....|....|....|

GM_scaffold270 ACTTCCTCATTGCGGCTTGTGCTACTGCGTGACCTTT-TAG----------TTGGGCAAGGAGATGATTTCATTATGCTAATGAGACTCTAAATTTTGGC

ZF_chr4 TG...T....G..A.........G..TT.........A...GAAAAAGCTT......C...CT............CAG......................

2610 2620 2630 2640 2650 2660 2670 2680 2690 2700

....|....|....|....|....|....|....|....|....|....|....|....|....|....|....|....|....|....|....|....|

GM_scaffold270 TTTGTTTGGCCACAGAAACATATATAGGGATATGAAATTCACTGATAATTCAGTCTGGCTGGTTGATTCCTCTGTGTGATCAGCTCAGTCTGCACAGTGA

ZF_chr4 .CCCA.............T....G............-......A.....G...C............C.................................

2710 2720 2730 2740 2750 2760 2770 2780 2790 2800

....|....|....|....|....|....|....|....|....|....|....|....|....|....|....|....|....|....|....|....|

GM_scaffold270 ACGTTATACTGTTAAATTACTTGCTAGTTATTTTCAGTTTAGCTCGTTGTTTCTCTTTTCTTATCGCAATACAGAACATCTTCTTTGGGCCAGTCCCATT

ZF_chr4 ..C...C..AT.......G...A..........C........T..-------------..G...T.A.T....A.G..C....AC.....ATA....TC.

2810 2820 2830 2840 2850 2860 2870 2880 2890 2900

....|....|....|....|....|....|....|....|....|....|....|....|....|....|....|....|....|....|....|....|

GM_scaffold270 CTTCTGAGATTTTTCTTTGGGAACTTTCTCT-TTTCTTTTTATTCTTGCCATGTGTTACATGTTGTTGTTGTTTTTGAACCTCAGACAGGTGAAGTACGG

ZF_chr4 G........C..........A....CAA.T.C...................CAA.CC.A.C.G..G..............................G...

2910 2920 2930 2940 2950 2960 2970 2980 2990 3000

....|....|....|....|....|....|....|....|....|....|....|....|....|....|....|....|....|....|....|....|

GM_scaffold270 CACATTTCCCACCTGAACTTCATCGCCTGGCCTGACCATGACACCCCTTCTCAGCCAGATGACCTGCTCACTTTCATCTCTTACATGCGGCACGTCCACA

ZF_chr4 .......................T..............C........C..G........C..T........C....................G.......

3010 3020 3030 3040 3050 3060 3070 3080 3090 3100

....|....|....|....|....|....|....|....|....|....|....|....|....|....|....|....|....|....|....|....|

GM_scaffold270 AGTCGGGACCCATTATAACTCACTGCAGTGCAGGCATCGGGCGATCGGGGACCCTTATTTGTATAGATGTTGTTCTGGGGTTAATCAGCAGAGACCTCGA

ZF_chr4 ..G.A........C..C..C.....................A.G..A..C.....C..C..C.....C..G..C......C.C.................

3110 3120 3130 3140 3150 3160 3170 3180 3190 3200

....|....|....|....|....|....|....|....|....|....|....|....|....|....|....|....|....|....|....|....|

GM_scaffold270 TGTGAGTATCCCAGAGGATGGGCTCAGTTGTGCTTTTGACCTGTAGGAAGTATGTTCCATTGAAGCTATTCAGTTGCTTAAAGAACATGCCATACTTTTG

ZF_chr4 ...........-.TG........C....A.....CC.......C.......G.......C....T.G..G..A.CTT.......GTG.A....G..G...

3210 3220 3230 3240 3250 3260 3270 3280 3290 3300

....|....|....|....|....|....|....|....|....|....|....|....|....|....|....|....|....|....|....|....|

GM_scaffold270 GCAGTAGCACACTGGACATGTGTTAATGTGTCATAAAGCAGGGTGTAAAACCTATGAAATGTTTCTTCATTGATGACCCCAAGAGAGAAGAGCTCTTATA

ZF_chr4 ......TT--------------------------------------------------.........TGC..G...T.T....T.G.G........A...

3310 3320 3330 3340 3350 3360 3370 3380 3390 3400

....|....|....|....|....|....|....|....|....|....|....|....|....|....|....|....|....|....|....|....|

GM_scaffold270 TCCAC-TGGTCCTTGGGTTAGCTCAGGGGATCTTAAGTGGCTTTGGCAATAGATGGAAAAGTTAAAAATGCCTGCCTTACCTGACTGTGTGTACTCAGTG

ZF_chr4 ...C.G...C..............G..CA.C..C.-.CA..CAG.C..C.T..A.T.T.--..GC..GGT.A.CTT......T---------------CT

3410 3420 3430 3440 3450 3460 3470 3480 3490 3500

....|....|....|....|....|....|....|....|....|....|....|....|....|....|....|....|....|....|....|....|

GM_scaffold270 GAACTTTCATGTTAAATAGCACTGGGGCTGAGCACTGTGGCATTCAGTCAGTCAGAGATCCATGGACTGTTTCAGGATAGCTAGAAGCTAACTAGAGTAT

ZF_chr4 .G....C---------.G....AT..CA..G.A.AAA...TG..G--------------------------C..T...G...------------------

3510 3520 3530 3540 3550 3560 3570 3580 3590 3600

....|....|....|....|....|....|....|....|....|....|....|....|....|....|....|....|....|....|....|....|

GM_scaffold270 CTGTTTAGGCAACTGAATCAAAAACTCAGTGTACTACAAACATCTCAGATGAGGGTATGTAAATTAACTTCTCCAGCACAACCAGGGATATGCTTTCTAG

ZF_chr4 -----.T.....T..-------------...G.-----------------AG...CG.............T....A........A....-..........

3610 3620 3630 3640 3650 3660 3670 3680 3690 3700

....|....|....|....|....|....|....|....|....|....|....|....|....|....|....|....|....|....|....|....|

GM_scaffold270 GATGATCCACAGGAGGGTGCCAGCTTCCTTATGTTTCTGTCCTTCAGCAGCCTGATGTAGCAAATCTTTTGGTGAACTTGTAGAGAAGATGAACTACGGT

ZF_chr4 ..CA................T....C..........A................AGG........A..G.-......T..C..A.........G...T.-.

3710 3720 3730 3740 3750 3760 3770 3780 3790 3800

....|....|....|....|....|....|....|....|....|....|....|....|....|....|....|....|....|....|....|....|

GM_scaffold270 CATGCTTAGCTTAGTGAGGTTACTAAGTACCCTCTAAATGTCTAGTACTTTATAGATCTGGGGAAATAAAAAGAGTTTTGTTAGGTTGCACCAAGGGGCT

ZF_chr4 ...........C....GA...T.....C.......G.......GAA.......-----------------------------------------------

3810 3820 3830 3840 3850 3860 3870 3880 3890 3900

....|....|....|....|....|....|....|....|....|....|....|....|....|....|....|....|....|....|....|....|

GM_scaffold270 CAACTTTCTGCACATTCAGCTCCCAATAAAAGTAAGCAAGAGGACCGGTGCACAGAGAAAGAAAACAAGAGAAGGATAGTGTCTGATGTAGGAGGGCAAC

ZF_chr4 ----........TGC.T....T------GG.A.........A.G..T...T.....A.G..C.C.AG.A.AGG..GG...............---..C..

3910 3920 3930 3940 3950 3960 3970 3980 3990 4000

....|....|....|....|....|....|....|....|....|....|....|....|....|....|....|....|....|....|....|....|

GM_scaffold270 ATCCAGATTGCCCAGAGACAGCACATGCTGGTTTTCTACCAAGCAGGCATAGGCTACGGTGAATCATTTATTTCACTACTAATGCACCTGGTTCTACTCC

ZF_chr4 .................................AC......GAT.AA........G.A......T...C......T........T....AA.C.C.T...

4010 4020 4030 4040 4050 4060 4070 4080 4090 4100

....|....|....|....|....|....|....|....|....|....|....|....|....|....|....|....|....|....|....|....|

GM_scaffold270 TTGGCTTGATGCTGGGGATGTTTCTTTTCTATGCATGTATGTCTTTTTCAAGTTCTTAACTTGCTGCTTGTTTTGGTAGTTTGACATCTCAGACCTGGTG

ZF_chr4 C..TT.................C....GT.C.......C.........TG.A.GT...........T..............C........G.........

4110 4120 4130 4140 4150 4160 4170 4180 4190 4200

....|....|....|....|....|....|....|....|....|....|....|....|....|....|....|....|....|....|....|....|

GM_scaffold270 CGCACGATGCGTCTGCAGAGACACGGAATGGTTCAGACAGAGGTGAGTCTGGCTTCCCCAGGTACACACTGAACCAGCTGACAAGCCAGAGAACTTCCTG

ZF_chr4 .....C..............................................A.........CCTT..........T....AGT........G....--A

4210 4220 4230 4240 4250 4260 4270 4280 4290 4300

....|....|....|....|....|....|....|....|....|....|....|....|....|....|....|....|....|....|....|....|

GM_scaffold270 TGATGCACACAGTGATTGTCTTCGTGCATTCTAATGCAACGTTTGTGTCACAGGAATTAAATCCCATGAGTAGCAGTATATATTTAACAGTTAGAAACAC

ZF_chr4 ............CA........T.....C.G.....TG..AC..........A....C.G.-----..........G..G.G.C.....T...T......

4310 4320 4330 4340 4350 4360 4370 4380 4390 4400

....|....|....|....|....|....|....|....|....|....|....|....|....|....|....|....|....|....|....|....|

GM_scaffold270 AAGCCATTTTTAACATGCTGTACTGTTTTGATCTTTGTTTAAAAAAAAAACAATAATAGCAAGTTTTCATCTTCAAAAGCATGTGTTTATAG--------

ZF_chr4 .....................GT...........G...............-----..-.TG......................C........AGGGGGGT

4410 4420 4430 4440 4450 4460 4470 4480 4490 4500

....|....|....|....|....|....|....|....|....|....|....|....|....|....|....|....|....|....|....|....|

GM_scaffold270 ---CTGACCTTTTATGGCTAAAAACTGCATTACAAACTGAAGAGAAGGCCTAACTATAGCTGCTATGAATCACCGATACTACCAGAGATGTCTTGAAATC

ZF_chr4 CAG..A.G.....T...T.T...GT.......T.G...........A........G........GG......AT....----------........G...

4510 4520 4530 4540 4550 4560 4570 4580 4590 4600

....|....|....|....|....|....|....|....|....|....|....|....|....|....|....|....|....|....|....|....|

GM_scaffold270 TACTTTGAGGAGATATTCAACCAGAAGTTAAATGAACCTTTTAAGAAAAATCTAGATCTAGAACTAATTAACTTTACATGAGAGATGGTCATCACAAGGT

ZF_chr4 .CT....GTA.T.......G.........C...----......G...T.....G........G.............-----........T....G.....

4610 4620 4630 4640 4650 4660 4670 4680 4690 4700

....|....|....|....|....|....|....|....|....|....|....|....|....|....|....|....|....|....|....|....|

GM_scaffold270 CTTGTTAAAATCTGCAGTTTTT-CTTTGAATATGAAGGAATTATGAAGTCATAGTTTAAAAAAAC---CCCTGAAATTAAACCACTAAATTATCAGCCCC

ZF_chr4 ................C.....T........GG...A.....G.............A.......AAAA.........C......G....C.........-

4710 4720 4730 4740 4750 4760 4770 4780 4790 4800

....|....|....|....|....|....|....|....|....|....|....|....|....|....|....|....|....|....|....|....|

GM_scaffold270 ATCCCAACCTCAAAGCCCAAAACATGTATTAACCTCTACGTCTATATCACTGAGATTAAGATAAGAAGTTGCACTGTC---TGAGT------TCTGTATT

ZF_chr4 .....T......C.C..........C...C.G..C.C..AC..C.......C........G.........C.......CTC..G.ACAGCCA..A.....

4810 4820 4830 4840 4850 4860 4870 4880 4890 4900

....|....|....|....|....|....|....|....|....|....|....|....|....|....|....|....|....|....|....|....|

GM_scaffold270 AGTTGGCA--------AGACATTGAAATTCACTTCCTGTCTTCATTTCTACT-----------------CTCCTTTAGGTGTTTTTGGTATGAAAGGAGG

ZF_chr4 ........TTGATGCA..T.C.......A.....G..............G..TATGTAAAGTGCCTAGT............G.......G......A.AA

4910 4920 4930 4940 4950 4960 4970 4980 4990 5000

....|....|....|....|....|....|....|....|....|....|....|....|....|....|....|....|....|....|....|....|

GM_scaffold270 ATAAATGTCCCATACAAAAATATATCTTTAGTGGCAGAGGAGTATCTGTATTTGGTTTAAGTTGAGGTTGAATATTTCTCCACTGTGGCCTTTTT-GTTT

ZF_chr4 ..............TG...-.......................G...A.....TT...............C......T.................T....

5010 5020 5030 5040 5050 5060 5070 5080 5090 5100

....|....|....|....|....|....|....|....|....|....|....|....|....|....|....|....|....|....|....|....|

GM_scaffold270 GTTCGGGATTTTTTGTTTTCTTTGTGTGTTGTTTATTATTATTATTA----TTGTTGTTGTTATTATTATTTAATAGTAGAATAAGTGTCGAAATCAGGT

ZF_chr4 .G.T..T-.........G.TG....T..GG..C.T..G..T.CT..GGGAT...G..A..G.GG.T..C..........T...T...A.T....----..

5110 5120 5130 5140 5150 5160 5170 5180 5190 5200

....|....|....|....|....|....|....|....|....|....|....|....|....|....|....|....|....|....|....|....|

GM_scaffold270 GCTGTACAACTGTGAAGTGCACATGAGAGCACTTAGGTGGTGCGGGCATTTTTCACAT-TTCTGTAATTTCC-----CTTGCAGGATCAGTACATTTTCT

ZF_chr4 ........G............AG................-...A..............G.............TTTTC...A...................

5210 5220 5230 5240 5250 5260 5270 5280 5290 5300

....|....|....|....|....|....|....|....|....|....|....|....|....|....|....|....|....|....|....|....|

GM_scaffold270 GCTATCAAGTTATCCTTTATGTCCTGAATCATCTCCAACATGAAGAACAGCAGAGAGACAAATAAAATACTGAAGCAGAAGCGCTCATAAAATAAGGAAG

ZF_chr4 ...........G................C..A.....GG........A.A.................C.......---...T..ATT.............

5310 5320 5330 5340 5350 5360 5370 5380 5390 5400

....|....|....|....|....|....|....|....|....|....|....|....|....|....|....|....|....|....|....|....|

GM_scaffold270 CAAGGAGGTCAAGAAGCTCTCAAGTGTTTTATTTGTTCTGCCAATAGTTTTCTAGTCCCTTTCTTCAGTTGCTGTCAGATGTCTTTTCTATAAAGTCTCT

ZF_chr4 .........TG...G...T.GG..CA..........CT................A.....................CA...C.........G......T.

5410 5420 5430 5440 5450 5460 5470 5480 5490 5500

....|....|....|....|....|....|....|....|....|....|....|....|....|....|....|....|....|....|....|....|

GM_scaffold270 CCATCATTCCAATTAAGGTATATAATTACAAGGGAAAGGGATAGCTCGTACAATACCACTAATGTCTTCAGCCACATGGACACATAGGTGAGATTTTTGT

ZF_chr4 ............A...C.......G..TT..................A............GT.A.........C......TG.CC.T.............

5510 5520 5530 5540 5550 5560 5570 5580 5590 5600

....|....|....|....|....|....|....|....|....|....|....|....|....|....|....|....|....|....|....|....|

GM_scaffold270 ATAACAATATTTAATAAAGCTGCATTTTTTTTGCAGCTTCCTTAATATGTCATAAAAATACAATAAATAAATAAAAATTAAAATGTTTTAATTTAAAAAA

ZF_chr4 .............................--..T.......................T------------.C.G....A.....A..............G

5610 5620 5630 5640 5650 5660 5670 5680 5690 5700

....|....|....|....|....|....|....|....|....|....|....|....|....|....|....|....|....|....|....|....|

GM_scaffold270 GAATTCAA-GAAGAA--AAAAAAGTAATCTTATAGTACTTCCTGCAGTTCTGCAACTGTTTTGGGATATTTTTTTCTTACAGAGTAGGGGAGTGGGAGGG

ZF_chr4 A..AGT..T......GA...........TC....C.......................C......G.....G......T...GC.G...A..........

5710 5720 5730 5740 5750 5760 5770 5780 5790 5800

....|....|....|....|....|....|....|....|....|....|....|....|....|....|....|....|....|....|....|....|

GM_scaffold270 ATGAAGCAGTATTTTTGTCCTGGTAAACTTTATTACAAAGACATTTTACTAGTCTCCCTTTGAAAGCTTGTATATGGCACAGAATTTTTGCCCTTTTAGT

ZF_chr4 ................A..T..-...................T.............T-.............TC......................C....

5810 5820 5830 5840 5850 5860 5870 5880 5890 5900

....|....|....|....|....|....|....|....|....|....|....|....|....|....|....|....|....|....|....|....|

GM_scaffold270 ACGATTACAGCTGTTTTATAGAGGGTATCTTGAAATCAGCATTTAAGCTTTAAGAGATGCCTGTTAGCATAAGGATAGTAGTGAATGTTAACTGTATCAC

ZF_chr4 ..A....G....A..............................A........................................................

5910 5920 5930 5940 5950 5960 5970 5980 5990 6000

....|....|....|....|....|....|....|....|....|....|....|....|....|....|....|....|....|....|....|....|

GM_scaffold270 AGTTAATAATGTTTACTTCATAGAATTTCATGTATATATTTATATACTTTTACA--AAAAAAAGG--CTGAATTATTGGGTGTCTAACCTTATAACTGAT

ZF_chr4 .....---....................T.........................CA.........AT..........T.................T....

6010 6020 6030 6040 6050 6060 6070 6080 6090 6100

....|....|....|....|....|....|....|....|....|....|....|....|....|....|....|....|....|....|....|....|

GM_scaffold270 TAGGCATCTCCACTCTGTAAATAAATAATATACCTAATGTACACTGATCGGTGGGTCTGGGTATTTTTGTGTCCTTGTGACTGATTTTCCTTGGCCTTAC

ZF_chr4 ..........T..............-.......................AA...........................A.........T.........C.

6110 6120 6130 6140 6150 6160 6170 6180 6190 6200

....|....|....|....|....|....|....|....|....|....|....|....|....|....|....|....|....|....|....|....|

GM_scaffold270 ATGGCATCATTCATTGTGGTGGCTGCCCTTCTTAAGACATATGCAACAGAAGGGTCTGTGCACGTTGTAAATTTTCAGTATCCTTCTGT--------GTG

ZF_chr4 ............C...CA..T......TC.---........--------....--.......TA..T...G...C....G.....T...TCTGCAGTA.C

6210 6220 6230 6240 6250 6260 6270 6280 6290 6300

....|....|....|....|....|....|....|....|....|....|....|....|....|....|....|....|....|....|....|....|

GM_scaffold270 CATTTGAAAAAGTCA-CAGAACTGGGGGTTCCTTTCTGAAGAGTTTCCCCACTATGGTGAAAGGAAGAGGATATTTTCCTTCCTTCCTTGTTTTTCAGCA

ZF_chr4 TG.........TCTGG....G...T...C.........G...A.....A.......A.....-----..TT.C......C...C................

6310 6320 6330 6340 6350 6360 6370 6380 6390 6400

....|....|....|....|....|....|....|....|....|....|....|....|....|....|....|....|....|....|....|....|

GM_scaffold270 TACCTGGGCATTGAGACCATTTCACTTTTTTGGAGGGGTGTGTTTGTACTTGTTTGCACTACAGCAATGATCTGACATGATTGCAGTGATTACAACAGTG

ZF_chr4 C.T.................A..........A.-.A..........C...-------G........G..T.......-----------------......

6410 6420 6430 6440 6450 6460 6470 6480 6490 6500

....|....|....|....|....|....|....|....|....|....|....|....|....|....|....|....|....|....|....|....|

GM_scaffold270 TAATCTACTCTGCTTACAGGAACAAGGTTTGAGGTGAGAGGCAGGATGTCTGGGTTCTAAGTAGCTCAGGGGTTTAGTGATACTGTAAACAGTCAAAACT

ZF_chr4 .............C..GG.C...C...C.......TG......A...A..-A.A...............T..........C..........A...C....

6510 6520 6530 6540 6550 6560 6570 6580 6590 6600

....|....|....|....|....|....|....|....|....|....|....|....|....|....|....|....|....|....|....|....|

GM_scaffold270 TTTCTGAAAGGAAAATGTCATTTTAGGTTCTGTAGGAACTTTGGATGGTGAATAACTGCCATCAGCCAACACAG--CAGCCTCGTGTTTGAGCAGTATTC

ZF_chr4 G......T..A..TT.T........A.C.....G...G.C...............A...........TCG.GT.GT..TTTGA.G.C...G...A....T

6610 6620 6630 6640 6650 6660 6670 6680 6690 6700

....|....|....|....|....|....|....|....|....|....|....|....|....|....|....|....|....|....|....|....|

GM_scaffold270 CTGTCCCAGCAACCCTTGCCAGAAGAGAAGCTTTTGACTTGTGCTATCAGGAAGGGTATAAGAGTACTAATTTAACAGCATTCTGCATTGAAGTAGTGGA

ZF_chr4 ..C.........TT....A.....CT........-----......G...A.......G.......G.....A......................G...A.

6710 6720 6730 6740 6750 6760 6770 6780 6790 6800

....|....|....|....|....|....|....|....|....|....|....|....|....|....|....|....|....|....|....|....|

GM_scaffold270 AATAAGGTTCAGAGGCCTGTGGTTATCCAAGGTTTTGACTCTTCAAGATAGTAGTCCACAGTGTTCCAGTGTCCCAGAACTGAAACAACCACTGTCTTAC

ZF_chr4 .G.C.A-.........TG..TA....T................T..A.......C.....................A......GG.G.......CA..G.

6810 6820 6830 6840 6850 6860 6870 6880 6890 6900

....|....|....|....|....|....|....|....|....|....|....|....|....|....|....|....|....|....|....|....|

GM_scaffold270 TTGGTTAATGGTATTCTAGCTGTCAATAGTATGACTTAAAAAAGGAGAAGAGCCTTTTTCACTTGTCAGAGTAATACTGTGTTTTTTATTCCCTTCAGCA

ZF_chr4 ------------------------..G..C................A.................T...--.C..........A...........C.....

6910 6920 6930 6940 6950 6960 6970 6980 6990 7000

....|....|....|....|....|....|....|....|....|....|....|....|....|....|....|....|....|....|....|....|

GM_scaffold270 TAACAGCATAATTGTGTGTTTTTCAGAGCAGCATAGTTTGAGAAAGGAAAATTATTTCTGTGTAGGTAGAAGCAGAGGCAAATTGACCAGCTGTGTTGAC

ZF_chr4 ........A.......CA....A.......C.T.........G...........A....A.....A.....C......T.......TT....A.....G.

7010 7020 7030 7040 7050 7060 7070 7080 7090 7100

....|....|....|....|....|....|....|....|....|....|....|....|....|....|....|....|....|....|....|....|

GM_scaffold270 AGCACACTTGTATGAAGGAGCTTTAAATTGAAGGCAGAAGATTTTTCAGGACTGTGGTTGTCAACAGGCAAAGTGAGTAAATGCAGTTAGTTGATACACT

ZF_chr4 ..T.T.TC.A............A..........AA.A...........A...AACA...A.AT.........A.A......CA...C.....A.......

7110 7120 7130 7140 7150 7160 7170 7180 7190 7200

....|....|....|....|....|....|....|....|....|....|....|....|....|....|....|....|....|....|....|....|

GM_scaffold270 GCTGCTGTTATTTTAACATAAATAGCGAGTTCAGAGGATTTGGGCATGTCTGGTTTTTAGAAACAAAAAAAAATGTTATGGTAACTAGCAAATATGGTTA

ZF_chr4 ..A....C.---.............T...........G.....A.....G...G..............G...G............C..AC.GC.CA....

7210 7220 7230 7240 7250 7260 7270 7280 7290 7300

....|....|....|....|....|....|....|....|....|....|....|....|....|....|....|....|....|....|....|....|

GM_scaffold270 AGGCAGTTGAATTCTTCTTTTAAATGTTTTGAGATACATGCCAAATAATGTAGAAACAGCAACAGATTTTTGTATAGTATGCTACATGAAGCCAAGTACA

ZF_chr4 ........A.....................C..G.G.........C.............G..........-.......G....G....G........C..

7310 7320 7330 7340 7350 7360 7370 7380 7390 7400

....|....|....|....|....|....|....|....|....|....|....|....|....|....|....|....|....|....|....|....|

GM_scaffold270 GCACAAACAATTTGCTTCAGATTACAGTGTATGGGAAACACAAATCA-TAAAAAGTTCCAAGATTTGCTGCTTGTCCTTACAAATA---TGATGGTTTGT

ZF_chr4 .........GC....A.......GT..CT.G..............G.G.G...GAGC...GAT.............A.C..T....CGA.....A.C...

7410 7420 7430 7440 7450 7460 7470 7480 7490 7500

....|....|....|....|....|....|....|....|....|....|....|....|....|....|....|....|....|....|....|....|

GM_scaffold270 GGTATGGGATGTTATACTGAATTTCCTTTTAATAATGGGTGCACAGAAAAGGAGAGGATAATTAACTTGAGCTAACTTCCCCATCCTCAGAAAAGGGACA

ZF_chr4 ..G....--........CAG.........G...GG....CA...GA.....C..................C.A...C..T..T...........A.....

7510 7520 7530 7540 7550 7560 7570 7580 7590 7600

....|....|....|....|....|....|....|....|....|....|....|....|....|....|....|....|....|....|....|....|

GM_scaffold270 CACACAGAGGCACAAAAAAAATTTAAAAAAAGAAGATATACAAAAAGGAGAGACACACACACACCAAACACCTCCCTTTCAGTTTCTCAGATGATTACCC

ZF_chr4 A.....C.CA....G.G...------------------C.A......C.....TCTG....---------------------------------------

7610 7620 7630 7640 7650 7660 7670 7680 7690 7700

....|....|....|....|....|....|....|....|....|....|....|....|....|....|....|....|....|....|....|....|

GM_scaffold270 TTGGAATATGAATAAATAGACATAATTAAGGAATAAATAGAACTGTCTATTCACCCTACTGATATTTAGGTATTGTACTGAACCACAACCGTCAGCCCCA

ZF_chr4 ----------------------------------------------------------------------------------------------------

7710 7720 7730 7740 7750 7760 7770 7780 7790 7800

....|....|....|....|....|....|....|....|....|....|....|....|....|....|....|....|....|....|....|....|

GM_scaffold270 GGTGCCCACTTGGGAAACCCAGTACCCCTACTCACTTACTATTTTAATAATTTTTAAGAAACTGGGGGTCTTGTGGAGCATAGCAACTTTGAAAAAAAAT

ZF_chr4 -------------------------------------------------------......------------------------------....GG.GG

7810 7820 7830 7840 7850 7860 7870 7880 7890 7900

....|....|....|....|....|....|....|....|....|....|....|....|....|....|....|....|....|....|....|....|

GM_scaffold270 GATAAATGTAGTTTTTCTATATCTGCCTTCAATCTCTTTAACCCATTTCTTTCCATCCGAAATCATCCTTCCCAGTAGCATCAGTATGCAGCTGAGAACA

ZF_chr4 A.C.C...C.-------------------------------------------..CG..C..--------------.....TCAGG..TT........--

7910 7920 7930 7940 7950 7960 7970 7980 7990 8000

....|....|....|....|....|....|....|....|....|....|....|....|....|....|....|....|....|....|....|....|

GM_scaffold270 AGGTCGAACAGGAATAATATAGAGTTCCTACACTGAAGTTTTCTTGGCTGAATTCCTGACTACTCTTGTCTTTATGATAAAATAGATGAGTCCAAGGAAG

ZF_chr4 ---------------------------------------------------------------------------------------------------A

8010 8020 8030 8040 8050 8060 8070 8080 8090 8100

....|....|....|....|....|....|....|....|....|....|....|....|....|....|....|....|....|....|....|....|

GM_scaffold270 TCAGGCACATGTGGTGAAAGGTGGCATTGGCCTCTCTTGCAAAGTGGTCTGAATTTCTTTGGGTTTTTGTCTCAACACTTAAGAGTTGGATATCTAATAA

ZF_chr4 ...T...TG.A.....G................G.T...AG.....C.T........C....T...........G.G..G.G..A.A.A....GG...C.

8110 8120 8130 8140 8150 8160 8170 8180 8190 8200

....|....|....|....|....|....|....|....|....|....|....|....|....|....|....|....|....|....|....|....|

GM_scaffold270 ACATTGAATAGTTCTCTAGTCTAACACTTGACACACTGGGCACTGGAAGGTTGTCTGTTTTCTGCTAAGCAGGTGGAACTTCCCATCTGTTTTACTTGAT

ZF_chr4 .G..C....-.C..........G..T.....TGA---..AA.......................G.........AC.....GT.C.....GAC..C..G.

8210 8220 8230 8240 8250 8260 8270 8280 8290 8300

....|....|....|....|....|....|....|....|....|....|....|....|....|....|....|....|....|....|....|....|

GM_scaffold270 GTGGTCAGGTTGGATGAAATTGGATCCCACAGCTCAAAACTCATTACAAGAAAATGGATGTGCTTGTACAATGTAATTGCTTAAATTTGGTTTCCATGGG

ZF_chr4 .......CT....T.....C-A..C.T.........T.........TT.A.....T.G.C.....A.G..G.AGGG.G.......C........TG...A

8310 8320 8330 8340 8350 8360 8370 8380 8390 8400

....|....|....|....|....|....|....|....|....|....|....|....|....|....|....|....|....|....|....|....|

GM_scaffold270 AAATCTATTTGGAAATATCCAC-CAATGTTTTTCTGTTTTTTTTTTTCTCTAAAGGATTAGATTACCTAACATCTTTGAAGAAATGAGAAGTAAAATGAC

ZF_chr4 .......G..........G.T.ATCT.A..C...AAAGGA..AGA..ACA.....TCC.------TA.GCTCC........................T.T

8410 8420 8430 8440 8450 8460 8470 8480 8490 8500

....|....|....|....|....|....|....|....|....|....|....|....|....|....|....|....|....|....|....|....|

GM_scaffold270 TGAGAAGCCTAGTTCTTTGAATGGAAAAAATGCTGTGTGCTTTCTTGGTATGCTGACTATATGGTTTGCAGTGGGAATAAAATCTTGAGTGACTTTTTTT

ZF_chr4 .A..C...G.G....C.....A....T.T-................AC..........T-----.......G..C..--..........A.G........

8510 8520 8530 8540 8550 8560 8570 8580 8590 8600

....|....|....|....|....|....|....|....|....|....|....|....|....|....|....|....|....|....|....|....|

GM_scaffold270 T------AGAACTGTGATGCCTTGCTGAGTAAGCATCCAGGACATGCTCCAGCTGAGAGGGATTTTCCTTTTTATAGGTATCTGATTTTTAGCAGGAAGT

ZF_chr4 .TTATTT......AG........A........A.......................A..............A....................G......C

8610 8620 8630 8640 8650 8660 8670 8680 8690 8700

....|....|....|....|....|....|....|....|....|....|....|....|....|....|....|....|....|....|....|....|

GM_scaffold270 TTACCAGACTAGGGAAAAAACTGATATATAATTCATTATGGTTCTAATTAAAGAACTGACCCCATGTGTTAACCCAAGAGAGAAAGGATCCTTTTGTTCT

ZF_chr4 G.............GG.....C..C.......C......A...G..................TG.................A................T.

8710 8720 8730 8740 8750 8760 8770 8780 8790 8800

....|....|....|....|....|....|....|....|....|....|....|....|....|....|....|....|....|....|....|....|

GM_scaffold270 TCCAACCAGAATTAGCTGACAGAAGCATATAAGCTTTGCCACATAAGCAGTCCCGTGCCATTAGTAGTTTATCATGCTTTT-TTTTCCTTGCTGGTCTTT

ZF_chr4 ....G.......G..............C.A.T..................C..T.....................ATC..AG...............G..

8810 8820 8830 8840 8850 8860 8870 8880 8890 8900

....|....|....|....|....|....|....|....|....|....|....|....|....|....|....|....|....|....|....|....|

GM_scaffold270 GGCTATGCAAGAGAAGGGCAACAATCTACAAAGGAGGAATTCCAGTTGCTCCTTGGAAACTGTTTTGAGG---GTTC----------------ATTACTG

ZF_chr4 .C.--..................-------.......G...............................TTCT....CAGTTGCTTCTTGGAA.A.GT..

8910 8920 8930 8940 8950 8960 8970 8980

....|....|....|....|....|....|....|....|....|....|....|....|....|....|....|....|....|...

GM_scaffold270 TTCCTTGCATACTTTTAAGCAGCCTCTTTCCTTACGACTTAAGTCCTTCTAAAACTAGCTATCTGGGGAAAACAAGAATTCTAGAAGG

ZF_chr4 ..A....A..G...C.G....A.T...........A...G.............C...C..G.G.........A..AC..CA.GTGG..

**(ii) ePaV *Egretta garzetta* and ePaV *Pelecanus crispus* 2**

10 20 30 40 50 60 70 80 90 100

....|....|....|....|....|....|....|....|....|....|....|....|....|....|....|....|....|....|....|....|

LE_scaffold81 CGTCTCAAGTATTAGTGCTAAAAGTCTGTAATAAACTAGTACAGCAGT---GGACTTCCTTCTGTGTTTCCCTCTTGCCAGTCATATTTAGGGTTGCCAA

DP_scaffold1346 T.......AC.....CA..........C.............TGAT.AACTA.T..........C.....T.......T..A.......C...........

110 120 130 140 150 160 170 180 190 200

....|....|....|....|....|....|....|....|....|....|....|....|....|....|....|....|....|....|....|....|

LE_scaffold81 GATGAGTCTTTAAAGCAAGTCCTGAATCTTAACAATGTCTCCATCTGCCATTTTGTTCCATAGACAGTATACAGTGTTTTGTGGCTATATATTGTGTGTA

DP_scaffold1346 ...A......................G.......G.C...T......A......................G.T..........CT.G...G...C.CT..

210 220 230 240 250 260 270 280 290 300

....|....|....|....|....|....|....|....|....|....|....|....|....|....|....|....|....|....|....|....|

LE_scaffold81 TCCTTGACTTCATGACTCTTTGCATACCATTATAAACTTTATTACTAAAGTAGTTTTGACCATCTTCCGTAAGAATACTCTACTAGCTCTCCAGTATGCT

DP_scaffold1346 A................AA.C.....T...........G..........A..........TG......A.........-----..T..............

310 320 330 340 350 360 370 380 390 400

....|....|....|....|....|....|....|....|....|....|....|....|....|....|....|....|....|....|....|....|

LE_scaffold81 CACCCATAACATAAAAAATGACATATTAAAGATAACTGATCTGGATACTCATTTTAAAACCCTCCTGATTTGCTTTCTCCTGCTTCTTTACTTTTTG---

DP_scaffold1346 T.........G...G.C........---G.......C.....A.......T......G.GTT................G.........C.......AAAA

410 420 430 440 450 460 470 480 490 500

....|....|....|....|....|....|....|....|....|....|....|....|....|....|....|....|....|....|....|....|

LE_scaffold81 ---TT-------TTGTTTTAGAGGTTGGCAATATTTAGATTCTATAAAATTCGTTACTAGTAAAGGATTGCCTATGCCTTTATGAATCTGCTTTTG----

DP_scaffold1346 TAA..ATTTCAA...........AC.......G......C...G........C...A...........G.......T.......G.........CTTTTG

510 520 530 540 550 560 570 580 590 600

....|....|....|....|....|....|....|....|....|....|....|....|....|....|....|....|....|....|....|....|

LE_scaffold81 -CTTTGTCTTTAGCGTGGTATCTTCTGCCTGG-----ATTTCTATTCCTAGCTTGGTTTTTTTTGAGGGGTGGGGTGGGGTGAGGTGTGTT----TCAGT

DP_scaffold1346 C......G.....TT.................TCTGC...A..........GGA.T.....A..TCA.TCCA.ACCT.TC...AA......GGGC.AGT.

610 620 630 640 650 660 670 680 690 700

....|....|....|....|....|....|....|....|....|....|....|....|....|....|....|....|....|....|....|....|

LE_scaffold81 CCAGCCTTGTCTGAAATTCATTTTTTT---TACACTCATAGGTGGTTG--------------------TTTGGGGTTTTTTGGGGTGGGGTGTTGGGA--

DP_scaffold1346 TTG.TT....T.TGCT..TT......CCCA.G...A.GCG.T......GTTGTTTTGTTTTTGTGGGT.............TT..G....G.CA....GA

710 720 730 740 750 760 770 780 790 800

....|....|....|....|....|....|....|....|....|....|....|....|....|....|....|....|....|....|....|....|

LE_scaffold81 --------------TGTTC--------TTGGTCTTGTTTTTGAGAAATTAGATTCCCCATACAGACTGGTAAAAGAGTTTTCAACCGGTTTTCCATTAAC

DP_scaffold1346 GGGAGAAGGGAGGG....TGAGGTATT..T......G...A...G......G....T..............G.G...C...TC...A.............

810 820 830 840 850 860 870 880 890 900

....|....|....|....|....|....|....|....|....|....|....|....|....|....|....|....|....|....|....|....|

LE_scaffold81 AAGTCTTTTCCTGGAGAAGGCCAGTACTCTCTTGAGTATTGCTAAAAGTAGTCACGGCATCATAACTGTGATTGACTTTGT----TAATGTGCAAGTTGT

DP_scaffold1346 ......G.......G..G..T...........CAG.........T..A......................G..........CTGT...............

910 920 930 940 950 960 970 980 990 1000

....|....|....|....|....|....|....|....|....|....|....|....|....|....|....|....|....|....|....|....|

LE_scaffold81 GAGAGGTAGATCTTCACTCTCACTTCTGCTCATCTGAGGTAAGTTCATTAACGTTTCCAATGGTTCCCAGTATCTATTCTACTGTGTCTTATGTTCTCTG

DP_scaffold1346 ..A...........T...G...G....-.....TCA..A...A..A..C...A...TT..CA.........G.........T..................

1010 1020 1030 1040 1050 1060 1070 1080 1090 1100

....|....|....|....|....|....|....|....|....|....|....|....|....|....|....|....|....|....|....|....|

LE_scaffold81 CCGCTTTCTTGGGATGTGAACTAACAGTAAGTCTTTTTCATGATATGAATTGTGCAAACATGTGACCATTCCCCCCTTCCTATGCTTCCCGACTCTTGGC

DP_scaffold1346 .T..........AT....................C..G.....A.......C......T..............TT..............T..........

1110 1120 1130 1140 1150 1160 1170 1180 1190 1200

....|....|....|....|....|....|....|....|....|....|....|....|....|....|....|....|....|....|....|....|

LE_scaffold81 CATATTCTGCTCTATCACATGTATTGTCAATCTACAAATATTTTGAAGGGTTTTTATGTTTGTGGTGACTGGAACATAACTCCACGTCTGGAGACAATCC

DP_scaffold1346 ...G.............A...............T....C........AT.....C.CA....C...............G.....T.........G.....

1210 1220 1230 1240 1250 1260 1270 1280 1290 1300

....|....|....|....|....|....|....|....|....|....|....|....|....|....|....|....|....|....|....|....|

LE_scaffold81 CACATGTGGTGGCATTGATATTATAGATGATGACTTTGGTAACCTGAGTGCCTCTGTTTTGTGGGGTTTTTTTTGTTTGTTTTTTTGTTTGTTTTTAAAT

DP_scaffold1346 ...G....ACATT........G........CC.....A..................C...-------------------.C.....T...T.....T...

1310 1320 1330 1340 1350 1360 1370 1380 1390 1400

....|....|....|....|....|....|....|....|....|....|....|....|....|....|....|....|....|....|....|....|

LE_scaffold81 CTTGTTTTCCTATTCCTTTCCATGAGTTTGCCATTTTTTCAGCAGCCAAGCCATTATCATTCTGGCTGTTTGTTGTCTTCTTGAGAAGAAAGCTAGCAAC

DP_scaffold1346 G...C.................CA.A............A-G.........T.T...........A...................................

1410 1420 1430 1440 1450 1460 1470 1480 1490 1500

....|....|....|....|....|....|....|....|....|....|....|....|....|....|....|....|....|....|....|....|

LE_scaffold81 AAGACGTAGGAAAACAAGCACTGGCCAAATATCTTTTCTTCCAGGGCCTAGCAAGGATGGTGAGGCTACCTTTTGTGTCTGCCTGTGCTTTCAGTAATCA

DP_scaffold1346 ....A....A....T..C....A...........G...CG..............A.....C.......TT..........-....AC.......C.....

1510 1520 1530 1540 1550 1560 1570 1580 1590 1600

....|....|....|....|....|....|....|....|....|....|....|....|....|....|....|....|....|....|....|....|

LE_scaffold81 GGACAGCTAGGATTGACATGAGCCTTCACAGCCTTGTTGGTGACTGAAGCCAGTTTCAGCTTACAGCGTAACTTGAACAGATATGTGTATTCTACAAATC

DP_scaffold1346 .....ATA...G..A........................A.....A....................T....G...T........C...............

1610 1620 1630 1640 1650 1660 1670 1680 1690 1700

....|....|....|....|....|....|....|....|....|....|....|....|....|....|....|....|....|....|....|....|

LE_scaffold81 ACACTTTTCATGCCTAACAAAATAATGCTTATAGTAATAATTACAGTTATTTACATTCATAGTAGTACAATACTTTGGTGTTAACATCTTGGCTTAAACA

DP_scaffold1346 ...G.........T....T.......--.A...T..............................T..TG..G.....A.A......G.............

1710 1720 1730 1740 1750 1760 1770 1780 1790 1800

....|....|....|....|....|....|....|....|....|....|....|....|....|....|....|....|....|....|....|....|

LE_scaffold81 TAGTTGCCAAACACTGTTCCTCAACATGTGTTGCATGTGTGCTTTCTTTTAAAGATTTTAATAATTTGAAATAATGAATTTTTTTATTAGACTTGAAATG

DP_scaffold1346 ...........TG................T...........T..C..........................C..........C....C............

1810 1820 1830 1840 1850 1860 1870 1880 1890 1900

....|....|....|....|....|....|....|....|....|....|....|....|....|....|....|....|....|....|....|....|

LE_scaffold81 CATCATAATGATGCACATATCATCCATGGTAATGAGTCATTGTCCAATAAAACAAGTATCTTGATAGGGGACCTCTTTTAAGCATTCTTGGCTGCTGCAG

DP_scaffold1346 ....G.C.A...........T---...AA..........C.----..C....AC..........A............................A......

1910 1920 1930 1940 1950 1960 1970 1980 1990 2000

....|....|....|....|....|....|....|....|....|....|....|....|....|....|....|....|....|....|....|....|

LE_scaffold81 CAGTTTCCAGGATAGGATTTAGAGGTGGCAGAGAGAT--AGTTGCTTCCAATGTATTCCAGAAGGGACTAGCGCAACAAAATATGGATTTGTTTTCTGCC

DP_scaffold1346 ....C...........................T....TA...............G................A....T......CA.............T.

2010 2020 2030 2040 2050 2060 2070 2080 2090 2100

....|....|....|....|....|....|....|....|....|....|....|....|....|....|....|....|....|....|....|....|

LE_scaffold81 AACACTTTAAAATCTAAATCAATATATTTTCCATCCACTCATGCAGAGTAGAAGTTGTGAACAGCAAGGCAGTAGAGTTTGAAGAGCTGTTAGTAGTAGG

DP_scaffold1346 ...G................................-.......T..A....................AA..............G...........A...

2110 2120 2130 2140 2150 2160 2170 2180 2190 2200

....|....|....|....|....|....|....|....|....|....|....|....|....|....|....|....|....|....|....|....|

LE_scaffold81 TTAAAGAGCTTTTGTCTGAGCACAAATGGACCTAATAAACACACTCTAGGGAGGAGAAATGACTGTGCTTGTGTCACAAGCACAAAATTTGGCCCTCATC

DP_scaffold1346 C......................G...T.............T.G..C......C..........T..........--...........C.....AG....

2210 2220 2230 2240 2250 2260 2270 2280 2290 2300

....|....|....|....|....|....|....|....|....|....|....|....|....|....|....|....|....|....|....|....|

LE_scaffold81 CCTGGTTCAGTCACAGACACAATCTGCTACACTGTGAAAGCCACTTAGTTTCTTCGAGAGGCACCTAGATCCATTTTTATGTGAATAAAAGGCGGTGAAT

DP_scaffold1346 A......T.A...T..................C.A.....T.............T........................G............T....G..

2310 2320 2330 2340 2350 2360 2370 2380 2390 2400

....|....|....|....|....|....|....|....|....|....|....|....|....|....|....|....|....|....|....|....|

LE_scaffold81 TTTCCAAAGTGATAATTGCGGTCTCCATTGATTTTATGAGTGCCTAGATATAGATTATTGTGCTTAATCATCCATGTTTAAAGCTGCTAATCTTAATTTG

DP_scaffold1346 ...........G...C...T..T..........G...T......C.....C...A........C.........................T.......CA.

2410 2420 2430 2440 2450 2460 2470 2480 2490 2500

....|....|....|....|....|....|....|....|....|....|....|....|....|....|....|....|....|....|....|....|

LE_scaffold81 TGATCACATATGTCACTCTTATCACCTGTAAAATCACAATTATATGTTCTTTCTCAAAAACTTTCTTTCATTTAACTTCAGAGTATAACTTGGATGG-AC

DP_scaffold1346 ...............T....C................T.C.G.G.....................----...............G.C......G...G..

2510 2520 2530 2540 2550 2560 2570 2580 2590 2600

....|....|....|....|....|....|....|....|....|....|....|....|....|....|....|....|....|....|....|....|

LE_scaffold81 AAAGTCTGTGTCTTGGTGTGCATAGGACAC--AACAGAATGGAGCCCTAATCTTTGTTGGCATCTCTAAACAGCTGTTACCTT-CATATAATGGTAAAAA

DP_scaffold1346 ......CA............T.....G...CT.....G...A........C.............................T..AT.......AA......

2610 2620 2630 2640 2650 2660 2670 2680 2690 2700

....|....|....|....|....|....|....|....|....|....|....|....|....|....|....|....|....|....|....|....|

LE_scaffold81 TGTGTTGTTT---TTTTTTTTTAACCTAAGCATTTTCAGAATGCAATATCGCAGAATTCGTAAGTATCACTACAGTTTGCTTTCAGACACCATTGTGATG

DP_scaffold1346 ..........GGT......C.......G..................C...A.....G..A.......................TG...GTT.........

2710 2720 2730 2740 2750 2760 2770 2780 2790 2800

....|....|....|....|....|....|....|....|....|....|....|....|....|....|....|....|....|....|....|....|

LE_scaffold81 TCATAAAATTATGTACTTTGTATAGCC-CTATCATAAGATAATTAGCTTGGACTGGGTAACATGGAATTTATTTTAAGTCAAGCTCAGCTTTGCGTGGTT

DP_scaffold1346 ...........................A...........G.G.................T.......C.....................C...TC...G.

2810 2820 2830 2840 2850 2860 2870 2880 2890 2900

....|....|....|....|....|....|....|....|....|....|....|....|....|....|....|....|....|....|....|....|

LE_scaffold81 GTGCCAAAAACCTTTCAAACTTACTATCCTTCCATGCATGAGTGGCAAAACGCTGCTCTTCCACTCTGTT----ACTACCTGCCTCAATGGGAAGAGGTA

DP_scaffold1346 ..........A....A...A..................CATC........A......T....G...CA..TAGT..A...............G.......

2910 2920 2930 2940 2950 2960 2970 2980 2990 3000

....|....|....|....|....|....|....|....|....|....|....|....|....|....|....|....|....|....|....|....|

LE_scaffold81 GAAAACTAAAAAATGGTGGTGGGATATCCATGTGCTGCAATAAAATTACTGCTGGTTTATATCAGCAAAATATTTGTTGAGAATGAGA-TGGAAAGCTTT

DP_scaffold1346 .......T.......A...............C...CAGG......A.....T...............G....................G..A........

3010 3020 3030 3040 3050 3060 3070 3080 3090 3100

....|....|....|....|....|....|....|....|....|....|....|....|....|....|....|....|....|....|....|....|

LE_scaffold81 CAGGATGGGGTAGCATTTCTTACACATGTTGTTAAACAGAGTGTGAGTTTTGTTTATCAAGGCTATATTCTCTACAGCACTTAATTGTGGAAAGACAAAT

DP_scaffold1346 AGA......C....G.............---........G.....G..............A......................T......G....G....

3110 3120 3130 3140 3150 3160 3170 3180 3190 3200

....|....|....|....|....|....|....|....|....|....|....|....|....|....|....|....|....|....|....|....|

LE_scaffold81 CTATCTGGAGAAAAAAAGTCTACTGAAAAAG-CATCTGACTTCTTTGGATACGTAAGTGTTGTGGAGCTGTCAGCAAGCTTGTTACCACTCCTGGCATGA

DP_scaffold1346 G...T.A.........C-...G........AA...GG....G.........A.....A.C..........................TG....G.......

3210 3220 3230 3240 3250 3260 3270 3280 3290 3300

....|....|....|....|....|....|....|....|....|....|....|....|....|....|....|....|....|....|....|....|

LE_scaffold81 CTGCTTGCTGTGATTCTTGACTGAAAAGC--ATTCTCTCGAATTTTAGGTGGCATCTGCAGAAAATGCCCAAAGGCATAATGGACAAGATGCCCAGAACT

DP_scaffold1346 .............................GC...T...G..............C............A.................TG..............

3310 3320 3330 3340 3350 3360 3370 3380 3390 3400

....|....|....|....|....|....|....|....|....|....|....|....|....|....|....|....|....|....|....|....|

LE_scaffold81 AATTATCTGCAACATGGAGGGAAGTTCATAGTGCTTCTATTT-GAATGAGTAATTTCAATTACTTGACGGCATTTTTAAAGCACTGGCATATTTGATGGG

DP_scaffold1346 .............T............T...............T............C...........G.A......A.................A....A

3410 3420 3430 3440 3450 3460 3470 3480 3490 3500

....|....|....|....|....|....|....|....|....|....|....|....|....|....|....|....|....|....|....|....|

LE_scaffold81 GTGGGGGGAAACCCAAACAGAGCTTGTCAATGGGATCTGTTGATGCTGGCATTTGGTGGTGTAGATATTAACAGTGGGATAATACTTGCAATTCATTTAG

DP_scaffold1346 .G.T.............T.C..............................G...A................G...........................A

3510 3520 3530 3540 3550 3560 3570 3580 3590 3600

....|....|....|....|....|....|....|....|....|....|....|....|....|....|....|....|....|....|....|....|

LE_scaffold81 CTTGAAAAATGAAAACATCCAGTGGAAGGCCAAGGTTTACAATAAATGAGTCTCTAAAGATAGGCTCCTCACTCAATGTTTATTTACTTAATAAAGAAGA

DP_scaffold1346 ...........G..GTG......................................G.........................................CA.

3610 3620 3630 3640 3650 3660 3670 3680 3690 3700

....|....|....|....|....|....|....|....|....|....|....|....|....|....|....|....|....|....|....|....|

LE_scaffold81 TTGCTTTTTAA--GTGTCAAGTACTCATCAACTGCTATTAATACCAGTGGAATTAGTAGCAAAGGAAATAACAAAGAATGGTGCCATAATTATTAAGTTG

DP_scaffold1346 ...........AA............T......A..............CA....G..................T................C..........

3710 3720 3730 3740 3750 3760 3770 3780 3790 3800

....|....|....|....|....|....|....|....|....|....|....|....|....|....|....|....|....|....|....|....|

LE_scaffold81 GTTTTGTGCTAGGTAATCTGTGCATGTGGTCAGAAGCAAGCCAAGTGTTCAGACTGTGGATTCTGGCCCAAATAAGCCTACTTTCTGATGTAAAATAATA

DP_scaffold1346 ......C...................G.........G.........A.....TT..CA.......................C..................

3810 3820 3830 3840 3850 3860 3870 3880 3890 3900

....|....|....|....|....|....|....|....|....|....|....|....|....|....|....|....|....|....|....|....|

LE_scaffold81 GAAAAAACCAGAAAGGTGTGTTATGGACAGGAAGGTAA-CAAGGGTAGTAGAAGAAAGATCATATGGTCAAGTCAGGAAGGTATATGTTTCTCTTGGGGA

DP_scaffold1346 T....CC......TT........CA.............A...................G........C...CG....G....G.............T...

3910 3920 3930 3940 3950 3960 3970 3980 3990 4000

....|....|....|....|....|....|....|....|....|....|....|....|....|....|....|....|....|....|....|....|

LE_scaffold81 TTTGATGTCCTATTTTAACTGCCCTTGTTCCTACAGGCAGCATGGAAAGCAAATGATGACAGAGAAGGTAGCTTGGCAGATGGACTTGGACAGCGTGCCC

DP_scaffold1346 .....CA.TG.....C.....T...C...........................CAT.......C.....C..........................A...

4010 4020 4030 4040 4050 4060 4070 4080 4090 4100

....|....|....|....|....|....|....|....|....|....|....|....|....|....|....|....|....|....|....|....|

LE_scaffold81 TGTGCAGAAGGGGCAGCATGGAAGGTGACACTGTGCC-CCTGTATGCCTTTGAAGAAGGAGGCAGGAGATACGGACAGATTGGAACGCACATGGCCAGTA

DP_scaffold1346 .................................A.G.A..................................A..T....A...................

4110 4120 4130 4140 4150 4160 4170 4180 4190 4200

....|....|....|....|....|....|....|....|....|....|....|....|....|....|....|....|....|....|....|....|

LE_scaffold81 TGATGTGAGCTCAAAATGTAGGCTTGCTGTGAAGGGGACTGAATTAGTTTCAGGTGACATCATACATAATTACTGATTATATTTAGAGTATGTGGTAATA

DP_scaffold1346 ....................................................TA............A....................A............

4210 4220 4230 4240 4250 4260 4270 4280 4290 4300

....|....|....|....|....|....|....|....|....|....|....|....|....|....|....|....|....|....|....|....|

LE_scaffold81 TGGTCTGAGACCTTCCATATGTTAATTCTGCAAGCTTCAATATATATCTACAGGAAGCTTGCAAAATCAAAGCAAGCCTAGGTAGGGAAAATATTTTCTC

DP_scaffold1346 ........TT.......................A...........................TG...................C....G............

4310 4320 4330 4340 4350 4360 4370 4380 4390 4400

....|....|....|....|....|....|....|....|....|....|....|....|....|....|....|....|....|....|....|....|

LE_scaffold81 TATATGGACTTTAAGCACAGTTCTGCAAAAAGTTAATATAGCACAACCAAAGTTGG---------TTTTTTTTATCATCCAAGTTGGTTGTCTTTAATGT

DP_scaffold1346 ......A.........................G...C...C..............TGCTTTTTTT..........T..........T.............

4410 4420 4430 4440 4450 4460 4470 4480 4490 4500

....|....|....|....|....|....|....|....|....|....|....|....|....|....|....|....|....|....|....|....|

LE_scaffold81 GCGTAAGAATTGCATACTTACATATTCAGGCTTAGTTCATATTTTGGGACATGAAAGTATTTGGTCCATATTAATCAAATGTA----GTGGTTTTGTTGA

DP_scaffold1346 .T.C.........................C.........................G...............AT....T..A..TATA.............

4510 4520 4530 4540 4550 4560 4570 4580 4590 4600

....|....|....|....|....|....|....|....|....|....|....|....|....|....|....|....|....|....|....|....|

LE_scaffold81 AAAGTAGAGTGTAAACATTTCTAATATATTACATTATAACATGAGTCTTAAGGCTCAAATAATTCTGTGAAATCTGTTAGCCAAAGTTGAAAATATCGTC

DP_scaffold1346 .........A.......C.....................................T.....CC......G..............T........C..T.C.

4610 4620 4630 4640 4650 4660 4670 4680 4690 4700

....|....|....|....|....|....|....|....|....|....|....|....|....|....|....|....|....|....|....|....|

LE_scaffold81 TCTGAATTATCTTAATAACTTATTATATATGGATGAGCTATTGAAAAACCCA-CAAACAACCCCCCTCCAAACAACAGCAAAAAACCAAACCAAACCCC-

DP_scaffold1346 ......A...............C...................A....GAA..G......C....T............A...C...A.........A.T.A

4710 4720 4730 4740 4750 4760 4770 4780 4790 4800

....|....|....|....|....|....|....|....|....|....|....|....|....|....|....|....|....|....|....|....|

LE_scaffold81 ------TCTTATAGCCTTACTTTTTATGTG--AGATCTCTTGCTGTCCTTCTGTCCATTCTTATGGTGATCTCAGAAGCATAATACCAAATACTTGAAAT

DP_scaffold1346 AAAAGC....G........T....C.....TG.........A................C....CA..T................................

4810 4820 4830 4840 4850 4860 4870 4880 4890 4900

....|....|....|....|....|....|....|....|....|....|....|....|....|....|....|....|....|....|....|....|

LE_scaffold81 TATGAATGTTCATCCTGATAGCAAATTTGTGTAGGTAAGTAACCTGGGCCCTTAAACTTAGGTACAGGGAGGATTAAATTAGTTAAAAGCATGTTATTAA

DP_scaffold1346 ........................C.C..............GT....A..........C.....................G........AGCA.......

4910 4920 4930 4940 4950 4960 4970 4980 4990 5000

....|....|....|....|....|....|....|....|....|....|....|....|....|....|....|....|....|....|....|....|

LE_scaffold81 CCTAGGCTGAGCTGAGACTGAGGCAGACTACTTACAGGACATTCCTTCTACCTTCAAAATTCCTAATCTATTTTACTACCAGTAATGCATGGATTTTGGC

DP_scaffold1346 T.............GT...............C.T...A................T....A.A........C.............................

5010 5020 5030 5040 5050 5060 5070 5080 5090 5100

....|....|....|....|....|....|....|....|....|....|....|....|....|....|....|....|....|....|....|....|

LE_scaffold81 TGAGTATCTGTGTGAGAAACTAACCTACAAATCCTTTTTTTTTTTTTAAAAAA--AAAAGAAAGTCATTGTCTTTTATAGGAAGTCTAGTGAAAAAATGG

DP_scaffold1346 .A..........G..C..........G....................TTTTTTTT..T...................C...................ATT

5110 5120 5130 5140 5150 5160 5170 5180 5190 5200

....|....|....|....|....|....|....|....|....|....|....|....|....|....|....|....|....|....|....|....|

LE_scaffold81 CTTTTTCTGAAAAGACTGAGAAGTTTTGCCTGTGTACCAAATAATTTGATTCGAAAACACTGGTGAAAGAAACTGTTGGAAATTGTAGATCAGTTGTCTT

DP_scaffold1346 AC.....A...........A........T.....C................GA.........C........G............................

5210 5220 5230 5240 5250 5260 5270 5280 5290 5300

....|....|....|....|....|....|....|....|....|....|....|....|....|....|....|....|....|....|....|....|

LE_scaffold81 ATGTTCCCAATCTACAAGGTAACACTTCG----GGTAGTTCTCATAATAAAAGTTACAAAATTGTTGGATAATTAGCTATAGTCTGG------AGTCCAG

DP_scaffold1346 ..A...T..G..........G.A....T.TCTG.A...A................G...............G..T.A..C.A....TTCAAGC..C....

5310 5320 5330 5340 5350 5360 5370 5380 5390 5400

....|....|....|....|....|....|....|....|....|....|....|....|....|....|....|....|....|....|....|....|

LE_scaffold81 TCCAGACAGAGTGTACAACATTAGGTTCCTGAACTACAACATCTTATCAAAAGAGACTTCAATTTTGAGATGAAATAGATATTTTCTGTAGCGAGCCTAC

DP_scaffold1346 .T.................G...A......................C......G.....T..A..................AC....A...AA.......

5410 5420 5430 5440 5450 5460 5470 5480 5490 5500

....|....|....|....|....|....|....|....|....|....|....|....|....|....|....|....|....|....|....|....|

LE_scaffold81 ACTTCTCTCTTTCCTACTCCTCCCCAACGTTTATT----AGCCCAAATTTCAATTGAAAAAGTGTTTTCACAGAAAATCTTTGACTGACCTACTTACAAC

DP_scaffold1346 ...................-.T......A......GGAA......G....T.G.....C..A......A.T.....G..C....................

5510 5520 5530 5540 5550 5560 5570 5580 5590 5600

....|....|....|....|....|....|....|....|....|....|....|....|....|....|....|....|....|....|....|....|

LE_scaffold81 CTTCTGTTTGAGAAAAATGGTTTACTCAAATCTGTTTAAAACCAAATCCTTGTTATGCAGGGAAGCTGTGATTTAGTCCAGGCCACGTGGCAACTGGGTG

DP_scaffold1346 ...................A....T......A.............T..T..T........................CT....T..T.........A...A

5610 5620 5630 5640 5650 5660 5670 5680 5690 5700

....|....|....|....|....|....|....|....|....|....|....|....|....|....|....|....|....|....|....|....|

LE_scaffold81 ACAGGGCTGAGAGGTGTCTTTGAGAAGAATTTCCCTTCTTCCTTGTCTTTACATTATGCATCTCTTCTCCCCTAGAAAATTTCTACTGCTATATGCAGGA

DP_scaffold1346 T....A.......A........G...A......TG...............TG...C.......G........C......................TG...

5710 5720 5730 5740 5750 5760 5770 5780 5790 5800

....|....|....|....|....|....|....|....|....|....|....|....|....|....|....|....|....|....|....|....|

LE_scaffold81 CTGAACTACCACTCTGGCTGTGCGCAG---GAGTACAAATGCTTGTCAGAATATTTTATTGAAGTTGTTTTTTGTAGTAAGTTCCAGTGTGGCAGTGTCC

DP_scaffold1346 .........T..........CA.A...AAG..............C.............C.............-.......A...TGA.............

5810 5820 5830 5840 5850 5860 5870 5880 5890 5900

....|....|....|....|....|....|....|....|....|....|....|....|....|....|....|....|....|....|....|....|

LE_scaffold81 CACCATTAGCGTTTGAAATGTGAACCTGCACCTTCACATTTCACGCAACGCTGCTTTATTTTCATTGAAGATTTCCGAATACTTAACAGTTATTCTACTG

DP_scaffold1346 ....G....T..................................C....A..A.C.CC..............C...A....TA.....T.....T.....

5910 5920 5930 5940 5950 5960 5970 5980 5990 6000

....|....|....|....|....|....|....|....|....|....|....|....|....|....|....|....|....|....|....|....|

LE_scaffold81 CCACGGTATTATGAAAGCACCGAGGCAGCACTTGCTGTCCTTGGAAGAATTGGGACTGTTTGTTCTTCTGACCTGACTAATGAAGAAATGGAAGAAACCG

DP_scaffold1346 ...T...TA...........TAT..........A.....A.........C.....................G...........................A

6010 6020 6030 6040 6050 6060 6070 6080 6090 6100

....|....|....|....|....|....|....|....|....|....|....|....|....|....|....|....|....|....|....|....|

LE_scaffold81 ATTTCATTAATCTCATATTTTTGTGCTCTGGTAGTTTTTAAAAAGAGAACTTGTTT--------TTTCCTCCCTGTGAATAAGGTGTAATAATAAACTTT

DP_scaffold1346 .......C.......C......................G.............CG..GTTGGTGG...T..T...........A.....G......G....

6110 6120 6130 6140 6150 6160 6170 6180 6190 6200

....|....|....|....|....|....|....|....|....|....|....|....|....|....|....|....|....|....|....|....|

LE_scaffold81 TCCATAACTTTATTAAACCAGATCACGTAAATGCTCTGGAATGGGCTCTT---ATACTGGTCGGGCAGAAGGAGGTATTTGCTGACTGGCCCTCATAGTC

DP_scaffold1346 ............C.............A........T..............CCT.........AT.......................T.A..........

6210 6220 6230 6240 6250 6260 6270 6280 6290 6300

....|....|....|....|....|....|....|....|....|....|....|....|....|....|....|....|....|....|....|....|

LE_scaffold81 CTCTTTGGTGTGGTTGTAGCTGGAGTGAAGCAGTGGAAATGACTGAATGCTAACATCAGGGGGCTGAGTAACCAAATCGAGAAATTTGTACTGGCAGTAG

DP_scaffold1346 ...........T...........T.....A..................A...........................CT.........A.G..A.......

6310 6320 6330 6340 6350 6360 6370 6380 6390 6400

....|....|....|....|....|....|....|....|....|....|....|....|....|....|....|....|....|....|....|....|

LE_scaffold81 GGGAAGTGAAATGGGCATCATAGGGATATTGTAAACAATGTACAGCAACTGTCTTGACTGGAATAGATGTGAAGGATATTTGATCTTCAGTTAAGGCAGG

DP_scaffold1346 .A.......................G..A.A......G....T......A.......................................T......T...

6410 6420 6430 6440 6450 6460 6470 6480 6490 6500

....|....|....|....|....|....|....|....|....|....|....|....|....|....|....|....|....|....|....|....|

LE_scaffold81 TGGTTTCGCACCAGGTGTTACTAATAAAAT---CTGCAAAAGAAAAACGAAAAATCACTTGAGAGAATGCTGAAGTAACCTCTGTTGGGCAAGTCTGCTC

DP_scaffold1346 ......T.............G.........TTT........AG....T.........G...G.....................A................

6510 6520 6530 6540 6550 6560 6570 6580 6590 6600

....|....|....|....|....|....|....|....|....|....|....|....|....|....|....|....|....|....|....|....|

LE_scaffold81 TAGACCATTTGGGCTGACTTTAAAAGGGAATAGAGATGTCTTTCTGCTGTTAGAAAGAGCGAGCGACAACTGGGAATTGCACTATTATGTGAAAGTTCAA

DP_scaffold1346 ....T........A......C......................T..............--...T...............T........A-.........T

6610 6620 6630 6640 6650 6660 6670 6680 6690 6700

....|....|....|....|....|....|....|....|....|....|....|....|....|....|....|....|....|....|....|....|

LE_scaffold81 CTTCCCACATTAGAGAGAGAATGTTGCAGAGTCCCACAAAACCCAACAAAACTGATATTATTTTAGGTTTAATTTTGGTAAGCAGTGAAGGCCCAGAAAA

DP_scaffold1346 .....................C.....G......A......AA..............C.G........................................

6710 6720 6730 6740 6750 6760 6770 6780 6790 6800

....|....|....|....|....|....|....|....|....|....|....|....|....|....|....|....|....|....|....|....|

LE_scaffold81 AAA-TAGGCATAGAAAATAACTTCGGACAATATGATGGAGCATGAATGCAGGAATTGTTGTAAACCCCTTTTACCTTCATTTTATTACAGCATTGTTGTA

DP_scaffold1346 ...A...................T............T....G.A.............C......A..TA..C................T...........

6810 6820 6830 6840 6850 6860 6870 6880 6890 6900

....|....|....|....|....|....|....|....|....|....|....|....|....|....|....|....|....|....|....|....|

LE_scaffold81 GTGAATAGATAGATTATTTTCTTGTCGGGTATCACTGGACGTTTCTTCACTGTCTGTTTT-------------------------------TGCCAAAGA

DP_scaffold1346 ....C..................T..A.A......CT..T.......T....C....C..GCCAGAGAGTTTTTAAAGAGTTGCTCAGCAGNNNNNNNNN

6910 6920 6930 6940 6950 6960 6970 6980 6990 7000

....|....|....|....|....|....|....|....|....|....|....|....|....|....|....|....|....|....|....|....|

LE_scaffold81 GTTCTTCAATAGAGAGCAAGGC----------------------------------TCTGGTTTACAAGTACCTGGACAAGTTCATCTGCAGCACTGCTT

DP_scaffold1346 NNNNNNNNNNNNNNNNNNNNNNACTTCTTCCCAAGGGGTACAACATAATCTCTTGT.T...............CA............A.......CA...

7010 7020 7030 7040 7050 7060 7070 7080 7090 7100

....|....|....|....|....|....|....|....|....|....|....|....|....|....|....|....|....|....|....|....|

LE_scaffold81 TGTTCAGGTTTACTGTCTGTATGAAAATAATGTGCTTAGTGGTTTAGAGCTTTCTGTCCTCTTAACAGTCCTGCAGATTTG---------CTATGTGTTT

DP_scaffold1346 .T..G...........................G.........C.................T...............C....GTGCAGGAG......A...

7110 7120 7130 7140 7150 7160 7170 7180 7190 7200

....|....|....|....|....|....|....|....|....|....|....|....|....|....|....|....|....|....|....|....|

LE_scaffold81 CAAGATGCTGGAAGTTGATTCCTTGCTTTCTTGGGAGGTCACCTTCTTGTTTCCTAAAAAAACAGGTTGGGAAAGGCTTTGTGGTTTTATTTTTTTAACC

DP_scaffold1346 ............G.............C..............GTC.............G.G.-T................GA.......C.......T.A.

7210 7220 7230 7240 7250 7260 7270 7280 7290 7300

....|....|....|....|....|....|....|....|....|....|....|....|....|....|....|....|....|....|....|....|

LE_scaffold81 TCTTTTTTTAGCTTCTTATGAAACA-GAGAGCTGCTTATAGGAGTTCTGTTATATAGTTTTATTGGTATGACTAAATAGAGGAGCCAAGAGATGACACCT

DP_scaffold1346 CTCC.....G......CG.A...A.A.....A.......G.A...GG....TC..........CA......A............................

7310 7320 7330 7340 7350 7360 7370 7380 7390 7400

....|....|....|....|....|....|....|....|....|....|....|....|....|....|....|....|....|....|....|....|

LE_scaffold81 TGGCATAGACCTTGTATACAGTGGAGAAAACCAAAAATTCTTACAAATTGAAGTTCATCTCTTACTACAGGCCCATCCATAAGTGGAAATGGGGGGAATA

DP_scaffold1346 ............G.........................................C.C....C..G......T.........G....G.............

7410 7420 7430 7440 7450 7460 7470 7480 7490 7500

....|....|....|....|....|....|....|....|....|....|....|....|....|....|....|....|....|....|....|....|

LE_scaffold81 ATGAGAAAAGAGAGTTGCCATGTATGTGATAATCCAGATGAGTATCATCCTTTCCTAGTGTTATGCTAATAGGATTACAGACTGCCATTGCAGCTGTCTT

DP_scaffold1346 ........C..--.....T...C......A.......C......A.T......T...C......................C....T.C....A.......

7510 7520 7530 7540 7550 7560 7570 7580 7590 7600

....|....|....|....|....|....|....|....|....|....|....|....|....|....|....|....|....|....|....|....|

LE_scaffold81 TAATCATTTGCTTGTTGTTGTGGTTTAACCCTACAGGCACATAAACACCACATAGCCCTTTGCTCACTCC-CCCAGCTCCCCAGTGGGGTGGAGGAGAGA

DP_scaffold1346 ....A.....T.........G..........------------------....GA..A..CA.......TG....C.C.A..T.....A...G.......

7610 7620 7630 7640 7650 7660 7670 7680 7690 7700

....|....|....|....|....|....|....|....|....|....|....|....|....|....|....|....|....|....|....|....|

LE_scaffold81 ACTGGGGCGGGGAT-----TTTCTACCTGGGCAGCACTATCTTGCCATGGTGTGGCATTTGAGATGAGTTTATCTTTACGCTGCCAGTTACTCTGTTTCC

DP_scaffold1346 .TCA...GAAAA.AAAAAA..CA.GGG.T.AG.TA.AGGCAG.TTA..A.GACA.A.AAG..AGG..AAA..A-..A.TAA.AAT.A.A.AAGAA.A.G.

7710 7720 7730 7740 7750 7760 7770 7780 7790 7800

....|....|....|....|....|....|....|....|....|....|....|....|....|....|....|....|....|....|....|....|

LE_scaffold81 ATTGCTGTAACTGCCGCCACAGGGCATTTGC-CACCATTTGTGACTCAGTATAGAGATAAAGCACTGGCCATTTTTCTCATTCAGCAATATCTAAAGCCA

DP_scaffold1346 .A---GA......AT..-.T.AT...A....T.....CC..CTGAC..A.G.CC..CC.GTTTCTGA..AGCAA.....--.GC..CCC.C.C..CT..C

7810 7820 7830 7840 7850 7860 7870 7880 7890 7900

....|....|....|....|....|....|....|....|....|....|....|....|....|....|....|....|....|....|....|....|

LE_scaffold81 GCTGGATGGCTTTC-ACCTCTGCAAACTGACGCTATTCACCTTCTCCTTCAGCATTTTCTGTGACTTGT--TGCATGGGACTCCATACAGCAGCCTTCCA

DP_scaffold1346 C.CA.T.TATA.A.TGAG.A..AC.T.AT.T.GC..GG.ATAG.C.T..GGCT.G...GG..C.GC...CC..GC..TAC.C..TCCT...T..-..GTG

7910 7920 7930 7940 7950 7960 7970 7980 7990 8000

....|....|....|....|....|....|....|....|....|....|....|....|....|....|....|....|....|....|....|....|

LE_scaffold81 CCTCTGATGCTTTCCCATAAGACAAGATCCATCCGTGAACAGTGCA--TACTGCTTCTCAGTTTCTGG-----TAGTTTATTAT--ACAGTGGGGCTTCT

DP_scaffold1346 .AC...GCAGA.CATGGG...CTG.A.AA-G...T...CTG...T.AGC...A...GG..ACAA..AAAATATC...G.G....CA...T.TATT..CA.

8010 8020 8030 8040 8050 8060 8070 8080 8090 8100

....|....|....|....|....|....|....|....|....|....|....|....|....|....|....|....|....|....|....|....|

LE_scaffold81 -------TCAACATGCATCACC---TCCTCCTCTGGCAATATTCTGAAATCTTTGCCTTCTGG---CCAGTCAATGATCACTTC--------CAAGATTC

DP_scaffold1346 ACTAAGTC...A.CA..G...TATG..AG.TA..A.G..G.AAA.T..C...A.C..AG.C.AAAC.AG.A..G.ATC...CC.TTATTCTA..TC..CT

8110 8120 8130 8140 8150 8160 8170 8180 8190 8200

....|....|....|....|....|....|....|....|....|....|....|....|....|....|....|....|....|....|....|....|

LE_scaffold81 CTGGGCAACTGGGGTTTCCTATTTGAGCCTGTTGTGTGATCAGTGTGACCCACTTACTCCACGTAGCATCAGTTGCATGATGTGTA-CAGGGGATCCTCC

DP_scaffold1346 A..TCATG.CCA...CCTACCC..TCCAA.ACAT.TCA..T.A.CACCA......T.C.TGTCACA..CAGA.AT...TCCC.TAGT.T.T..GC.A...

8210 8220 8230 8240 8250 8260 8270 8280 8290 8300

....|....|....|....|....|....|....|....|....|....|....|....|....|....|....|....|....|....|....|....|

LE_scaffold81 CTTTGAACGTTCAGCCCAGCACAGGCAGTTGGGGTGTCAGGAGGAGCTGTGCTTC---------------------------------------------

DP_scaffold1346 ....AG.ATG..C.TTG..TTA.TTT...CT.TTAC.TT..GCTCCA.C..TCATAACAGTCTTTTAGAGCAGGAGAGATGGTGTGTGGTGTTGGATTGT

8310 8320 8330 8340 8350 8360 8370 8380 8390 8400

....|....|....|....|....|....|....|....|....|....|....|....|....|....|....|....|....|....|....|....|

LE_scaffold81 ----------------------------------------------------------------------------------------------------

DP_scaffold1346 TGCATGCTGAAGCCAGTTCTGGTTCCATCACTGCTGCACTTTGCTTGGTTTCATCGAAGTTCATTCCTCATTAATCTGGGTGATTGTTACTGTAATACCA

8410 8420 8430 8440 8450 8460 8470 8480 8490 8500

....|....|....|....|....|....|....|....|....|....|....|....|....|....|....|....|....|....|....|....|

LE_scaffold81 ----------------------------------------------------------------------------------------------------

DP_scaffold1346 TTGCTATGGCATATAGCAACCATGAAAGAGATGACATACAGTATTATATAGCAATTAACATAATACCCTTCAGTTCATTGGCTATTTTCACCCAGAATCA

8510 8520 8530 8540 8550 8560 8570 8580 8590 8600

....|....|....|....|....|....|....|....|....|....|....|....|....|....|....|....|....|....|....|....|

LE_scaffold81 ----------------------------------------------------------------------------------------------------

DP_scaffold1346 AATCCCCTTGAGGTACACATCGGAATTCCCCATCCTTTCGCATTACCCACAAAGTGCACCCAGGTCCTTGAGCAAAAGCAATCCCACGAATGGTGTTCCT

8610 8620 8630 8640 8650 8660 8670 8680 8690 8700

....|....|....|....|....|....|....|....|....|....|....|....|....|....|....|....|....|....|....|....|

LE_scaffold81 ----------------------------------------------------------------------------------------------------

DP_scaffold1346 TTGCCCGAGGCAGGAGTAACCCAGACTGTCTTCCCCAGCATATTTTTTACGTGTACTACAGGGACTTTATCCCTTTTACTGTATGTAAAGGTTTTGATTG

8710 8720 8730 8740 8750 8760 8770 8780 8790 8800

....|....|....|....|....|....|....|....|....|....|....|....|....|....|....|....|....|....|....|....|

LE_scaffold81 ----------------------------------------------------------------------------------------------------

DP_scaffold1346 GGCAGGGCCTGCTTGATTGGCAGATCCCCTAGTATTGACTAACCAGGTGGCTTTTGCTAAATGTGTATCCCGATGTTTGAATATCCCAGCTCTCAGTGTA

8810 8820 8830 8840 8850 8860 8870 8880 8890 8900

....|....|....|....|....|....|....|....|....|....|....|....|....|....|....|....|....|....|....|....|

LE_scaffold81 ----------------------------------------------------------------------------------------------------

DP_scaffold1346 GTCTTTAACAGTCCATCGTATCATTCGATTTTCCCAGAGGATAGTTCTATGATAGGGGATGTGATATACCCACTCAATGCCATACTCTTTGGCCGAAGTG

8910 8920 8930 8940 8950 8960 8970 8980 8990 9000

....|....|....|....|....|....|....|....|....|....|....|....|....|....|....|....|....|....|....|....|

LE_scaffold81 ----------------------------------------------------------------------------------------------------

DP_scaffold1346 CCTATGAGGTTGTTTCAGAAATGAGTCCTGTTGTCTGACTCTGTTTTTTCTGGGGTGCCACGTCACCATAGGACTTGCTTTTCAAGGCCCAGGATAGTGT

9010 9020 9030 9040 9050 9060 9070 9080 9090 9100

....|....|....|....|....|....|....|....|....|....|....|....|....|....|....|....|....|....|....|....|

LE_scaffold81 ----------------------------------------------------------------------------------------------------

DP_scaffold1346 TCTGGACATTGGCATGGAGCACGGGATATGTTTCCAGCTATCTGGTGGCTGCTTTCACCATGGTGAGCACATAGCACTTGCCTTGGTGGGTTTGCAGGAG

9110 9120 9130 9140 9150 9160 9170 9180 9190 9200

....|....|....|....|....|....|....|....|....|....|....|....|....|....|....|....|....|....|....|....|

LE_scaffold81 ----------------------------------------------------------------------------------------------------

DP_scaffold1346 TGTGGTAAAATTAACATGCTAGACCTCCCCATATTTATATTTCAGCAATTGTCCTGCTTACCAGAGAGGTTTGAACTGCTTGGCTTGCTTGATTGCAGCA

9210 9220 9230 9240 9250 9260 9270 9280 9290 9300

....|....|....|....|....|....|....|....|....|....|....|....|....|....|....|....|....|....|....|....|

LE_scaffold81 ----------------------------------------------------------------------------------------------------

DP_scaffold1346 CATGTTTGACATTCATGGATAACCTGTGCCATAGTGTCTACGGTCAAGTCCACCCCTCGATCATGAGCCCATCTATATGTTGCATCCCTTCCTTGATGGC

9310 9320 9330 9340 9350 9360 9370 9380 9390 9400

....|....|....|....|....|....|....|....|....|....|....|....|....|....|....|....|....|....|....|....|

LE_scaffold81 ----------------------------------------------------------------------------------------------------

DP_scaffold1346 CTGAGGTGTCATGGGCCCACCAAGCTATAAATAATTCACACTTATGTTGCCAGTCCAGATCCACCTGAGCCGCTTCAATCTTAGCAGCCTGATCCACCTG

9410 9420 9430 9440 9450 9460 9470 9480 9490 9500

....|....|....|....|....|....|....|....|....|....|....|....|....|....|....|....|....|....|....|....|

LE_scaffold81 ----------------------------------------------------------------------------------------------------

DP_scaffold1346 CTGGTTGTTTTGAAGTTCTTCAGTGGCCGGAATCCTGGGTACATAAGCATCTACATGATGTACTTTTACAACCAGGTTCTCTACCCAGGCAGCAATACCT

9510 9520 9530 9540 9550 9560 9570 9580 9590 9600

....|....|....|....|....|....|....|....|....|....|....|....|....|....|....|....|....|....|....|....|

LE_scaffold81 ----------------------------------------------------------------------------------------------------

DP_scaffold1346 TGCCACAATGTGGCAGCTCAGATGGGTTTGCCTCTGTGCTGCCGGTTGCTCTGCTTCCGTTCCTGTAACCACCATCCATGAGTCAGTATGGAGATAAAGC

9610 9620 9630 9640 9650 9660 9670 9680 9690 9700

....|....|....|....|....|....|....|....|....|....|....|....|....|....|....|....|....|....|....|....|

LE_scaffold81 ----------------------------------------------------------------------------------------------------

DP_scaffold1346 ACTGGCCATTTTTCTTGTTCAGCAATATCTAAAGCCAACTGGATGGATCTCACCTCCGCAAACTGACTCGATTCACCTTCTCTTTCCTGAGTTTCTGTGA

9710 9720 9730 9740 9750 9760 9770 9780 9790 9800

....|....|....|....|....|....|....|....|....|....|....|....|....|....|....|....|....|....|....|....|

LE_scaffold81 ----------------------------------------------------------------------------------------------------

DP_scaffold1346 CTTGTGTAGGACTCTATACGGCAGCCAGCCTTCCACCTCTGATGTTTTCCCACAAGACAACAGGACCCATTAGTGAACAGGGCACATTGCTTCTCATTTT

9810 9820 9830 9840 9850 9860 9870 9880 9890 9900

....|....|....|....|....|....|....|....|....|....|....|....|....|....|....|....|....|....|....|....|

LE_scaffold81 ----------------------------------------------------------------------------------------------------

DP_scaffold1346 CTGGTAGTTTATTATACAGCAAGGCCTCTTTAGCACACCCCACCTCCTCCTCTAGTGATATTTTGAAATCTTTGCCTTCTGGCCAGTCCATGATCATTTC

9910 9920 9930 9940 9950 9960 9970 9980 9990 10000

....|....|....|....|....|....|....|....|....|....|....|....|....|....|....|....|....|....|....|....|

LE_scaffold81 ----------------------------------------------------------------------------------------------------

DP_scaffold1346 CAAGATTCCTGGGCGACTGGACTTTCCTATTTGAACCCATTGTGTGATCAGTGCGACCCACTTACTCCACGTAGCATCAGTTGCATAATGTGTACAGGGG

10010 10020 10030 10040 10050 10060 10070 10080 10090 10100

....|....|....|....|....|....|....|....|....|....|....|....|....|....|....|....|....|....|....|....|

LE_scaffold81 --------------------------------------------------------------AGTACCAATCACTTCCGAAGGGCTACTCGAAGACAAAC

DP_scaffold1346 ATCTTCCCTTTGAACATCCAACCCAGCACCAGCAGCTGGGGTGCCAGGAAGAGTTGTGCTTC................A..TA...G.A.A...G.....

10110 10120 10130 10140 10150 10160 10170 10180 10190 10200

....|....|....|....|....|....|....|....|....|....|....|....|....|....|....|....|....|....|....|....|

LE_scaffold81 AATCTTTTTGCAGTTTTCAAAGCATGACTTCACATAGCCATCAGGTGAAAACT------------GTGATCCAAAACGTCATCTCCAAAATGATTCTGAT

DP_scaffold1346 ........CA................G.....A.C.A.............G..TGTGTCTTTGGAAG........GT............-..G......C

10210 10220 10230 10240 10250 10260 10270 10280 10290 10300

....|....|....|....|....|....|....|....|....|....|....|....|....|....|....|....|....|....|....|....|

LE_scaffold81 TAAGAAAGCGTCTGACTC----ATTGGAAAAGAAGACTACAGAGAACACCACTCAGTAGAAAATCTACAAAATACTGACTTTGAATGGATATGACCCCGC

DP_scaffold1346 C........A.......ACCTG.C.........T.....TG....-..T......AA.C......................................A..

10310 10320 10330 10340 10350 10360 10370 10380 10390 10400

....|....|....|....|....|....|....|....|....|....|....|....|....|....|....|....|....|....|....|....|

LE_scaffold81 CTATGTAGGAAGCATATTCGTTAGGTGGTGCAGAATAACCTTCAA-AAGAGGAACAAGATATGGCTGTATGGACCTGCCAACACCAG-AAAACCAACATC

DP_scaffold1346 ...CA.G.......C.......G............G.GA......T..C.......T...........................TG.G...........T

10410 10420 10430 10440 10450 10460 10470 10480 10490 10500

....|....|....|....|....|....|....|....|....|....|....|....|....|....|....|....|....|....|....|....|

LE_scaffold81 GCTTAAGCTATATTGACTCATGTACCCTTTCACAAATGCGTC----GGTCAAATGAGAACTTTCTGTTTAACAATACTGTGGACAAAACAGTTATTTGGT

DP_scaffold1346 ...C.G...G...C....GCCA.G........TGGG..T...AATT.......G..C...C...A..A....G..GTCA.........TG..........

10510 10520 10530 10540 10550 10560 10570 10580 10590 10600

....|....|....|....|....|....|....|....|....|....|....|....|....|....|....|....|....|....|....|....|

LE_scaffold81 GGGAAGAGGGCAGATCGGGACACCGTGCCTCTTCGCGTACCTCATATAATG-CAATATATGTGTCACGTTGGATAATGAACTGAAACAGACTTTTAAAAG

DP_scaffold1346 ................AA......T........T.AC...T.........TT..............T..C.......T.G....................

10610 10620 10630 10640 10650 10660 10670 10680 10690 10700

....|....|....|....|....|....|....|....|....|....|....|....|....|....|....|....|....|....|....|....|

LE_scaffold81 AAAACCCTTTTCCTGTTACAGAAAGGAACATGGATTCCCATGATGTGTACAGTGCAGAGGGGAACTCAATACCCAATTAATCGTTCTTTGCATGGCATTT

DP_scaffold1346 ...T.....................-....-.A...T....C..................AA.................G.A....AC............

10710 10720 10730 10740 10750 10760 10770 10780 10790 10800

....|....|....|....|....|....|....|....|....|....|....|....|....|....|....|....|....|....|....|....|

LE_scaffold81 AAAATCAGTATAATGATGCCAAAATCAGCAATGGATTATAACAGCAAGCTTTTCTTTGCTTCAGAAAAGTTTTAAATATTCATAGACAGGATCTTACTTG

DP_scaffold1346 .............A...A.........--------............C...................TA......G.........C............-.

10810 10820 10830 10840 10850 10860 10870 10880 10890 10900

....|....|....|....|....|....|....|....|....|....|....|....|....|....|....|....|....|....|....|....|

LE_scaffold81 AGAGGAAGCTCTGCAGAAGATGAGGATCTTCCTTTGTTCCTGTTCTCTGGACAGTGGATTTTCCTGGGGGCAAGG-AGCAGAATTTGGTGACATTTAGAG

DP_scaffold1346 .........................G.....T......G.................A....C..CAA...TG.A.T..................CC....

10910 10920 10930 10940 10950 10960 10970 10980 10990 11000

....|....|....|....|....|....|....|....|....|....|....|....|....|....|....|....|....|....|....|....|

LE_scaffold81 GAAGCCACAAAACCTCACTGTGATGCCCACCTATGAACAGACTCGCCTAAGAGGAGAAGAGAATCAAATGATGCCGTCTATCACCATCTGCCTGTTATCT

DP_scaffold1346 .......T............CA....T..G..............A...............A...........A.AA......................T.

11010 11020 11030 11040 11050 11060 11070 11080 11090 11100

....|....|....|....|....|....|....|....|....|....|....|....|....|....|....|....|....|....|....|....|

LE_scaffold81 GCAATTGTAGTTAGATATTGTAA---GGAGAAACGATCGTTCAGTGAGTGAGTTCAGTTCAATTTCCTTCCTCCCTACAGTGCTCAAGGATAGATTGTTG

DP_scaffold1346 .........T.............CGA.......T...G.......T.......A.................C.AT.G...A.........C.........

11110 11120 11130 11140 11150 11160 11170 11180 11190 11200

....|....|....|....|....|....|....|....|....|....|....|....|....|....|....|....|....|....|....|....|

LE_scaffold81 AGCTCTTTCTGCTTAACAGGATAACTACCTTGACTTATAAAGTCAAATGGTCTGACTCACGCCTTAGACACTGAAAAATAATAACTTGGTCACTGCTTAG

DP_scaffold1346 ..T....GT....C.......................C.....................GT.........A......TGGG..G.......T........

11210 11220 11230 11240 11250 11260 11270 11280 11290 11300

....|....|....|....|....|....|....|....|....|....|....|....|....|....|....|....|....|....|....|....|

LE_scaffold81 CTTCCCAAATGTAAATAATAGTGCAGAGGTGTTCTGAGGTTTTGAGAAGAATTGTGTGATACTTGGCTTGGATACAAGGGCAATGGGAGCAACAGAAGAG

DP_scaffold1346 .C................C..CA......................A.....GCA....................T....-......A...........C.

11310 11320 11330 11340 11350 11360 11370 11380 11390 11400

....|....|....|....|....|....|....|....|....|....|....|....|....|....|....|....|....|....|....|....|

LE_scaffold81 GCAGAACATAATAGATAGCCTATCTGATAAAGACGGAAAAGCATTTATGATAAAGGTAACAGGAAAGACCCTTGGAACTGCTACAGTTTTGTTCTGTCTT

DP_scaffold1346 ......TGC...G....................T......A...C.G...C...A.............GT......G...T.......G......C....

11410 11420 11430 11440 11450 11460 11470 11480 11490 11500

....|....|....|....|....|....|....|....|....|....|....|....|....|....|....|....|....|....|....|....|

LE_scaffold81 CTGACTGAATGGGGAAACTCCTGCAAGCACTGAAAAGTGTCCTTGTGGTACTGCATTTGAAGTTAGGGACTTTTCCTGGAAACAACTTAAGCACAACTCC

DP_scaffold1346 ......................AA.....T.........................C.C..........................G......TG.......

11510 11520 11530 11540 11550 11560 11570 11580 11590 11600

....|....|....|....|....|....|....|....|....|....|....|....|....|....|....|....|....|....|....|....|

LE_scaffold81 AGCCAGGTAGTAAAACCCCTCCTCAGATCTTCACCCTAAGAACT--TAGCTCTGATGTATTAAGATAATGGCTTAGTTAGTTAG---------CCAACTC

DP_scaffold1346 ........TA.....T...........C...........C....GAC...........G......C.C.......T....CC.ATGCTGCCTT..T.T..

11610 11620 11630 11640 11650 11660 11670 11680 11690 11700

....|....|....|....|....|....|....|....|....|....|....|....|....|....|....|....|....|....|....|....|

LE_scaffold81 ATTTCCCAGTGTACAGGAGTTAAAGAACTGCGTAGTGTTAAAACAC-CCTTGAATGCCCCTAAGACCTGTTTGTATGCTTCTGGCTGGAGGAGTTGTTAA

DP_scaffold1346 .......G.....................AAA..............A.T..............C.....C...-.........A.........C....CT

11710 11720 11730 11740 11750 11760 11770 11780 11790 11800

....|....|....|....|....|....|....|....|....|....|....|....|....|....|....|....|....|....|....|....|

LE_scaffold81 GTCATTCTTGT------------------GGTAACTGTGTACATCTAATTGTGCATGTAGTCTAATGCACATCAACCAAATAAGATTGCCCCATGGCTGT

DP_scaffold1346 ...........CTGACAAGAGGTCTTTGTA......C.....C...G........CA..A.T..............T.....G..............A..

11810 11820 11830 11840 11850 11860 11870 11880 11890 11900

....|....|....|....|....|....|....|....|....|....|....|....|....|....|....|....|....|....|....|....|

LE_scaffold81 GCGTGGTGTCAGAAGGGTTTTGAAACTCTCGTTGTCTTCAGTTTTAGAAAACTTGAAACTCTCCACTTTTCTTTGTTCTGGCTTTGTATTGCTTGACCTT

DP_scaffold1346 ..T......T...................T.....T..GG...................................G..............T.....T...

11910 11920 11930 11940 11950 11960 11970 11980 11990 12000

....|....|....|....|....|....|....|....|....|....|....|....|....|....|....|....|....|....|....|....|

LE_scaffold81 CACGTTCAGTCTCAAAAA-GAGGTTTGGCTATATGAGGCATCACATAACTGTGTGCATAGTCACAGATGTGCACAGTTGGGTACTATCTGAGGCTTTCCC

DP_scaffold1346 ..T.......T.......A...A............C-------------------......A...A..C.------------------------------

12010 12020 12030 12040 12050 12060 12070 12080 12090 12100

....|....|....|....|....|....|....|....|....|....|....|....|....|....|....|....|....|....|....|....|

LE_scaffold81 AAACATGCTTTCATTCCTTCCTCCAGCTCATGTGTCCAGATGCTTCCTGAGAGAAGCCTAGAACCAAACATAAGAGAAAGTATTTTGCCCTTTCAAATTT

DP_scaffold1346 -------...C...--------------------------------------------------------------------------------------

12110 12120 12130 12140 12150 12160 12170 12180 12190 12200

....|....|....|....|....|....|....|....|....|....|....|....|....|....|....|....|....|....|....|....|

LE_scaffold81 TAATCACTGAATGTTACTGATCCCCTTGCTTGGGGTTGGTACAAGTTTTTCGCTGTTCTAAGCAGGGTAGAAAATTGCCAGCACCCTTTTTTTCCCCATC

DP_scaffold1346 -----------------------------------------------------------------------------------------------...C.

12210 12220 12230 12240 12250 12260 12270 12280 12290 12300

....|....|....|....|....|....|....|....|....|....|....|....|....|....|....|....|....|....|....|....|

LE_scaffold81 TTCACAGGTTTTATACACGTGCAGTCAATGCTGATTGGATCCTCCCTTTTCAGGCACCCAGCTGCCTGAATGCTCACCCCAGTCTGCTTGCCAGTTGTAT

DP_scaffold1346 ...-----------------------------------------------------------...G....------------------------------

12310 12320 12330 12340 12350 12360 12370 12380 12390 12400

....|....|....|....|....|....|....|....|....|....|....|....|....|....|....|....|....|....|....|....|

LE_scaffold81 AAGCTTTACAGCACCATATTTTTAGTCAATCCAATCTGAAATTGAGCATCACCTCAAATGCTCTACCTTTAAAAAAAAAAACAAAACCCCAAACCAAACC

DP_scaffold1346 ----------------------------------------------------------------------------------------------------

12410 12420 12430 12440 12450 12460 12470 12480 12490 12500

....|....|....|....|....|....|....|....|....|....|....|....|....|....|....|....|....|....|....|....|

LE_scaffold81 CAAAAGGAACAAACAAAAAACAAAACCTGAAAAAACAAACCACAAAACCTAAAATCCAATCCCCCCACCTCCCCCAAGCCCCAACCTCACATGGCTTAAT

DP_scaffold1346 ----------------------------------------------------------------------------------------------------

12510 12520 12530 12540 12550 12560 12570 12580 12590 12600

....|....|....|....|....|....|....|....|....|....|....|....|....|....|....|....|....|....|....|....|

LE_scaffold81 TGTCTGGGATCTCCCACTGTAGAATGACTGTTATGCCATGTCTGTCATGACAAGAGATGCATCCAAGCTGGAGTAAGGTTGGGCCCCTTTTGATAATTTC

DP_scaffold1346 ----------------------------------------------------------------------------------------------------

12610 12620 12630 12640 12650 12660 12670 12680 12690 12700

....|....|....|....|....|....|....|....|....|....|....|....|....|....|....|....|....|....|....|....|

LE_scaffold81 AGTGGTTGGGTTGCACTGTTCCTCCTTGAATATCTCACCTTTTCTGCCATGGCAGAACTCTCCTCAGTCATCACCTTTGCAAGCTTAAGGTGCCTTATGC

DP_scaffold1346 ----------------------------------------------------------------------------------------------------

12710 12720 12730 12740 12750 12760 12770 12780 12790 12800

....|....|....|....|....|....|....|....|....|....|....|....|....|....|....|....|....|....|....|....|

LE_scaffold81 CATGGCAACAGATCTTTTCCAGCCACCCTCAGAAAAAAATGTCTTGTCAAGTAATGACTGAAGTTTCATAATTCTTTTCCCATGTTGAATGGTCCATTAT

DP_scaffold1346 ---------------------------------.G....A.......A.---------..................C....T.........C........

12810 12820 12830 12840 12850 12860 12870 12880 12890 12900

....|....|....|....|....|....|....|....|....|....|....|....|....|....|....|....|....|....|....|....|

LE_scaffold81 TTTGTACAGAAACTCTTTTTTTTTTTTGTATGTTTTTTTCTTCTTTCATTTGAAAATTGAGCTGCTTCCATGAGCCAACCCATTATGAGCTTGGGAGAAT

DP_scaffold1346 .....GG....G.......C.G.A.A.T.---.C.....AAC....G.........C......A.........CA...G.........A...CA......

12910 12920 12930 12940 12950 12960 12970 12980 12990 13000

....|....|....|....|....|....|....|....|....|....|....|....|....|....|....|....|....|....|....|....|

LE_scaffold81 CAAAGCTGATTTTATGTCTGGAGGGGGAGACGTTGTGAAACTTAAAGGATCTATAGGCTTTTGACTGCAAACCAGAGAAACTACAGCAGTTTTAGTCTTG

DP_scaffold1346 ..............CAG......A..-....C....A.................G.C..........T..............G.........-.......

13010 13020 13030 13040 13050 13060 13070 13080 13090 13100

....|....|....|....|....|....|....|....|....|....|....|....|....|....|....|....|....|....|....|....|

LE_scaffold81 TTTGCCATGGCTCAGATTTGCTGTCAGATACATCTCTCTCTTGTATGATTGTCTTCAAAGCATTTCTCCTATTGATATGCTTGAATGATAGCATGCTATG

DP_scaffold1346 A...T.............-.........G............C...........................CC.............................

13110 13120 13130 13140 13150 13160 13170 13180 13190 13200

....|....|....|....|....|....|....|....|....|....|....|....|....|....|....|....|....|....|....|....|

LE_scaffold81 TTTTGAATAGGGTGCTGACGCTGCAAAACAACAAATTTATGTTTACATAAGTAGTCATTGCTATTTCTTTTTTTAT---------GCTTTTATCAAAAGG

DP_scaffold1346 ...........C......G.......G.T.......G.....................................T.TTTTTTAAA.T.......G.C...

13210 13220 13230 13240 13250 13260 13270 13280 13290 13300

....|....|....|....|....|....|....|....|....|....|....|....|....|....|....|....|....|....|....|....|

LE_scaffold81 GATGTGACATTTGTGTCAAAACTGTCTCTAAACACTTCTCCAATGTTATTGGTACAACAGCCTTTGTTAAGAAGGCTTTAGGGTTCTGCAAAAACTAAAT

DP_scaffold1346 ................A....T..A........................A..C.T..........A.................................G

13310 13320 13330 13340 13350 13360 13370 13380 13390 13400

....|....|....|....|....|....|....|....|....|....|....|....|....|....|....|....|....|....|....|....|

LE_scaffold81 CTAATCTTTGAATAATAACATATGTAAGCTTTACTTCAGAAACCTACTGGAAATAGTAGAAAACTGACAGAAAGTTGTCATGGAAACCACTGACACCTAG

DP_scaffold1346 ...................G.............A..A.A.................G..................................A........

13410 13420 13430 13440 13450 13460 13470 13480 13490 13500

....|....|....|....|....|....|....|....|....|....|....|....|....|....|....|....|....|....|....|....|

LE_scaffold81 ATATGGGTGAAAAAAGAAGCTTTCATTCAGAGACCACAATAATTTGCAAAAGAATCTGTAGGTTTCTGAAATATTTTTTTCTACTCAGTTCTGATGGTAG

DP_scaffold1346 .............C..........................T.......G.....A................C.......-.....T..............

13510 13520 13530 13540 13550 13560 13570 13580 13590 13600

....|....|....|....|....|....|....|....|....|....|....|....|....|....|....|....|....|....|....|....|

LE_scaffold81 GACTGCTTTTAATTTTCATAAACGTATGTGTGGATTCTCATACGTACACAAATGAGGGAT------------GCTTTGTGCTGGATGAATAAAAAATAGT

DP_scaffold1346 .......................A...A.........A..C..A...T.........A..ATTGTGTATTGT.......................G....

13610 13620 13630 13640 13650 13660 13670 13680 13690 13700

....|....|....|....|....|....|....|....|....|....|....|....|....|....|....|....|....|....|....|....|

LE_scaffold81 TTCCGAAGACGAAATTGCTGAGAACCAAGGACACAGACTAAGAGGAGGATTTTATTGGCCTCTTTGTGTTTGATTAAGGCTTTTTCATTTTTGGTAAAAT

DP_scaffold1346 ..TG.....T......T...........A..GG.........................T.....-......A.....AA..............A-.....

13710 13720 13730 13740 13750 13760 13770 13780 13790 13800

....|....|....|....|....|....|....|....|....|....|....|....|....|....|....|....|....|....|....|....|

LE_scaffold81 GTGTTTAATTCTTGTTGACTGAGCACAAGGGAGAAAACTGTGCTTATAAAATCAATGTATGGGCATGATCCAGAATAGAGAGAGAATATCTGACAAGTCT

DP_scaffold1346 .............................A.......A.....A........A......CA..T.....GTG.....A..----................

13810 13820 13830 13840 13850 13860 13870 13880 13890 13900

....|....|....|....|....|....|....|....|....|....|....|....|....|....|....|....|....|....|....|....|

LE_scaffold81 GGCTGGTCAGGAAACAAAATAGCAGCTGAAGTAA----GAAGAAAGGAGGACTGGATTTATTGCCTGTATTTTAGATAGTCTTCTATTGAGTTTTTCTTG

DP_scaffold1346 .....A........T..........T....A...AGAA..........A....A.G.....A......G.........C.....................

13910 13920 13930 13940 13950 13960 13970 13980 13990 14000

....|....|....|....|....|....|....|....|....|....|....|....|....|....|....|....|....|....|....|....|

LE_scaffold81 CTAAAAGTAACTCAGCAGTTACTCAACATTCCTTGCAAATATGTAGTAAGTTTCTAAATATGGATT-ATGCAGCAATTTTTTTTAAATTCAGATTTTTAA

DP_scaffold1346 .....G......T....................A.......CA.....T..A..............T.CAG...........AA......GAT.C.AA.C

14010 14020 14030 14040 14050 14060 14070 14080 14090 14100

....|....|....|....|....|....|....|....|....|....|....|....|....|....|....|....|....|....|....|....|

LE_scaffold81 ATTAT--TGGTTGATGATAGGTCTGCATCATTTAGGTAGACTTTTAAAAATGTCACCTTCTCTTTCTTTACAGAGCTCTTTCAACTCAAATATTCAAGTG

DP_scaffold1346 ...G.GT...A..T...A........G..C.........T.....T............C....A......T...A.AA................T.....

14110 14120 14130 14140 14150 14160 14170 14180 14190 14200

....|....|....|....|....|....|....|....|....|....|....|....|....|....|....|....|....|....|....|....|

LE_scaffold81 TTACAATAATCTTTGGAATGTACAGAACTGACAGTTCTGCACAGAAAATTCACACTATGTTCTCAAAGGCCTATTATATACACTTTGAGAAATCTGAAGT

DP_scaffold1346 ................................................--.G......A..G........---------........T............

14210 14220 14230 14240 14250 14260 14270 14280 14290 14300

....|....|....|....|....|....|....|....|....|....|....|....|....|....|....|....|....|....|....|....|

LE_scaffold81 ACAGAGTGTCTTCTAATACAGAAAGACAAATCAGATCTTGAAATTTTTTTGTGTTCTTCAGGTTGATCAGAAAA-CACATCTACAGAAATGATTTGAGAT

DP_scaffold1346 .....A..A....A....GG.........G.........C...........C............C.........AT.TG...G......A..........

14310 14320 14330 14340 14350 14360 14370 14380 14390 14400

....|....|....|....|....|....|....|....|....|....|....|....|....|....|....|....|....|....|....|....|

LE_scaffold81 GGTTCAGCAAGACAAGTAGAATTTAAAACCATGTTTCAAGCCCACTGTGACAGAAGAAAATTCATGACTGTTTATACAAATTGTCCACAAAGCCCAGAAC

DP_scaffold1346 .....................C...G...A.....C...........G..G.T...C.C......A..........G..................C...G

14410 14420 14430 14440 14450 14460 14470 14480 14490 14500

....|....|....|....|....|....|....|....|....|....|....|....|....|....|....|....|....|....|....|....|

LE_scaffold81 TTTATTTCTAGGGAGTGGACATATACGGCCAGATACCTGGG-GAAGAGT-GAGAATGGGTCAAACATACGTGCTATTTCTACCCTAATACTTTTCAGGCT

DP_scaffold1346 ...............C.......C..A.G.....G......A.......T.....CA......T.....A.........C......G.............

14510 14520 14530 14540 14550 14560 14570 14580 14590 14600

....|....|....|....|....|....|....|....|....|....|....|....|....|....|....|....|....|....|....|....|

LE_scaffold81 GTGACTACACTC-CCTCAGAGAATTTCCTGAGCATGCTTATGCCTAGTCTGCTTTG-TGATCTCCAATGCATCTTTACAAACACCTGTT---TGCTTTTA

DP_scaffold1346 ......GT....A..............T...............T............C......A................G...AAAACAGG........

14610 14620 14630 14640 14650 14660 14670 14680 14690 14700

....|....|....|....|....|....|....|....|....|....|....|....|....|....|....|....|....|....|....|....|

LE_scaffold81 AACCTGCATTCAAAGTGGCAAGGAGTTCTGTAGTTCTACTCTACCCACTGCGTGAAGAACCACCCTCCTTAAATTTGTTTTGTACCAGTCTCATTGCAGC

DP_scaffold1346 .........G..................CA...G.........T....CA.ACTG.......................................G.....

14710 14720 14730 14740 14750 14760 14770 14780 14790 14800

....|....|....|....|....|....|....|....|....|....|....|....|....|....|....|....|....|....|....|....|

LE_scaffold81 TTCATAAGATGGACCCTACTTCACCCTGGAAGAAACAGGGAACAGGCAGTCCCTATCTCTGCTTTCTCTGTCATTCAGGATTTCATAGACTTCAACCATA

DP_scaffold1346 ......G........T.........T.............A.....T..A.................AT.........A..................T---

14810 14820 14830 14840 14850 14860 14870 14880 14890 14900

....|....|....|....|....|....|....|....|....|....|....|....|....|....|....|....|....|....|....|....|

LE_scaffold81 TCCCCCCATGTCATCTTT-CCCAGGCCATAAAGTCCTCTTCCTTATAGAGAAGCCATTGTGTACTTTGACAAGCCTTGCTGCTCTTGTCTGAATCTTTCC

DP_scaffold1346 -----.....C.......T.......TGA........T.........A..C.....C.......................A............C....G.

14910 14920 14930 14940 14950 14960 14970 14980 14990 15000

....|....|....|....|....|....|....|....|....|....|....|....|....|....|....|....|....|....|....|....|

LE_scaffold81 CAGTTCTAACAGATTCTTTTTCAGATGATGGGACCAGAACAACATACATTATTCAAGGTATGAACAAACCATCAATACGTACAATGGCACAATTAGGGAG

DP_scaffold1346 .....G.....T....C....G..GC................TG....................TG...........TA.......A..T..........

15010 15020 15030 15040 15050 15060 15070 15080 15090 15100

....|....|....|....|....|....|....|....|....|....|....|....|....|....|....|....|....|....|....|....|

LE_scaffold81 CATAATAACATTTGACTAGATTAGTGAGATTACCTCAGTTTCTGTAAAGTGGATAATTTCACAAAAAGCAGGCATGGCATTTCTCAGTTTGTTCATGAAA

DP_scaffold1346 .......G.........G......GA.....................T........CC...........................T..............

15110 15120 15130 15140 15150 15160 15170 15180 15190 15200

....|....|....|....|....|....|....|....|....|....|....|....|....|....|....|....|....|....|....|....|

LE_scaffold81 TGTTTATGTAGGTAATCTGACCCTTGAATTGCTATTTTTCTGCCATGCAACACTTATTTCCAAGACTCAGCACTGTACAGCAACAGGTTTCGTATCGATT

DP_scaffold1346 ...G......A.......C.T..A..T...C....G.........CA.....TG.........A.........AA....AT..........A....A...

15210 15220 15230 15240 15250 15260 15270 15280 15290 15300

....|....|....|....|....|....|....|....|....|....|....|....|....|....|....|....|....|....|....|....|

LE_scaffold81 TTGCAGAAAACACTCCTCAGATTTTGATCTTCATTGATG-GGATGGGTCTCAGGTCTTGAGACGTGCTAGCATGTATGGGGCTTTTTGTTGTTGTTTTTG

DP_scaffold1346 ......G...........T.G..G..G.......A..CCT.....A.....C......T...T...T.............................G...

15310 15320 15330 15340 15350 15360 15370 15380 15390 15400

....|....|....|....|....|....|....|....|....|....|....|....|....|....|....|....|....|....|....|....|

LE_scaffold81 G---CATCAGTATTTCTGTGAAGGCC-------TAATTCAGCTTGTTTCCATAACGCTGAGAAGGCTTAATAACATACAAGGGAT-----AGTAAGACTT

DP_scaffold1346 TTGT.............T.....A..GGGCACCC......A..........C..T..........T...............AAG.TAATA..........

15410 15420 15430 15440 15450 15460 15470 15480 15490 15500

....|....|....|....|....|....|....|....|....|....|....|....|....|....|....|....|....|....|....|....|

LE_scaffold81 AAGGTCAAGCTTTTAGCTGAAAGACCAGAAACACTTGGTGAAAAATGAGTAATAGCTGTAATTCTGTTCTGTCTTGGCAACTGGGTAGTCATCTAAACCC

DP_scaffold1346 ..............................C.................A...C.AT..............A...CAA............T........AT

15510 15520 15530 15540 15550 15560 15570 15580 15590 15600

....|....|....|....|....|....|....|....|....|....|....|....|....|....|....|....|....|....|....|....|

LE_scaffold81 TAAATTTTCTTTCTGAAGGTACTGTAGATATATTCTTGCTGCTAGGGTGGGGGGACTCAAAGTCCACAAAGAGTCCAGTGAATTTCTGCTGCTTGGATTC

DP_scaffold1346 ............................C..T.........T...ACAT.....G.................A.T.........G.....-.........

15610 15620 15630 15640 15650 15660 15670 15680 15690 15700

....|....|....|....|....|....|....|....|....|....|....|....|....|....|....|....|....|....|....|....|

LE_scaffold81 TGAGACAAAGCTGGAGAGAGAGGCTTGTGGCTCAGCATCAGACTACTCTG--CTGGCAAGTTCTTGCCGGAGTCTTTCAACCCTATCTTTCCCAACTTGG

DP_scaffold1346 ..................GC......A.A.....AT........G.....TC........C.......A.........C........C.....G......

15710 15720 15730 15740 15750 15760 15770 15780 15790 15800

....|....|....|....|....|....|....|....|....|....|....|....|....|....|....|....|....|....|....|....|

LE_scaffold81 AGACCTACCAGCTTCAAGGTGTGGTTTTCATTGAGTTACTCCAACTTTGGTATCTTCATTCAGTCATAAGTGGTGATGACTTCTCACTTTCTTTTTTTTT

DP_scaffold1346 ...T......A....G...............G..C......................C..................CA..........C.----------

15810 15820 15830 15840 15850 15860 15870 15880 15890 15900

....|....|....|....|....|....|....|....|....|....|....|....|....|....|....|....|....|....|....|....|

LE_scaffold81 TTTTTTTTTTTTTTTTTTTTTTAACCCTAAGCCTGGCTTCCTATTTCTAGTCCAATACAAATGCATTTTTGAAGCCTCGTGTCACTTGCTG-TGTGGTAT

DP_scaffold1346 --...C...............A...T....A..................A..........CCA.........T.A..T.CT..T.C.....C........

15910 15920 15930 15940 15950 15960 15970 15980 15990 16000

....|....|....|....|....|....|....|....|....|....|....|....|....|....|....|....|....|....|....|....|

LE_scaffold81 CTGGATATAAATGCAAATAAATTCACAGCTCTTCT----GGGGATAAGTGCCTTGGATGTGTGAGGGGTATTATTCCTGGTTCAGTTTGCTACCCTCTGT

DP_scaffold1346 T....---------.T....G..............TTCT...AT....G...........T..................-...........G........

16010 16020 16030 16040 16050 16060 16070 16080 16090 16100

....|....|....|....|....|....|....|....|....|....|....|....|....|....|....|....|....|....|....|....|

LE_scaffold81 CTCATGCAGAGTTTTAAAGTGATGCTGTGCTTCCTTGATCACTAATACTTGATCTTTCTGATGCGGGGTCATTTACTGTTACATGCTTGATCTCGACTAG

DP_scaffold1346 ..A...........................G...................A.......CAG..T.T.A..........AC....T.............T.

16110 16120 16130 16140 16150 16160 16170 16180 16190 16200

....|....|....|....|....|....|....|....|....|....|....|....|....|....|....|....|....|....|....|....|

LE_scaffold81 GAATAGTGAGGCAGTAACCTTATATCTTAATTAACCAGAATTCA-GATGCAGTGCCTATACTGACCTTCTGCTTTCATGTCAGCACTGAGAGAGCAGACA

DP_scaffold1346 .............A....T..G....................TGT.........TT..C.T...........................G...GA......

16210 16220 16230 16240 16250 16260 16270 16280 16290 16300

....|....|....|....|....|....|....|....|....|....|....|....|....|....|....|....|....|....|....|....|

LE_scaffold81 ATATGTGACTCTATAAAAAAATCCTAATACAAGAGTAACAAATCTGAAATGGCAAAATGATTACTCTTGAT---AGAGTAGAGGCCCTAGAGACAGAGCT

DP_scaffold1346 ...........AG.......-...CC.........C..G.........T......................TTAGC.........A.....A........

16310 16320 16330 16340 16350 16360 16370 16380 16390 16400

....|....|....|....|....|....|....|....|....|....|....|....|....|....|....|....|....|....|....|....|

LE_scaffold81 ACTGAATAAAGATGCAGACCCTGAGACACCTGGTGAGACCTGCTGTTGACTCATGCTAACAAGTATATGGAAAAGTAGCGTATGTATTCTGTAAAGCTCA

DP_scaffold1346 .............--------------............................T.....GT.....AA........T..G..................

16410 16420 16430 16440 16450 16460 16470 16480 16490 16500

....|....|....|....|....|....|....|....|....|....|....|....|....|....|....|....|....|....|....|....|

LE_scaffold81 TGGTTTCTTCTACATTATGAGTTTACCAGTGAAACTTCATGTTGAATAAGAACGGCATGTCTGGGCCAGATCCACCTAGTCCTGTATCTTGTCTCTGATA

DP_scaffold1346 ..................T.....G................C..........T.....A...............T..............-----......

16510 16520 16530 16540 16550 16560 16570 16580 16590 16600

....|....|....|....|....|....|....|....|....|....|....|....|....|....|....|....|....|....|....|....|

LE_scaffold81 GTGACAGTAGTGGCAATATAAGGCAGGACAGAAGAATCAAGCAAAGATACAGTTCCTGAGTCAAAGGTTGTGTCTTTGAGTTCAAAAGAGAGCAATGTGG

DP_scaffold1346 ............................T........A.................TG....A......A.C.......G...T..........T......

16610 16620 16630 16640 16650 16660 16670 16680 16690 16700

....|....|....|....|....|....|....|....|....|....|....|....|....|....|....|....|....|....|....|....|

LE_scaffold81 ATGGCAGGTCGAGTAGTGAGCTCTTTATTCAACCTGCCTCTTCAGACACCAGTGTGTGTGTGTTGAGGGCAGCCTCTGCTTCTCACTGTTTGCAAAGTGC

DP_scaffold1346 .........T......CA.............G.....T..C..T.G.....---...........G...............A.....C.C..........

16710 16720 16730 16740 16750 16760 16770 16780 16790 16800

....|....|....|....|....|....|....|....|....|....|....|....|....|....|....|....|....|....|....|....|

LE_scaffold81 TGCACCGACAGCACTGTCTACATGACCAAGGGCTGTAACAACTCAGACTGCAGGATTAATACCCTTCATCTGGACAGAAGGTAGAGCTCTGACCCAAAGA

DP_scaffold1346 .....T.....T.....G......G......A......TG..C.........AT.C.....T....T................A................

16810 16820 16830 16840 16850 16860 16870 16880 16890 16900

....|....|....|....|....|....|....|....|....|....|....|....|....|....|....|....|....|....|....|....|

LE_scaffold81 AGTCAAATATGATTGCTTATGTTTCTGAAGCTTGGATTGTGGCTGTATCTTGAAATGCTAAGGGTCTGCATTAAACCTGTTATAGAAATCATTCTTACAA

DP_scaffold1346 .........C........................AG........T............TG.....A.........G......G..................

16910 16920 16930 16940 16950 16960 16970 16980 16990 17000

....|....|....|....|....|....|....|....|....|....|....|....|....|....|....|....|....|....|....|....|

LE_scaffold81 AAGCTTAGTTGACTACTCTGCCTTGTTCTGTGGATACTAGTATAAACCAAGTAAAGGTTTAATAGGGATTTCGAGCAGGTAGCCCGC--TATTCATTTTG

DP_scaffold1346 ...........G...................T........G.....A.................A.......A............A.AC....T.....A

17010 17020 17030 17040 17050 17060 17070 17080 17090 17100

....|....|....|....|....|....|....|....|....|....|....|....|....|....|....|....|....|....|....|....|

LE_scaffold81 CATGAATGGTTATATACCCATGTCTGTTTAGCTATTCATTACCTTGCCCTTT-GGTACAATTGCTAACTAAAGACTAATTAATTCCTATTTTTAATAAAC

DP_scaffold1346 ..................................C............TT...C.....C........G.....G......C.............T.....

17110 17120 17130 17140 17150 17160 17170 17180 17190 17200

....|....|....|....|....|....|....|....|....|....|....|....|....|....|....|....|....|....|....|....|

LE_scaffold81 CAAGTGTACTTGGTGATATAATGATGAGCAACAGACTAAATCCATGGTGGAAGAGAATTACAGCTGGAGCCAGAAAGGGTTTAGGAGATCAACATAACAT

DP_scaffold1346 .....A.........CCG..................................................A......G.C.....A................

17210 17220 17230 17240 17250 17260 17270 17280 17290 17300

....|....|....|....|....|....|....|....|....|....|....|....|....|....|....|....|....|....|....|....|

LE_scaffold81 TTTTGGTGGGATAGAATGGTAGTTTAATAAAATATGAAACAGGATTAATGCTGCAGATGACCTTAAAATACTGAATAGCAAAGAGCAGAGTGGCAATTAC

DP_scaffold1346 A...T....A.A.........................................T..........G..................................T

17310 17320 17330 17340 17350 17360 17370 17380 17390 17400

....|....|....|....|....|....|....|....|....|....|....|....|....|....|....|....|....|....|....|....|

LE_scaffold81 ACATACATGTATAGCTGTAACTAGCTGATAGTAAGTGCCTGTAAGTTTTTAAAAATGACAATCAAAATGGTATAAGAGGCCCTAAATAATGAATGCTTGT

DP_scaffold1346 .....G......T.........G..................C.......C.....A...................A............C.....A.....

17410 17420 17430 17440 17450 17460 17470 17480 17490 17500

....|....|....|....|....|....|....|....|....|....|....|....|....|....|....|....|....|....|....|....|

LE_scaffold81 GTTCATATAGTTTAATGTGAATGCTATCTTACCAGAGAAGTAAACAAAAATAAACCCTA----TTCTCCCATTCTGTGAAAACATAAAAAAAAATTGGTT

DP_scaffold1346 .C.....C...............T..G.............C..............T...CTAT...................TG....C...--C...G.

17510 17520 17530 17540 17550 17560 17570 17580 17590 17600

....|....|....|....|....|....|....|....|....|....|....|....|....|....|....|....|....|....|....|....|

LE_scaffold81 TTGGAAAGAGAAATTCACTCAAGCAAATTTAACAGTGTTTGACTATGGTGTTTTATCTGCCTGGGCAGGCTCACCAGTCTCTCAGGCTGGCTTTCAATTC

DP_scaffold1346 .......C........TT.A..........C..............C.........A........C......TGA............T.A......C.C..

17610 17620 17630 17640 17650 17660 17670 17680 17690 17700

....|....|....|....|....|....|....|....|....|....|....|....|....|....|....|....|....|....|....|....|

LE_scaffold81 TCATGCAAACTGTATCCCTCTCTGTTCCAGGACTCCCACTGTGCACCAGGGATGTCCATGTGGCAGGATGTGTTCTTCTGCTGGGAGCGGTCCTTAACCT

DP_scaffold1346 .......G...........T..CA.........C.....C...T.TG....G....T...........C...................A...........

17710 17720 17730 17740 17750 17760 17770 17780 17790 17800

....|....|....|....|....|....|....|....|....|....|....|....|....|....|....|....|....|....|....|....|

LE_scaffold81 GCTCAGGGCACCTAGTGCTGCACTAAGCTCCCACAAACAGCAGATGATTTCCTTGGTGTCGTGTTCTTGGAGACTGTTAGCCATGGCT-----------A

DP_scaffold1346 ......C.TG.G.......................G.........T.....T..A......................A........GCTGATGCTGGGG.

17810 17820 17830 17840 17850 17860 17870 17880 17890 17900

....|....|....|....|....|....|....|....|....|....|....|....|....|....|....|....|....|....|....|....|

LE_scaffold81 ACACAGCCTGCCTCCACCAAGTTCTCCCAGAAATTTGAAGGCAGTATACTATATTTTTCCCAAGACCTTACTCATTATGTTTATAAATGCTTATTTTTAA

DP_scaffold1346 .TG...T.........G..G............C.....G....A..A...G.G...............C...TG.............A...C........

17910 17920 17930 17940 17950 17960 17970 17980 17990 18000

....|....|....|....|....|....|....|....|....|....|....|....|....|....|....|....|....|....|....|....|

LE_scaffold81 AAATAAACTCCAGCAAGCATTTATTATGTATC--AGTTCCTCTGAGATGTGTACTAGGTCCTTGCCTTAGCCTTTTGTAGTAACCTTTTAATT-AATTCA

DP_scaffold1346 .................T....C.C...C...TT.....AA.......T....A...................................C...T......

18010 18020 18030 18040 18050 18060 18070 18080 18090 18100

....|....|....|....|....|....|....|....|....|....|....|....|....|....|....|....|....|....|....|....|

LE_scaffold81 TAGCAACACCGAACAAAACTTATGAGACAATGCTGAGATTTGCTTATACCCTGTGTCAAAAATAAGGCATTATAGGAATGACTTGTGACTGAATTATCAT

DP_scaffold1346 C........A............CA...T..................C....A..........C...........A......T..................

18110 18120 18130 18140 18150 18160 18170 18180 18190 18200

....|....|....|....|....|....|....|....|....|....|....|....|....|....|....|....|....|....|....|....|

LE_scaffold81 TAACCTTCTGTTATTATTGAGTTTTAGGCTATGCACACTGAAATGACACTTCAATGACTTTTGTTAGAATTTTAAAACCTA----ATGCATTTTTAATAA

DP_scaffold1346 .C...............................T...........G.............C...C.T..GC.......A...TTTA.........C.....

18210 18220 18230 18240 18250 18260 18270 18280 18290 18300

....|....|....|....|....|....|....|....|....|....|....|....|....|....|....|....|....|....|....|....|

LE_scaffold81 CATTGTGACGTATTTTCCAAAGACGTTGGTTTAGTTTTATTAGAAATTGATCCATTTTTATTATACATTTTAATGTTAGTAGTACCTAGCCTTTTGCTAA

DP_scaffold1346 .........A...........TGT.........................G................T........C.....A..T........A......

18310 18320 18330 18340 18350 18360 18370 18380 18390 18400

....|....|....|....|....|....|....|....|....|....|....|....|....|....|....|....|....|....|....|....|

LE_scaffold81 CACATATTTCATAATAGCTATGCTTTAGTCTTGTAAATGTTTACATTTGTAGTAGACATATCAAGTAATCACATGCCCACAGGTATTAAGGCCTTGTTCT

DP_scaffold1346 ..AGC..C.....................T.........................G.C...........A..............................

18410 18420 18430 18440 18450 18460 18470 18480 18490 18500

....|....|....|....|....|....|....|....|....|....|....|....|....|....|....|....|....|....|....|....|

LE_scaffold81 ACTTTAACCATAAGCTGTTTTCCCCAAGTGCAGTTTATTCGTGCTGAATTTGCAGTGCCAGGATAAAGGTCTTTGGCGCTTGCTGAAGGAACATGTTGAA

DP_scaffold1346 G......T.....A.........................T..................A.....G..A.C.......A...............C.CCA..

18510 18520 18530 18540 18550 18560 18570 18580 18590 18600

....|....|....|....|....|....|....|....|....|....|....|....|....|....|....|....|....|....|....|....|

LE_scaffold81 GAAGTATGGTAAATCCAGTGTTTTCACTCAAGCCTACTAATGGTGCTGTGCTGTACCCCAGTCTGGAGAGTGCAAGCACTCACTCAGCTCCCTGTGAATG

DP_scaffold1346 ..G..........A.G....A.......G..A...GG..CCT.....................C...C..........G................C....

18610 18620 18630 18640 18650 18660 18670 18680 18690 18700

....|....|....|....|....|....|....|....|....|....|....|....|....|....|....|....|....|....|....|....|

LE_scaffold81 AACGCCTTGGGTCTGACAGAAAGGTCTGGAAGGAACTTATCCACAGGTACTCTGATCACCTGTATGCTGTAATCAGCTTGGGGTTTAAATATGGGTATCT

DP_scaffold1346 ..........A..................G........GC................G...................G...........G..........C

18710 18720 18730 18740 18750 18760 18770 18780 18790 18800

....|....|....|....|....|....|....|....|....|....|....|....|....|....|....|....|....|....|....|....|

LE_scaffold81 TTGAGCTTTTCTTTTGGGACATATCTGATGTGAGGCAATTGCTGTGTGGAATGGACTTGTATACATGTAAAAGCAGTATGGCTTCCTTTATTAATGCTCA

DP_scaffold1346 .....................................T.................G..........AG.....TGA....A.........A.........

18810 18820 18830 18840 18850 18860 18870 18880 18890 18900

....|....|....|....|....|....|....|....|....|....|....|....|....|....|....|....|....|....|....|....|

LE_scaffold81 GTTATCTTGACTTCTGAGCATTTTCAGTATTGAATCCTGGTTATGGACAATGTATGCTGGTTCTAAGTCAACAATTTTAATGTGGGCTGAACAGCCTGTT

DP_scaffold1346 ..........T....................A......CA............C..A.......C.....................C..............

18910 18920 18930 18940 18950 18960 18970 18980 18990 19000

....|....|....|....|....|....|....|....|....|....|....|....|....|....|....|....|....|....|....|....|

LE_scaffold81 CATGGAGCAGGGTGCTGCGTGCCTCCATCGCTTTTGCAACTGTCAGCCTCTAAGCATGTCATAGTCTGTAAAAAACGATGCAGTGAATGTCTGTTAAGTG

DP_scaffold1346 .G.............C.A..........G..............................TCC............GT.......CT...........C...

19010 19020 19030 19040 19050 19060 19070 19080 19090 19100

....|....|....|....|....|....|....|....|....|....|....|....|....|....|....|....|....|....|....|....|

LE_scaffold81 TTCTTTAGTCTCTCAGCTTTTCCCCTGGAGGACAAATGTGCATGCTGTTATTTGTACCATTTTGTATGGGTTTTGTTTCAGAACAATCCACAGCTGTATT

DP_scaffold1346 ..G.....................................TG...........C.......................C..A...................

19110 19120 19130 19140 19150 19160 19170 19180 19190 19200

....|....|....|....|....|....|....|....|....|....|....|....|....|....|....|....|....|....|....|....|

LE_scaffold81 CTTGTGGTAGAAAGAGAGAAGGGCAAGGTTAAAAAATTGTGCTTTGCATACAGAGTGGGATGTGGTAAGACTTGTCTTAGCTTCTGTGAGAATTTTTTTG

DP_scaffold1346 .................A...............G..G.................T.........................................--..

19210 19220 19230 19240 19250 19260 19270 19280 19290 19300

....|....|....|....|....|....|....|....|....|....|....|....|....|....|....|....|....|....|....|....|

LE_scaffold81 GACTGTCCTTCAGGAAATCTCAGTATCCTGCACAGCTGAGCTTCGCCTTTACACAAACAAAGTTGTACAGTGTTAGTGTGTGATCACTGCTGGCGACATG

DP_scaffold1346 ...A..T....................................T........GT.............TT...C........C............A.TG..

19310 19320 19330 19340 19350 19360 19370 19380 19390 19400

....|....|....|....|....|....|....|....|....|....|....|....|....|....|....|....|....|....|....|....|

LE_scaffold81 ATTTTCATCCTGCAGCTTTGGGAGAGATCTTAAACATATACCTGCAGTACCTAAAGGCACCTGCATGATGAGCAGATAAGAACTGTGACCTGGCATTAGG

DP_scaffold1346 ..........................G..............G........T.G......AT..TG...................................

19410 19420 19430 19440 19450 19460 19470 19480 19490 19500

....|....|....|....|....|....|....|....|....|....|....|....|....|....|....|....|....|....|....|....|

LE_scaffold81 CTTTTTT--CTGTGGGAAGCCGTGACAGGAGAGAAGAGTAAGTCAGAGCTGCTCATGATAGTTTGAATTGCAGAGGAGAGGTGTTCCAGCATCTTTATTT

DP_scaffold1346 .......TT...........T............C.....T.A------------G..T...........................G...A.....C....

19510 19520 19530 19540 19550 19560 19570 19580 19590 19600

....|....|....|....|....|....|....|....|....|....|....|....|....|....|....|....|....|....|....|....|

LE_scaffold81 ATCCAGAGTTTATTAGGAGCTGTTGGTTTTCTGTCTGGGCATGCTATGCTCTTGTGGTAGGTGTGTGTCGGTTTAGACTGTGGCATCTTCCACAGAAATG

DP_scaffold1346 G.............................T.C........C...C.........A..........A..T...A.........TG...............

19610 19620 19630 19640 19650 19660 19670 19680 19690 19700

....|....|....|....|....|....|....|....|....|....|....|....|....|....|....|....|....|....|....|....|

LE_scaffold81 GGATAAATCCT------GATTGCAGATGGCAAGAACCAGCCAAAAAGATATCACTTACAGTTTCTCTACACAGCTGAAAGCAGGATGCTAGCATTCAATG

DP_scaffold1346 ...C....T..TCTGCT.....G......T........A.T.......C....T.............G....A............CA...C.........

19710 19720 19730 19740 19750 19760 19770 19780 19790 19800

....|....|....|....|....|....|....|....|....|....|....|....|....|....|....|....|....|....|....|....|

LE_scaffold81 GGCAGTTAAACAGGAAAGCGATTTCCCATTTCTAGAAATGTATTAGTACTTTTTTCATCCCCATATTTTCTACAGAACTTT-ATAAAAAACCTGGAGGCT

DP_scaffold1346 ..G..........A....TA...................TC....A....CCC.---C.......................T....GC..A....T..T.

19810 19820 19830 19840 19850 19860 19870 19880 19890 19900

....|....|....|....|....|....|....|....|....|....|....|....|....|....|....|....|....|....|....|....|

LE_scaffold81 TATGATCCAATGCAGCAGCATACTGAAGTAAGTACTTGCTTAAGTTTAAGCAGATAGTCAGTCCTTTTGAAACCTGAAGATTAAACCCTGACACCAGTGG

DP_scaffold1346 .....C.................C----..................G...T......G....................T...........G.........

19910 19920 19930 19940 19950 19960 19970 19980 19990 20000

....|....|....|....|....|....|....|....|....|....|....|....|....|....|....|....|....|....|....|....|

LE_scaffold81 AATGAGTAGCAGGCTCTCATTGACTTCAGTGGGCCAGGATTCAATCTGATTGCCTAATTAAATGATCCTGTTCTTCAGAAAAAGTATTAGAAAGATAGAT

DP_scaffold1346 .G..........A...C......T.......A...............A...C.......G...T.................................A..

20010 20020 20030 20040 20050 20060 20070 20080 20090 20100

....|....|....|....|....|....|....|....|....|....|....|....|....|....|....|....|....|....|....|....|

LE_scaffold81 GTTCAGTAATTGCTTCGAGTATTTTTACTGAAATCTATTTGCTTAGGATAAAGCTTTTCCACGACAGTGAAATAGCTACTTTTTTGATTTCAAACTGTGC

DP_scaffold1346 ...............T.........C.A..............................G..T..G.........A......C.G........G.......

20110 20120 20130 20140 20150 20160 20170 20180 20190 20200

....|....|....|....|....|....|....|....|....|....|....|....|....|....|....|....|....|....|....|....|

LE_scaffold81 AAGAAATTAATTTGAAGGTGTGGTAGTACGTTTGTGGACTAT----------------------------------------------------------

DP_scaffold1346 ....G...............A.A...................CATGAAGCTGGTGAAGGGTCTGGAGCACAGGCCTTATGAGGAGCGGCTGAGGGAACTG

20210 20220 20230 20240 20250 20260 20270 20280 20290 20300

....|....|....|....|....|....|....|....|....|....|....|....|....|....|....|....|....|....|....|....|

LE_scaffold81 ----------------------------------------------------------------------------------------------------

DP_scaffold1346 GGGTTGTTTAGCCTAGAGAAGAGGAGGCTGAGGGGAGACCTTATCGCTCTCTACAACTACCTGAAAGGAGGCTGTAGTGAGGTGGGTGTTGGTCTCTTCT

20310 20320 20330 20340 20350 20360 20370 20380 20390 20400

....|....|....|....|....|....|....|....|....|....|....|....|....|....|....|....|....|....|....|....|

LE_scaffold81 ----------------------------------------------------------------------------------------------------

DP_scaffold1346 CCCATGTAGTTAGCGATAGGACGAGAGGAAATGGGCTCAGGCTGCACCAGGGGAGGTTTAGGTTGGAAATTAGGAGAAATTTCTTCACGGAAAGGGTAGT

20410 20420 20430 20440 20450 20460 20470 20480 20490 20500

....|....|....|....|....|....|....|....|....|....|....|....|....|....|....|....|....|....|....|....|

LE_scaffold81 ----------------------------------------------------------------------------------------------------

DP_scaffold1346 CAAGCATTGGAACAGGCTGCCCAGAGAGGTGGTGGAGTCCCCATCCCTGGAAGTGTTCAAAAAACGGGTAGATGTAGCACTTCAGGACATGGTTTAGTCT

20510 20520 20530 20540 20550 20560 20570 20580 20590 20600

....|....|....|....|....|....|....|....|....|....|....|....|....|....|....|....|....|....|....|....|

LE_scaffold81 ----------------------------------------------------------------------------------------------------

DP_scaffold1346 AGTCTACCCTTAATTGGTTTAGTGTGGGCTTGGTAATGTTAGGTTAATGGTTGGACTGGATGATCTTAAAGGTCTTTTCCAAACTAAATGATTCTATGCT

20610 20620 20630 20640 20650 20660 20670 20680 20690 20700

....|....|....|....|....|....|....|....|....|....|....|....|....|....|....|....|....|....|....|....|

LE_scaffold81 --------------------TCTCTTTAGTTAGTGATAGTGGGCTCCCTTGTTGTGGTGAATCAGTTTCCTTCATTTGCTTGCTTTTCTGTCTCA--TTG

DP_scaffold1346 TCTATGATTCTATGATTTAT.....C............T...A....................T..............T.CA.............GAC..

20710 20720 20730 20740 20750 20760 20770 20780 20790 20800

....|....|....|....|....|....|....|....|....|....|....|....|....|....|....|....|....|....|....|....|

LE_scaffold81 TTGAAAACAAAGATGGCAAAAGATGTTTAAAATACTTCTGCAAAATCAACTCTTAAAATAACATTTTAGTTTTTTACATTCAGACCTGAATACAATTATT

DP_scaffold1346 ......T.....G....T...............C.....T........C........G.............................A............

20810 20820 20830 20840 20850 20860 20870 20880 20890 20900

....|....|....|....|....|....|....|....|....|....|....|....|....|....|....|....|....|....|....|....|

LE_scaffold81 TAGAA-AACAAAAGCCCGGAGAAGGAAGATCTTTCTCATATGAACAATTTCTCCGTTATTCTTTCTGAGCTAATTGGCCTAATGCAAAACTCCTTC---C

DP_scaffold1346 .....G..A.......TT.G...A..G......------G...G.......C.T.......C......T.............A...........CTATAT

20910 20920 20930 20940 20950 20960 20970 20980 20990 21000

....|....|....|....|....|....|....|....|....|....|....|....|....|....|....|....|....|....|....|....|

LE_scaffold81 ATTAAGAGGAAGAAAAGGGACTTTTTTTTTTCCTGCTTCAAGGAGAAAAAA--CATGATTTGGCAGCCTCTATTCAAATAAATGGTTGTATTTACTACTT

DP_scaffold1346 T..CCATTA.GA.G.................---.T...............AT.............TT.........G.....A................

21010 21020 21030 21040 21050 21060 21070 21080 21090 21100

....|....|....|....|....|....|....|....|....|....|....|....|....|....|....|....|....|....|....|....|

LE_scaffold81 TTGCACTGGAGAAATATTTTGCATTTGTAGAATTAGTCCTGAAGATTTGACCAGGTTTTCTGAATGAGGTGAC--ATGTGTGTTTAAAAAAAAAAAAAAA

DP_scaffold1346 .............T.................................................GC.....A..TT.CA...T...TGT............

21110 21120 21130

....|....|....|....|....|....|....|..

LE_scaffold81 TCAAACCCCGAAGTTGCCTGAACAAGTCTCACTGACA

DP_scaffold1346 AA...AAAAA.....T..A.........C.....C.G

**(iii) ePaV *Corvus brachyrhynchos* and ePaV *Acanthisitta chloris***

10 20 30 40 50 60 70 80 90 100

....|....|....|....|....|....|....|....|....|....|....|....|....|....|....|....|....|....|....|....|

AC_scaffold61 GGAACAACTGATGCTGACTCTAAAGTCACAAAAAAACCCCATACCAGAAGATAACATTATTCAAGGTATTGTTACAAGGTGGAAATGTAGCTACCAGTGA

RF_scaffold6994 ..............CT..AT.G......TGG..T..-AG...............AGA....A................TA......A.......TG....

110 120 130 140 150 160 170 180 190 200

....|....|....|....|....|....|....|....|....|....|....|....|....|....|....|....|....|....|....|....|

AC_scaffold61 TGGTCTAGACATGGCAGGACAGACCTCAGAGCAGATTAACTTTCTTTACTAAGAGAGCAAGGAAGGCACGTTAAACCCCCAGTGTGAAACATGCTTCAGT

RF_scaffold6994 .......................GG.............................C.........AATG.-...C.TT.---A...........A......

210 220 230 240 250 260 270 280 290 300

....|....|....|....|....|....|....|....|....|....|....|....|....|....|....|....|....|....|....|....|

AC_scaffold61 ACCGACAGTTCTCTCACCAAATGTTCTGAGTCTACCTAAATGATGGATACAGATTTCCCTTTTCAAGGCTTTCTTCACAGCCAGGAGGGGCACGTATTTA

RF_scaffold6994 ..T......C.................CT...........C...........................T.....C........A.....C.T.T......

310 320 330 340 350 360 370 380 390 400

....|....|....|....|....|....|....|....|....|....|....|....|....|....|....|....|....|....|....|....|

AC_scaffold61 AATATAAGTACATACATATGTAACTGTAAGTTTAGGTGAAAGATGATCTTCTCACATGTTTTCTTGGCTCCTAGGAGACACCAAATAGCAAGAAACTAAT

RF_scaffold6994 ........................CA...A.....A......T.T.....................T..T------.G..........TG...G......

410 420 430 440 450 460 470 480 490 500

....|....|....|....|....|....|....|....|....|....|....|....|....|....|....|....|....|....|....|....|

AC_scaffold61 AATCGAAGCACAAGAGTTGGCACTACTCATTTTGTGTGTTGCAATTTAAAGGTCTAAAGATCTAAATCCACTAAGTGGCTGTGAAAACCTGAACTACAGA

RF_scaffold6994 ....A........A.C....TG.C....GC.....TG.C....GCG.-...ACA.....G.......-----------A.C...................

510 520 530 540 550 560 570 580 590 600

....|....|....|....|....|....|....|....|....|....|....|....|....|....|....|....|....|....|....|....|

AC_scaffold61 TTCCAGTTTTATTTTATATCTTACGTGATACTGAATTTTTCGTGTAAACAGCTTTTGAAGTTTCACGGTAGGTTTTGCATACAGGAGAAAAAGGTTTGTC

RF_scaffold6994 .......C.......C.G.....GA..........C..C.A...............AC........AT...AC..............G...G.......T

610 620 630 640 650 660 670 680 690 700

....|....|....|....|....|....|....|....|....|....|....|....|....|....|....|....|....|....|....|....|

AC_scaffold61 TGGTTCAAGACTCAGTTCTCTAATTATCTTTCTTTCACTTCCTCTGCTGATTTTATTTCTAGTAGAGTGAGAAGCCTTAAACTACTCCCTGAGGCTTTCT

RF_scaffold6994 .........................G.----A...........................................T......A.........A...CC..

710 720 730 740 750 760 770 780 790 800

....|....|....|....|....|....|....|....|....|....|....|....|....|....|....|....|....|....|....|....|

AC_scaffold61 TCTTGATCCCAAGCTTTGTAAAATGTAGGTGGTTTCCTTGAGATGATGGCTTTTATAAGCTGTGAGTTAATTGTGATTAGAGCAACAGCCACATCTGGAA

RF_scaffold6994 ............A........C........-........A.C......CT.............C...................................T

810 820 830 840 850 860 870 880 890 900

....|....|....|....|....|....|....|....|....|....|....|....|....|....|....|....|....|....|....|....|

AC_scaffold61 CTCCTCTTCTGCTAAAGCATCCTTGGAGAACTGGCATGAAAGTTGTTCTCAACTGTAAAATATGTGATTGGGAAATATTTTTCAAAACTTAAAAATAAGC

RF_scaffold6994 ..T........A.G.C.....T..................T........G......-.........G....A..........T.C....A..........

910 920 930 940 950 960 970 980 990 1000

....|....|....|....|....|....|....|....|....|....|....|....|....|....|....|....|....|....|....|....|

AC_scaffold61 AAAATTAAATTGTTTAA-TTGGTTCTCAGCAGTTGCTACATGTAGGGTTGTGAGTTAACTTTCAATTCATTAGTAGATTTTGCCTGTCAAATAAGCTCAC

RF_scaffold6994 .....CT..G.......T...C.................G.T....A....A............G....C...C.....C...............G....

1010 1020 1030 1040 1050 1060 1070 1080 1090 1100

....|....|....|....|....|....|....|....|....|....|....|....|....|....|....|....|....|....|....|....|

AC_scaffold61 AAAATTTCCAGTGAAGGGCAAGCCAT--AACCTTTTGGTATATCAGGGATCTTTAGGGTACTTGGCTTTCAATTTCTGACCTACTATACATAAGTCTCAG

RF_scaffold6994 .T...............A...A.TG.CAC...........C.C.T.A-------.A.A.C.C...A.C..G.......G.T.----C.T..CT..G....

1110 1120 1130 1140 1150 1160 1170 1180 1190 1200

....|....|....|....|....|....|....|....|....|....|....|....|....|....|....|....|....|....|....|....|

AC_scaffold61 TGGGTGCCCTCCCTACCTCCTCAGAAATATTGCCAGGGTACATTTTTCCTTTTCCAAAGATTACATAGAAATTGAAAATCATGGCTTTAAAATATCTAGC

RF_scaffold6994 ..........G.............G..................G.....G.....T..A.........C....AC..............G.C........

1210 1220 1230 1240 1250 1260 1270 1280 1290 1300

....|....|....|....|....|....|....|....|....|....|....|....|....|....|....|....|....|....|....|....|

AC_scaffold61 ATTAATTATGCAGCTTTCTCATGAGCTAGGACTTCTTGC--CAGTGAGTGATAAATCCCTCTCTCCCTTTCATCCAGAATTTGACTAC------------

RF_scaffold6994 ...................T......C....G..T....CA.......G...G...........T...................G.G.TAAGCTCTCCTG

1310 1320 1330 1340 1350 1360 1370 1380 1390 1400

....|....|....|....|....|....|....|....|....|....|....|....|....|....|....|....|....|....|....|....|

AC_scaffold61 TTTAGCAGGGTACTAGAGCACTCTAAAACTGCTTCCATTTGAAAAGAGAGTATTATCACTCCTAACTCCCTAATTAGAACCATGACACTATTTTAAATAC

RF_scaffold6994 ........A.......G.....TC--G...A............G.........C.C..........C.....T........T...T....C........T

1410 1420 1430 1440 1450 1460 1470 1480 1490 1500

....|....|....|....|....|....|....|....|....|....|....|....|....|....|....|....|....|....|....|....|

AC_scaffold61 TATTTTACACTATGATTTTTCATTC------TGTTTAATTTGTGTTAC-TAATTAACTTGCTCTCCAAGCAAGGTTATCAATTGTTTACTGGTCAGGAAA

RF_scaffold6994 ............G......G.T..TCAGGCA.T.CC.....AAT...TG.T.....T.A..............A...G......C..........-----

1510 1520 1530 1540 1550 1560 1570 1580 1590 1600

....|....|....|....|....|....|....|....|....|....|....|....|....|....|....|....|....|....|....|....|

AC_scaffold61 AGGCACTCTTGGGTTTTACTGAGCACAGCTTAGTGTTACTATAATTGCAGAGTATGTCATACCAACAGTGAGCCTAAATTGTG--AATTCTGCTTTTTGG

RF_scaffold6994 --...TC............G..AA..G..............C....C....T...C................A..........TAT.....A..C.....

1610 1620 1630 1640 1650 1660 1670 1680 1690 1700

....|....|....|....|....|....|....|....|....|....|....|....|....|....|....|....|....|....|....|....|

AC_scaffold61 TGGATATTTTCAACTTGAGCATTGTTTTATTTCTAGTCTATAAATACTGAAAGACCCTTTCATATGAATTACACAGCAGAAAAGAAGTGCTCTCTTGCCT

RF_scaffold6994 ...................................T...G............TG...CAA.............T......G..C.......T....T...

1710 1720 1730 1740 1750 1760 1770 1780 1790 1800

....|....|....|....|....|....|....|....|....|....|....|....|....|....|....|....|....|....|....|....|

AC_scaffold61 TTGGACTAGACTGAGAGTTTTCTTTCAGTTGCAGAGGGGAAGAACTGTAAAACACCTGATCAGCCTGGCTTCAGCTCCAGCAGGAGTAAAAATTGCCAGA

RF_scaffold6994 ...............---.............A...........G.............-----............-NNNNNNN....G.GG..........

1810 1820 1830 1840 1850 1860 1870 1880 1890 1900

....|....|....|....|....|....|....|....|....|....|....|....|....|....|....|....|....|....|....|....|

AC_scaffold61 TA-----TCTATCTTCTGTCAGCTGGAATAGCTGCCTGTTTGCAAAGTTGGTATAGAGCCTACCACAAGAAAACCTAAGAGTTTACTTTCTCCTCACGAC

RF_scaffold6994 .GATTTC..........................T...AG..A....T......C..-................T.....CA...............G...

1910 1920 1930 1940 1950 1960 1970 1980 1990 2000
[truncated: 51,622 more chars]
