# Supplementary material for: Low frequency of paleoviral infiltration across the avian phylogeny
Source: Genome Biol. 2014 Dec 11;15(12):539. doi: 10.1186/s13059-014-0539-3 (PMC4272516; doi:10.1186/s13059-014-0539-3)
Supplement: Additional file 5: Figure S3 — Alignment of a hepadnaviral element in the genome of mallard duck with orthologous (and partial) sequences found in the genomes of chicken and turkey. Note that we found a 94% match to the 5′ conserved region (marked as C) in turkey, and a 39% match to the orthologous chicken sequence; 45% of the central 12,042-bp virus-like sequence matched the 5′ variable region (marked as V). The relatively conserved nucleotides in chicken showing virus-like characteristics are boxed. Asterisks indicate the conserved nucleotides in the alignment, dashes denote deletions. [file 13059_2014_539_MOESM5_ESM.pdf]

```
                          10            6402   10871
                           |     C         |  V  |
                    ** *******     **** *****
Duck scaffold490    TTGCAGGTCT  //  TTTGATTATC  //  GAAAATGTCCAGTGTCTTAGGCCAAATATTTCTTGGACTAATAATTTAGTAGCATGTTTTTTCTTATT
Chicken chrZ        TTTCAGGTCT  //  TTTGGTTATC  //  GCCAAAGAC-AATAATAAATGCTTGAAATTTGT--GCTTGATTTCTTCAAATCAAGCTGTTCTTC---
Turkey chrZ         TTTCAGGTCT  //  TTTGATTATC  //  ---------------------------------------------------------------------

Duck scaffold490    AACAAAAATCCTAGAAATGCCAAGGAAGATAGACTTGCTGTGGACTCGGTTTTCCTGCAGGCCAAATGTGTTGTCCTTTC-CAAAGTACTGCT---CTC
Chicken chrZ        --CATATATTTCAAAGA--CCAA--AATACTAATTTGATTTTCCTTAGCTGTAAATATACGTCAA-TGTACTGTTGTGTAATTATGTTCTGCATCACTG
Turkey chrZ         ----------------------------------------------------------------------------------------------------

Duck scaffold490    CAAACCT-CT-CAGCACTTTT-CATGACTTCACC-TGGACATGCCCAGGATTTCTTTGGATGTGTC--TCAGGCTTTTTATCATATTCCTCTTGGTGT
Chicken chrZ        CAAATTTGCTTTAGTAAGACTACAGGACTAAACCACAGAGAAGGGGAAAAGTTACCAGAAATAGCCATTTAAAACTTCTTTGACTGCCTTCTTTG---
Turkey chrZ         ---------------------------------------------------------------------------------------------------

Duck scaffold490    TGCTAGTGATACTCAGCTATCTGTATCTGACAGAGAACTGATCTACTATTTTTGCAAA----GCTTTAGTGGGGGTTGGTATCAGCCCT--TTTCTCC
Chicken chrZ        -ACTGATAATAGTCATTTGTGCTAATGCAACATGTGACATTATTATATATGTAACAAGTTATGCACTAGTGACAGT-GGCATAAGACAAAGCTTAAAC
Turkey chrZ         ---------------------------------------------------------------------------------------------------

Duck scaffold490    TCCAGGTCTTCACAGCTGCCTTCACAGACAAAATTTCTTGTCACTGGAACTTTTGCTTATGACTTTCAGTACTTGAAGGGGGCTTATAAAAAA-AGAT
Chicken chrZ        ACCAGGTCATCTGCACCACCAAAAAAAAGAACAACAGAGGTAAAGAGCAGTGCTGGGTGGAGGCTGTTGTGATGCAGTGGAACGGAGGGAAAATAAAT
Turkey chrZ         ---------------------------------------------------------------------------------------------------

Duck scaffold490    GGAGAACAACTTTTTGCTGGATCCGATAATGACAGGATTAGAGGGAATGGTCTTAAAGTAGAAGATGGAAGATTTAGATTACAGTTTAGGAGAAAACT
Chicken chrZ        GGAAAAAAATGATATGGGCAGGCCTG-AAGGACAGT-TTTGCGTGAATTGTATTTGAAAAGCTTTTCTTTGAAAAAGACTA--GTAAAGCATGAAAG-
Turkey chrZ         ---------------------------------------------------------------------------------------------------

                    ____________________________________________ unknown intron _______________
Duck scaffold490    TCACTCAGAGGGTGGTGAGGCACTGGAACAGGTTGCCCAGCCCAGAGAGGCTGTGGGT--GCCCCATCCCTGGAGAT-GTTCAAGACTGGGTTAGATG
Chicken chrZ        -CATTAAGTGGA-AGTCTGCAATTGTGGCATGTCATTCTGGGCCATTTCACTTCAATTTAAATGTATTCAAGGTGAGAGACTGCTGCAGTGCAGGATG
Turkey chrZ         ---------------------------------------------------------------------------------------------------

Duck scaffold490    AGGCCCTGAGCAACCTGATGTAGGGGTGGCATCCCTGTCCATGGCAGGGGGGTTG--GTACTAGGCGATCCTTGAAGTCCCTTTCCAACTC------G
Chicken chrZ        AAAGCTTCAGGACCTGGGAGCAAAGGTTGGAACCTGGGAAAGAGAAAGGCAGTCAAAGGATTGTTTTAACCTCTGAGCGCCTTGCATGTTCTCCACCG
Turkey chrZ         ---------------------------------------------------------------------------------------------------

Duck scaffold490    AGCCATTCTATGATTATATGATTCTGTGATTCTATTACTTCCTCC-TCTGTTACCCAAGAGCTTATTACCTTAACATAGTTTGCCACAGCGTTAGCTG
Chicken chrZ        TTTTGTTCTATCAGTGTATGAG-CAACCTTCCTCTCAGCACTTCAGTTTTTTTGTGGAGAGCATGG-ACC--AATCTCCATTGGAACA-CAATAACTC
Turkey chrZ         ---------------------------------------------------------------------------------------------------

Duck scaffold490    TTTTCTACAAGCCATAGGAGTAAGAATAAACTTGGACAAGAATACATGGTTACCATCACATGAGATTCAATATTTGGGACAGTTATTCAAGGATTGGA
Chicken chrZ        TACCTCACCAGCTGTTCTTGGAAAG-------TGAAAAAGAAGGCAAAAGTGGTTTCAAGAACAATATACAGCCTGAGCCTTCTTTAAAATGCTTGAA
Turkey chrZ         ---------------------------------------------------------------------------------------------------

                                                                                        11904       19401
                                                                                            |   V   |
                                                                                                  *** **** *
Duck scaffold490    CAGTTGAAGTTCCAACTGATCAATGGATGAAATTAAAGACTATTATTAGAACAACAAAACCAGGAGTTCAATATGATTATAAA  //  AAACAAATCT
Chicken chrZ        TATTTGATTTTGAAACTGA--AATGGCTGA-------GCACATTTCTATAGTATCAAGACTGGGAAAAAAAAAAAAAAGGAAC  //  AAACAAATTT
Turkey chrZ         -----------------------------------------------------------------------------------  //  AAAAAAATTT

                     C
                        **********
Duck scaffold490    //  GCTGTGACAC
Chicken chrZ        //  GCTGTGACAC
Turkey chrZ         //  GCTGTGACAC
```
