# Supplementary material for: Low frequency of paleoviral infiltration across the avian phylogeny
Source: Genome Biol. 2014 Dec 11;15(12):539. doi: 10.1186/s13059-014-0539-3 (PMC4272516; doi:10.1186/s13059-014-0539-3)
Supplement: Additional file 2: Figure S1 — Phylogenetic tree of endogenous retroviruses (ERVs). The tree was inferred using the conserved motif ‘DTGA-YMDD’ within the Pro-Pol region of retroviruses (approximately 320 amino acids in length, although this differs among retrovirus genera). Bootstrap values lower than 70% are not shown; single asterisks indicate values higher than 70%, while double asterisks indicate values higher than 90%. Branch lengths are drawn to a scale of amino acid substitutions per site (subs/site). The tree is midpoint rooted for purposes of clarity only. The host name indicates the species from which the ERV was obtained. Exogenous retroviruses are highlighted using family names. ERVs of alligator, turtle and lizard origin are also highlighted. [file 13059_2014_539_MOESM2_ESM.pdf]

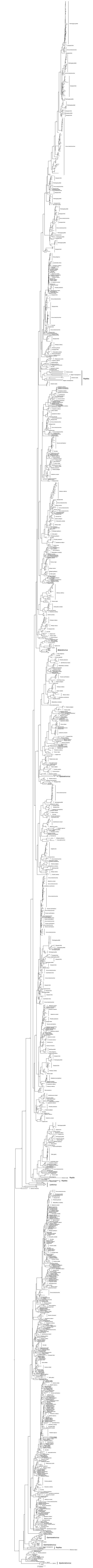
ALV
Alpharetrovirus
Gammaretrovirus
Reptiles
WEHV1
WEHV2
Epsilonretrovirus
